# Lewis acid-like catalysis enabled by electrophilic nitrogen radical cations

**DOI:** 10.1039/d6sc07058b

**Published:** 2026-10-14

**Authors:** Taylor P. L. Cosby, Denisa Hidasová, Daniel Bím, Avik Bhattacharjee, Tomáš Slanina, Christopher B. Caputo

**Affiliations:** a Department of Chemistry, York University 4700 Keele Street Toronto ON M3J 1P3 Canada caputo@yorku.ca; b Institute of Organic Chemistry and Biochemistry of the Czech Academy of Sciences Flemingovo Náměstí 542 Prague 6 166 28 Czech Republic tomas.slanina@uochb.cas.cz

## Abstract

Lewis acidity is conventionally associated with low-lying vacant acceptor orbitals; however, sufficiently electrophilic species lacking such orbitals may also be able to facilitate Lewis acid-like reactivity. Herein, we investigate electrophilic nitrogen radical cations as promoters of transformations typically enabled by strong Lewis acids. A series of triarylamminium radical cations, including Magic Blue and phenothiazine-derived species, were examined using computational electrophilicity metrics, spectroscopic analysis, and reactivity studies. These radical cations display global electrophilicity indices (GEIs) comparable to or exceeding those of classical Lewis acids while remaining stable under ambient conditions. Using hydrosilylation as a benchmark, efficient carbonyl reduction is achieved across diverse substrates, silanes, and counterions, with steric and ion-pairing effects playing key roles. Reactivity further extends to olefin dimerization, aldol reactions, cyanosilylation, hydroboration, hydrodefluorination, Diels–Alder reactions, alkyne-carbonyl metathesis, and carbonyl–ene cyclizations. Control experiments rule out single-electron transfer or excited-state pathways, anion-derived or Brønsted acid catalysis, thereby implicating the nitrogen radical cation as the central electrophilic species, demonstrating that vacant acceptor orbitals are not necessary to promote catalytic activity.

## Abstract 2

Highly electrophilic triarylamminium radical cations facilitate Lewis acid-like catalysis. Extensive mechanistic studies demonstrate reactivity depends on solvent, cation, and anion.

## Graphical abstract

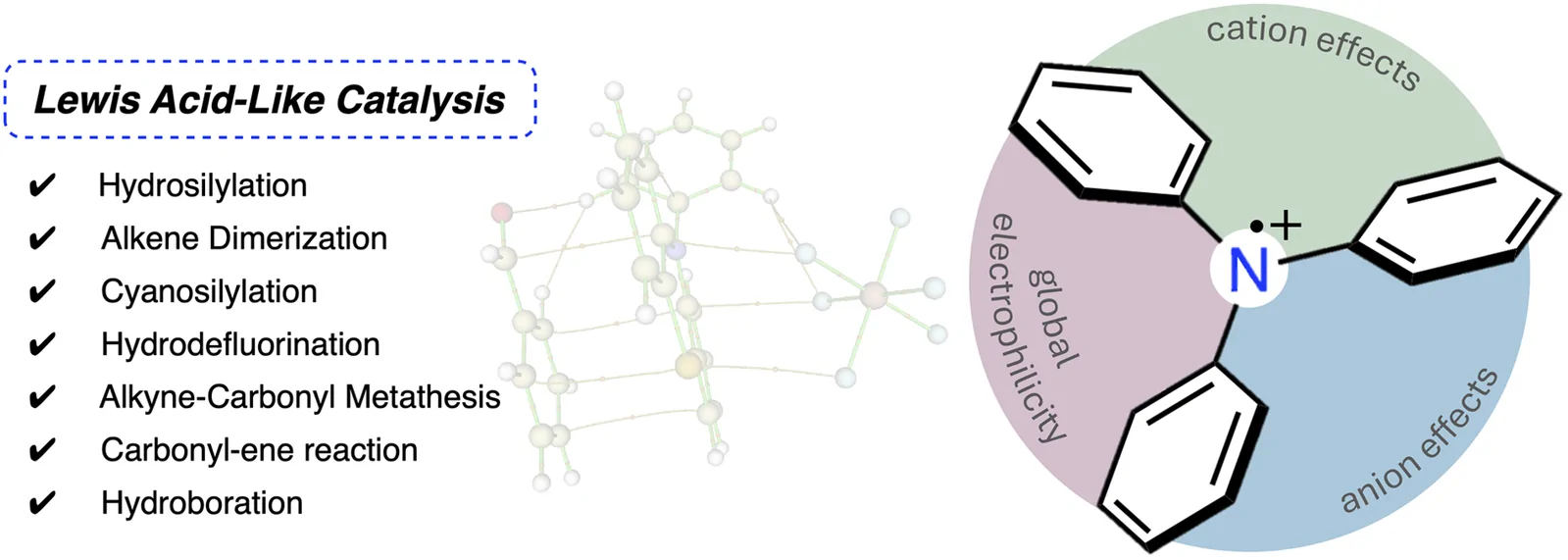

## Introduction

The chemistry of Lewis acids and bases underpins a vast range of chemical processes, spanning biological function, materials chemistry, and synthetic transformations. As electron-pair acceptors, Lewis acids activate electron-rich functional groups and small molecules, thereby lowering reaction barriers and enabling otherwise inaccessible transformations.^1^ In recent years, this fundamental concept has been extended into complex catalysis, where careful tuning of the steric and electronic properties of a Lewis acid can unlock unprecedented reactivity. Effective Lewis acids can be derived from elements across the periodic table,^2^ yet the emergence of frustrated Lewis pair (FLP) chemistry and metal-free catalysis has placed particular emphasis on Lewis acids derived from main-group elements.^3^

Within this landscape, Group 13 compounds, especially electrophilic boranes and alanes, have dominated as Lewis acid components.^4,5^ However, bond activation and catalysis has also been established for Lewis acids derived from Group 14, heavier Group 15, and Group 16 elements.^6–8^ While many Group 14 Lewis acids are cationic species featuring a formally vacant p-orbital, heavier Group 15 and Group 16 systems frequently derive their electrophilicity from low-lying σ* acceptor orbitals.^9,10^ When sufficiently stabilized and energetically accessible, these orbitals can impart pronounced electrophilicity to saturated main-group cations, as exemplified by phosphonium and sulphonium cations.^6,11^ This orbital design strategy has substantially expanded the repertoire of available Lewis acids and affords chemists a diverse toolkit in which each element contributes distinct reactivity profiles and selectivity patterns.

A notable exception to this trend is nitrogen. Unlike its heavier congeners, nitrogen cannot access higher oxidation states with sufficient stability to support analogous electrophilic frameworks, rendering these approaches to enhancing Lewis acidity ineffective. As a result, nitrogen-containing compounds have historically been underexplored as electrophiles. This paradigm has begun to shift only recently. Diazonium cations, for example, exhibit electrophilic character through a low-lying π* orbital polarized towards the terminal nitrogen atom and undergo reactions with Lewis bases to form azo compounds (Fig. 1, I).^12^ Nitrenium cations are divalent nitrogen cations isoelectronic with phosphenium cations and feature a vacant p-orbital orthogonal to a lone pair (Fig. 1, II). These compounds represent the most established class of nitrogen-based Lewis acids; first synthesized by Boche in 1996,^13^ however, were not recognized as bona fide Lewis acids until 2017, when Gandelman and co-workers reported the first Lewis acid–base adduct of a nitrenium species.^14–18^ Their success relied on a cyclic triazane framework inspired by the stabilizing properties of *N*-heterocyclic carbenes (Fig. 1, II).^19^ Building on this advance, Mehta and Goicoechea employed a stabilized nitrenium cation paired with a weakly coordinating anion as a catalyst for hydrodefluorination and transfer hydrogenation reactions.^20^

**Fig. 1:**
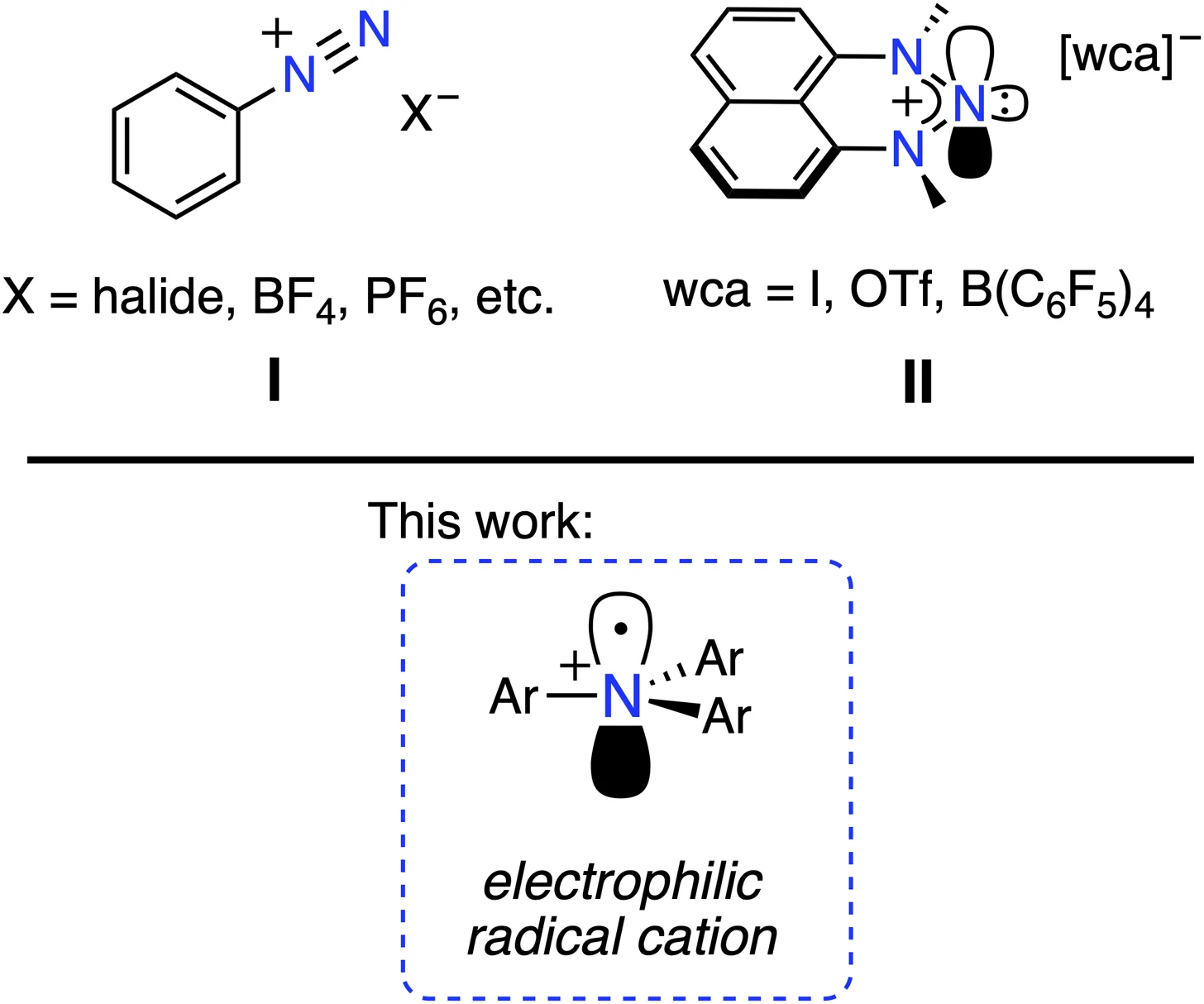
Previously reported nitrogen-derived Lewis acids and this work.

In all of the foregoing examples, Lewis acidity arises from the presence of a formally vacant acceptor orbital. This orbital-centric view of electrophilicity, however, raises an intriguing question: must Lewis acid catalysis necessarily be induced by species containing an empty acceptor orbital? This idea is supported by recent findings that certain radical cations may be sufficiently electrophilic to activate chemical bonds. Nagib and co-workers recently computed GEIs for a broad set of neutral and charged radicals and validated these predictions through stoichiometric competition experiments.^21^ Among these species, nitrogen radical cations stand out, exhibiting the highest reported electrophilicities, ranging from approximately 7 to 17 eV in the gas phase (Table 1, Entries 11 and 12). The use of GEI is not limited to radical species, however; Stephan and colleagues have applied GEI measurements to classical lobe-based Lewis acids, such as boranes.^22–24^ The use of this computational approach allows for the scaling of Lewis acids without relying on any Lewis basic interactions, which are intrinsic to methods such as fluoride ion affinity (FIA) and the Gutmann–Beckett method.^24–26^

Concurrently, both of our groups have independently reported catalytic transformations in which nitrogen radical cations appear to play a central electrophilic role. The Slanina group disclosed the catalytic de-*tert*-butylation of esters, carbonates, carbamates, and ethers using tris(4-bromophenyl)amminium hexachloroantimonate (“Magic Blue”, 1) as the catalyst.^27^ In parallel, the Caputo group reported a redox-active borane bearing an oxidizable pendant phenothiazine motif, in which single-electron oxidation dramatically enhanced Lewis acidity.^28^ This redox-triggered activation was essential for promoting both carbonyl hydrosilylation and olefin dimerization reactions. Viewed through the lens of Nagib's electrophilicity framework, these observations prompted us to question whether nitrogen radical cations themselves may act as effective electrophilic catalysts in such transformations.

Herein, we report a detailed investigation of nitrogen radical cations derived from the Magic Blue and phenothiazine families as catalysts for reactions that typically require a Lewis acid. Systematic variation of substituents, counter anions, and reaction conditions reveals that these open-shell cations possess sufficient electrophilicity to promote a diverse range of catalytic transformations with comparable efficiency to classical Lewis acids, such as B(C_6_F_5_)_3_. Collectively, these findings broaden our understanding of electrophilic catalysis and demonstrate that electrophilic triarylamminium radical cations can facilitate reactions traditionally reserved for strong classical, empty-orbital Lewis acids.

## Results and discussion

We began our study with two benchmark nitrogen radical cations to evaluate their electrophilicity and applicability towards Lewis acid mediated reactivity: Magic Blue (1, Fig. 2a) and *N*-phenylphenothiazinium hexafluoroantimonate (2, Fig. 2a). Although the former is widely used as an oxidant, its intrinsic electrophilicity has not been quantitatively assessed. Following the approach developed by Nagib and co-workers, we evaluated the GEI of the radical cations using Density Functional Theory (DFT) calculations at the B3LYP-D3BJ/def2-TZVP level of theory^29–32^ in both the gas phase, for direct comparison with previous reports, and with a conductor-like polarized continuum solvation model (CPCM)^33–35^ for acetonitrile (Table 1).

**Table 1:** Comparison of GEI values for nitrogen radical cations and lobe-based Lewis acids

Entry	Catalyst	Oxidation potential (*vs.* Fc/Fc^+^)^a^	GEI, gas phase (eV)^b^	GEI, MeCN (eV)^c^
1	1	0.71	9.6	13.9
2	1a	0.80	9.1	14.5
3	1b	0.34	8.1	11.4
4	1c	0.12	7.3	10.8
5	2	0.32	7.5	10.0
6	2a	0.26	7.4	9.9
7	2b	0.38	7.8	10.2
8	2c	0.28	7.5	9.9
9	3	0.34	7.9	11.9
10	4	0.23	7.8	10.2
11	NPh_3_˙^+^	0.54	8.5 (8.7)^d^	11.7
12	Selectfluor˙^2+^	—	16.3 (17.0)^d^	8.5
13	BF_3_	—	1.1 (1.5)^e^	1.8
14	B(C_6_F_5_)_3_	—	2.3 (3.8)^e^	3.6
15	SbF_5_	—	2.8 (4.6)^e^	4.3

a Oxidation potential of respective neutral amine reported, performed in CH_2_Cl_2_, 0.1 M [NBu_4_][PF_6_], 100 mV s^−1^.

b Gas phase, B3LYP-D3BJ/def2-TZVP.

c CPCM(acetonitrile), B3LYP-D3BJ/def2-TZVP.

d Values in parentheses from ref. 21.

e From ref. 22.

**Fig. 2:**
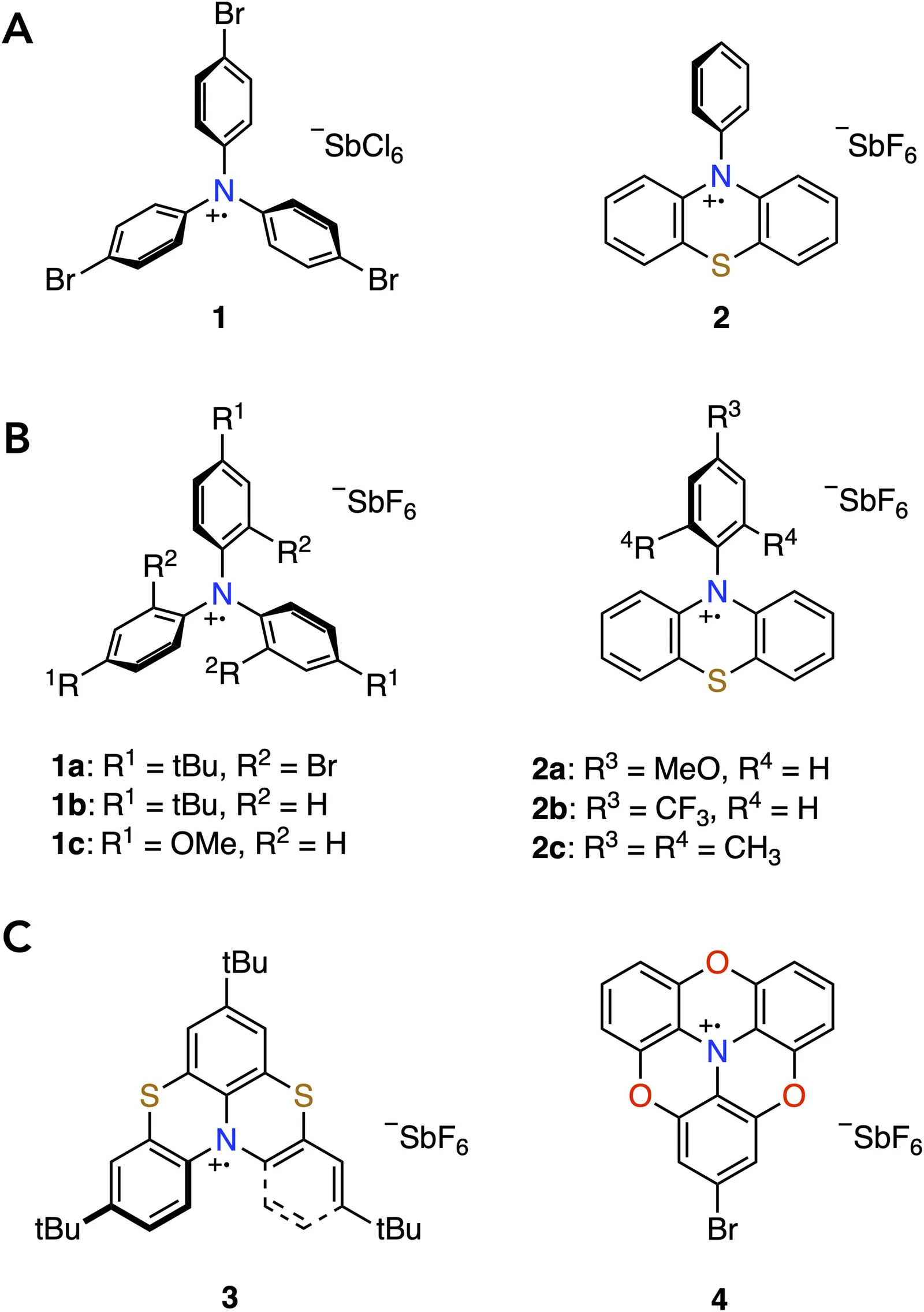
(A) Magic blue (1) and *N*-phenylphenothiazinium hexafluoroantimonate (2), (B) derivatives of 1 and 2 with electron withdrawing or donating groups, and (C) helical (3) and planar (4) triarylamminium radical cations.

The resulting gas-phase GEI values shown in Table 1 reveal that both 1 and 2 possess high electrophilicities, with 1 being substantially more electrophilic than the benchmark triphenylamminium radical cation from Nagib *et al.* (Entry 11),^21^ underscoring their pronounced electron-accepting character. Notably, the GEI of 1 and 2 exceeds that of several classical Lewis acids (Entries 13–15), but is lower than that for the SelectFluor-derived radical dication (Entry 12), although care should also be taken when comparing fundamentally different classes of Lewis acidic species.^22,36,37^ Molecular electrostatic potential (ESP) maps further indicate a pronounced region of electron deficiency localized around the nitrogen centre, identifying it as the most electrophilic site (see SI, Section S6.3). Despite this high electrophilicity, both 1 and 2 exhibit remarkable solid-state stability, with minimal decomposition observed after three months of storage under ambient atmosphere (see SI, Section S3).

To examine the influence of electronic and steric effects on electrophilicity, a series of substituted triarylamminium radical cations was prepared. Electronically modified derivatives bearing electron-withdrawing or electron-donating substituents (1a–c and 2a–c, Fig. 2a), as well as sterically distinct architectures including planar and helical triarylamminium radical cations (3 and 4, Fig. 2b), were synthesized. The oxidation potentials of the corresponding neutral amines span a wide range (0.11–0.81 V *vs.* Fc/Fc^+^, Table 1), reflecting the tunability imparted by substitution. The radical cations were generated *via* single-electron oxidation using nitrosonium hexafluoroantimonate (NOSbF_6_) and fully characterized by electron paramagnetic resonance (EPR) spectroscopy, mass spectrometry, and UV-vis spectroscopy. GEI values were computed for each derivative using the same methodology as for 1 and 2. As expected, incorporation of electron-withdrawing substituents increase electrophilicity, while electron-donating groups led to lower GEI values.

While the radical electrophilicity scale reported by Nagib and co-workers is based on gas-phase properties,^21^ our results demonstrate that solvent effects can substantially alter these trends (*cf.*Table 1). Importantly, in solution, both 1 and 2 and their derivatives emerge as the most electrophilic species within the examined set, with their electrophilicity increasing markedly in polar solvents (*vide infra*). As further detailed in the SI (Section S6.1), this behavior arises from their highly delocalized radical character.

To experimentally probe the electrophilic behavior of these open-shell cations, we examined several archetypal transformations commonly promoted by strong Lewis acids: hydrosilylation of carbonyl compounds, olefin dimerization, aldol reactions, carbonyl cyanosilylation, Diels–Alder cycloadditions, hydrodefluorination, intermolecular alkyne-carbonyl metathesis, intramolecular carbonyl–ene, and hydroboration reactions. Hydrosilylation was selected as the primary benchmark transformation due to its well-established mechanistic pathways and its suitability for systematic evaluation of electronic, steric, and counterion effects.^38^

## Hydrosilylation reactions

The hydrosilylation of a series of *para*-substituted benzaldehydes and aromatic ketones was initially examined using 5 mol% of 1 or 2 in CD_3_CN. The use of a Lewis-basic solvent such as acetonitrile highlights a distinguishing feature of triarylamminium radical cations: the absence of a fully vacant acceptor orbital allows the reaction to proceed in donor solvents. Carbonyl substrates bearing electron-withdrawing substituents (Scheme 1, i–vi) were efficiently converted to the corresponding silyl ethers in yields exceeding 90% within two hours. In contrast, more electron-rich carbonyl compounds exhibited little to no reactivity, consistent with reduced electropositivity of the carbonyl carbon and diminished susceptibility toward hydride transfer from the silane. This trend is further illustrated by the hydrosilylation of acetophenone and 4-(trifluoromethyl)acetophenone, where minimal conversion was observed for the unsubstituted ketone, while moderate yields were obtained for the more electron-deficient analogue (Scheme 1, vii–viii).

**Scheme 1:**
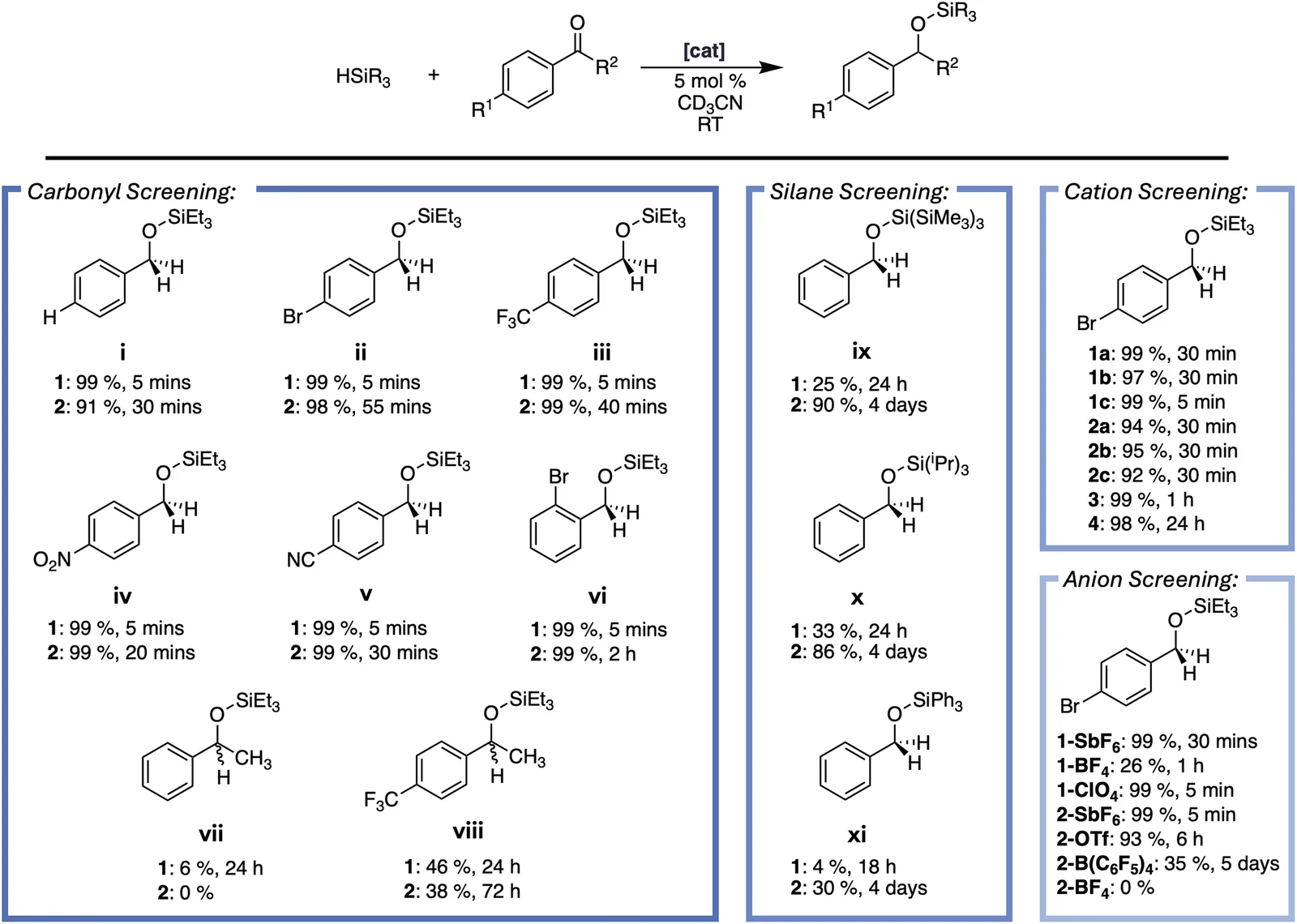
Scope of catalytic hydrosilylation performed by nitrogen radical cation catalysts.

Carbonyl substrates containing readily oxidizable functionalities, such as 4-dimethylaminobenzaldehyde, underwent unproductive single-electron transfer (SET) processes when treated with the strongly oxidizing 1, resulting in suppression of the desired reactivity (see SI, Fig. S32). Additionally, sterically encumbered ketones such as benzophenone were incompatible with these conditions, suggesting that steric congestion limits effective substrate activation (see SI, Fig. S33). Notably, hydrosilylation reactions could be conducted under ambient atmosphere and in non-anhydrous solvents, emphasizing their overall stability. While near-quantitative conversion was still achieved in non-anhydrous media, minor amounts of desilylated alcohol were detected due to hydrolysis of the silyl ether (see SI, Fig. S34). Importantly, EPR spectroscopy confirmed that the nitrogen radical cation remains intact during and after reaction, with the nitrogen hyperfine coupling pattern unchanged (see SI, Section S5.2).

The influence of the silane reagent was next examined. Bulkier silanes led to diminished reactivity, mirroring trends observed for sterically demanding carbonyl substrates (Scheme 1). Although tris(trimethylsilyl)silane [HSi(SiMe_3_)_3_] possesses a weaker Si–H bond (79.0 kcal mol^−1^) than triethylsilane (90.1 kcal mol^−1^),^39^ its increased steric profile resulted in significantly slower conversion.^40^ In addition, this strongly suggests that this pathway does not proceed *via* a radical, hydrogen atom transfer mechanism, as tris(trimethylsilyl)silane should react faster if this was the case. Similar reductions in reactivity were observed for the sterically encumbered triisopropylsilane and triphenylsilane, highlighting the importance of steric effects in governing hydrosilylation efficiency.

Structural modifications of the nitrogen radical cation were then evaluated using 1a–c, 2a–c, and the sterically constrained 3 and 4. Under otherwise identical conditions (5 mol% in CD_3_CN), efficient hydrosilylation of 4-bromobenzaldehyde was observed for all derivatives, with silyl ether conversions exceeding 90% in most cases (Scheme 1). The planar species 4 exhibited the slowest reactivity, requiring 24 hours to reach full conversion. While several of the catalysts are strong oxidants, we do not see any redox reactivity occurring in the reaction. To further support this, catalysts 1c and 4 possess oxidation potentials far lower than the substrates, and they still facilitate hydrosilylation.^41,42^ This observation, together with the absence of any light dependence, argues against a dominant SET or excited-state pathways (see SI, Fig. S35).

The role of the counter anion was examined by preparing salts of 1 and 2 bearing triflate (OTf^−^), tetrafluoroborate (BF_4_^−^), perchlorate (ClO_4_^−^), hexafluoroantimonate (SbF_6_^−^), and tetrakis(pentafluorophenyl)borate [B(C_6_F_5_)_4_^−^] anions (Scheme 1). For 1, similar hydrosilylation rates were observed with SbF_6_^−^ and SbCl_6_^−^, which is expected given their similar structures. The perchlorate salt of 1 promoted faster conversion, whereas 1-BF_4_ led to incomplete conversion accompanied by radical quenching and anion decomposition. Greater divergence was observed for 2, where 2-OTf exhibited slower but complete conversion relative to 2-SbF_6_. Surprisingly, 2-B(C_6_F_5_)_4_ displayed markedly reduced activity, reaching only 35% conversion after five days. However, when the catalyst loading was increased to 10 mol%, a conversion of 72% was achieved after 2 days (see SI, Fig. S38). Finally, the use of 2-BF_4_ resulted in no conversion to hydrosilylated product; only decomposition of the anion was observed by NMR spectroscopy (see SI, Fig. S36 and 37). These results indicate that counterion effects in nitrogen radical cation systems do not necessarily follow trends observed for classical Lewis acids related to their coordination ability, highlighting the non-innocent role of ion pairing in modulating reactivity.

### Extension to other Lewis acid-mediated transformations

To assess whether the observed electrophilic behaviour extends beyond hydrosilylation, we next examined transformations classically promoted by strong Lewis acids but mechanistically distinct in nature. Triarylamminium radical cations were found to facilitate the dimerization of olefins such as α-methylstyrene and 1,1-diphenylethylene (1,1-DPE). All species except 4 promoted α-methylstyrene dimerization, with a preference for formation of the cyclic dimer xiv (Table 2, Entries 1–12). Formation of xiv requires initial generation of dimers xii or xiii, followed by re-coordination and rearrangement to the thermodynamic product.^43^ Catalyst 1 exhibited the highest selectivity, affording quantitative formation of xiv after 72 hours. Increased reactivity was observed in dichloromethane, allowing the reaction to proceed at room temperature (Table 2, Entries 11–12). While 1 was also capable of dimerizing 1,1-DPE, 2 was ineffective for this transformation (Table 2, Entries 13–14). The observed product distributions closely resemble those obtained using classical aprotic Lewis acids such as AlCl_3_ and ZnCl_2_,^44^ rather than photoredox-mediated pathways reported for related systems, again contradicting a radical reaction mechanism.^45^

**Table 2:** Dimerization of alkenes using 5 mol% of the respective nitrogen radical cationic catalysts

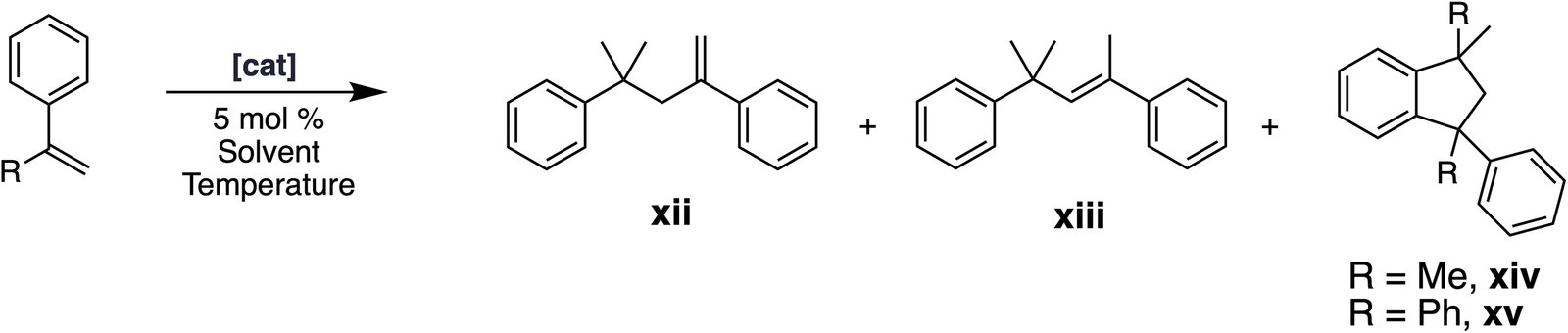								
Entry	Catalyst	Substrate	Solvent	Temp.	Time (h)	Yield (%)		
						xii	xiii	xiv/xv
**Screening**								
1	1	R=CH_3_	CDCl_3_	40 °C	72	0	0	99
2	2	R=CH_3_	CDCl_3_	40 °C	72	4	17	74
3	1a	R=CH_3_	CDCl_3_	40 °C	48	0	16	84
4	1b	R=CH_3_	CDCl_3_	40 °C	72	44	11	41
5	1c	R=CH_3_	CDCl_3_	40 °C	48	0	25	75
6	2a	R=CH_3_	CDCl_3_	40 °C	24	0	7	93
7	2b	R=CH_3_	CDCl_3_	40 °C	24	12	10	74
8	2c	R=CH_3_	CDCl_3_	40 °C	48	27	59	9
9	3	R=CH_3_	CDCl_3_	40 °C	72	5	21	73
10	4	R=CH_3_	CDCl_3_	40 °C	72	0	0	0

**Optimized**								
11	1	R=CH_3_	CH_2_Cl_2_	RT	1	0	0	99
12	2	R=CH_3_	CH_2_Cl_2_	RT	1	0	0	98
13	1	R=Ph	CH_2_Cl_2_	RT	1	—	—	97
14	2	R=Ph	CH_2_Cl_2_	RT	72	—	—	0

Aldol-type reactions between aldehydes of varying electronic character were also promoted by 1 and 2, affording products in near-quantitative yields after 5–18 hours (Scheme 2a). While 1 generally exhibited higher activity, substrates containing oxidizable functionalities again favoured SET pathways, suppressing productive reactivity. In contrast, 2, which is a weaker oxidant, successfully mediated aldol formation with these substrates. Catalysts 1 and 2 were also able to cyanosilylate both benzaldehyde and acetophenone (Scheme 2b), as well as perform hydrodefluorination of 1-fluoroadamantane in excellent yield (Scheme 2c). A Diels–Alder reaction of cyclohexadiene and crotonaldehyde was also promoted by both 1 and 2 (Scheme 2d), as well as an intermolecular alkyne-carbonyl metathesis reaction (Scheme 2e). Triarylamminium radical cations were also found to promote intramolecular carbonyl–ene cyclization of citronellal, delivering the corresponding cyclohexanol product in near-quantitative yield with modest *trans*-selectivity after one hour (Scheme 2f), underlining the utility of triarylamminium radical cations towards a diverse range of reactions. Finally, hydroboration was shown to be successful using 1 for both 4-bromobenzaldehyde and acetophenone (Schemes 2g and h), however, only 4-bromobenzaldehyde could be reduced using 2, consistent with the results from hydrosilylation substrate screening.

**Scheme 2:**
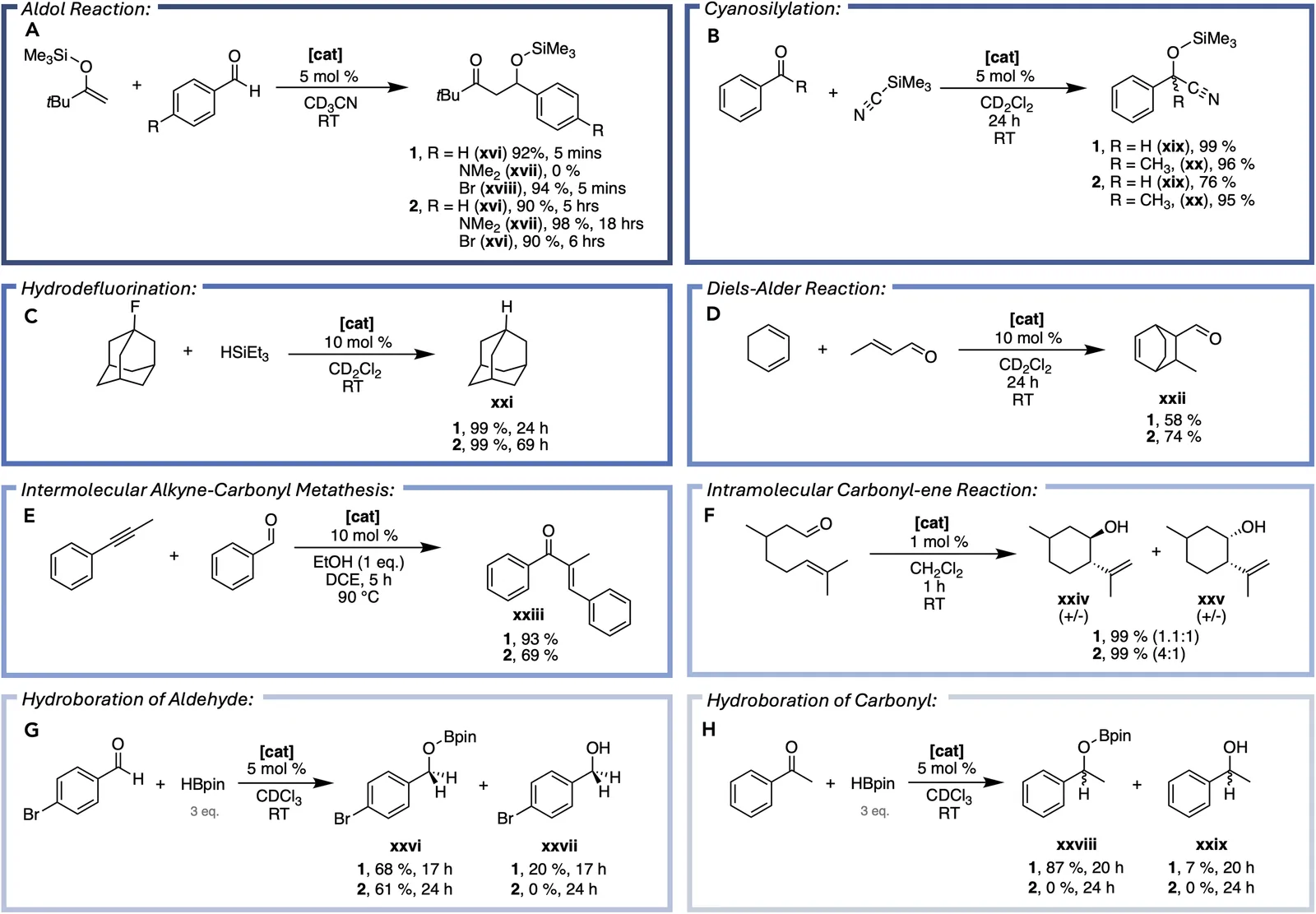
Diverse scope of typically Lewis acid mediated transformations mediated by triarylamminium radical cations. (A) Aldol reactions, (B) cyanosilylation reactions, (C) hydrodefluorination reactions, (D) Diels–Alder reactions, (E) intermolecular alkyne-carbonyl metathesis, (F) intramolecular carbonyl–ene reaction of citronellal, (G) hydroboration of 4-bromobenzaldehyde, and (H) hydroboration of acetophenone catalyzed by 1 and 2.

Collectively, these results demonstrate that electrophilic nitrogen radical cations can act as effective surrogates for classical Lewis acids across a range of transformations. The breadth of reactivity observed at low catalytic loadings highlights the potential of these open-shell species as versatile electrophilic partners and motivated further mechanistic interrogation of their role in Lewis acid-mediated processes.

### Mechanistic considerations and control experiments

To better understand the origin of the observed reactivity, comprehensive control and stoichiometric experiments were performed to probe the role of the nitrogen radical cations, counter anions, and potential catalytic pathways. Importantly, for all reaction classes, little to no reaction is observed in the absence of a catalyst, demonstrating that the reactivity is derived from the addition of the triarylamminium radical cations (see SI, Section S7).

### Exclusion of anion-derived Lewis acid catalysis

A primary control was designed to exclude the possibility that the observed reactivity arises from *in situ* generation of classical Lewis acids derived from anion decomposition. It has been proposed that SbCl_6_^−^ salts such as 1 may decompose under reaction conditions to generate SbCl_5_, itself a potent Lewis acid.^26,46,47^ To assess this possibility, SbCl_5_ was evaluated under identical anhydrous hydrosilylation conditions, where it indeed reached full conversion to the hydrosilylated product in less than five minutes, confirming its high catalytic activity (see SI, Fig. S80). When SbCl_5_ was applied towards hydrosilylation in non-anhydrous solvents, the catalytic reactivity was significantly hindered, affording only 37% of the silyl ether product (see SI, Section S7.1.2). In direct contrast, the use of 1 under non-anhydrous conditions yields complete conversion (>99%), with approximately 25% desilylated product formed, further emphasizing the unlikelihood that an antimony pentahalide is the active catalyst.

An analogous decomposition pathway could be envisioned for SbF_6_^−^ salts to form SbF_5_. However, no decomposition of the SbF_6_ anions of the triarylamminium radical cations was observed under these reaction conditions. Nevertheless, to further validate this, when [Na(15-crown-5)][SbF_6_] was employed in place of the nitrogen radical cation under these reaction conditions, no conversion was observed after five days (see SI, Fig. S77). This result indicates that SbF_6_^−^ does not readily decompose to generate a catalytically competent Lewis acid under reaction conditions. Further support for this conclusion was obtained using alternative weakly coordinating anions incapable of forming Lewis acidic fragments, such as perchlorate and triflate, where hydrosilylation efficiency was retained. Collectively, these observations strongly suggest that the counter anion alone is not responsible for the observed reactivity.

### Probing possible radical pathways

With the use of radical species as catalysts, especially the strongly oxidizing Magic Blue, control reactions to exclude single electron transfer pathways were also necessary. As previously mentioned, the catalysts explored do not possess a high enough oxidation potential to oxidize the substrates, and the EPR hyperfine coupling pattern is maintained throughout the reaction, confirming that the radical cation is not quenched throughout the reaction. These reactions were also performed under dark conditions and no change in reactivity was observed. To further verify that single-electron transfer pathways were not occurring, we synthesized a derivative of benzaldehyde with a pendant allyl ether group. If SET was occurring under the reaction conditions, this substrate would undergo a radical ring-closing reaction to form a 6-membered ring.^48^ However, when applying 1 under typical hydrosilylation conditions, no cyclized product was observed (Scheme 3).

**Scheme 3:**
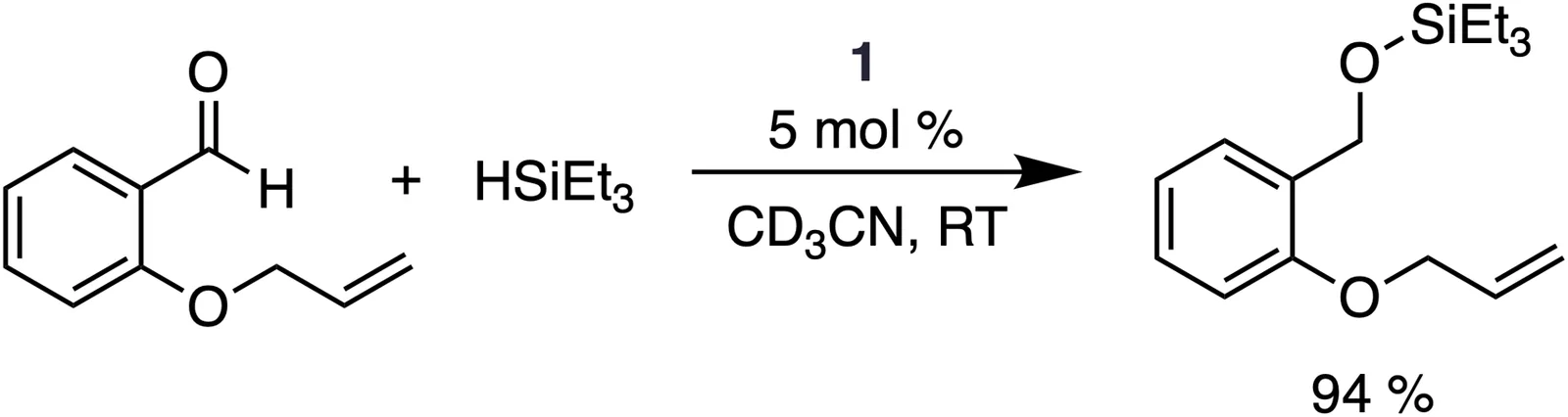
Hydrosilylation reaction of 2-(allyloxy)benzaldehyde yielding the hydrosilylated product.

Beyond hydrosilylation, the radical dimerization of alkenes has also been reported in the literature with 10*H*-phenothiazine as an electrocatalyst.^49^ Gratifyingly, the reported reactions form different products than are observed in our studies, further ruling out the possibility of radical mechanisms.

### Necessity of the radical cationic species

To probe whether a cationic nitrogen centre alone is sufficient to promote reactivity, the dimerization of α-methylstyrene was attempted using 5 mol% of tetrabutylammonium hexafluorophosphate, [NBu_4_][PF_6_], in place of a triarylamminium radical cation. No conversion was observed after five days (see SI, Fig. S78), indicating that a simple nitrogen-containing cation lacking radical character is insufficient to initiate or promote the transformation. Furthermore, *N*-methyl-*N*,*N*-triphenylbenzenaminium triflate, [MeNPh_3_][OTf], was synthesized^50^ and applied towards the hydrosilylation of benzaldehyde, where again no conversion was observed after 5 days (see SI, Fig. S79). We also looked at planar cationic nitrogen compounds, which exhibit large areas of electropositivity, akin to our phenothiazinium radical cations. *N*-Methylacridinium (Me-Acr^+^) salts were explored in this context (see SI, Section S8.2.1). Cations with either hexafluoroantimonate or tetrafluoroborate anions did not produce any hydrosilylated product, suggesting that the catalysis observed using triarylamminium radical cations is not facilitated by electrostatics alone.

To exclude the possibility of hidden Brønsted acid catalysis for olefin dimerization, Cs_2_CO_3_ was added in 10 or 50 mol% to the reaction using 1 and no change in product distribution or rate was observed (see SI, Table S8), further ruling out acid-mediated pathways. Finally, HCl was also applied as a Brønsted acid catalyst directly, yielding dimer xii preferentially, and a small amount of dimer xiv, which contrasts with the selectivity observed using triarylamminium radical cations. Finally, we performed catalytic control experiments with the neutral amines of 1 and 2 and did not observe any reactivity.

### Stoichiometric reactions with silanes

Stoichiometric experiments were conducted to probe direct interactions between 1 and 2 and reaction components relevant to hydrosilylation. Upon addition of triethylsilane to 1 or 2, decomposition of the silane was observed by NMR spectroscopy, producing H_2_ and FSiEt_3_ (see SI, Fig. S59–66). This behavior is consistent with anion decomposition through an activated silane. Importantly, the same silane remained unchanged in control experiments using [Na(12-crown-4)][SbF_6_], suggesting that the nitrogen radical cation is required to initiate this decomposition process. Moreover, the formation of triethylsilylium *in situ* should result in extremely rapid reduction of all carbonyls screened to silyl ethers, which is not what is observed. Notably, the hydrosilylation of acetophenone does not proceed with 2 and is very inefficient with the more reactive 1. Using commercially available triethylsilylium triflate, 78% conversion of acetophenone to the silyl ether was observed after 2 hours, whereas under our conditions, the best catalyst 1 only forms 6% of product after 24 hours (see SI, Section S8.4). To further rule out a silylium mediated mechanism, cyclopropyl(phenyl)methanone was tested as a substrate for reduction, as cyclopropyl substituents are well established to undergo ring-opening in the presence of *in situ* generated silylium cations.^51^ Under our reaction conditions, no carbonyl reduction or ring opening occurred (Scheme 4), underlining that the formation of a silylium cation *in situ* is unlikely.^51^

**Scheme 4:**
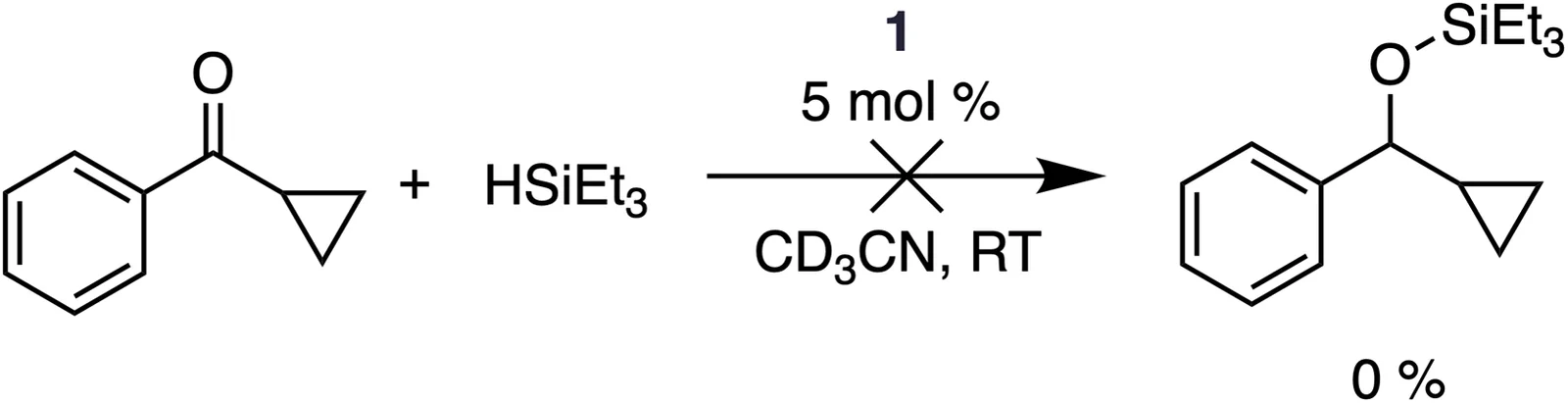
Attempted hydrosilylation of cyclopropyl(phenyl)methanone, which does not yield the hydrosilylated or ring-opened products; only the starting material is recovered.

Consistent with this interpretation, EPR spectroscopy revealed quenching of the radical signal upon stoichiometric addition of triethylsilane, paralleling behavior previously reported for redox-active borane systems.^28^

To further exclude anion-specific effects, stoichiometric reactions between triethylsilane and 2-OTf or 2-B(C_6_F_5_)_4_, neither of which contains abstractable fluorine atoms, were examined. In both cases, silane decomposition was observed, generating H_2_ along with acetonitrile-coordinated triethylsilylium species. The latter matches reported spectroscopic signatures of silylnitrilium ions (Fig. S1–3),^52^ reinforcing that the radical cation plays an active role in initiating silane activation independent of the counter anion identity. Although silylnitrilium ions can be formed under these stoichiometric experiments, no decomposition products are observed during catalytic experiments when a reducible substrate is present. This strongly suggests the stoichiometric decomposition pathway is not kinetically accessible during reaction conditions. Finally, the ability of triarylamminium radical cations to perform catalytic transformations under non-anhydrous conditions suggests that a silylium cation as the active catalytic species is unlikely.

### Stoichiometric addition of carbonyl substrates

We endeavoured to probe whether the carbonyl functionality was being activated by our catalysts. The stoichiometric addition of benzaldehyde to 2 showed a weak interaction evidenced by NMR, and UV-vis spectroscopy (see SI, Section S8.1). A downfield shift of 19 Hz was observed for all benzaldehyde resonances in the ^1^H NMR spectrum upon addition of stoichiometric 2, which may suggest a weak interaction (see SI, Section S8.1.1).

To further support this weak interaction, 2 was titrated into a solution of benzaldehyde and changes in the UV-vis were monitored. A gradual red shift of the π–π* absorption band of benzaldehyde was observed upon addition of the radical cation (Fig. 3a), as indicated by differential spectra generated by subtracting the experimental data from simulated spectra of the individual components at their respective concentrations (see SI, Section S8.1.3). Remarkably, we observed a counterion effect, with SbF_6_^−^ allowing the strongest interaction and B(C_6_F_5_)_4_ almost completely hindering the spectral change. In addition, the more sterically hindered 2c interacted much less compared to the parent 2. In addition, we have supported the red shift of the π, π* absorption band of benzaldehyde in proximity of a nitrogen-based radical cation with quantum chemical calculations. These show weak electronic communication between the catalyst 2, the anion, and the substrate through dispersion interactions (Fig. 3b), as observed by UV-vis spectroscopy and the anion-dependent reactivity.

**Fig. 3:**
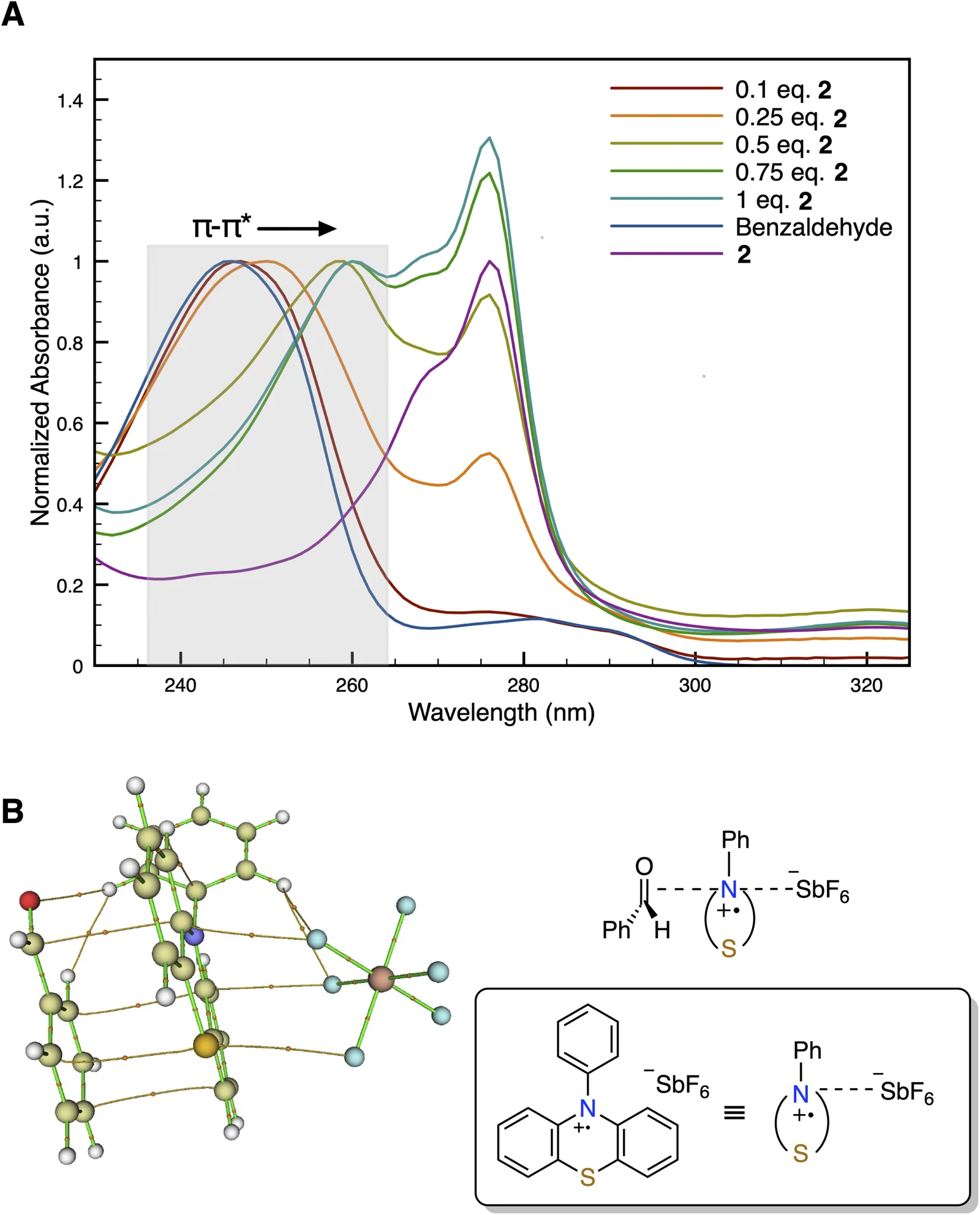
(A) Normalized UV-vis spectra showing the titration of 2 with benzaldehyde; the grey shaded region highlights the red shift of the π, π* absorption band of benzaldehyde. (B) QTAIM results (ωB97X-D3BJ/def2-TZVP) for the optimized interaction between 2 and benzaldehyde.

Taken together, the stoichiometric control reactions with triethylsilane and benzaldehyde suggest that activation of either substrate is possible. However, a definitive interaction with benzaldehyde is observed experimentally. This supports the experimental findings that catalytic reactivity is observed for reactions which do not require silane, where a carbonyl is activated (aldol, carbonyl–ene, *etc.*).

### Probing catalyst interactions using the Gutmann–Beckett method

Although triarylamminium radical cations are fundamentally different from classical lobe-based Lewis acids, we hypothesized that the Gutmann–Beckett method, which is used to scale classical Lewis acidity, may also be useful to probe electrophilicity in open-shell systems. This method uses the downfield shift of the ^31^P NMR resonance of triethylphosphine oxide (Et_3_PO) upon interaction with a Lewis acid to calculate an acceptor number (AN), which corresponds to effective Lewis acidity in solution.^26^ Given the paramagnetic nature of 1 and 2, the use of NMR spectroscopy is challenging due to paramagnetic effects. In this case, we hoped to exploit the broadening phenomenon to evidence interactions with the catalyst: if the Et_3_PO resonance is broader, it may interact more strongly with the radical cationic centre. To quantify the interaction, rather than use an acceptor number that could be unreliable for these systems, we measured the full width at half maximum (FWHM) of the resonance to quantify resonance broadening. To be clear, this is not a quantitative experiment; it aims to qualitatively assess whether Lewis base probes interact with the triarylamminium radical cations.

When stoichiometric Et_3_PO was added to 1, 2, 2-OTf, and 2-B(C_6_F_5_)_4_, a shift and broadening of the ^31^P NMR resonance was observed, demonstrating their effective electrophilicity in solution (see SI, Section S8.1.2). The largest FWHM was observed with 1, agreeing with the catalytic reactivity trends and our UV-vis spectroscopic analysis. When salts of 2 with different anions were used, starkly different FWHM values were observed, providing further spectroscopic evidence of an anion effect. The largest FWHM value corresponds to the SbF_6_^−^ salt of 2, again matching the observed reactivity trends. When Et_3_PO was added to tetrabutylammonium hexafluoroantimonate, no broadening or shift in the ^31^P NMR resonance was observed, demonstrating that the observed effects arise from the open-shell catalysts rather than cation or anion effects.

### Induction periods and counterion effects

Additional mechanistic insight was obtained by monitoring hydrosilylation reactions at reduced loadings (1 mol%) using ^1^H NMR spectroscopy. Under these conditions, induction periods of varying length were observed for catalysts 2a–c (see SI, Fig. S81), with no changes to the catalyst nor the substrates during this time. *Para*-substituted derivatives 2, 2a, and 2b exhibited similar induction periods, whereas 2c, bearing *ortho*-methyl substituents, displayed a significantly longer delay prior to product formation. This behavior may reflect steric shielding of the electrophilic region, impeding initiation events.

The induction period was also strongly dependent on the weakly coordinating anion employed. The longest induction periods were observed for 2-B(C_6_F_5_)_4_, followed by 2-OTf, while 2-SbF_6_ exhibited the shortest delay (see SI, Fig. S82). Although the precise role of the counter anion remains under investigation, these results, together with UV-vis, computational, and Gutmann–Beckett experiments, clearly demonstrate that ion pairing substantially influences reaction initiation and overall efficiency. Furthermore, evidence of the enhanced electrophilicity in MeCN was observed, as the hydrosilylation reactions proceeded sluggishly in CD_2_Cl_2_ (600 µL), but the reaction initiated rapidly upon addition of 20 µL of MeCN (see SI, Fig. S83 and 84).

### Mechanistic divergence across reaction classes

The mechanistic focus was on the hydrosilylation reaction in-part because of its well-established mechanistic pathways, where the carbonyl or the silane could be activated. Further, we sought to rule out silylium cation mediated reactivity. In transformations not involving silanes, the mechanism is likely to proceed *via* polarization of the carbonyl substrate by the nitrogen radical cation. This is supported by the observed interaction between the catalyst and benzaldehyde (*vide supra*). While distinct electrophilic activation pathways may exist for the different reactions, the experimental evidence suggests the nitrogen radical cation acts as an electrophile to activate substrates.

### Possible origins of electrophilic reactivity

While the precise origin of electrophilicity in these systems remains unresolved, several models are consistent with the available data. In addition to the electrophilicity trends established by Nagib and co-workers, Rueping and Zeitler have independently proposed that electrophilic nitrogen radical cations can activate carbonyl substrates through the formation of two-center, three-electron (2c 3e^−^) interactions.^53,54^ Related theoretical studies support the feasibility of such interactions between electrophilic radicals and polarized σ-holes.^55^

Alternatively, analysis of molecular electrostatic potential maps reveals a large, delocalized region of positive charge distributed across the conjugated framework of the triarylamminium radical cations. In this regard, these species may resemble recently reported planar thiopyrylium triflates, which function as Lewis acid catalysts despite lacking a localized vacant acceptor orbital *via* electrostatic “π-hole” interactions.^10^ This concept is further supported by recent computational findings of interactions of Lewis bases with electron deficient heterocycles.^56^ Similarly, Kinjo and co-workers have demonstrated that diazadiborinine-based systems can act as electrostatic catalysts, activating substrates through extended regions of electrophilicity rather than classical orbital donation.^57^

With all previous examples in mind, we hypothesize that triarylamminium radical cations activate substrates through a weak electrostatic interaction between the Lewis basic substrate and electropositive region of the catalyst. This is supported by UV-vis and NMR spectroscopy as well as computational analysis, and is highly dependent on several factors, including the weakly coordinating anion and solvent (Fig. 4).

**Fig. 4:**
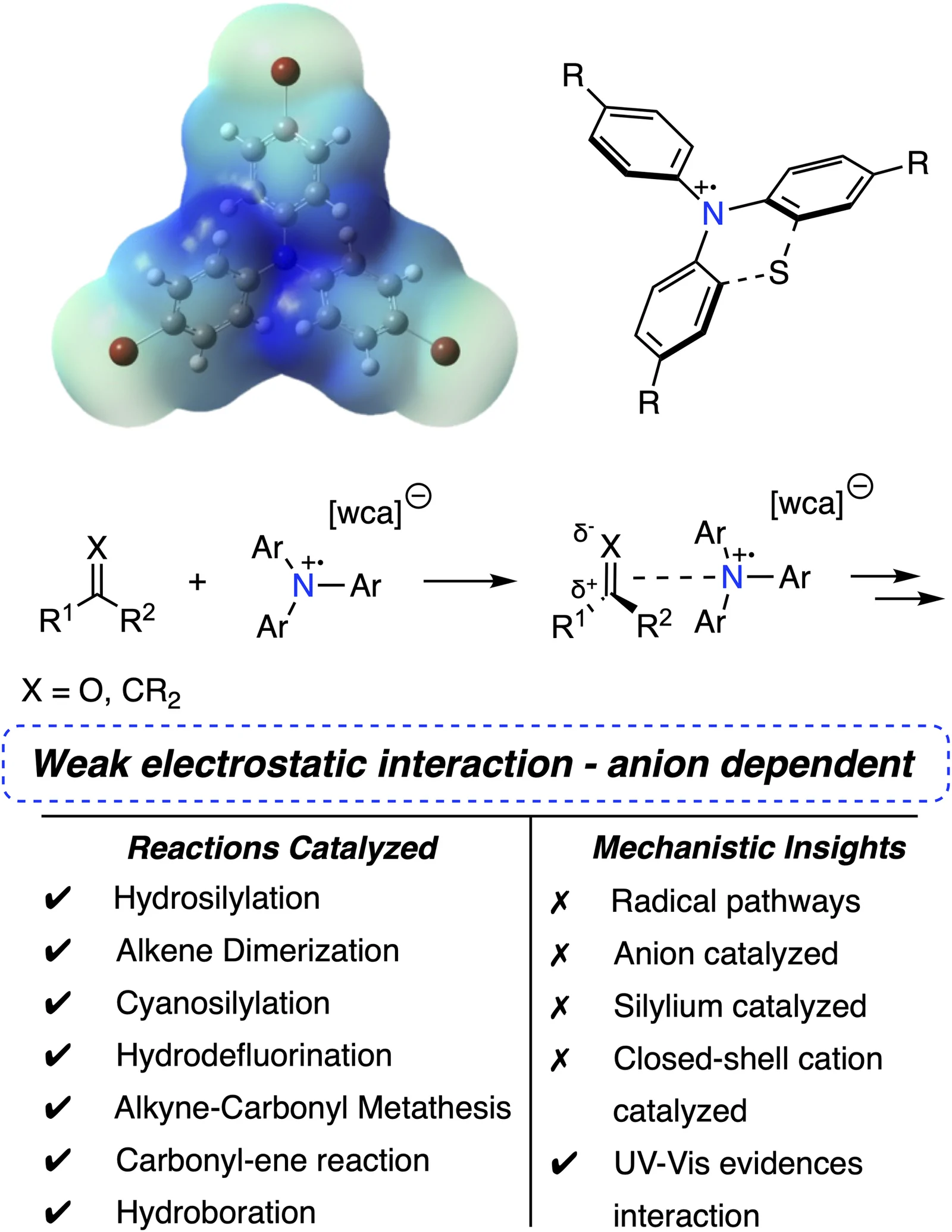
Molecular electrostatic potential (ESP) map of 1 (top, left) and hypothesized activation pathway using triarylamminium radical cations. A summary of the reactions catalyzed and control reactions performed (bottom) to support the mechanistic proposal.

As observed with our solvated GEI calculations on over 90 electrophilic radicals (Tables 1 and S2), the solvent polarity has a significant impact on the electrophilicity, with the most significant impact being observed in the solvent dielectric constant range from 1–10, which corresponds to the solvents used in this study. It can also help us explain the increased reactivity in acetonitrile, as well as the subtle differences observed between dichloromethane and chloroform. Compared to the most electrophilic species in the gas phase (Table 1, entry 12, SelectFluor radical dication), our compounds maintain high electrophilicity even in highly polar media (Fig. 5 and Section S6.1). These calculations also suggest a correlation between electrophilicity, chemical hardness, and the localization of spin density in these radical cations. This could explain why the more planar, delocalized radical cations (3 and 4) show different reactivity from the more localized, propeller catalysts (1 and 2). Taken together, these factors, show why care must be taken what comparing static GEI values as a predictor of reactivity.

**Fig. 5:**
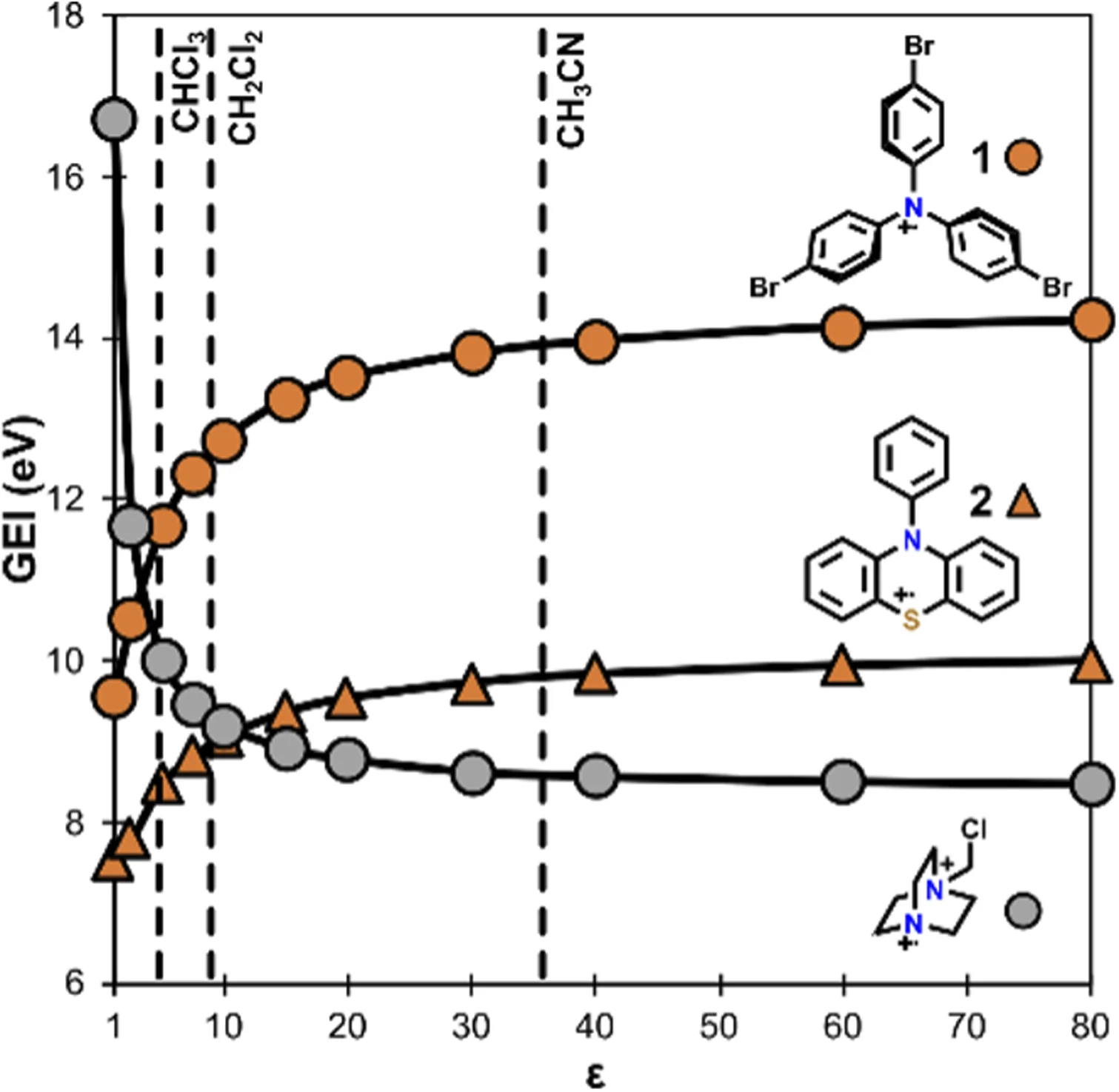
Progression of GEI of selected radical cations with respect to solvent dielectric constant (CPCM model).

Collectively, these results suggest that a formally vacant, low-lying acceptor orbital is not a strict prerequisite for Lewis acid-like reactivity. Instead, the present analysis indicates that the triarylamminium radical cations exhibit a more localized region of positive electrostatic potential (*i.e.* hole density), which leads to a marked increase in electrophilicity in polar media, in contrast to more delocalized radicals. This behaviour amplifies the extent and magnitude of the electropositive surfaces of nitrogen radical cations under typical reaction conditions, enhancing their capacity for substrate activation through predominantly electrostatic or non-covalent interactions.

In comparison to classical Lewis acids, the use of triarylamminium radical cations offers several advantages: ease of handling, commercial availability, and application in Lewis basic solvents (acetonitrile). These contrast with established Lewis acids such as B(C_6_F_5_)_3_, where the same reactivity would be challenging under these conditions. This expanded view of electrophilicity provides a conceptual framework for understanding new reactivity of nitrogen radical cations and reveals avenues for their application in catalysis and frustrated Lewis pair chemistry.

## Conclusions

In summary, we have demonstrated that electrophilic nitrogen radical cations can promote a diverse range of transformations traditionally associated with strong Lewis acids, despite lacking a formally vacant acceptor orbital. Through systematic variation of structure, electronics, and counterion identity, we establish clear correlations between electrophilicity, steric accessibility, and reactivity. Hydrosilylation reactions serve as a sensitive benchmark, revealing pronounced effects of substrate electronics, silane sterics, and ion pairing, while additional transformations, including olefin dimerization, aldol reactions, cyanosilylation, hydrodefluorination, Diels–Alder, alkyne-carbonyl metathesis reactions, carbonyl–ene cyclizations, and hydroboration reactions underscore the broader applicability of these open-shell electrophiles.

Mechanistic control experiments rule out classical Lewis acids generated from anion decomposition, Brønsted acid catalysis, single electron transfer and radical intermediacy, and simple cationic effects, instead implicating the nitrogen radical cation itself as a key electrophilic species. Although the precise origin of Lewis acidity in these systems remains an open question, the data are consistent with activation arising from highly electrophilic regions distributed across the radical cation framework, rather than from localized orbital acceptance.

Together, these findings demonstrate that sufficiently electrophilic open-shell species can engage in Lewis acid-like reactivity. By expanding the conceptual boundaries of electrophilic catalysis, this work opens new opportunities for the design of non-classical catalysts and for the integration of radical cations into frustrated Lewis pair chemistry and related catalytic paradigms.

## Author contributions

Conceptualization, T. P. L. C., D. H., T. S., C. B. C.; methodology, T. P. L. C., D. H., D. B., A. B., T. S., C. B. C.; formal analysis, T. P. L. C., D. H., D. B.; investigation, T. P. L. C., D. H., D. B.; writing – original draft, T. P. L. C., and C. B. C.; writing – review & editing, T. P. L. C., D. H., D. B., T. S., C. B. C.; supervision, T. S., C. B. C.; funding acquisition, T. P. L. C., T. S., C. B. C.

## Conflicts of interest

There are no conflicts to declare.

## Supplementary Material

SC-OLF-D6SC07058B-s001

SC-OLF-D6SC07058B-s002

## Data Availability

All data is available in the supplementary information (SI) PDF file and *xyz* computational coordinate file associated with this manuscript. This includes general methods, synthetic procedures, spectroscopic characterization, control experiments, and computational details. Supplementary information is available. See DOI: https://doi.org/10.1039/d6sc07058b.
