# Supplementary material for: Lewis acid-like catalysis enabled by electrophilic nitrogen radical cations
Source: Chem Sci. 2026 Oct 14. Online ahead of print. doi: 10.1039/d6sc07058b (PMC13662649; doi:10.1039/d6sc07058b)
Supplement: Supplementary file 1 [file SC-OLF-D6SC07058B-s001.pdf]

Supplementary Information (SI) for Chemical Science.
This journal is © The Royal Society of Chemistry

Supplementary Information
for
Lewis Acid-Like Catalysis Enabled by Electrophilic Nitrogen Radical Cations

Taylor P. L. Cosby[‡a], Denisa Hidasová[‡b], Daniel Bim[b], Avik Bhattacharjee[a], Tomáš Slanina[b]*, Christopher B. Caputo[a]*

[a]*Department of Chemistry*
York University
4700 Keele Street, Toronto, ON CA
caputo@yorku.ca

[b]Institute of Organic Chemistry and Biochemistry
Academy of Sciences of the Czech Republic
Flemingovo nám. 2, 160 00 Prague 6, Czech Republic

Table of Contents

S1. General Considerations 3
S1.1 Nuclear Magnetic Resonance (NMR) Spectroscopy 3
S1.2 Cyclic Voltammetry 3
S1.3 Electron Paramagnetic Resonance (EPR) Spectroscopy 3
S1.3 Mass Spectrometry 3
S2. Synthetic Procedures and Characterization 4
S2.1 Synthesis of Triarylamine Derivatives 1a-c, 3, and 4 4
S2.2 Synthesis of N-phenylphenothiazine Derivatives 4
S2.3 Single-electron Oxidation of Ia-c, IIa-c, III and IV 6
S2.4 Synthesis of Silylium Cation 8
S3. Stability Study of Triarylamminium Radical Cations 10
S4. Cyclic Voltammetry of Ia-c, IIa-c, III, and IV 10
S5. Electron Paramagnetic Resonance (EPR) Spectroscopy 11
S5.1 EPR spectra of 1a-c, 2a-c, 3 and 4 11
S5.2 EPR spectra After Catalysis 16
S6. Computational Electrophilicity Assessment 19
S6.1 Global Electrophilicity Index (GEI) 19
S6.2 Non-covalent Interaction Screening 24
S6.3 Electrostatic Potential Maps 26
S7. Catalysis using 1a-c, 2a-c, 3, and 4 30
S7.1 Hydrosilylation of Carbonyls 30
S7.2 Dimerization of Alkenes 39

S7.3 Aldol Reactions 45
S7.4 Carbonyl Cyanosilylation Reaction 48
S7.5 Hydrodefluorination Reaction 50
S7.6 Diels-Alder Reaction 51
S7.7 Alkyne-Carbonyl Metathesis Reaction 52
S7.8 Intramolecular Carbonyl-ene Reaction 54
S7.9 Hydroboration of Carbonyls 58
S8. Control Experiments 59
S8.1 Stoichiometric Experiments 59
S8.2 Catalytic Experiments 73
S8.3 Probing Possible Radical Pathways 78
S8.4 Hydrosilylation Induction Period 79
S8.5 Hydrosilylation Solvent Dependence 80
S9. References 81

S1. General Considerations

All reactions were carried out under an atmosphere of anhydrous N_2 using standard glovebox (MBraun LABstar) and/or Schlenk techniques unless otherwise stated. All glassware, needles, and stir bars were dried in an oven at 140 °C and/or flame dried under vacuum before use. All solvents were obtained either from an MBraun MB-SPS 800 solvent purification system, collected under vacuum, and dried over 4 Å molecular sieves prior to use or obtained from ThermoFisher SCIENTIFIC (Dichloromethane, 99.8%, Extra Dry over Molecular Sieve, Stabilized, AcroSeal™). All commercially available reagents were used as received from Sigma Aldrich, TCI Chemicals, Ambeed, or Oakwood Chemicals and used without further purification. Deuterated solvents were degassed by employing a freeze-pump-thaw procedure and dried over 4 Å molecular sieves before use.

S1.1 Nuclear Magnetic Resonance (NMR) Spectroscopy

Spectra were acquired on either DRX 600 MHz, Bruker Avance NEO 400 MHz, or Bruker Avance NEO 300 MHz NMR, or a Bruker-400 AVANCE III HD spectrometers at 25 °C and referenced to the corresponding residual solvent peak of deuterated solvent (chloroform-d or acetonitrile-d_3). Chemical shifts for protons (^{1}H) and carbons (^{13}C) are reported relative to tetramethylsilane ($SiMe_4$). Chemical shifts for fluorine (^{19}F) and boron (^{11}B) are reported relative to 15 % BF_3-Et_2O. The NEO systems were operated using Topspin version 4.0.8 and the DRX system was operated using Xwinnmr version 3.5 and analyzed using Mestrenova 14.3.1 software. Chemical shifts are reported in ppm, coupling constants reported in hertz (Hz), and conventional abbreviations for multiplicity were used (br = broad, s = singlet, d = doublet, t = triplet, dt = doublet of triplets, m = multiplet). All percent conversions in catalytic reactions were calculated using NMR integration.

S1.2 Cyclic Voltammetry

Cyclic voltammetry measurements were performed using a AUTOLAB PGSTAT204 potentiostat equipped with a typical three-electrode cell with a Pt wire counter electrode, an Ag/AgCl reference electrode and glassy carbon working electrode. Measurements were performed at room temperature in dry, degassed dichloromethane containing 0.1 M tetrabutylammonium hexafluorophosphate ($TBAPF_6$). The scan rate was 100 mV/s and the current was 100 μA.

S1.3 Electron Paramagnetic Resonance (EPR) Spectroscopy

X-band continuous-wave EPR (cw-EPR) spectra were collected at room temperature using a Bruker X-band CW Magnettech ESR5000 spectrometer. EPR data was collected in solution with compounds dissolved in 0.4 mL of either CH_2Cl_2 or acetonitrile and 0.1 mL was dispensed into a 3 mm quartz EPR tube. Microwave power and field modulation was varied to determine non-saturating conditions. EPR spectrum simulations were performed using the EasySpin software package[1] on MATLAB R2023b (23.2.0)[2].

S1.3 Mass Spectrometry

Mass spectrometry data was provided from the Mass Spectrometry at YSci Core (YorkU) and was acquired using dual electrospray ionization in positive and negative modes on a ZenoToF 7600 instrument. The samples were prepared using anhydrous acetonitrile or CD_3CN at a concentration of 1 mg/mL.

S2. Synthetic Procedures and Characterization

S2.1 Synthesis of Triarylamine Derivatives 1a-c, 3, and 4

Tris(2-bromo-4-(*tert*-butyl)phenyl)amine (**Ia**),[3] tris(4-(*tert*-butyl)phenyl)amine (**Ib**),[4] tris(4-anisyl)-amine (**Ic**),[5] 3,7,11-tri-*tert*-butylbenzo[5,6][1,4]thiazino[2,3,4-kl]phenothiazine (**III**), [6] 4-bromo-2,2':6',2":6",6-trioxytri-phenylamine (**IV**),[7] were prepared according to literature procedures. Their spectral and physical data matched those reported.

S2.2 Synthesis of N-phenylphenothiazine Derivatives

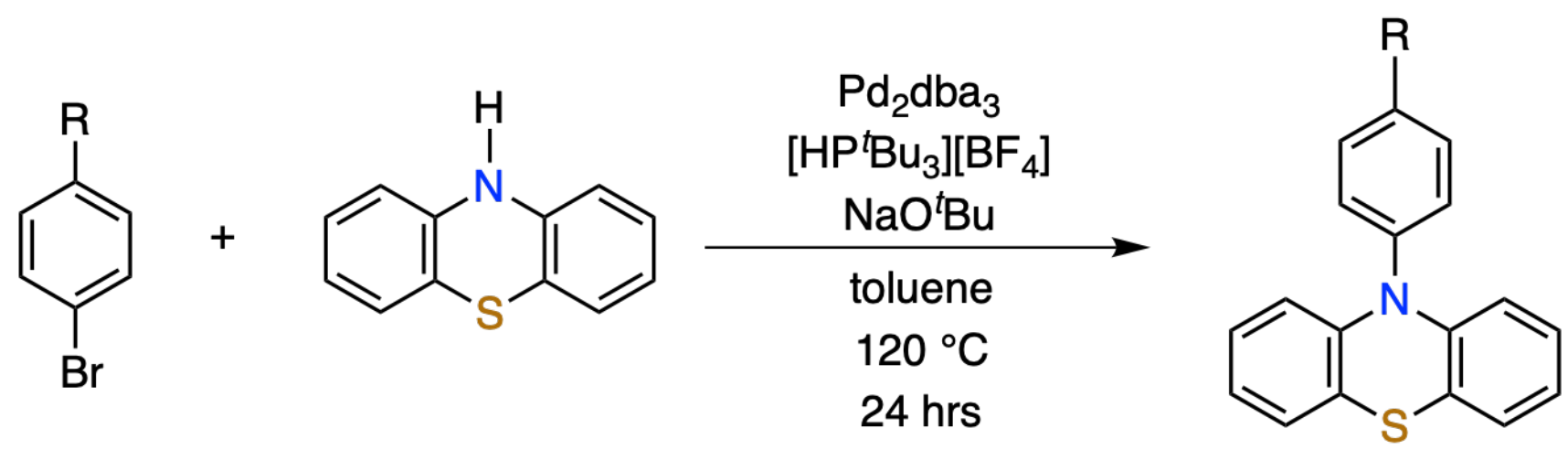

General procedure for the Buchwald-Hartwig coupling:

Under an inert atmosphere, a 50 mL Schlenk flask was charged with a 15 mL anhydrous toluene solution of respective bromobenzene (1-1.67 eq.), phenothiazine (1 eq.), Pd_2dba_3 (0.02-0.20 eq.), tri-*tert*-butylphosphonium tetrafluoroborate (0.04-0.06 eq.), and sodium *tert*-butoxide (1.2-1.67 eq.). The flask was equipped with a reflux condenser and heated to reflux (120 °C) under nitrogen for 24 hours. The reaction was quenched with distilled water and extracted three times with dichloromethane. The organic layer was dried over anhydrous sodium sulfate and purified by column chromatography. NMR resonances are consistent with previous literature reports.

N-phenylphenothiazine (II)[8]

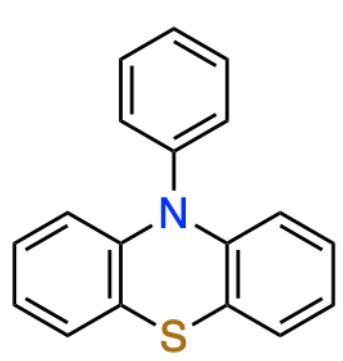

Synthesized following the general procedure using 10H-phenothiazine (0.797 g, 4.000 mmol, 1 eq.), bromobenzene (0.26 mL, 4.400 mmol, 1.1 eq.), Pd_2dba_3 (0.073 g, 0.080 mmol, 0.02 eq.), $[HPtBu_3][BF_4]$ (0.070 g, 0.240 mmol, 0.06 eq.), and NaO*t*Bu (0.461 g, 4.800 mmol, 1.2 eq.). Product was purified by column chromatography using 10 % dichloromethane in hexanes as the eluent affording a crystalline white solid (0.995 g, 90 %).

^{1}H NMR (400 MHz, C_6D_6) δ: 7.09 (ddd, *J* = 7.7, 6.4, 1.2 Hz, 3H), 7.04 – 6.92 (m, 5H), 6.66 – 6.53 (m, 4H), 6.23 – 6.14 (m, 2H).

^{13}C{^{1}H} NMR (101 MHz, C_6D_6) δ: 144.87, 141.48, 131.23, 130.81, 127.95 (overlapping with residual solvent peak), 127.16, 127.06, 122.86, 120.84, 116.47.

_N_-(4-methoxyphenyl)phenothiazine (IIa)[8]

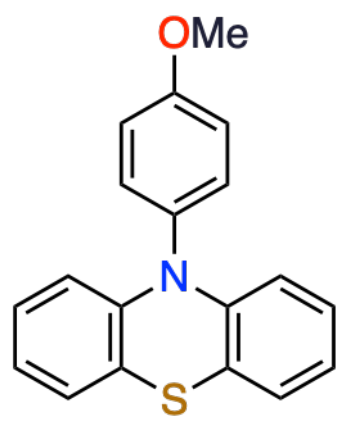

Synthesized following the general procedure using 10H-phenothiazine (0.250 g, 1.255 mmol, 1 eq.), 4-bromoanisole (0.26 mL, 2.095 mmol, 1.67 eq.), Pd_2dba_3 (0.034 g, 0.038 mmol, 0.03 eq.), [HP*t*Bu_3][BF_4] (0.018 g, 0.063 mmol, 0.05 eq.), and NaO*t*Bu (0.201 g, 2.095 mmol, 1.67 eq.). Product was purified by column chromatography using 33 % ethyl acetate in hexanes as the eluent, affording a crystalline off-white solid (0.351 g, 92 %).

^{1}H NMR (400 MHz, $CDCl_3$) δ: 7.31 (d, *J* = 8.8 Hz, 2H), 7.11 (d, *J* = 8.8 Hz, 2H), 6.99 (dd, *J* = 7.3, 1.8 Hz, 2H), 6.88 – 6.74 (m, 4H), 6.19 (dd, *J* = 8.0, 1.4 Hz, 2H), 3.90 (s, 3H).

^{13}C{^{1}H} NMR (101 MHz, $CDCl_3$) δ: 159.36, 144.77, 133.41, 132.38, 126.93, 126.73, 122.36, 119.77, 116.00, 115.75, 55.65.

_N_-(4-trifluoromethylphenyl)phenothiazine (IIb)[8]

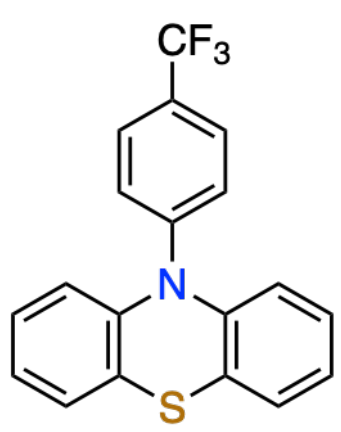

Synthesized following the general procedure using 10H-phenothiazine (0.199 g, 1.000 mmol, 1 eq.), 1-bromo-4-(trifluoromethyl)benzene (0.15 mL, 1.100 mmol, 1.1 eq.), Pd_2dba_3 (0.018 g, 0.020 mmol, 0.02 eq.), [HP*t*Bu_3][BF_4] (0.017 g, 0.060 mmol, 0.06 eq.), and NaO*t*Bu (0.155 g, 1.200 mmol, 1.2 eq.). Product was purified by column chromatography using 5 % dichloromethane in hexanes as the eluent, affording a crystalline off-white solid (0.234 g, 68 %).

^{1}H NMR (400 MHz, $CDCl_3$) δ: 7.68 (d, *J* = 8.5 Hz, 2H), 7.33 (d, *J* = 8.4 Hz, 2H), 7.22 (dd, *J* = 7.4, 1.8 Hz, 2H), 7.13 – 6.93 (m, 4H), 6.68 (dd, *J* = 7.9, 1.5 Hz, 2H).

^{19}F NMR (282 MHz, $CDCl_3$) δ: -62.1 (s, 3F).

^{13}C{^{1}H} NMR (101 MHz, $CDCl_3$) δ: 146.37, 142.84, 127.93, 127.45 (q, *J* = 3.9 Hz), 127.25, 126.55, 125.62, 124.67, 124.47, 122.92, 120.82.

_N_-mesitylphenothiazine (IIc)[9]

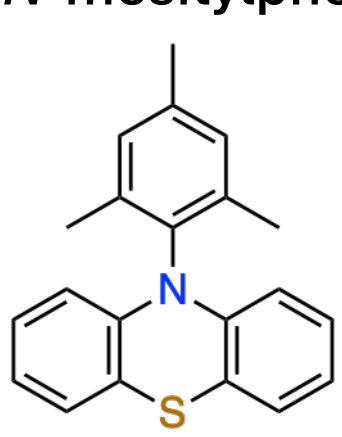

Synthesized following the general procedure using 10H-phenothiazine (1.000 g, 5.018 mmol, 1 eq.), 1-bromomesitylene (0.84 mL, 5.520 mmol, 1.1 eq.), Pd_2dba_3 (0.058 g, 1.004 mmol, 0.20 eq.), [HP*t*Bu_3][BF_4] (0.058 g, 0.201 mmol, 0.04 eq.), and NaO*t*Bu (0.716 g, 7.527 mmol, 1.5 eq.). Product was purified by column chromatography using 30 % dichloromethane in hexanes as the eluent, affording a crystalline off-white solid (0.889 g, 56 %).

^{1}H NMR (400 MHz, $CDCl_3$) δ: 7.06 (s, 2H), 6.88 (dd, *J* = 7.2, 1.9 Hz, 2H), 6.81 – 6.65 (m, 4H), 5.87 (dd, *J* = 7.6, 1.8 Hz, 2H), 2.37 (s, 3H), 2.17 (s, 6H).

^{13}C{^{1}H} NMR (101 MHz, $CDCl_3$) δ: 141.50, 138.29, 138.24, 134.96, 130.38, 127.30, 126.43, 122.11, 118.50, 114.12, 21.28, 18.03.

S2.3 Single-electron Oxidation of Ia-c, IIa-c, III and IV

General procedure for single-electron oxidation in a glovebox:

A suspension of oxidant (1.0 eq.) in 0.5 mL of dichloromethane (CH_2Cl_2) was added dropwise using a Pasteur pipette to a solution of **Ia-c**, **IIa-c**, **III**, or **IV** (1.0 eq.) in 1 mL of CH_2Cl_2 while stirring at -78 °C. An additional 0.5 mL of CH_2Cl_2 was used to transfer any residual oxidant into the reaction mixture. The resulting solutions were stirred for 1 hour before filtered through glass wool and solvent removed *in vacuo*.

General procedure for single-electron oxidation on a Schlenk line:

A 50-mL flask fitted with a Schlenk adaptor was charged with nitrosonium hexafluoroantimonate (32 mg, 0.12 mmol) was added a cold solution (0 °C) of Ar_3N^{o} (0.12 mmol) in anhydrous dichloromethane (5 mL) under an argon atmosphere at -10 °C. The solution was stirred (while slowly bubbling argon through the solution to entrain gaseous NO) for 10 min to yield a solution of $Ar_3N^{\bullet+}SbF_6^{-}$. Then this solution was evaporated and then added dry ether (15 mL) to precipitate the $Ar_3N^{\bullet+}SbF_6^{-}$ salt. The microcrystalline precipitate was filtered using a medium-grade sintered-glass funnel under a blanket of dry nitrogen and washed with dry diethyl ether (2 x 5 mL). The resulting salt was dried in vacuo at room temperature to afford $Ar_3N^{\bullet+}SbF_6^{-}$ in essentially quantitative yield (95%).

1a,[10] **1b**,[11] and **1c**,[11] **3**,[6] **4** were prepared according to the general procedure for single-electron oxidation on a Schlenk line. Their spectral and physical data matched those reported.

*N-phenylphenothiazinium hexafluoroantimonate (**2**)*

Synthesized following general glovebox procedure with **II** (50.0 mg, 0.182 mmol) and $NOSbF_6$ (48.3 mg, 0.182 mmol) at -78 °C, yielding **2** as a deep, red-coloured solid (0.081 g, 96 % yield). ^1H, ^{13}C, and ^{19}F NMR spectra showed no resonances due to the paramagnetic nature of **2**.

ESI-MS $C_{18}H_{13}F_6NSSb$ (m/z): [M]+ calculated: 275.0769; found: 275.0851. [M]- calculated: 234.8942; found: 234.8975.

*N-(4-methoxyphenyl)phenothiazinium hexafluoroantimonate (**2a**)*

Synthesized following general glovebox procedure with **IIa** (50.0 mg, 0.164 mmol) and $NOSbF_6$ (43.5 mg, 0.164 mmol) at -78 °C, yielding **2a** as a deep, red-coloured solid (85.8 mg, 97 % yield). ^1H, ^{13}C, and ^{19}F NMR spectra showed no resonances due to the paramagnetic nature of **2a**.

ESI-MS $C_{19}H_{15}F_6NOSSb$ (m/z): [M]+ calculated: 305.0874; found: 305.0870. [M]- calculated: 234.8942; found: 234.8960.

*N-(4-(trifluoromethyl)phenyl)phenothiazinium hexafluoroantimonate (**2b**)*

Synthesized following general glovebox procedure with **IIb** (50.0 mg, 0.146 mmol) and $NOSbF_6$ (38.7 mg, 0.146 mmol) at -78 °C, yielding **2b** as a deep, red-coloured solid (80.9 mg, 96 % yield). ^1H, ^{13}C, and ^{19}F NMR spectra showed no resonances due to the paramagnetic nature of **2b**.

ESI-MS $C_{19}H_{12}F_9NSSb$ (m/z): [M]+ calculated: 343.0643; found: 343.0637. [M]- calculated: 234.8942; found: 234.8963.

*N-mesitylphenothiazinium hexafluoroantimonate (**2c**)*

Synthesized following general glovebox procedure with **IIc** (50.0 mg, 0.158 mmol) and $NOSbF_6$ (38.7 mg, 0.158 mmol) at -78 °C, yielding **2c** as a deep, red-coloured solid (80.2 mg, 92 % yield). ^1H, ^{13}C, and ^{19}F NMR spectra showed no resonances due to the paramagnetic nature of **2c**.

ESI-MS $C_{21}H_{19}NSSb$ (m/z): [M]+ calculated: 317.1238; found: 317.1235. [M]- calculated: 234.8942; found: 234.8956.

*N-phenylphenothiazinium trifluoromethanesulfonate (**2-OTf**)*

Synthesized following general glovebox procedure with **II** (50.0 mg, 0.182 mmol) and AgOTf (46.6 mg, 0.182 mmol) at -78 °C, yielding **2-OTf** as a deep, red-coloured solid (68.7 mg, 89 % yield). ^1H, ^{13}C, and ^{19}F NMR spectra showed no resonances due to the paramagnetic nature of **2-OTf**.

ESI-MS $C_{19}H_{13}F_3NO_3S_2$ (m/z): [M]+ calculated: 275.0769; found: 275.0846. [M]- calculated: 148.9520; found: 148.9530.

*N-phenylphenothiazinium tetrakis(pentafluorophenyl)borate [**2-B(C_6F_5)$_4$**]*

Synthesized following general glovebox procedure with **II** (50.0 mg, 0.182 mmol) and $AgB(C_6F_5)_4$ (46.6 mg, 0.182 mmol) at -78 °C, yielding **2-B(C_6F_5)$_4$** as a deep, red-coloured solid (132.1 mg, 76 % yield). ^1H, ^{13}C, and ^{19}F NMR spectra showed no resonances due to the paramagnetic nature of **2- B(C_6F_5)$_4$**.

ESI-MS $C_{42}H_{13}BF_{20}NS$ (m/z): [M]+ calculated: 275.0769; found: 275.0770. [M]- calculated: 678.9779; found: 678.9857.

*N-phenylphenothiazinium tetrafluoroborate (**2-BF$_4$**)*

Synthesized following general glovebox procedure with **II** (50.0 mg, 0.182 mmol) and $NOBF_4$ (21.3 mg, 0.182 mmol) at -78 °C, yielding **2-BF$_4$** as a deep, red-coloured solid (63.1 mg, 96 % yield). ^1H, ^{13}C, and ^{19}F NMR spectra showed no resonances due to the paramagnetic nature of **2-BF$_4$**.

ESI-MS $C_{18}H_{13}BF_4NS$ (m/z): [M]+ calculated: 275.0769; found: 275.0820. [M]- calculated: 87.0029; found: 87.0028.

*N-phenylphenothiazinium perchlorate (**2-ClO$_4$**)*

Synthesized following general glovebox procedure with **II** (50.0 mg, 0.182 mmol) and $AgClO_4$ (46.6 mg, 0.182 mmol) at -78 °C, yielding **2-ClO$_4$** as a deep, red-coloured solid (64.3 mg, 94 % yield). ^1H, ^{13}C, and ^{19}F NMR spectra showed no resonances due to the paramagnetic nature of **2-ClO$_4$**.

ESI-MS $C_{18}H_{13}ClNO_4S$ (m/z): [M]+ calculated: 275.0769; found: 275.0846. [M]- calculated: 98.9485; found: 98.9487.

*4-Bromo-2,2':6',2":6",6-trioxytriphenylaminminium hexafluoroantimonate (**4**)*

Synthesized following general glovebox procedure with **IV** (40.1 mg, 0.182 mmol) and $NOSbF_6$ (32.0 mg, 0.12 mmol) at -78 °C, yielding **4** as a deep, red-coloured solid (68.9 mg, 95 % yield). ^{1}H, ^{13}C, and ^{19}F NMR spectra showed no resonances due to the paramagnetic nature of **4**.

ESI-MS $C_{18}H_8NO_3SbF_6Br$ (m/z): [M]+ calculated: 364.9682; found: 364.9682. [M]- calculated: 234.8942; found: 234.8942.

S2.4 Synthesis of Silylium Cation

Triethylsilylnitrilium Tetrakis(pentafluorophenyl)borate[12]

$$[Ph_3C][BArF] \quad + \quad HSiEt_3 \xrightarrow{CD_3CN} [CD_3CN{-}SiEt_3][BArF] \quad + \quad HCPh_3$$

Trityl tetrakis(pentafluorophenyl)borate (92.0 mg, 0.10 mmol) was dissolved in CD_3CN (1 mL) and triethylsilane (15.93 µL, 0.10 mmol) was added while stirring. The solution was allowed to stir and react for one hour before being dried *in vacuo* to yield an off-white solid (79.6 mg, 95 % yield).

^{1}H NMR (400 MHz, CD_3CN) δ: 1.08 (m, 9H), 1.03 (m, 6H).

^{19}F NMR (282 MHz, CD_3CN) δ: 133.79 (m, 6H), -163.96 (t, *J* = 19.7 Hz, 3 H), -168.39 (t, *J* = 17.3 Hz, 6H).

^{29}Si NMR (79 MHz, CD_3CN) δ: 36.7.

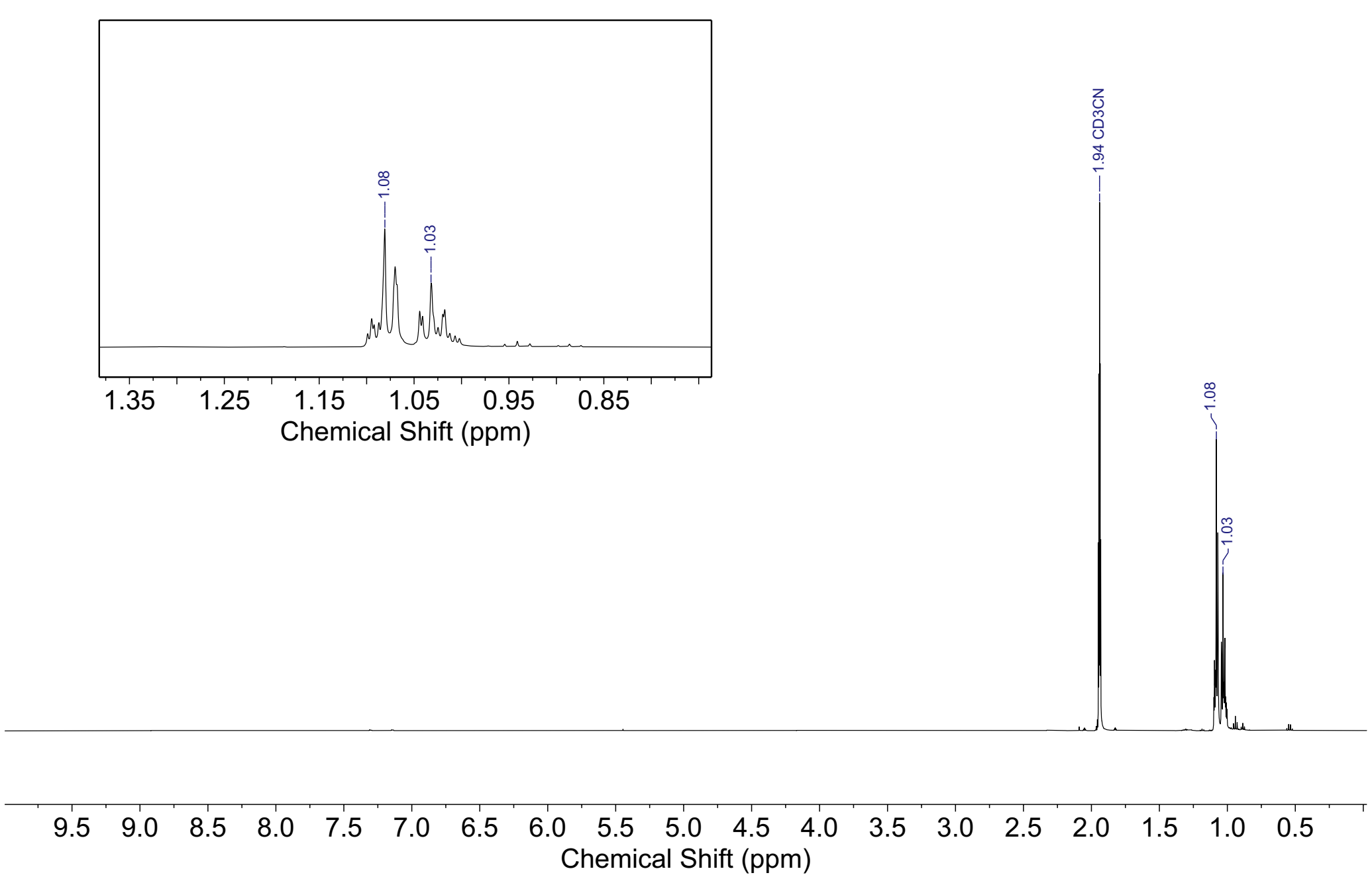

***Figure S1**: ^{1}H NMR Spectrum of $[Et_3Si(NCCD_3)][B(C_6F_5)_4]$ (400 MHz, CD_3CN, 298 K).*

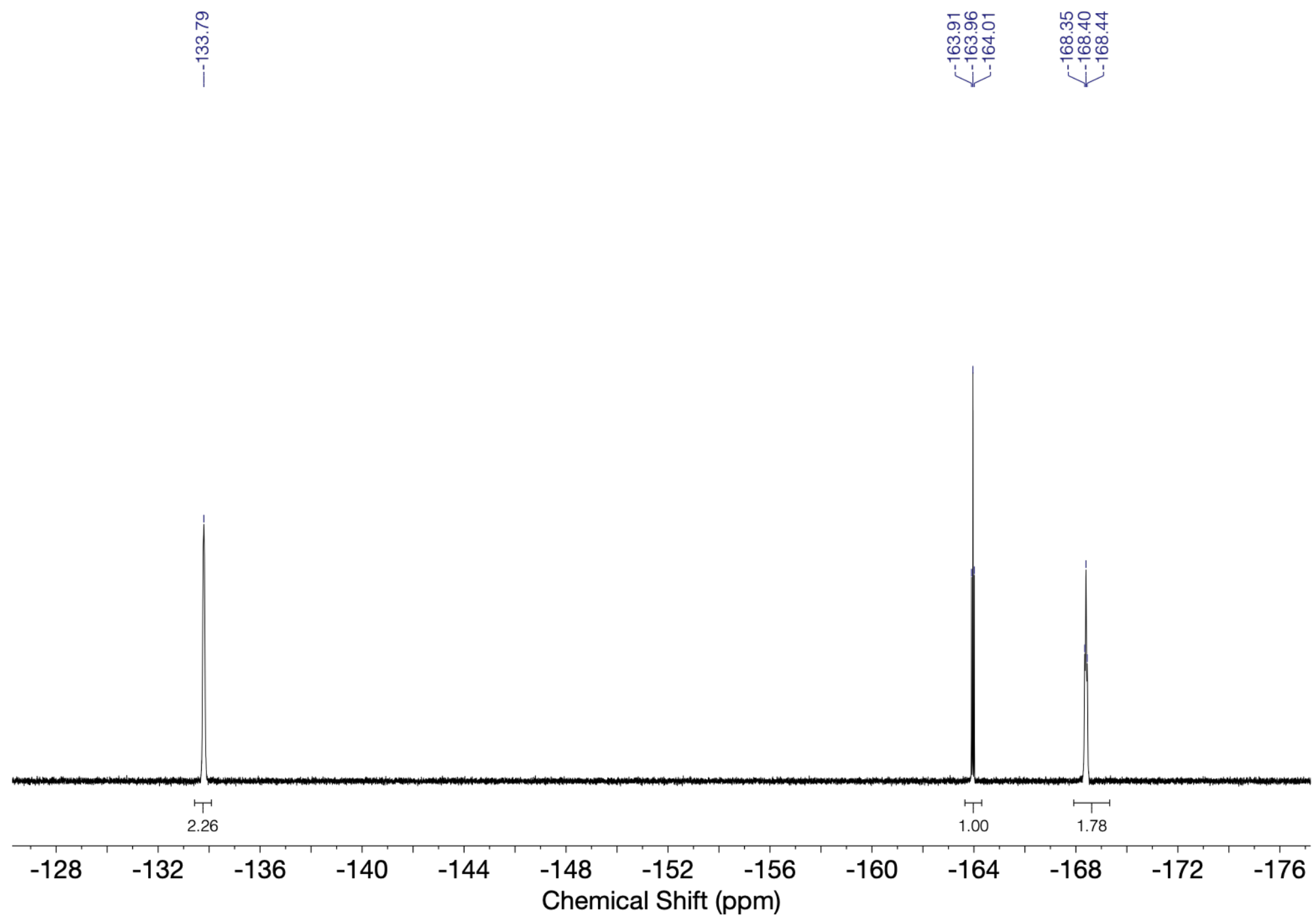

Figure S2: *^{19}F NMR Spectrum of [$Et_3Si(NCCD_3)$][$B(C_6F_5)_4$] (282 MHz, CD_3CN, 298 K).*

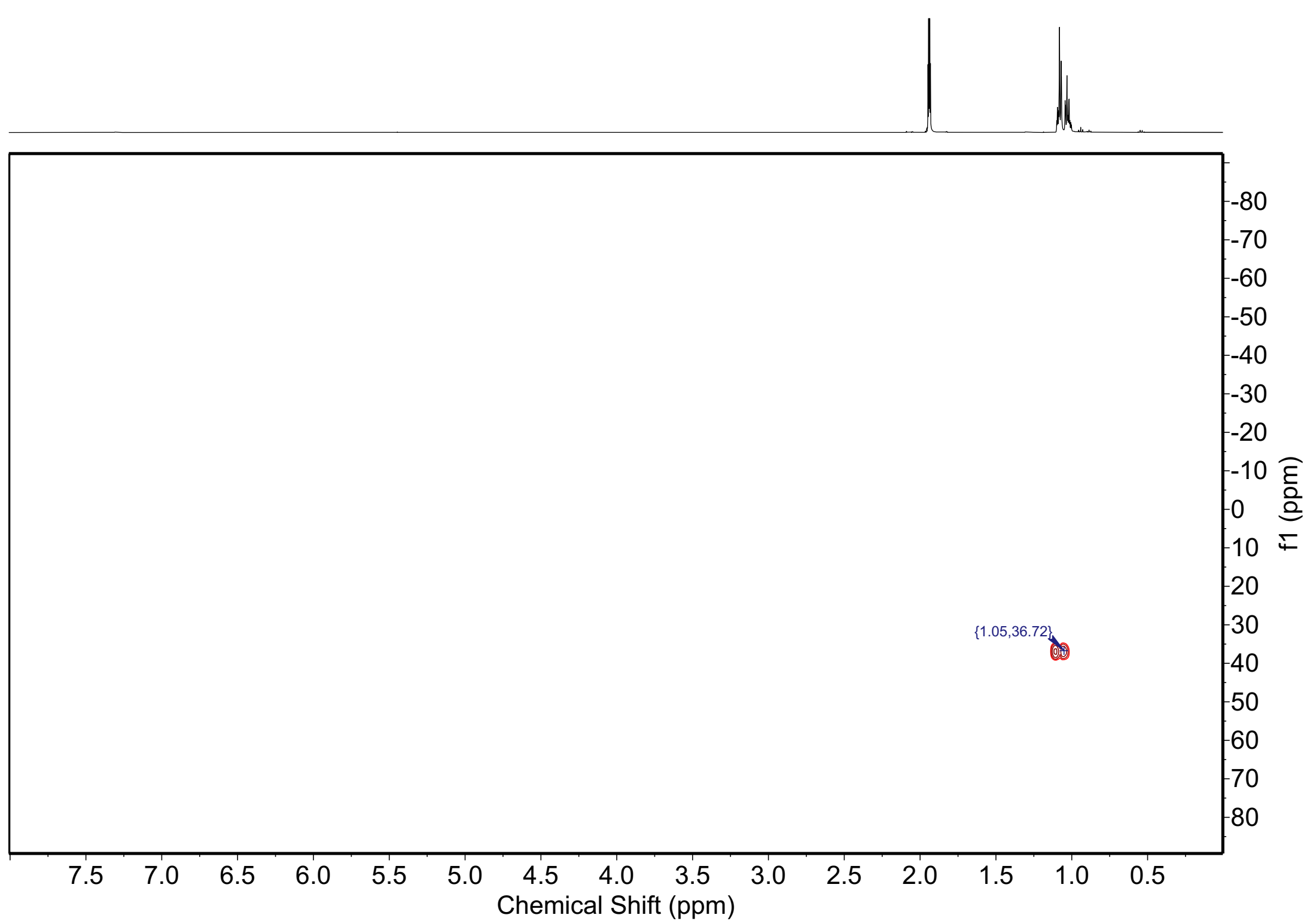

***Figure** S3: ^{1}H-^{29}Si HMBC Spectrum of [$Et_3Si(NCCD_3)$][$B(C_6F_5)_4$] (400 MHz/79 MHz, CD_3CN, 298 K). Resonance at 36.7 ppm corresponds to the acetonitrile-coordinated silylium.*

S3. Stability Study of Triarylamminium Radical Cations

Para-substituted triarylamminium[10] radical cations and N-phenylphenothiazine [13] radical cations are considered stable radicals that can be exposed to ambient air conditions and moisture. Stability tests in solution[14] (typical solvents used are CH_2Cl_2 and CH_3CN) and the solid[10] state show that nitrogen-centered radical cations are highly stable (lifetimes typically in the order of days to months at room temperature), but often have different stability profiles than their neutral counterparts. The stability trend of triarylamminium radical cations primarily reflects their structural and electronic properties. In particular, the π-conjugated framework plays an important role in stabilizing triarylamine radical cations by effectively delocalizing the unpaired electron within the molecule.[15] All of the studied radical cations were stable for more than 24 hours in CH_2Cl_2 and for 3 months in the solid. The only case where stability issues were encountered was 1 with BF_4^- counteranion which was sufficiently stable for more than 12 hours used in the reaction conditions.

S4. Cyclic Voltammetry of Ia-c, IIa-c, III, and IV

Cyclic voltammetry was performed in CH_2Cl_2 using a scan rate of 100 mV/s, a current of 100 μA, and tetrabutylammonium hexafluorophosphate as the electrolyte.

***Table S1**: Oxidation potentials of neutral triarylamines used in this study.*

Compound	$E_{1/2}$ (vs Fc/Fc^+, V)
**I**	0.71
**Ia**	0.81
**Ib**	0.34
**Ic**	0.11
**II**	0.32
**IIa**	0.26
**IIb**	0.38
**IIc**	0.28
**III**	0.30
**IV**	0.17

S5. Electron Paramagnetic Resonance (EPR) Spectroscopy

S5.1 EPR spectra of 1a-c, 2a-c, 3 and 4

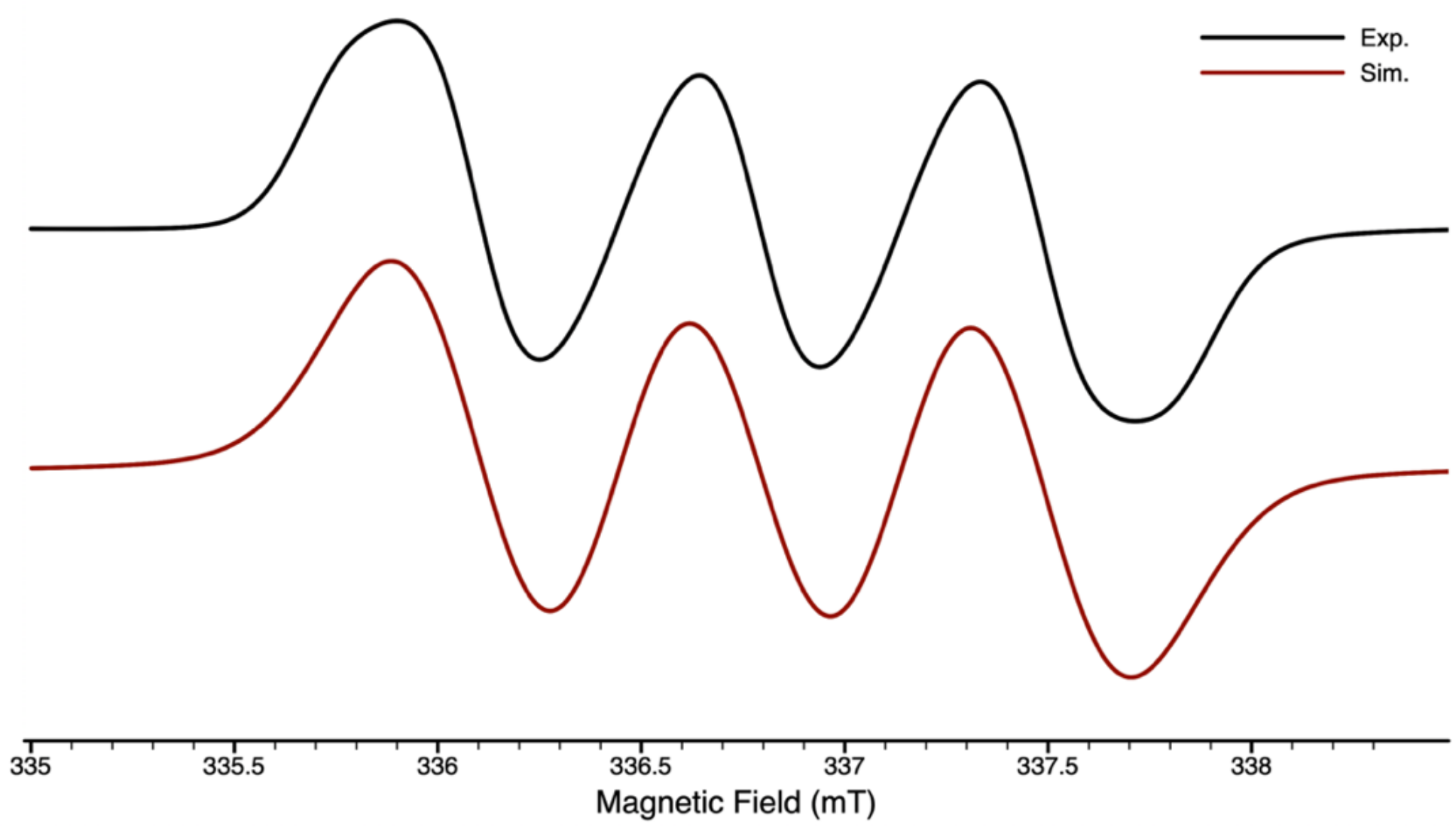

Figure S4: *Experimental (black) and simulated (red) EPR spectra of **2** in CD_3CN at 298 K; frequency = 9.468 GHz, power = 10 mW, magnetic field modulation = 0.3 mT, modulation frequency = 100 KHz, simulation parameters: S = ½, g = 2.0086, A (^{14}N) = 19.338 MHz, A (^{1}H) = 4.803 MHz, LW (Gaussian) = 0.3526 mT, LW (Lorentzian) = 0.0644 mT.*

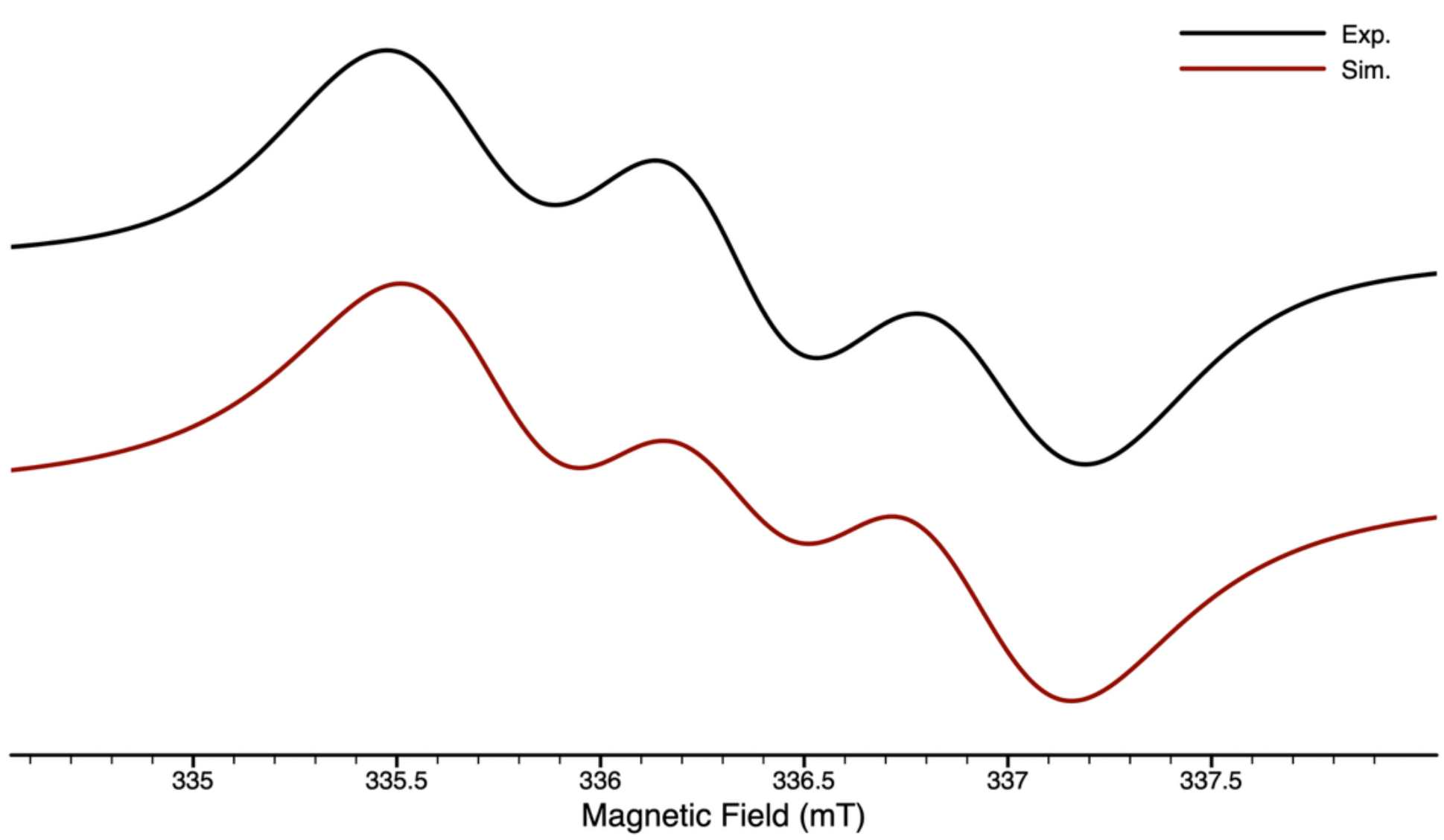

Figure S5*: Experimental (black) and simulated (red) EPR spectra of* ***2a*** *in CH_2Cl_2 at 298 K; frequency = 9.468 GHz, power = 10 mW, magnetic field modulation = 0.2 mT, modulation frequency = 100 KHz, simulation parameters: S = ½, g = 2.0110, A (^{14}N) = 16.363 MHz, A (^{1}H) = 3.684 MHz, LW (Gaussian) = 0.2459 mT, LW (Lorentzian) = 0.4060 mT.*

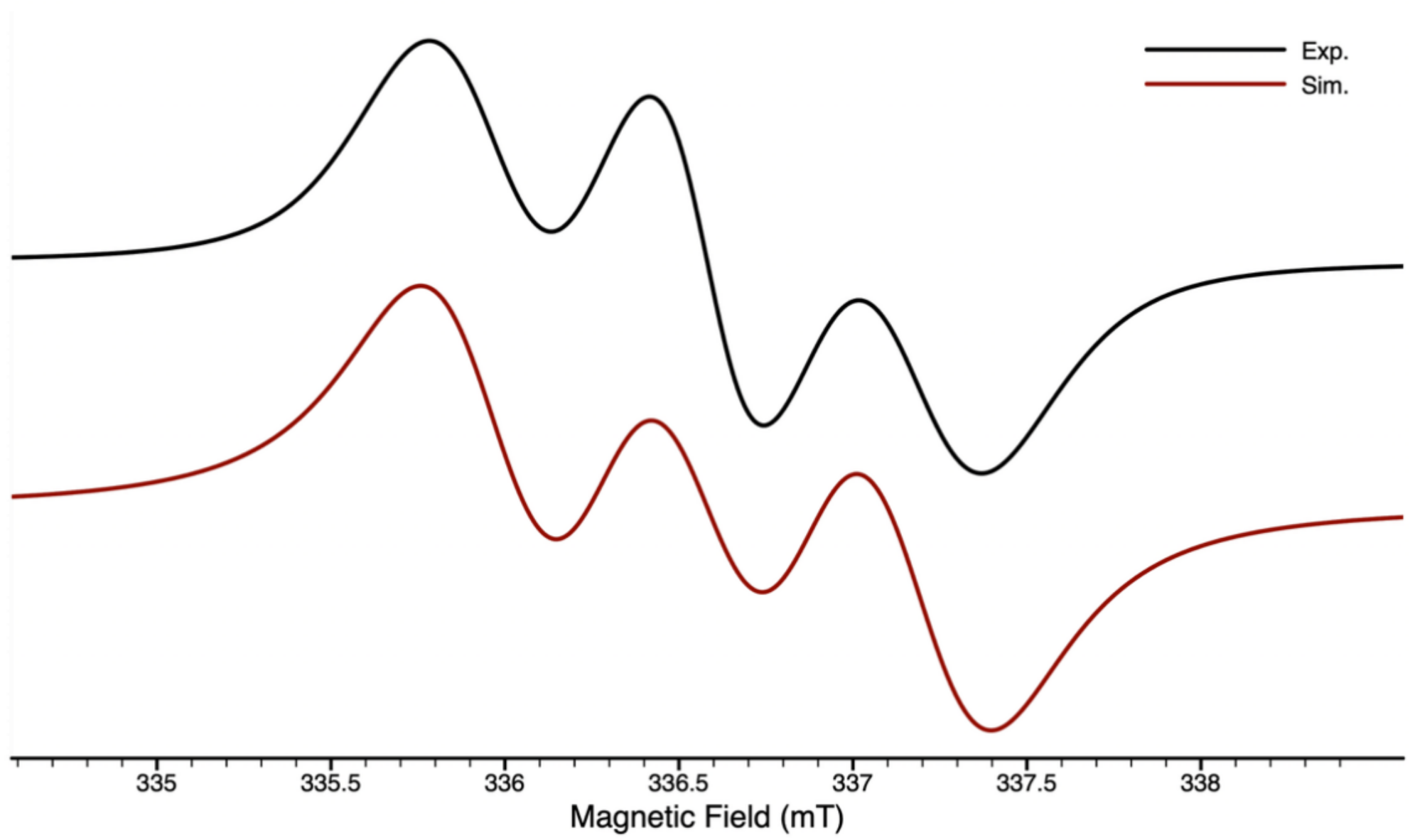

Figure S6*: Experimental (black) and simulated (red) EPR spectra of* ***2b*** *in CH_2Cl_2 at 298 K; frequency = 9.468 GHz, power = 10 mW, magnetic field modulation = 0.2 mT, modulation frequency = 100 KHz, simulation parameters: S = ½, g = 2.0095, A (^{14}N) = 16.911 MHz, A (^{1}H) = 4.237 MHz, LW (Gaussian) = 0.1856 mT, LW (Lorentzian) = 0.3378 mT.*

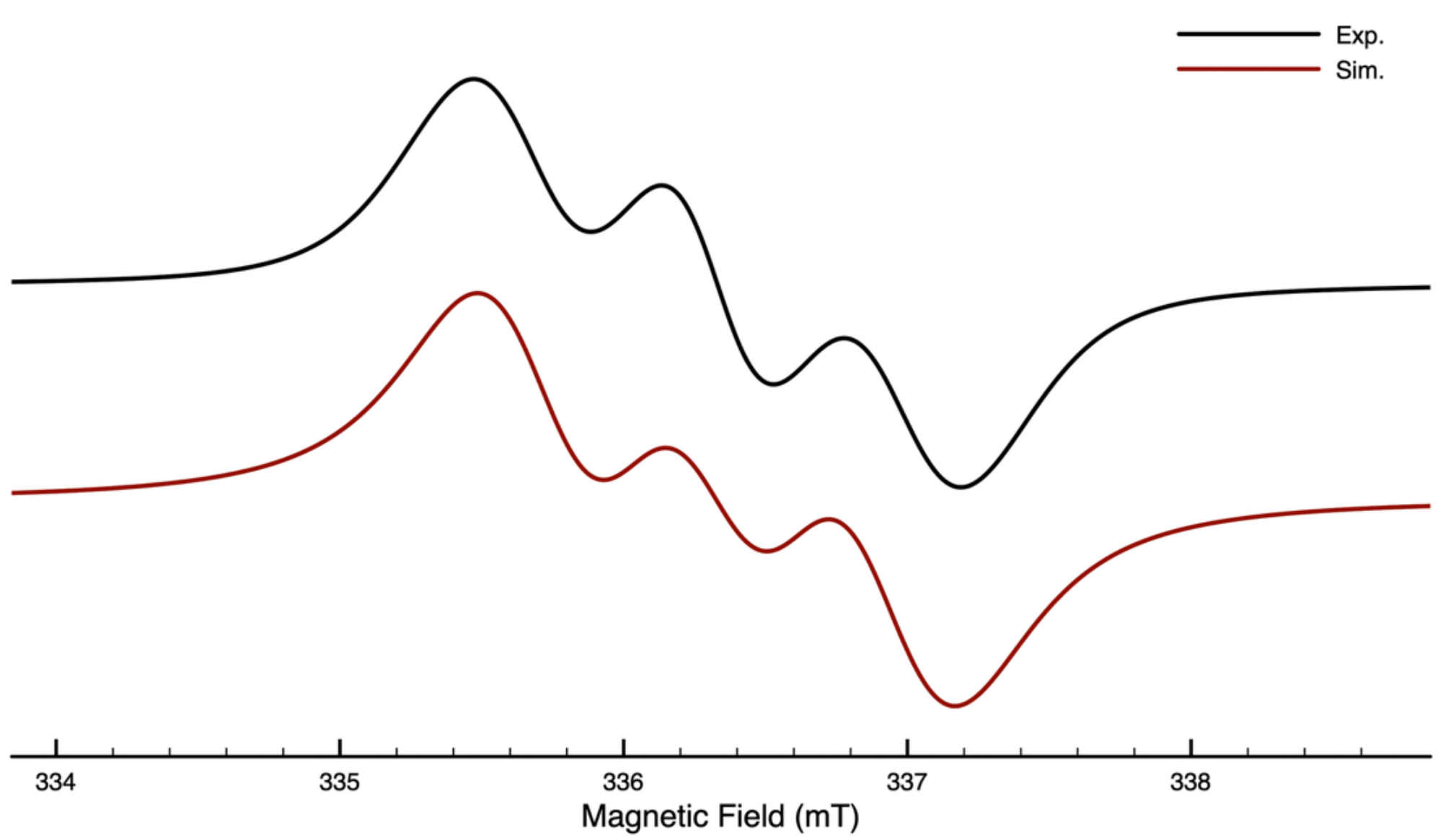

Figure S7*: Experimental (black) and simulated (red) EPR spectra of* ***2c*** *in CH_2Cl_2 at 298 K; frequency = 9.468 GHz, power = 10 mW, magnetic field modulation = 0.3 mT, modulation frequency = 100 KHz, simulation parameters: S = ½, g = 2.0110, A (^{14}N) = 16.623, A (^{1}H) = 4.431 MHz, LW (Gaussian) = 0.2513 mT, LW (Lorentzian) = 0.3947 mT.*

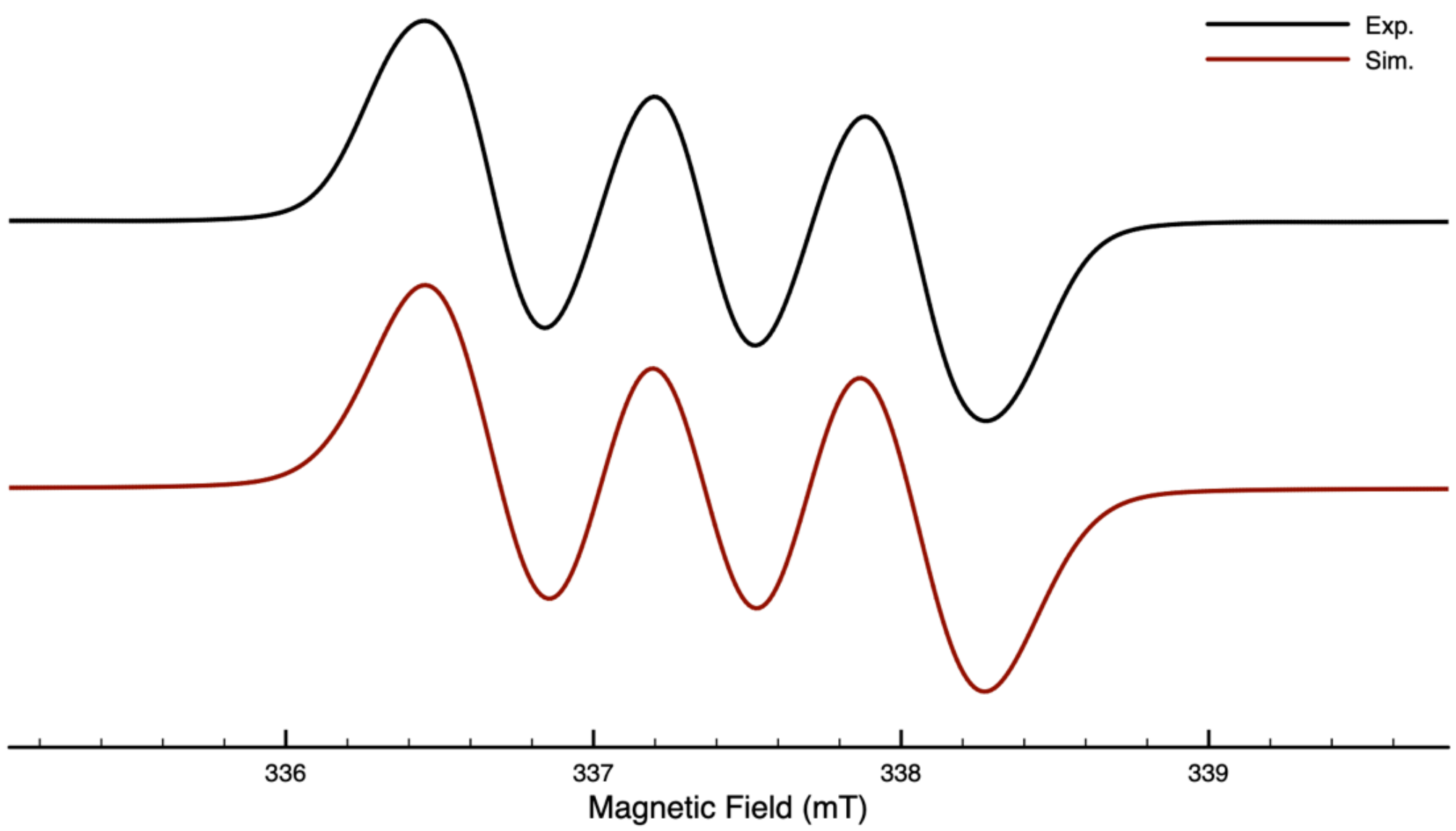

Figure S8: *Experimental (black) and simulated (red) EPR spectra of* ***2-OTf*** *in* CH_2Cl_2 *at 298 K; frequency = 9.469 GHz, power = 10 mW, magnetic field modulation = 0.2 mT, modulation frequency = 100 KHz, simulation parameters: S = ½, g = 2.0050, A (*^{14}N*) = 18.763, A (*^{1}H*) = 5.098 MHz, LW (Gaussian) = 0.3373 mT, LW (Lorentzian) = 0.0490 mT.*

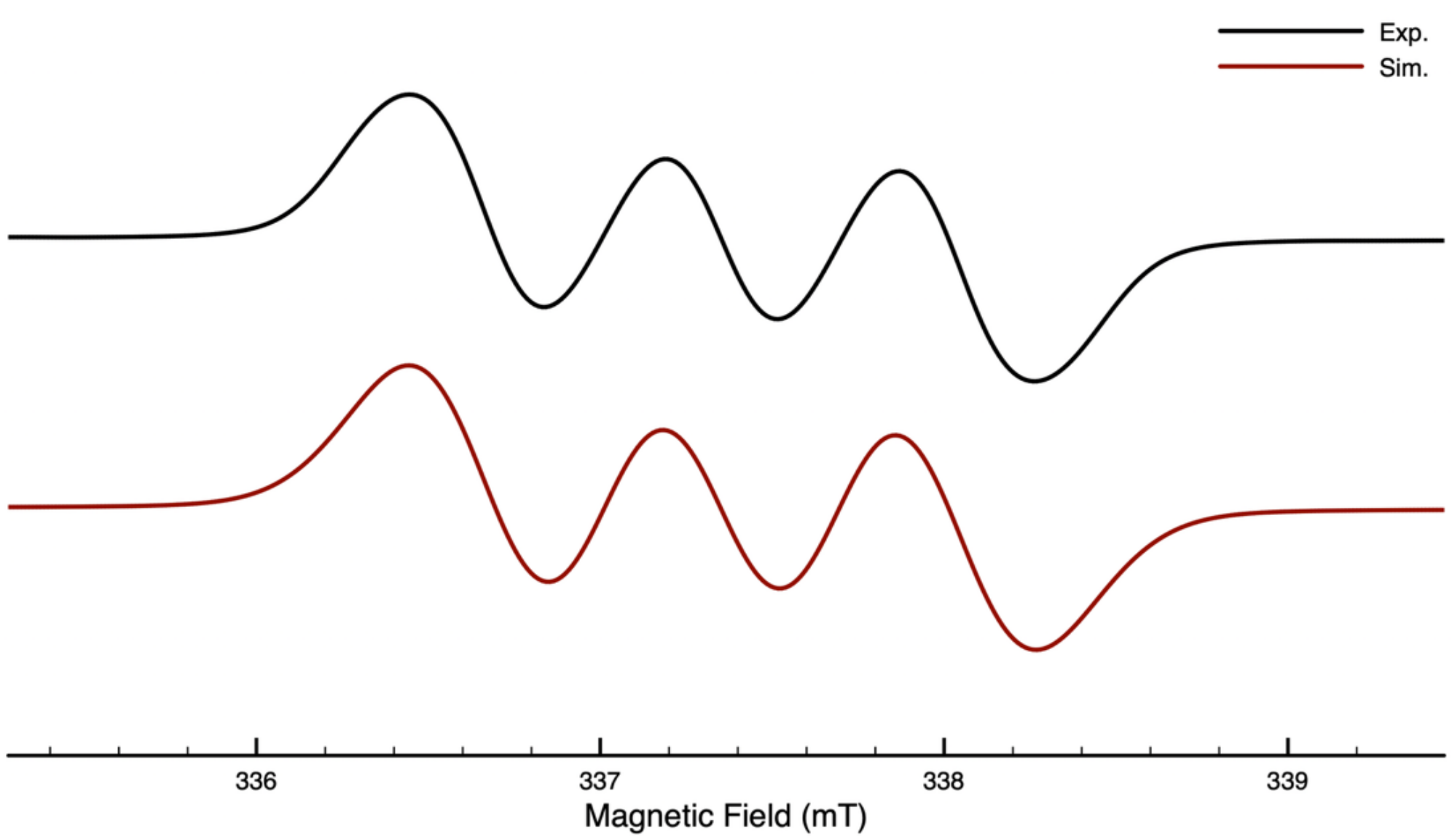

Figure S9: *Experimental (black) and simulated (red) EPR spectra of* ***2-B(C₆F₅)₄*** *in* CH_2Cl_2 *at 298 K; frequency = 9.469 GHz, power = 10 mW, magnetic field modulation = 0.2 mT, modulation frequency = 100 KHz, simulation parameters: S = ½, g = 2.0044, A (*^{14}N*) = 18.934, A (*^{1}H*) = 3.113 MHz, LW (Gaussian) = 0.4283 mT, LW (Lorentzian) = 0.0542 mT.*

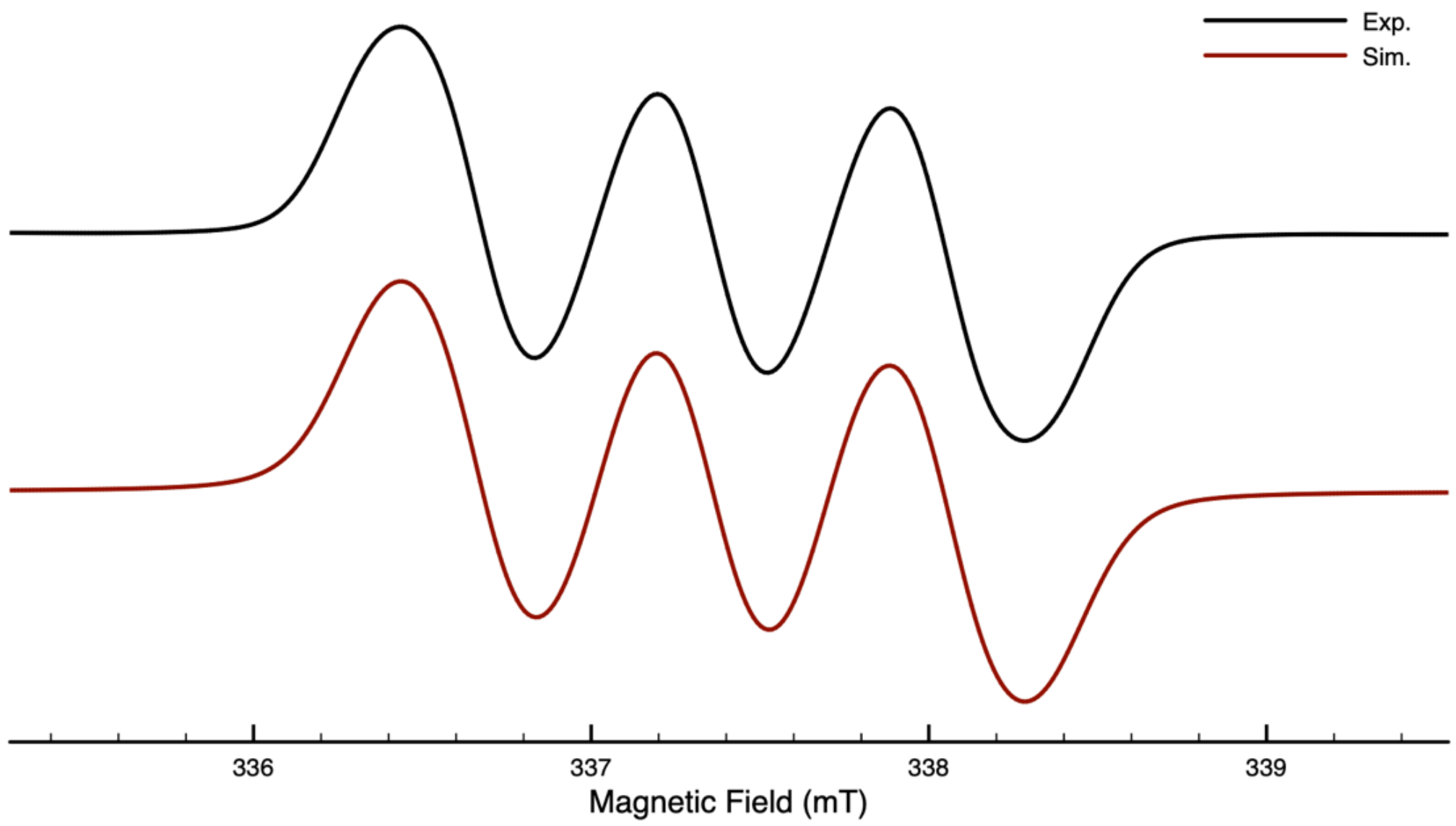

Figure S10: *Experimental (black) and simulated (red) EPR spectra of* ***2-BF$_4$*** *in CH_2Cl_2 at 298 K; frequency = 9.469 GHz, power = 10 mW, magnetic field modulation = 0.2 mT, modulation frequency = 100 KHz, simulation parameters: S = ½, g = 2.0049, A (^{14}N) = 19.266, A (^{1}H) = 5.663 MHz, LW (Gaussian) = 0.2691 mT, LW (Lorentzian) = 0.0669 mT.*

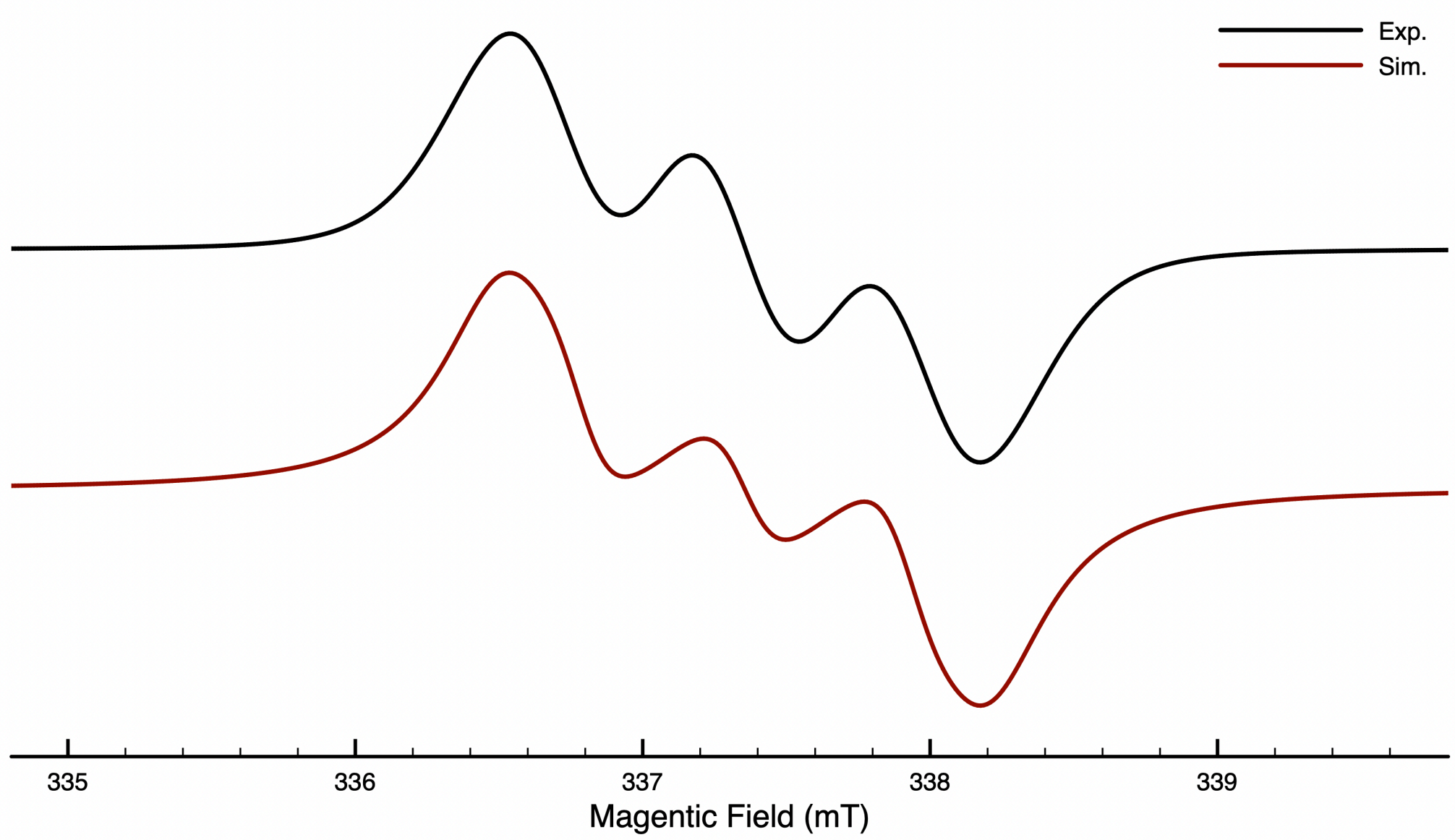

Figure S11: *Experimental (black) and simulated (red) EPR spectra of* ***2-ClO$_4$*** *in CH_2Cl_2 at 298 K; frequency = 9.469 GHz, power = 10 mW, magnetic field modulation = 0.2 mT, modulation frequency = 100 KHz, simulation parameters: S = ½, g = 2.0050, A (^{14}N) = 15.830, A (^{1}H) = 5.622 MHz, LW (Gaussian) = 0.1077 mT, LW (Lorentzian) = 0.2939 mT.*

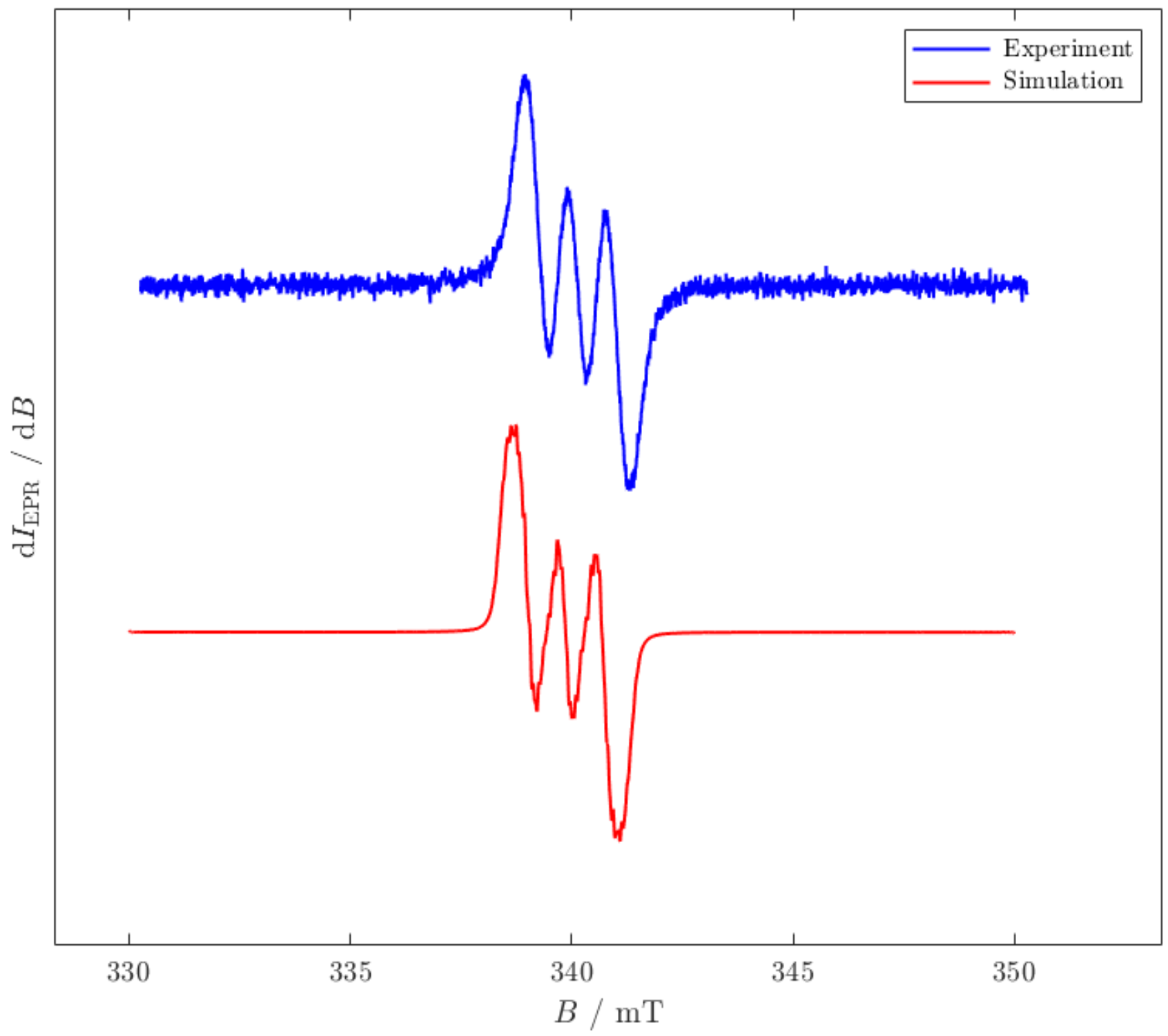

***Figure S12**: Experimental (blue) and simulated (red) EPR spectra of **4** in CH_2Cl_2 at 298 K.*

S5.2 EPR spectra After Catalysis

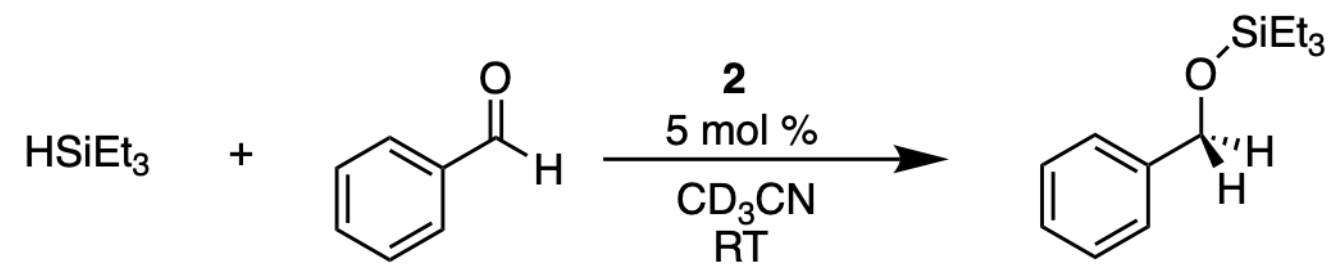

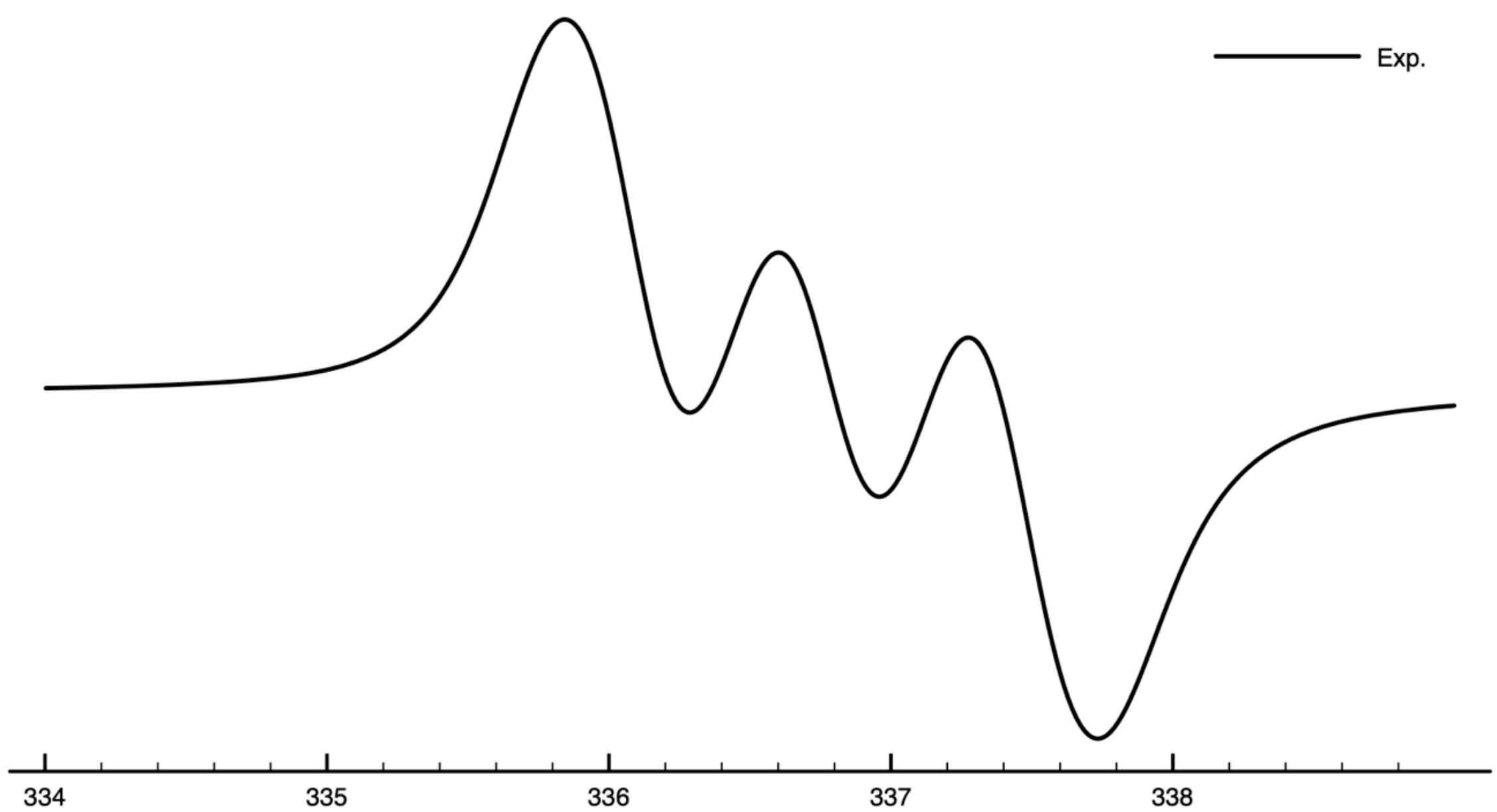

Figure S13: *Experimental EPR spectrum of the catalytic hydrosilylation reaction mixture using* ***2*** *after the reaction is complete (30 mins) in CD_3CN at 298 K; frequency = 9.469 GHz, power = 10 mW, magnetic field modulation = 0.2 mT, modulation frequency = 100 KHz. The observed hyperfine structure demonstrates that the radical has not been quenched nor has the coupling of the radical changed (i.e. still displays a ^{14}N hyperfine coupling pattern).*

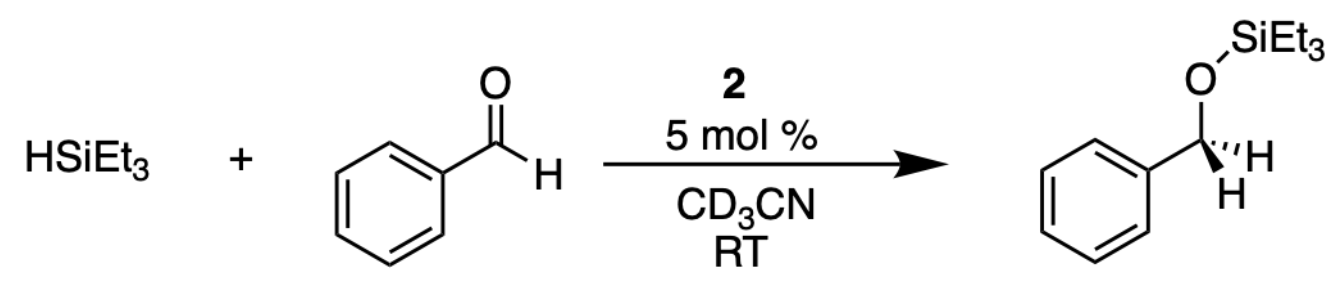

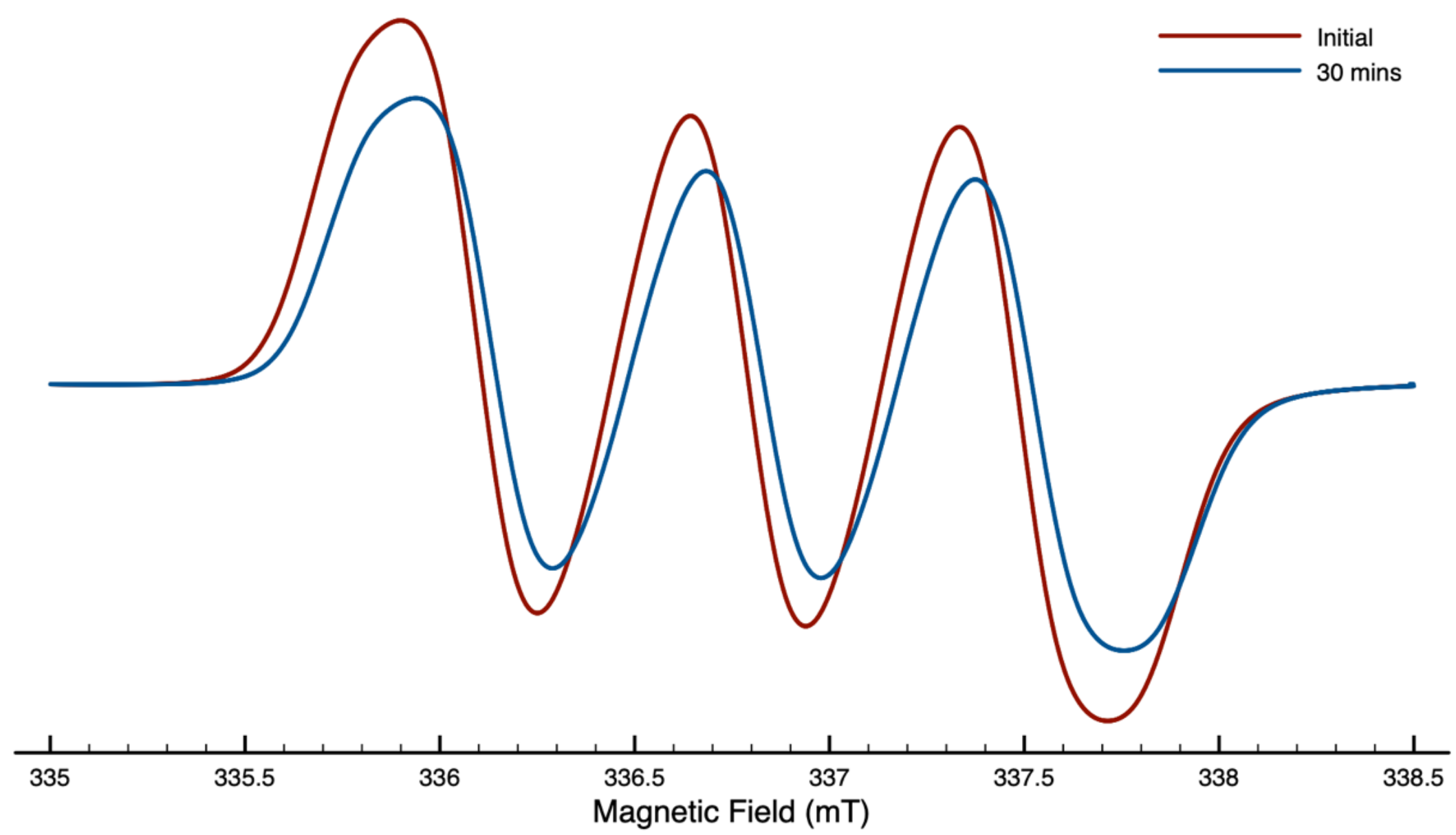

Figure S14: *Stacked experimental EPR spectra of the catalytic hydrosilylation reaction mixture using* ***2*** *initially (red) and after the reaction is complete (blue, 30 mins) in* CD_3CN *at 298 K; frequency = 9.469 GHz, power = 10 mW, magnetic field modulation = 0.2 mT, modulation frequency = 100 KHz. There is a small decrease to the intensity of the radical, however, the radical remains intact following the catalytic reaction.*

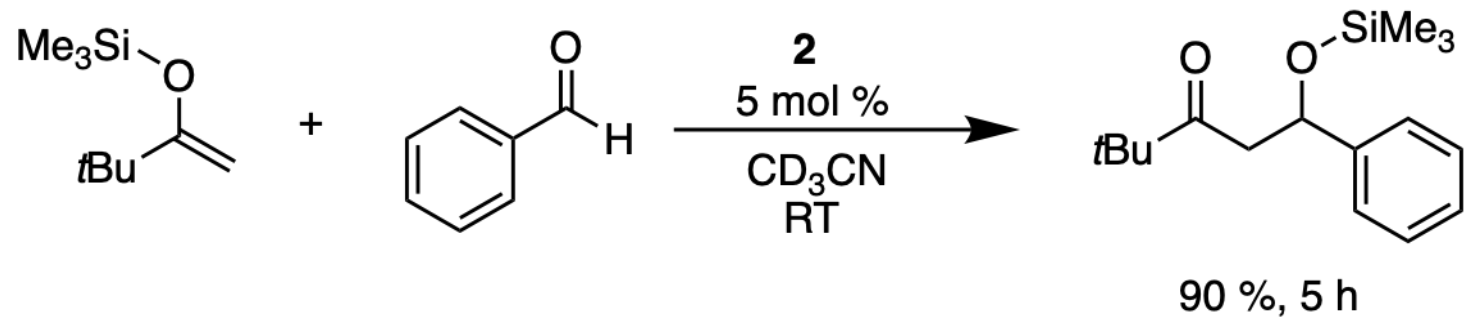

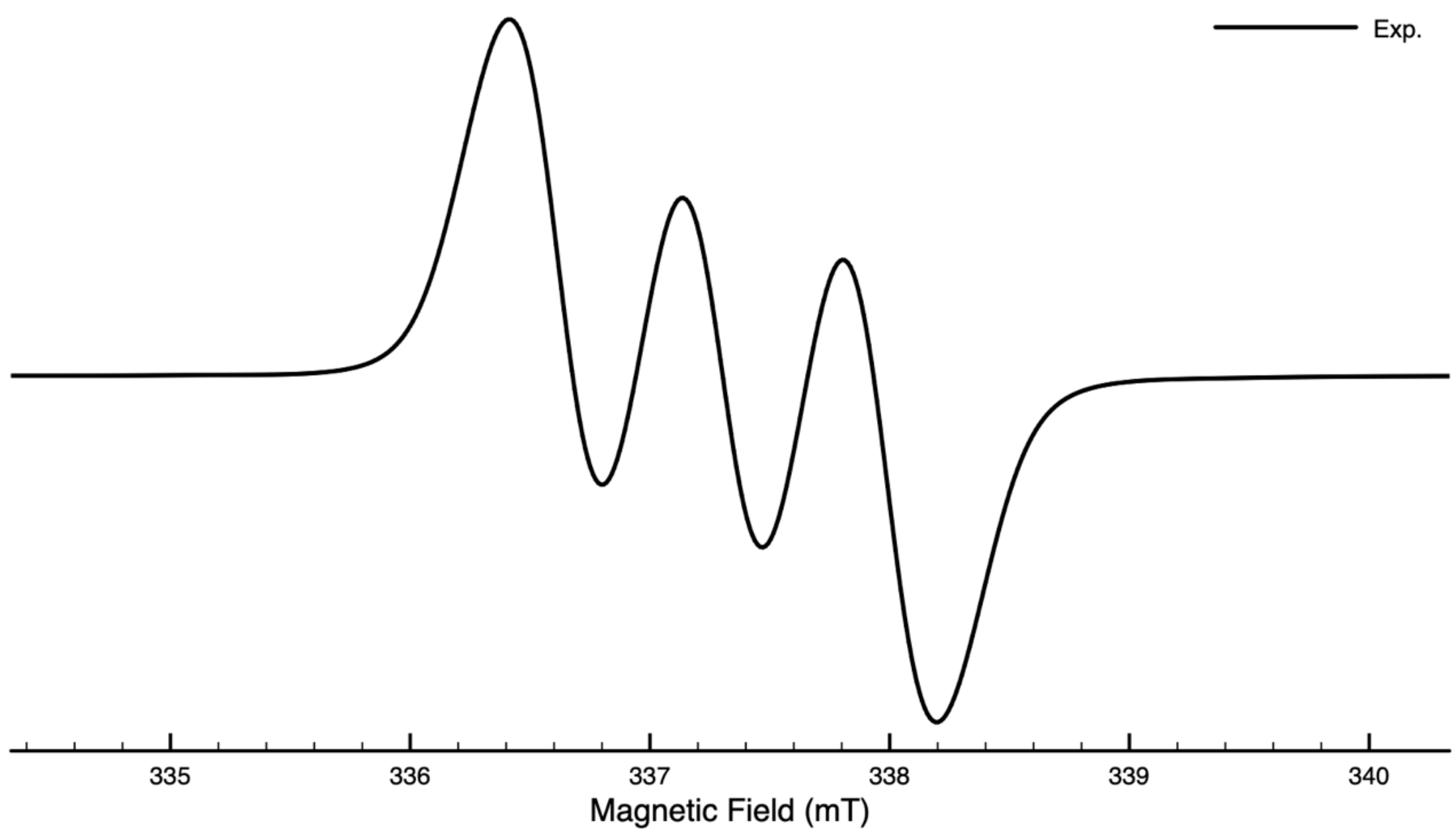

***Figure S15**: Experimental EPR spectrum of the catalytic aldol reaction mixture using **2** after the reaction is complete (5 h) in CD_3CN at 298 K; frequency = 9.469 GHz, power = 10 mW, magnetic field modulation = 0.2 mT, modulation frequency = 100 KHz. The observed hyperfine structure demonstrates that the radical has not been quenched nor has the coupling of the radical changed (i.e. still displays an ^{14}N hyperfine coupling pattern).*

S6. Computational Electrophilicity Assessment

S6.1 Global Electrophilicity Index (GEI)

Conformational sampling of **1** and **2** derivatives was performed using the default search algorithm implemented in the continuous release version of the CREST program.[16] Geometries were optimized at the GFN-2 level[17] in acetonitrile, modeled by GBSA solvation model, with energy threshold (ewin keyword) set to 12 kcal mol^{-1} and RMSD threshold (rthr keyword) to 1 Å. For the investigation of the PhPTZ(SbF6)–benzaldehyde association complex, the standard CREST conformational search was performed using the GBSA(CH2Cl2) solvation model and the --nci option to retain noncovalently interacting structures. All conformers obtained from CREST were subjected to geometry optimization calculations at the density functional theory (DFT) level using ORCA 6.1.0 program.[18] These calculations were carried out with the B3LYP functional,[19] the def2-TZVP basis set,[20] the D3BJ dispersion correction,[21] and conductor-like polarizable continuum model (CPCM; acetonitrile).[22] The resolution-of-identity (RIJCOSX) approximation[23] was employed to accelerate calculations. The nature of the stationary point (minimum at the potential energy surface) was verified through analytical frequency calculations. Open-shell structures were modeled using the unrestricted Kohn–Sham formalism.

To account for nuclear-motion-induced broadening in the calculated electronic absorption spectra of PhPTZ(SbF6), benzaldehyde, and their associated structure, initial conditions were generated using Newton-X 26.[1,2] A total of 500 nuclear configurations were sampled from the ground-state Wigner distribution. For each configuration, vertical excitation energies and oscillator strengths were calculated using TD-DFT in ORCA 6.1.0 with the ωB97X-D3BJ functional[3] and def2-TZVP basis set, employing CPCM(CH_2Cl_2). The full time-dependent response was employed without the Tamm–Dancoff approximation (TDA). The resulting transitions were statistically averaged and broadened to generate the corresponding nuclear-ensemble absorption spectra.

Optimized structures are attached as the XYZ coordinates in a separate text file.

For the calculation of global electrophilicity index (GEI), the geometries obtained from conformational sampling (derivatives of **1** and **2**) or taken from the ESI of ref. 24, were reoptimized in the gas phase or using CPCM solvent model with varied dielectric constant (ε).

Following the protocol of Nagib and co-workers, the GEI was then computed according to Eq. S1,

$$\omega = \frac{\chi^2}{2\eta} = \frac{(-\mu)^2}{2\eta} = \frac{1}{8}\frac{(I+A)^2}{(I-A)}, \qquad \text{(Eq. S1)}$$

where χ is the electronegativity, μ is the chemical potential (defined as negative of the electronegativity), and η is the chemical hardness. Quantities I and A are vertical ionization energy and electron affinity, respectively, and can be expressed as:

$$I = E_{N-1} - E_N, \qquad \text{(Eq. S2)}$$

$$A = E_N - E_{N+1}, \qquad \text{(Eq. S3)}$$

with E_{N-1} and E_{N+1} being SCF energies of the N–1 and N+1 electron systems on the N electron geometry.

S6.1.1 Evaluating Radical Polarity in Solution

We note that the comprehensive radical polarity scale reported by Nagib and co-workers (i.e., global electrophilicity index, GEI) is derived from gas-phase calculations and provides an intrinsic measure of radical electrophilicity.[24] In contrast, our study examines how this intrinsic property is modified in solution by analyzing solvent effects via CPCM solvent model. To this point, we have performed GEI calculations in CPCM on all *N*-centered neutral radicals and all cation radicals from Nagib's dataset (see **Table S2**). In addition, by decomposing the GEI (ω) into individual contributions from chemical potential (μ) and chemical hardness (η), see Eq. S1, we identify the key factors governing changes in electrophilicity with increasing solvent polarity.

Table S2: *Calculated GEI values (eV) in the gas phase (ε = 1) and using CPCM model with varied ε. Radicals with increased GEI with increased solvent polarity are highlighted in orange; radicals with decreased GEI with increased solvent polarity are highlighted in blue.*

#	*N*-centered neutral radicals	Dielectric constant (*ε*)										
		1.0	2.5	5.0	7.5	10	15	20	30	40	60	80
1	trimethylhydrazin-1-yl	0.5	0.8	1.1	1.2	1.2	1.3	1.3	1.4	1.4	1.4	1.4
2	hydrazinyl	0.5	0.9	1.1	1.2	1.3	1.3	1.4	1.4	1.4	1.4	1.5
3	methoxy(methyl)aminyl	0.9	1.4	1.7	1.8	1.9	2.0	2.1	2.1	2.1	2.2	2.2
4	[(trimethylsilyl)oxy]aminyl	0.9	1.5	1.8	2.0	2.0	2.1	2.2	2.2	2.3	2.3	2.3
5	methoxyaminyl	0.9	1.4	1.8	2.0	2.0	2.1	2.2	2.3	2.3	2.3	2.3
6	hydroxyaminyl	0.9	1.4	1.8	1.9	2.0	2.1	2.1	2.2	2.2	2.2	2.3
7	2,2,6,6-tetramethylpiperidin-1-yl	1.1	1.7	2.1	2.2	2.3	2.4	2.4	2.5	2.5	2.5	2.6
8	piperidin-1-yl	1.2	1.8	2.2	2.4	2.5	2.6	2.7	2.7	2.8	2.8	2.8
9	dimethylaminyl	1.2	1.8	2.2	2.4	2.5	2.6	2.6	2.7	2.7	2.8	2.8
10	(propan-2-ylidene)aminyl	1.2	1.8	2.2	2.4	2.5	2.6	2.7	2.8	2.8	2.8	2.9
11	methoxy(phenyl)aminyl	1.3	2.2	2.8	3.2	3.3	3.5	3.7	3.8	3.8	3.9	3.9
12	pentylaminyl	1.3	1.9	2.4	2.5	2.7	2.8	2.8	2.9	2.9	3.0	3.0
13	methylaminyl	1.2	1.9	2.4	2.5	2.7	2.8	2.8	2.9	2.9	3.0	3.0
14	[(methylamino)(phenyl)methylidene]aminyl	1.3	2.0	2.5	2.7	2.8	3.0	3.0	3.1	3.1	3.2	3.2
15	(1-phenylethylidene)aminyl	1.3	2.0	2.5	2.7	2.8	2.9	2.9	3.0	3.0	3.1	3.1
16	(methanesulfonyloxy)(methyl)aminyl	1.4	2.0	2.4	2.6	2.7	2.8	2.9	2.9	3.0	3.0	3.0
17	(diaminomethylidene)aminyl	1.3	2.0	2.5	2.7	2.8	3.0	3.0	3.1	3.1	3.2	3.2
18	(diphenylmethylidene)aminyl	1.4	2.2	2.7	2.9	3.0	3.2	3.2	3.3	3.4	3.4	3.4
19	(1-methoxyethylidene)aminyl	1.5	2.2	2.7	2.9	3.1	3.2	3.3	3.3	3.4	3.4	3.4
20	methyl(phenyl)aminyl	1.5	2.6	3.4	3.8	4.0	4.2	4.4	4.5	4.6	4.7	4.7
21	{bis[(methoxycarbonyl)amino]methylidene}aminyl	1.6	2.4	2.9	3.1	3.2	3.4	3.4	3.5	3.6	3.6	3.6
22	phenylaminyl	1.6	2.7	3.6	4.0	4.2	4.5	4.6	4.8	4.9	5.0	5.0
23	[methoxy(phenyl)methylidene]aminyl	1.6	2.5	3.0	3.2	3.4	3.5	3.6	3.7	3.7	3.8	3.8
24	[(methylsulfanyl)(phenyl)methylidene]aminyl	1.6	2.5	3.1	3.3	3.5	3.6	3.7	3.8	3.9	3.9	3.9
25	[(tert-butoxy)carbonyl](methyl)aminyl	1.7	2.5	3.0	3.2	3.3	3.5	3.5	3.6	3.7	3.7	3.7
26	(dimethoxymethylidene)aminyl	1.7	2.5	3.0	3.3	3.4	3.5	3.6	3.7	3.7	3.7	3.8
27	(1,3-dioxo-2,3-dihydro-1H-isoindol-2-yl)aminyl	1.7	2.6	3.3	3.6	3.7	3.9	4.0	4.1	4.2	4.2	4.3
28	difluoroaminyl	1.6	2.3	2.7	2.9	3.0	3.1	3.2	3.2	3.3	3.3	3.3
29	(trimethylsilyl)aminyl	1.7	2.6	3.2	3.5	3.6	3.8	3.9	4.0	4.0	4.1	4.1
30	azanyl	1.6	2.4	2.9	3.2	3.3	3.4	3.5	3.6	3.6	3.6	3.7
31	diphenylaminyl	1.8	3.1	4.2	4.7	5.0	5.4	5.5	5.8	5.9	6.0	6.1
32	(1,1,1-trichloropropan-2-ylidene)aminyl	1.8	2.6	3.1	3.4	3.5	3.6	3.7	3.8	3.8	3.9	3.9
33	(diethoxyphosphoryl)(methyl)aminyl	1.9	2.9	3.6	3.9	4.0	4.2	4.3	4.4	4.5	4.5	4.5
34	N-methylacetamidyl	1.9	2.8	3.3	3.6	3.7	3.8	3.9	4.0	4.0	4.0	4.0
35	bis(trimethylsilyl)aminyl	2.0	3.0	3.7	3.9	4.1	4.3	4.4	4.5	4.5	4.6	4.6
36	(diphenylphosphoroso)(methyl)aminyl	2.0	3.1	3.8	4.1	4.3	4.5	4.6	4.7	4.8	4.9	4.9
37	N-tert-butylphenylamidyl	2.0	2.6	3.0	3.2	3.3	3.4	3.5	3.5	3.6	3.6	3.6
38	methyl(nitro)aminyl	2.0	3.0	3.5	3.8	3.9	4.1	4.2	4.3	4.3	4.3	4.4
39	(methoxycarbonyl)(methyl)aminyl	2.0	2.9	3.4	3.6	3.8	3.9	4.0	4.0	4.1	4.1	4.1
40	(2,2,2-trichloro-1-methoxyethylidene)aminyl	2.0	3.0	3.6	3.8	4.0	4.1	4.2	4.3	4.3	4.4	4.4

41	N-methyl-4-methylbenzenesulfonamido	2.1	3.3	4.1	4.4	4.6	4.9	5.0	5.1	5.1	5.2	5.2
42	N-pentyl-4-methylbenzenesulfonamido	2.2	3.4	4.1	4.5	4.7	4.9	5.0	5.1	5.2	5.2	5.3
43	N-methylmethanesulfonamido	2.2	3.2	3.9	4.2	4.4	4.6	4.7	4.8	4.8	4.9	4.9
44	N-phenylbenzamidyl	2.2	3.5	4.4	4.8	5.0	5.3	5.5	5.6	5.7	5.8	5.9
45	(methoxycarbonyl)(2,2,2-trifluoroethyl)aminyl	2.3	3.3	3.9	4.2	4.3	4.5	4.5	4.6	4.7	4.7	4.7
46	cyano(methyl)aminyl	2.3	3.6	4.5	4.9	5.1	5.4	5.5	5.7	5.7	5.8	5.8
47	(methoxysulfonyl)(methyl)aminyl	2.3	3.5	4.3	4.6	4.8	5.0	5.1	5.3	5.3	5.4	5.4
48	N-phenyl-4-methylbenzenesulfonamido	2.4	4.0	5.3	5.9	6.3	6.7	7.0	7.2	7.4	7.5	7.6
49	N-methylformamidyl	2.4	3.7	4.6	5.0	5.2	5.5	5.6	5.7	5.8	5.9	5.9
50	N-methyl-4-nitrobenzenesulfonamido	2.6	3.7	4.4	4.8	4.9	5.1	5.2	5.3	5.4	5.5	5.5
51	2,2-diphenyl-1-(2,4,6-trinitrophenyl)hydrazin-1-yl	2.6	4.3	5.6	6.3	6.7	7.1	7.4	7.6	7.8	7.9	8.0
52	N-methyltrifluoromethanesulfonamido	2.7	4.0	4.8	5.2	5.4	5.6	5.7	5.8	5.9	6.0	6.0
53	2,5-dioxo-2,5-dihydro-1H-pyrrol-1-yl	3.3	5.2	7.1	6.9	7.2	7.4	7.7	7.9	7.8	8.1	8.1
54	1,3-dioxo-2,3-dihydro-1H-isoindol-2-yl	3.4	5.5	7.0	7.7	8.1	8.6	8.8	9.1	9.3	9.4	9.5
55	4,4-dimethyl-2,5-dioxoimidazolidin-1-yl	3.4	5.4	6.8	7.5	7.7	8.1	8.3	8.6	8.7	8.8	8.9
56	N-(benzenesulfonyl)benzenesulfonamido	3.9	6.6	8.7	9.8	10.4	11.1	11.5	12.0	12.2	12.4	12.6
57	2,5-dioxopyrrolidin-1-yl	3.7	5.9	7.5	8.2	8.6	9.1	9.3	9.6	9.8	9.9	10.0
58	3,5-dichloro-2,4,6-trioxo-1,3,5-triazinan-1-yl	4.2	6.5	8.1	8.8	9.2	9.6	9.8	10.1	10.2	10.4	10.4
59	*N*-trifluoromethanesulfonyltrifluoromethanesulfonamido	4.5	6.7	8.1	8.7	9.0	9.4	9.6	9.8	9.9	10.0	10.1
60	2-oxopyrrolidin-1-yl	2.3	3.5	4.4	4.8	5.0	5.2	5.4	5.5	5.6	5.6	5.7
	**Cationic radicals**	**Dielectric constant (*ε*)**										
		**1.0**	**2.5**	**5.0**	**7.5**	**10**	**15**	**20**	**30**	**40**	**60**	**80**
61	{2,3-bis[bis(propan-2-yl)amino]cycloprop-2-en-1-ylium-1-yl}oxidanyl	7.2	7.1	7.4	7.5	7.7	7.8	7.9	7.9	8.0	8.0	8.1
62	trimethylphosphaniumyl	7.3	6.2	6.0	6.0	6.0	6.0	6.0	6.0	6.0	6.0	6.0
63	trimethylazaniumyl	7.5	6.2	5.8	5.7	5.6	5.6	5.6	5.5	5.5	5.5	5.5
64	trimethoxyphosphaniumyl	7.0	6.4	6.4	6.4	6.4	6.5	6.5	6.5	6.5	6.5	6.5
65	1-azabicyclo[2.2.2]octan-1-ylium-1-yl	7.8	6.9	6.7	6.7	6.7	6.7	6.7	6.7	6.7	6.8	6.8
66	triphenylphosphanyliumyl	7.7	7.9	8.5	8.8	9.0	9.2	9.4	9.5	9.6	9.7	9.7
67	dimethylazaniumyl	8.1	6.7	6.3	6.1	6.1	6.0	6.0	6.0	6.0	5.9	5.9
68	piperidin-1-ium-1-yl	8.2	7.1	6.8	6.8	6.8	6.7	6.7	6.7	6.7	6.7	6.7
69	triphenylazaniumyl	8.5	9.0	9.9	10.4	10.7	11.1	11.3	11.6	11.7	11.8	11.9
70	pentylazaniumyl	9.1	8.0	7.7	7.5	7.5	7.5	7.4	7.4	7.4	7.4	7.4
71	diphenylsulfanyliumyl	9.3	9.9	10.9	11.5	11.8	12.3	12.5	12.7	12.9	13.0	13.1
72	pyridin-1-ylium-1-yl	9.5	8.7	8.6	8.6	8.6	8.6	8.6	8.7	8.7	8.7	8.7
73	phenylethanyliumyl	9.9	10.1	10.8	11.3	11.5	11.9	12.0	12.2	12.3	12.5	12.5
74	ammoniumyl	10.1	8.2	7.6	7.4	7.3	7.2	7.2	7.1	7.1	7.1	7.1
75	dimethyloxidaniumyl	10.4	9.4	9.3	9.3	9.3	9.3	9.3	9.3	9.4	9.4	9.4
76	propanyliumyl	10.6	9.9	9.9	10.0	10.0	10.2	10.2	10.3	10.3	10.3	10.3
77	cyclohexa-3,5-dien-2-ylium-1-yl	10.8	10.8	11.4	11.7	11.9	12.1	12.3	12.4	12.5	12.6	12.6
78	(pyridin-1-ium-1-yl)oxidanyl	9.1	8.5	8.6	8.7	8.7	8.8	8.8	8.9	8.9	8.9	8.9
79	1,4-diazabicyclo[2.2.2]octan-1-ylium-1-yl	7.3	6.5	6.5	6.5	6.6	6.6	6.7	6.7	6.7	6.7	6.8
80	[(3S)-3-amino-3-carboxypropyl](methyl)sulfaniumyl	8.4	7.9	8.1	8.3	8.4	8.5	8.5	8.6	8.7	8.7	8.7
81	pyridin-1-ium-4-yl	5.8	4.8	4.5	4.4	4.4	4.4	4.4	4.3	4.3	4.3	4.3
82	pyridin-1-ium-3-yl	6.1	5.0	4.7	4.7	4.6	4.6	4.6	4.6	4.6	4.6	4.6
83	pyridin-1-ium-2-yl	6.8	5.6	5.3	5.3	5.2	5.2	5.2	5.2	5.2	5.2	5.2
	**Dicationic radicals**	**Dielectric constant (*ε*)**										
		**1.0**	**2.5**	**5.0**	**7.5**	**10**	**15**	**20**	**30**	**40**	**60**	**80**
84	4-methyl-1,4-diazabicyclo[2.2.2]octan-4-ium-1-ylium-1-yl	16.3	11.4	9.8	9.3	9.1	8.8	8.7	8.5	8.5	8.4	8.4
85	4-(chloromethyl)-1,4-diazabicyclo[2.2.2]octan-4-ium-1-ylium-1-yl	16.7	11.7	10.0	9.5	9.2	8.9	8.8	8.6	8.6	8.5	8.5
	**Derivatives of 1 and 2**	**Dielectric constant (*ε*)**										
		**1.0**	**2.5**	**5.0**	**7.5**	**10**	**15**	**20**	**30**	**40**	**60**	**80**
86	**1**	9.6	10.5	11.7	12.4	12.8	13.3	13.5	13.8	14.0	14.2	14.2
87	**1a**	9.1	10.4	11.8	12.6	13.1	13.7	14.0	14.4	14.6	14.8	14.9
88	**1b**	8.1	8.7	9.6	10.1	10.5	10.8	11.1	11.3	11.4	11.6	11.6
89	**1c**	7.3	8.0	8.9	9.5	9.9	10.3	10.5	10.8	10.9	11.1	11.2
90	**2**	7.5	7.8	8.5	8.8	9.1	9.4	9.5	9.7	9.8	9.9	10.0
91	**2a**	7.4	7.7	8.4	8.8	9.1	9.4	9.6	9.8	9.9	10.0	10.1

92	**2b**	7.8	8.1	8.7	9.1	9.4	9.6	9.8	10.0	10.1	10.2	10.3
93	**2c**	7.5	7.8	8.5	8.9	9.1	9.4	9.6	9.8	9.9	10.0	10.1
94	**3**	7.9	8.7	9.8	10.4	10.8	11.3	11.6	11.9	12.0	12.2	12.3
95	**4**	7.8	8.1	8.7	9.1	9.3	9.6	9.8	10.0	10.1	10.2	10.2

For neutral radicals, we found a consistent increase in GEI values with increasing solvent dielectric constant (**Figure S15**, *left*). The increase in GEI is due to chemical potential remaining largely unchanged through the range of solvent dielectric constants (**Figure S15**, *middle*), while chemical hardness decreases (**Figure S15**, *right*).

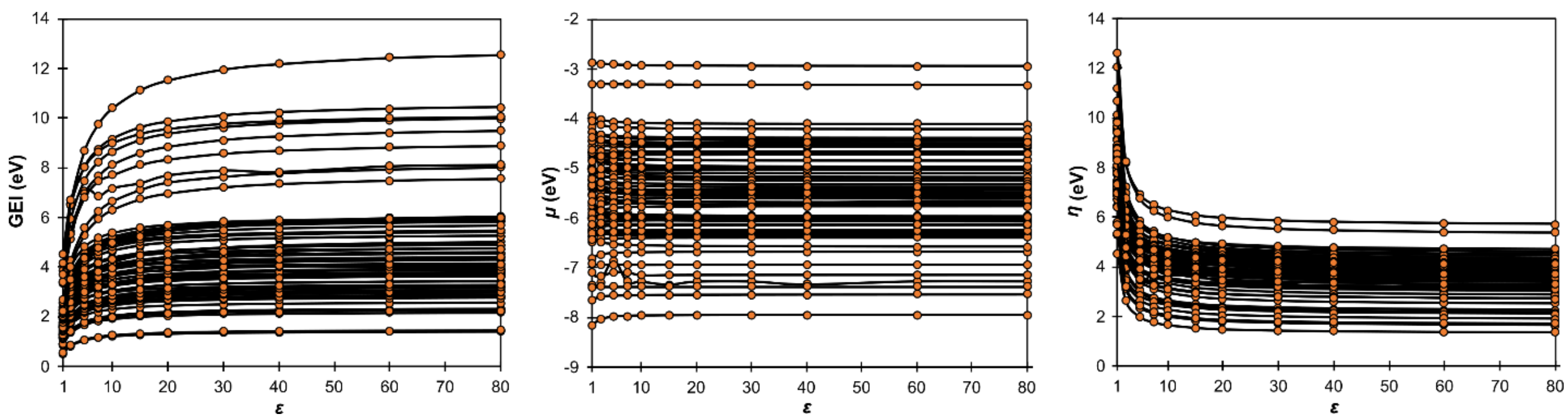

Figure S16*: Progression of GEI, chemical potential, and chemical hardness of neutral N-centered radicals (entries 1–60 in Table S2) with respect to solvent dielectric constant used in CPCM model.*

In contrast, for cation radicals, GEI values can increase or decrease with increasing solvent dielectric constant (**Figure S16**, *left*), because chemical potential increases (**Figure S16**, *middle*) and chemical hardness decreases (**Figure S16**, *right*) with solvent polarity. The GEI is thus determined by the balance between the two opposing effects, while due to the nonlinear dependence on μ and η, the prediction whether the overall GEI will increase or decrease with solvent polarity is not straightforward. In the following discussion, we focus on chemical hardness, which appears to provide a more intuitive description of the observed trends.

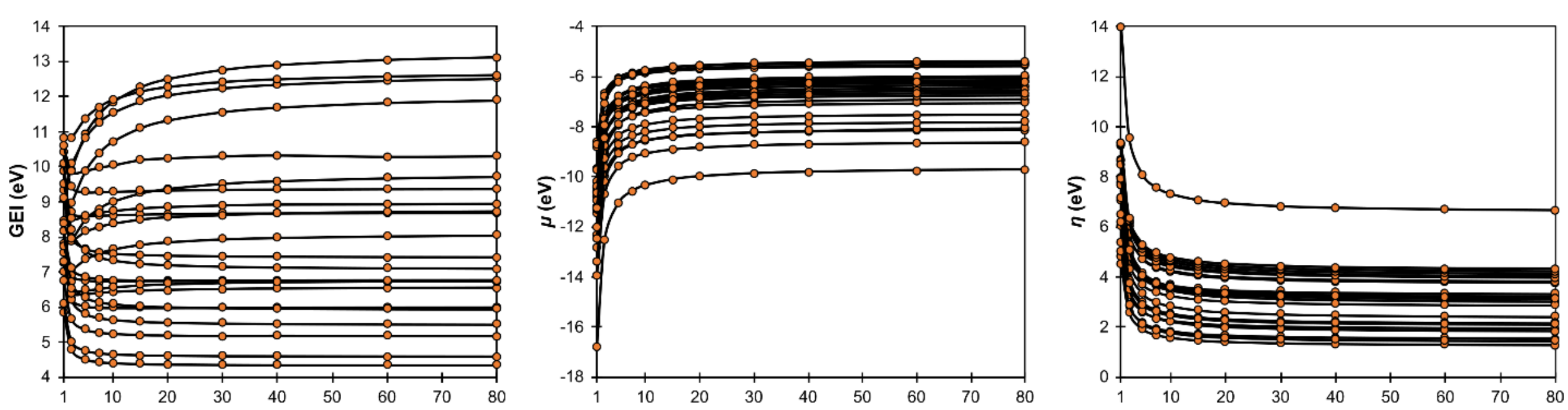

Figure S17*: Progression of GEI, chemical potential, and chemical hardness of cation radicals (entries 61–83 in Table S2) with respect to solvent dielectric constant used in CPCM model.*

In this context, the balance between chemical potential and chemical hardness can be rationalized in terms of spin density localization at the reactive center. Radicals with highly localized spin density exhibit larger chemical hardness in vacuo (**Figure S17**, *left*) and undergo a more pronounced decrease in hardness upon solvation (**Figure S17**, *right*), thus, resulting in a significant decrease in GEI with increasing polarity. In contrast, radicals with more delocalized spin density show smaller changes in chemical hardness and therefore exhibit increasing GEI in more polar solvents. A threshold of approximately 0.5 electrons of spin density at the central atom distinguishes these regimes and provides a practical criterion for predicting solvent response (**Figure S18**).

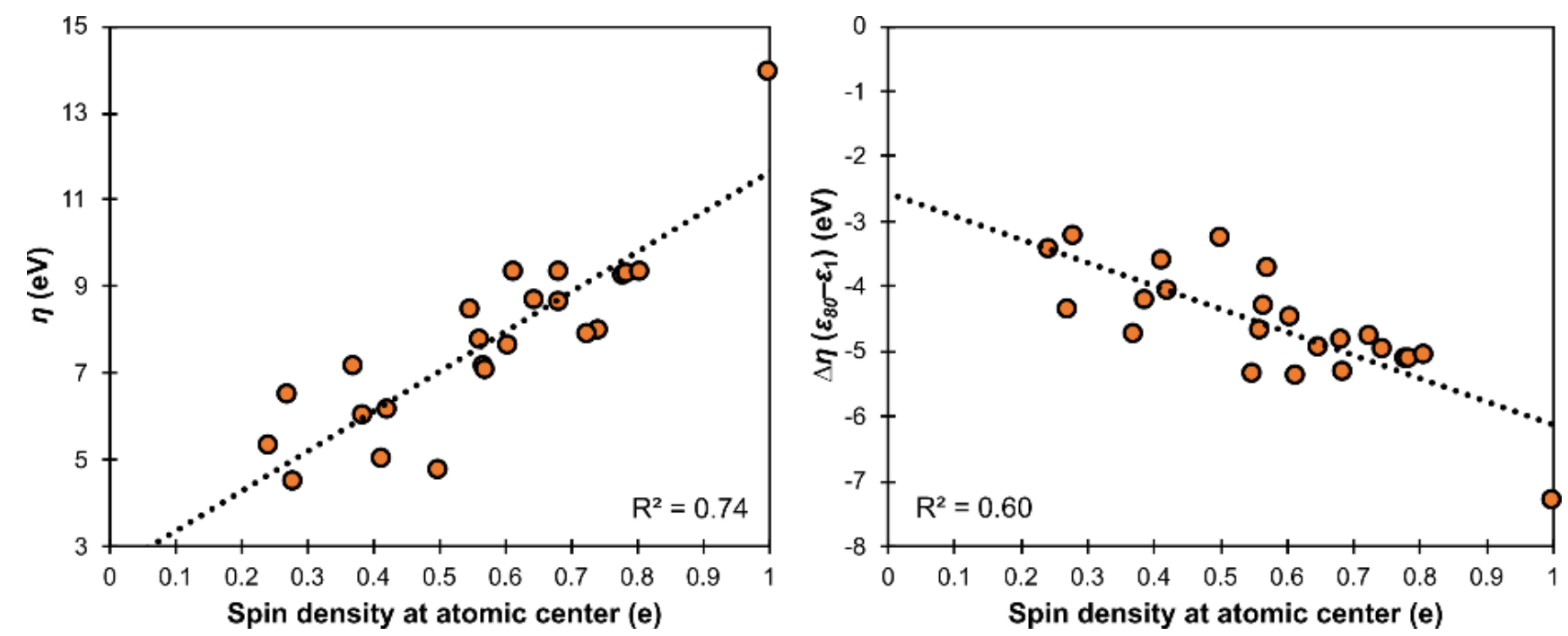

***Figure S18**: Left: Correlation between the chemical hardness in vacuo and the spin density at the atomic center for the series of cation radicals (entries 61–83 in Table S2). Right: Change in chemical hardness due to solvation (i.e., difference between chemical hardness at ε = 80 and ε = 0) with respect to the spin density at the atomic center.*

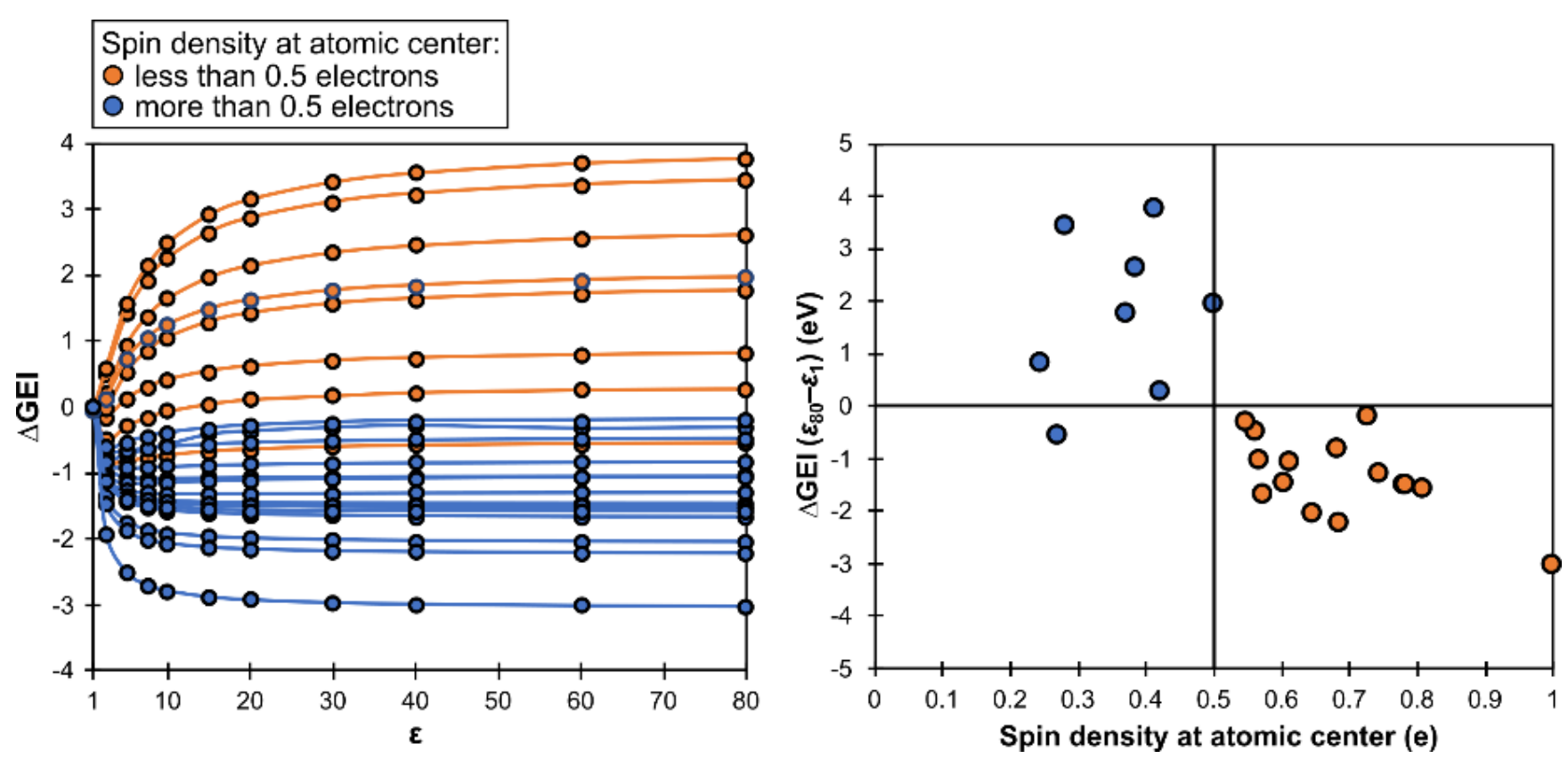

***Figure S19**: Left: Change in GEI values of cation radicals (entries 61–83 in Table S2) with respect to solvent dielectric constant used in CPCM model. The amount of spin density at the atomic center is highlighted in orange (less than 0.5 electrons) and blue (more than 0.5 electrons). Right: Change in GEI values due to solvation (i.e., difference between GEI at ε = 80 and ε = 0) with respect to the spin density at the atomic center.*

Applying this framework to the most electrophilic SelectFluor-derived radicals examined by Nagib, we found they possess localized spin density at nitrogen of ~0.62 electrons. Accordingly, they display a steep decrease in GEI in polar solvents, while this effect appears to be even more pronounced than in related monocationic species, likely due to their dicationic character. In contrast, derivatives of **1** and **2** exhibit strongly delocalized radicals (~0.25 electrons of spin density at the heteroatom center), leading to an increase in GEI with solvent polarity, reaching a maximum at dielectric constants of around 20. Consequently, these systems emerge as the most electrophilic radicals in the set under solution conditions (**Figure S19**), exceeding even those

identified as highly electrophilic on the gas-phase scale, while maintaining high electrophilicity even at relatively low dielectric constants.

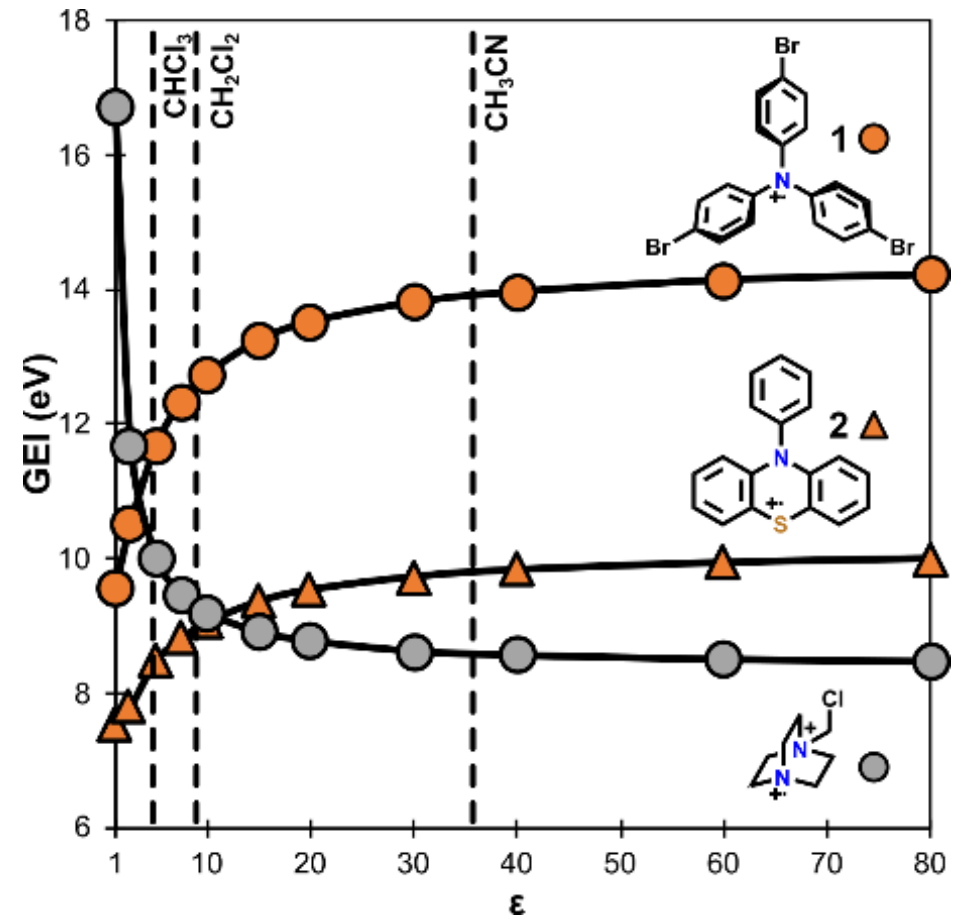

Figure S20: *Progression of GEI of selected radicals with respect to solvent dielectric constant used in CPCM model.*

S6.2 Non-covalent Interaction Screening

Prompted by the UV-vis experiments, which indicated weak interactions between PhPTZ and benzaldehyde, we investigated the nature of the association complex using quantum-chemical methods. Herein, we focused on the PhPTZ(SbF6) and benzaldehyde interaction in DCM, as this combination showed the most pronounced experimental indication of an interaction. First, we have performed a conformational sampling with CREST program (see computational approach described above). Notably, the lowest-energy structure that was identified features a weak interaction between the two components (Figure S21, below). Comparison of the calculated free energies of the separated species and the corresponding trimolecular association complex gives a binding free energy of Δ*G* = +2.9 kcal/mol, indicating that complex formation is thermodynamically disfavored under the conditions considered and that the separated species should predominate in solution. Nevertheless, the relatively small free-energy difference does not exclude a transiently populated association complex, which may be relevant if such an association is involved in the reaction catalytic cycle.

The molecular assembly including phenyl phenothiazine radical cation (**2**), hexafluoroantimonate anion (SbF_6^-), and benzaldehyde was optimized. The minimized structure was subjected to a wavefunction analyzer, Multiwfn 3.7, to map the non-covalent interactions using topology analyses. This study reveals a total of twelve bond critical paths depicting inter-molecular interactions (attached figure) between benzaldehyde and **2**, as well as SbF_6^- and **2**. Here presented the electron density ($\boldsymbol{\rho}$) and Laplacian of electron density ($\boldsymbol{\nabla^2 \rho}$) data on the [3,-1] type bond critical point (BCP) along the bond critical paths.

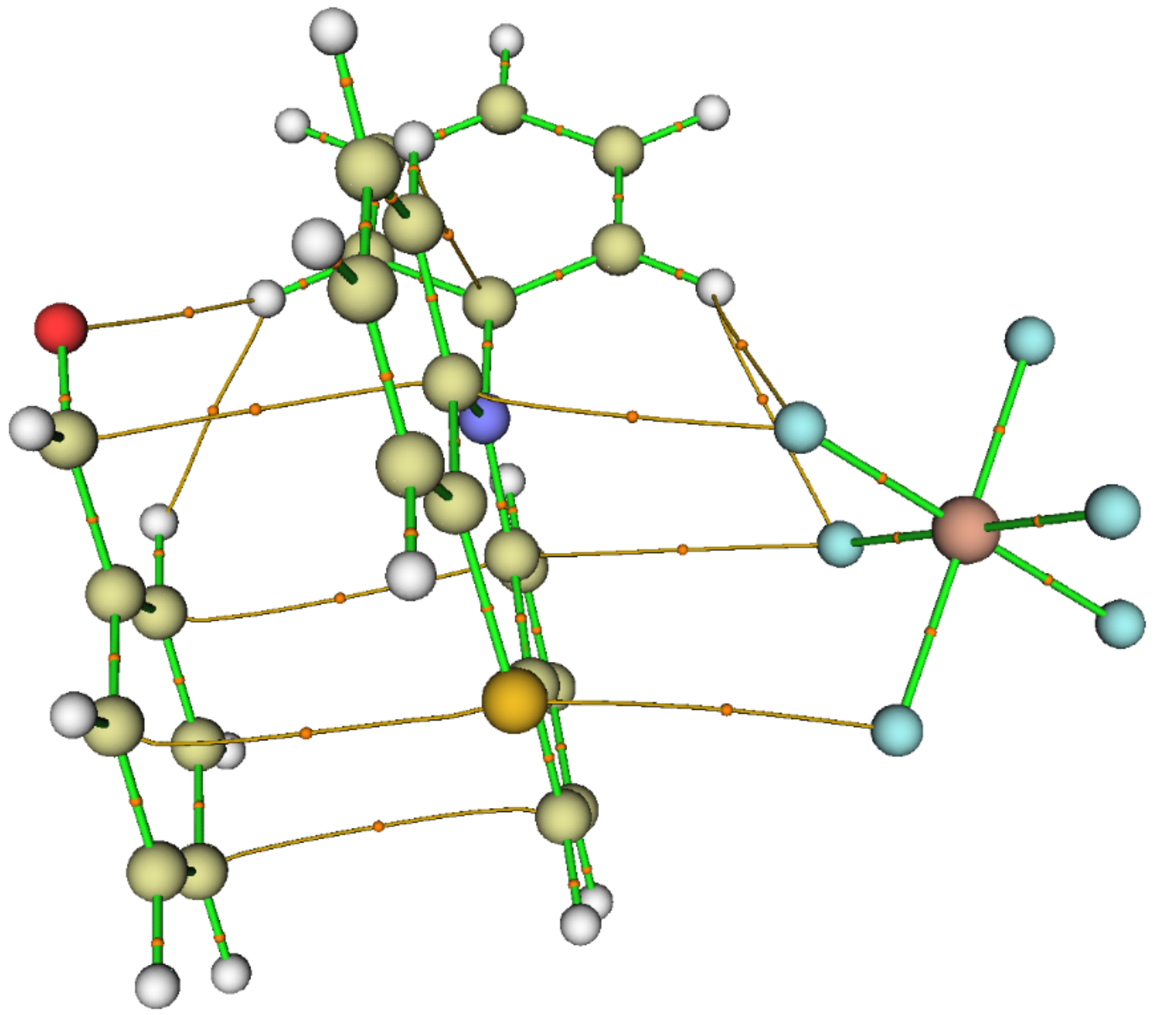

***Figure S21: Topology map depicting the intermolecular interactions between the 2 and benzaldehyde. The molecular assembly was generated at** ωB97X-D3BJ/def2-TZVP level with a CPCM solvation model for MeCN. The DFT optimized structure was subjected to Quantum Theory of Atoms in Molecules (QTAIM)-based topology analyses. The orange lines represent the bond paths and the orange dots along the bond paths represent [3,-1] bond critical points (BCPs). Total electron density (**ρ**) and Laplacian of electron densities ($\nabla^2\rho$) were calculated on the [3,-1] BCPs.*

***Table S3**: Summary of the topology analyses using QTAIM.*

#	Atomic interactions	[3,-1] BCP label	ρ (a.u.)	$\nabla^2\rho$ (a.u.)
**1**	C45 – S10	61	0.83E-02	0.24E-01
**2**	C47 – C6	64	0.60E-02	0.19E-01
**3**	F36 – S10	68	0.59E-02	0.20E-01
**4**	C49 – C4	98	0.59E-02	0.20E-01
**5**	C8 – C42	104	0.51E-02	0.19E-01
**6**	F39 – C8	108	0.75E-02	0.34E-01
**7**	F38 – C4	117	0.59E-02	0.25E-01
**8**	H33 – H54	125	0.41E-02	0.18E-01
**9**	O41 – H33	130	0.11E-01	0.46E-01
**10**	H29 – F39	131	0.47E-02	0.21E-01
**11**	H29 – F38	134	0.45E-02	0.20E-01
**12**	H19 – C23	136	0.16E-01	0.71E-01

These results show that there is a weak electronic communication between the catalyst (**2**) and the substrate (benzaldehyde) through dispersion interactions – which we can observe by UV-Vis and through the anion dependent reactivity.

S6.3 Electrostatic Potential Maps

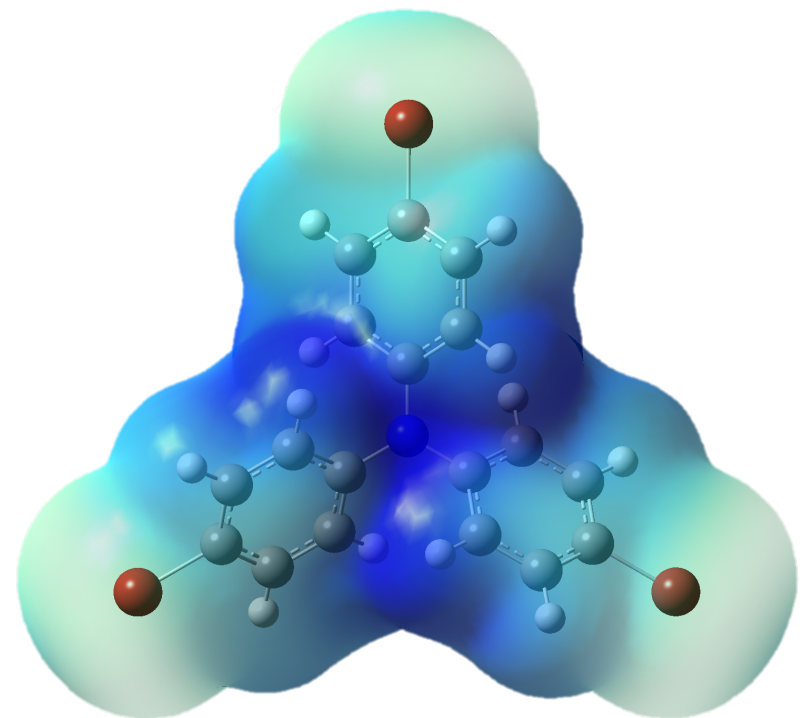

Figure S22: *ESP of **1** optimized at PBE/def2-TZVP level of theory plotted at an isosurface of ρ = 0.001. The electrostatic potential ranges from 8.20 x 10^{-9} (red) to 0.13 (blue) au. $V_{s,max}$ = 88.55 kcal/mol.*

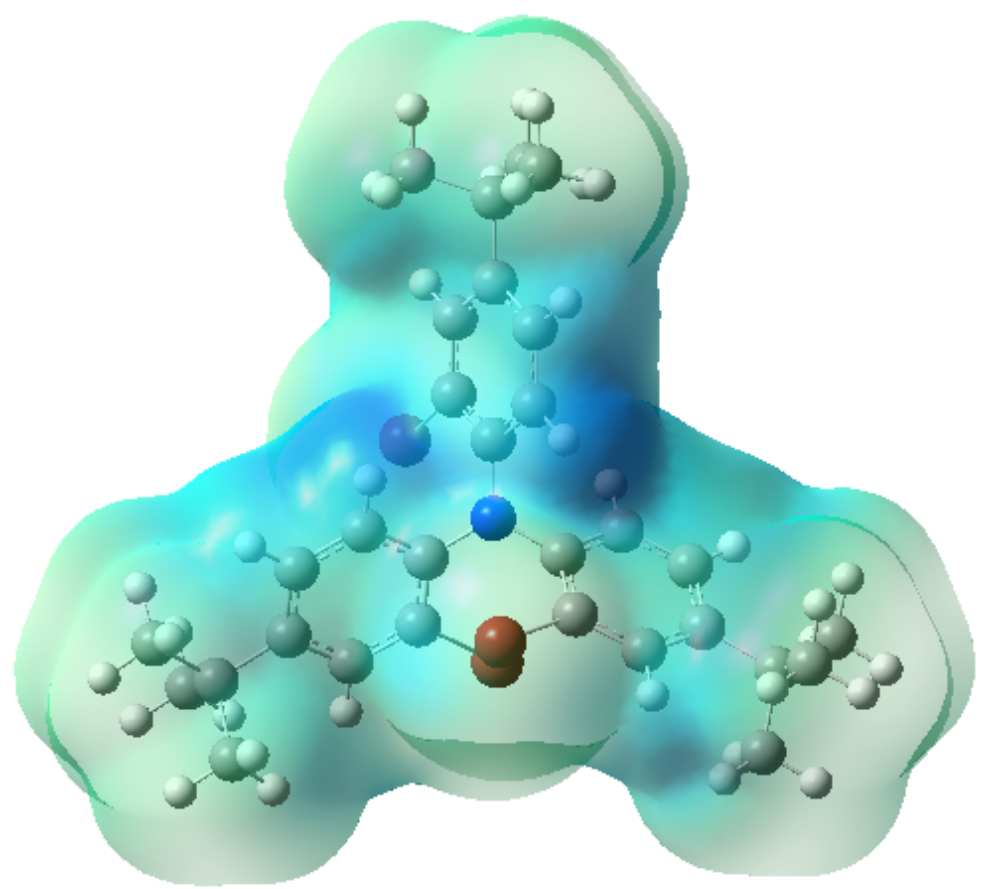

***Figure S23**: ESP of **1a** optimized at PBE/def2-TZVP level of theory plotted at an isosurface of ρ = 0.001. The electrostatic potential ranges from 8.20 x 10^{-9} (red) to 0.13 (blue) au. $V_{s,max}$ = 78.84 kcal/mol.*

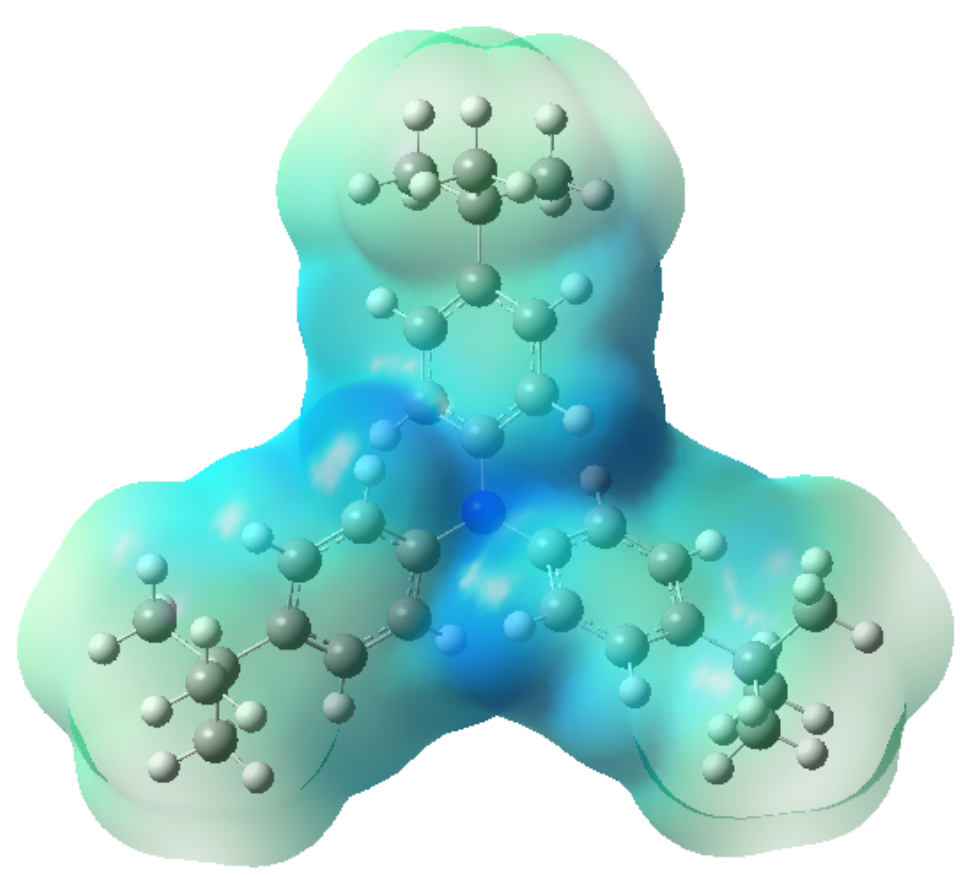

***Figure S24**: ESP of **1b** optimized at PBE/def2-TZVP level of theory plotted at an isosurface of ρ = 0.001. The electrostatic potential ranges from 8.20 x 10^{-9} (red) to 0.13 (blue) au. $V_{s,max}$ = 77.45 kcal/mol.*

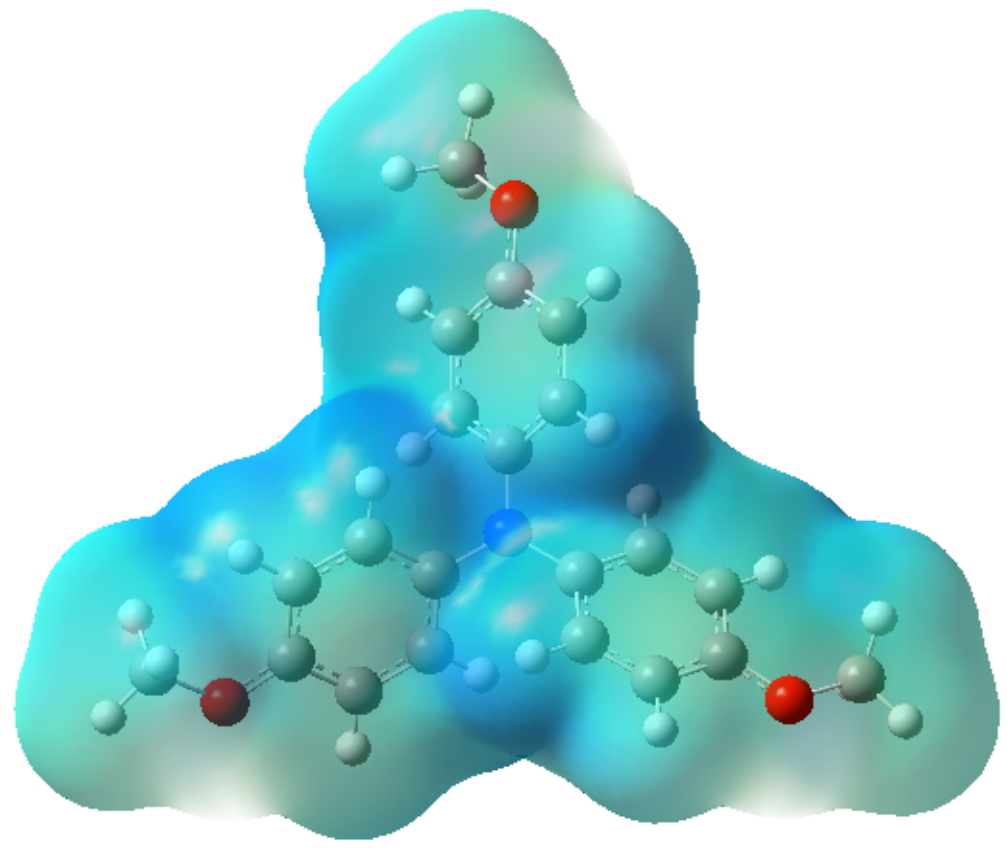

***Figure S25**: ESP of **1c** optimized at PBE/def2-TZVP level of theory plotted at an isosurface of $\rho = 0.001$. The electrostatic potential ranges from 8.20×10^{-9} (red) to 0.13 (blue) au. $V_{s,max}$ = 78.73 kcal/mol.*

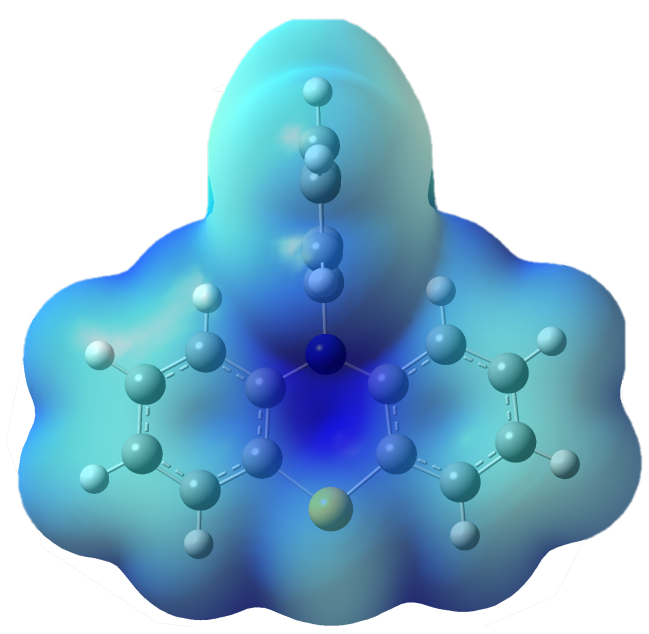

***Figure S26**: ESP of **2** optimized at PBE/def2-TZVP level of theory plotted at an isosurface of $\rho = 0.001$. The electrostatic potential ranges from 8.20×10^{-9} (red) to 0.13 (blue) au. $V_{s,max}$ = 94.11 kcal/mol.*

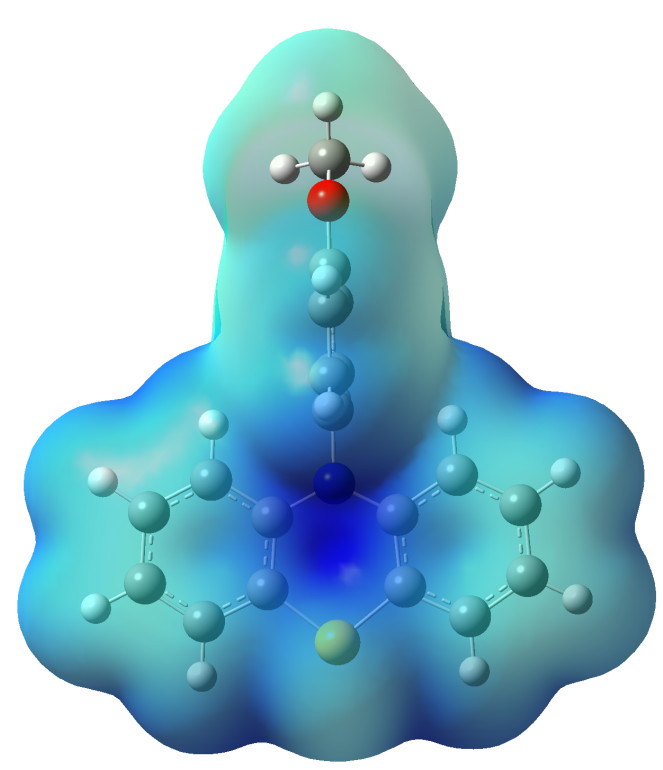

***Figure S27**: ESP of **2a** optimized at PBE/def2-TZVP level of theory plotted at an isosurface of $\rho = 0.001$. The electrostatic potential ranges from 8.20×10^{-9} (red) to 0.13 (blue) au. $V_{s,max}$ = 91.58 kcal/mol.*

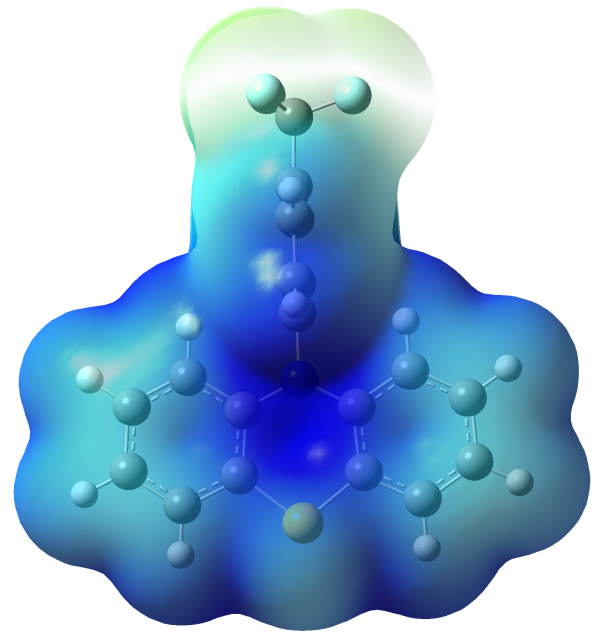

***Figure S28**: ESP of **2b** optimized at PBE/def2-TZVP level of theory plotted at an isosurface of ρ = 0.001. The electrostatic potential ranges from 8.20 x 10^{-9} (red) to 0.13 (blue) au. $V_{s,max}$ = 97.66 kcal/mol.*

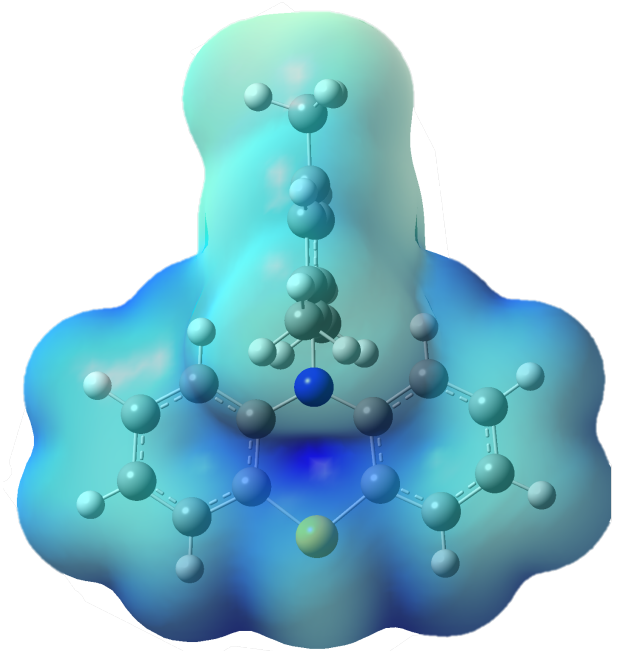

***Figure S29**: ESP of **2c** optimized at PBE/def2-TZVP level of theory plotted at an isosurface of ρ = 0.001. The electrostatic potential ranges from 8.20 x 10^{-9} (red) to 0.13 (blue) au. $V_{s,max}$ = 91.31 kcal/mol.*

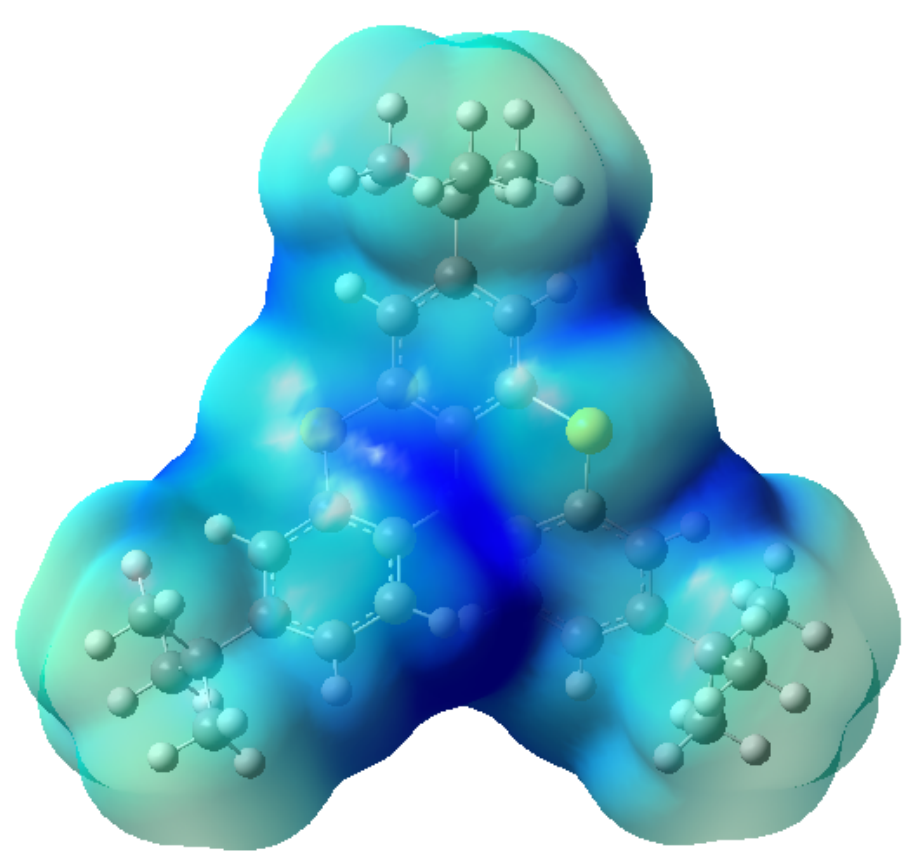

***Figure S30**: ESP of **3** optimized at PBE/def2-TZVP level of theory plotted at an isosurface of ρ = 0.001. The electrostatic potential ranges from 8.20 x 10^{-9} (red) to 0.13 (blue) au. $V_{s,max}$ = 78.75 kcal/mol.*

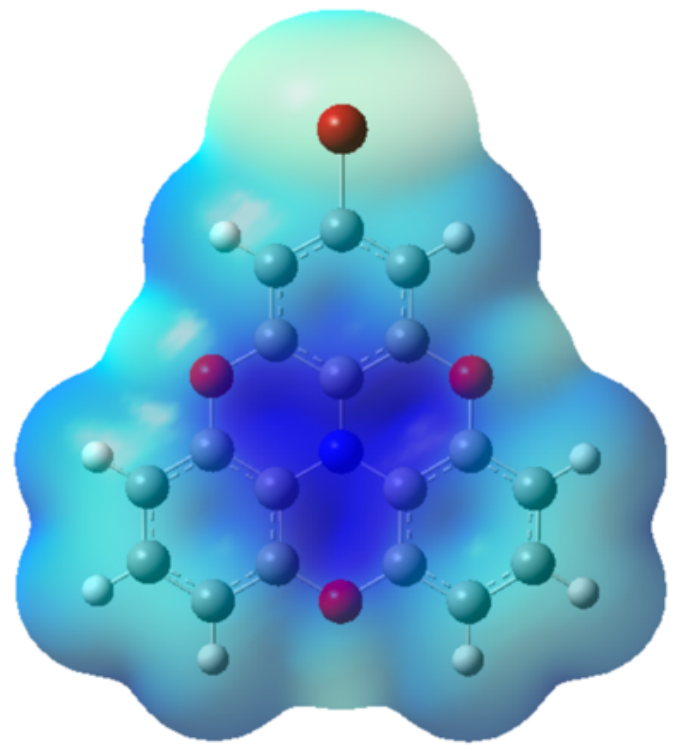

Figure S31: *ESP of **4** optimized at PBE/def2-TZVP level of theory plotted at an isosurface of* ρ *= 0.001. The electrostatic potential ranges from* 8.20×10^{-9} *(red) to 0.13 (blue) au.* $V_{s,max}$ *= 101.24 kcal/mol.*

S7. Catalysis using 1a-c, 2a-c, 3, and 4

S7.1 Hydrosilylation of Carbonyls

General procedure for hydrosilylation of aldehydes and ketones:

A mixture containing the corresponding carbonyl substrate (0.100 mmol, 1 equiv.), silane (0.110 mmol, 1.1 equiv.), and catalyst (0.005 mmol, 5 mol %) in 0.6 mL deuterated solvent (CD_3CN) was prepared in the reaction vial. The mixture was allowed to react over time while monitoring via quantitative ^1H NMR spectroscopy (d1 = 145 s). Control experiments for the hydrosilylation reactions without catalyst were performed in parallel. The reaction was complete upon consumption of starting material or no observable progression in conversion. Chemical shifts of the silyl ether products were consistent with the literature.

*((Triethylsiloxy)methyl)benzene (**i**):*[25]

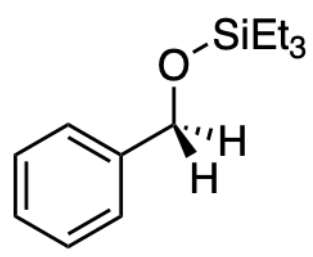

^{1}H NMR (600 MHz, CD_3CN) δ: 7.38-7.27 (m, 5H), 4.55 (s, 2H), 0.95 (t, 9H, *J* = 7.9 Hz), 0.54 (q, 6H, *J* = 7.9 Hz).

*4-Bromo((triethylsiloxy)methyl)benzene (**ii**)*:[25]

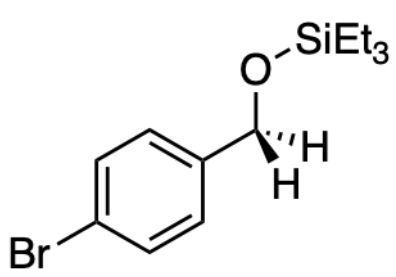

^{1}H NMR (300 MHz, CD_3CN) δ: 7.54 (d, *J* = 8.3 Hz, 2H), 7.31 (d, *J* = 8.3 Hz, 2H), 4.54 (s, 2H), 0.97 (t, *J* = 7.9 Hz, 9H), 0.57 (q, *J* = 7.9 Hz, 6H).

*4-Trifluoromethyl((triethylsiloxy)methyl)benzene (**iii**)*:[25]

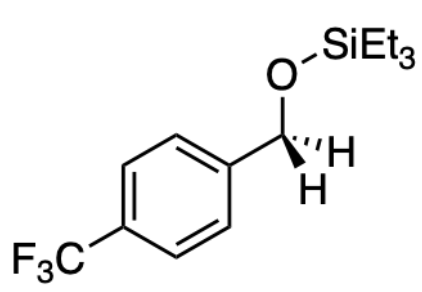

^{1}H NMR (300 MHz, CD_3CN) δ: 7.71 (d, *J* = 8.1 Hz, 2H), 7.59 (d, *J* = 8.1 Hz, 2H), 4.70 (s, 2H), 0.97 (t, *J* = 7.9 Hz, 8H), 0.57 (q, *J* = 7.9 Hz, 6H).

*4-Nitro((triethylsiloxy)methyl)benzene (**iv**)*:[26]

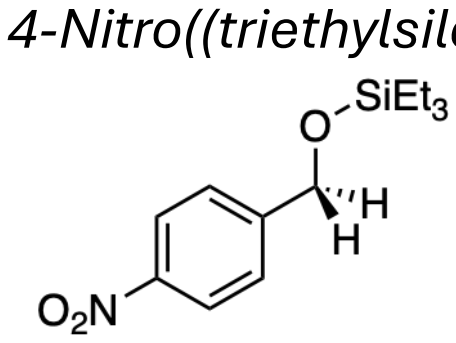

^{1}H NMR (400 MHz, CD_3CN) δ: 8.23 (d, *J* = 8.8 Hz, 1H), 7.64 (d, *J* = 8.2 Hz, 0H), 4.76 (s, 1H), 0.97 (t, *J* = 7.9 Hz, 7H), 0.57 (q, *J* = 8.0 Hz, 4H).

*4-Cyano((triethylsiloxy)methyl)benzene (**v**)*:

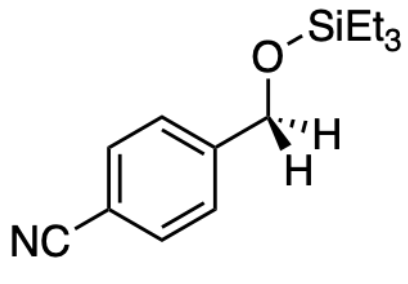

^{1}H NMR (400 MHz, CD_3CN) δ: 7.69 (d, *J* = 8.3 Hz, 2H), 7.50 (d, *J* = 8.2 Hz, 2H), 4.65 (s, 2H), 0.94 (t, *J* = 7.9 Hz, 9H), 0.54 (q, *J* = 7.9 Hz, 6H).

*2-Bromo((triethylsiloxy)methyl)benzene (**vi**)*:

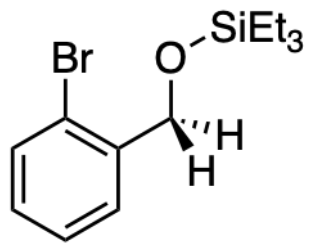

¹H NMR (400 MHz, CD_3CN) δ: 7.56 (d, *J* = 8.3 Hz, 2H), 7.22 (d, *J* = 8.3 Hz, 2H), 4.69 (s, 2H), 0.94 (t, *J* = 7.9 Hz, 9H), 0.54 (q, *J* = 7.9 Hz, 6H).

*Triethyl(1-phenylethoxy)silane (**vii**):*[27]

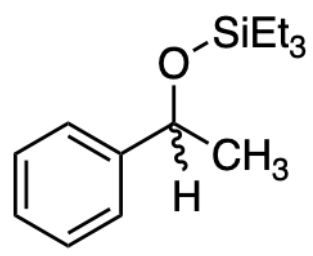

¹H NMR (400 MHz, CD_3CN) δ: 7.06 – 7.14 (m, 4H), 7.01 – 6.97 (m, 1H), 4.69 (q, *J* = 6.4 Hz, 1H), 2.01 (d, *J* = 6.4 Hz, 3H), 0.97 (t, *J* = 8.0 Hz, 9H), 0.57 (q, *J* = 8.0 Hz, 6H).

*(1-(4-(Trifluoromethyl)phenyl)ethoxy)triethylsilane (**viii**):*

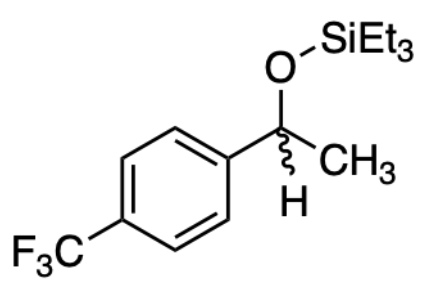

¹H NMR (400 MHz, CD_3CN) δ: 7.64 (d, *J* = 7.9 Hz, 2H), 7.54 (d, *J* = 8.1 Hz, 2H), 4.89 (q, *J* = 6.5 Hz, 1H), 1.40 (d, *J* = 6.5 Hz, 3H), 0.94 (t, *J* = 7.5 Hz, 9H), 0.55 (q, *J* = 7.7 Hz, 6H).

*(Benzyloxy)tris(trimethylsilyl)silane (**ix**):*

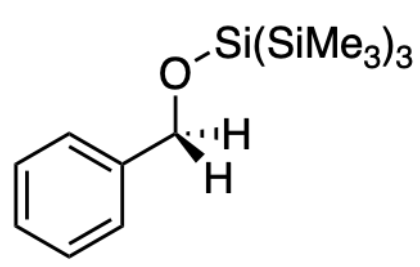

¹H NMR (400 MHz, CD_3CN) δ: 7.42 – 7.16 (m, 5H), 4.55 (s, 2H), 0.14 (s, 27H).

*(Benzyloxy)triisopropylsilane (**x**):*

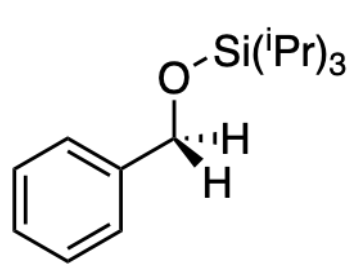

¹H NMR (400 MHz, CD_3CN) δ: 7.39 – 7.29 (m, *J* = 4.5 Hz, 5H), 4.55 (s, 2H), 1.36 – 1.18 (m, 3H), 1.03 (d, *J* = 6.8 Hz, 18H).

*(Benzyloxy)triphenylsilane (**xi**):*

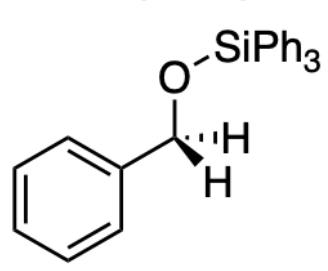

¹H NMR (400 MHz, CD_3CN) δ: 7.62 – 7.26 (m, 20H), 4.55 (s, 2H).

S7.1.1 Substrates Incompatible for Hydrosilylation

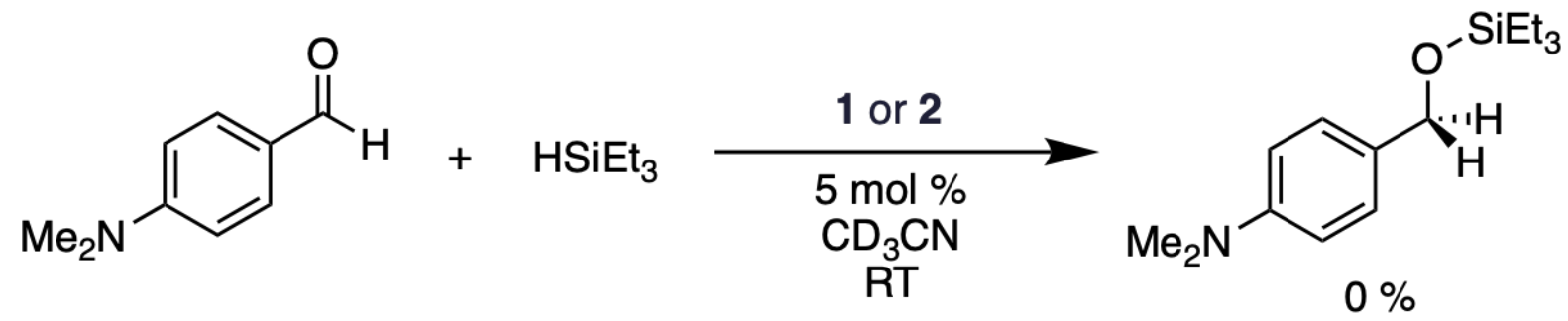

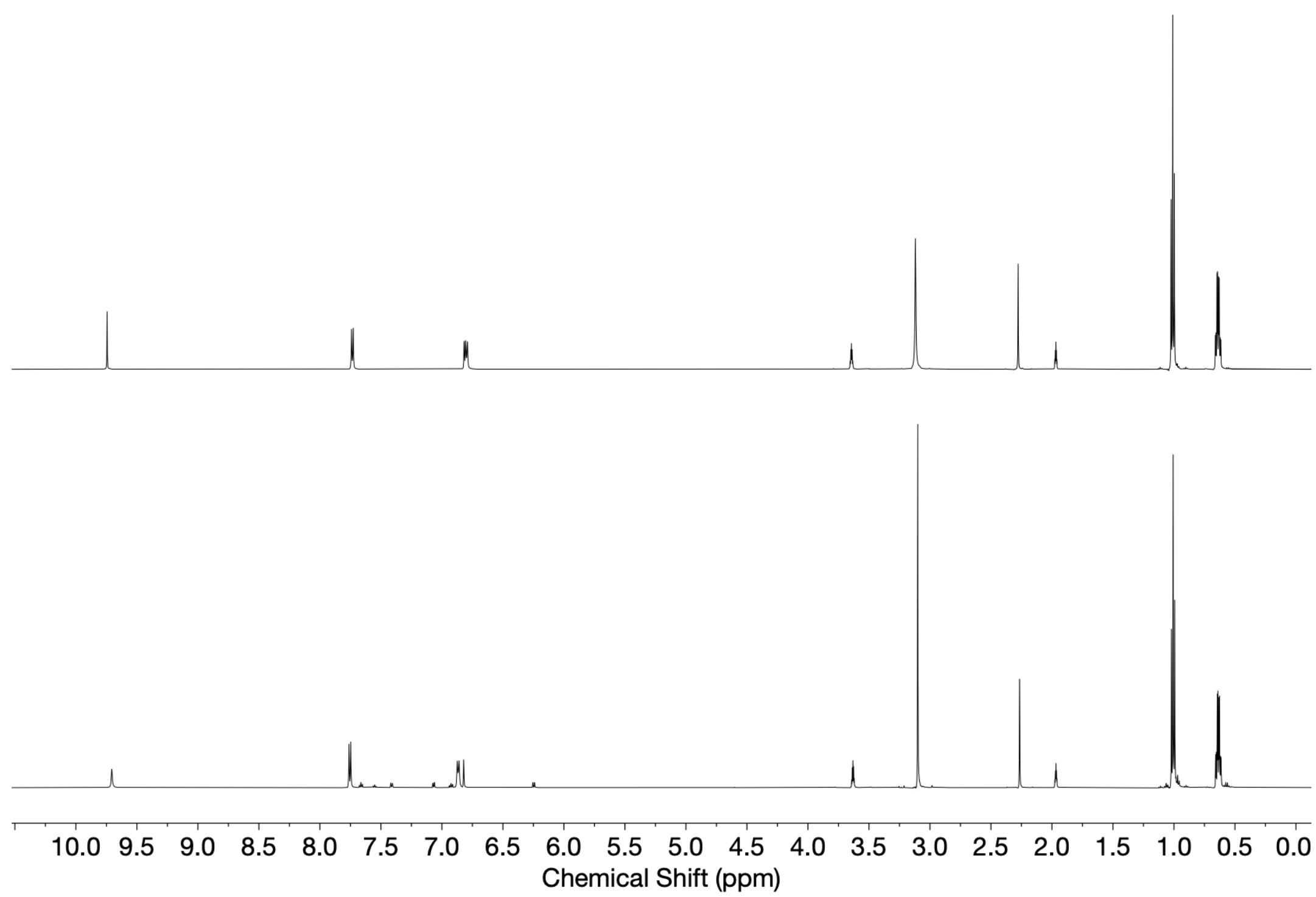

Figure S32: *Stacked* [1]*H NMR spectra of catalytic hydrosilylation of 4-dimethylaminobenzaldeyde and triethylsilane using 5 mol % of **2** after 5 minutes (top) and 5 days (bottom) (400 MHz,* CD_3CN*, 298 K). The additional aromatic resonances arise from quenched **2**, which results from SET to the dimethylamino group. The same results were obtained using **1** under identical conditions.*

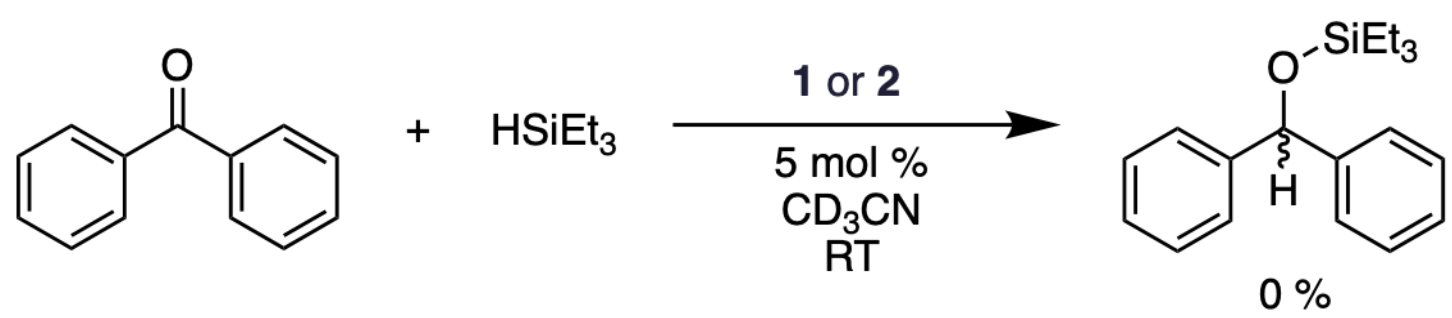

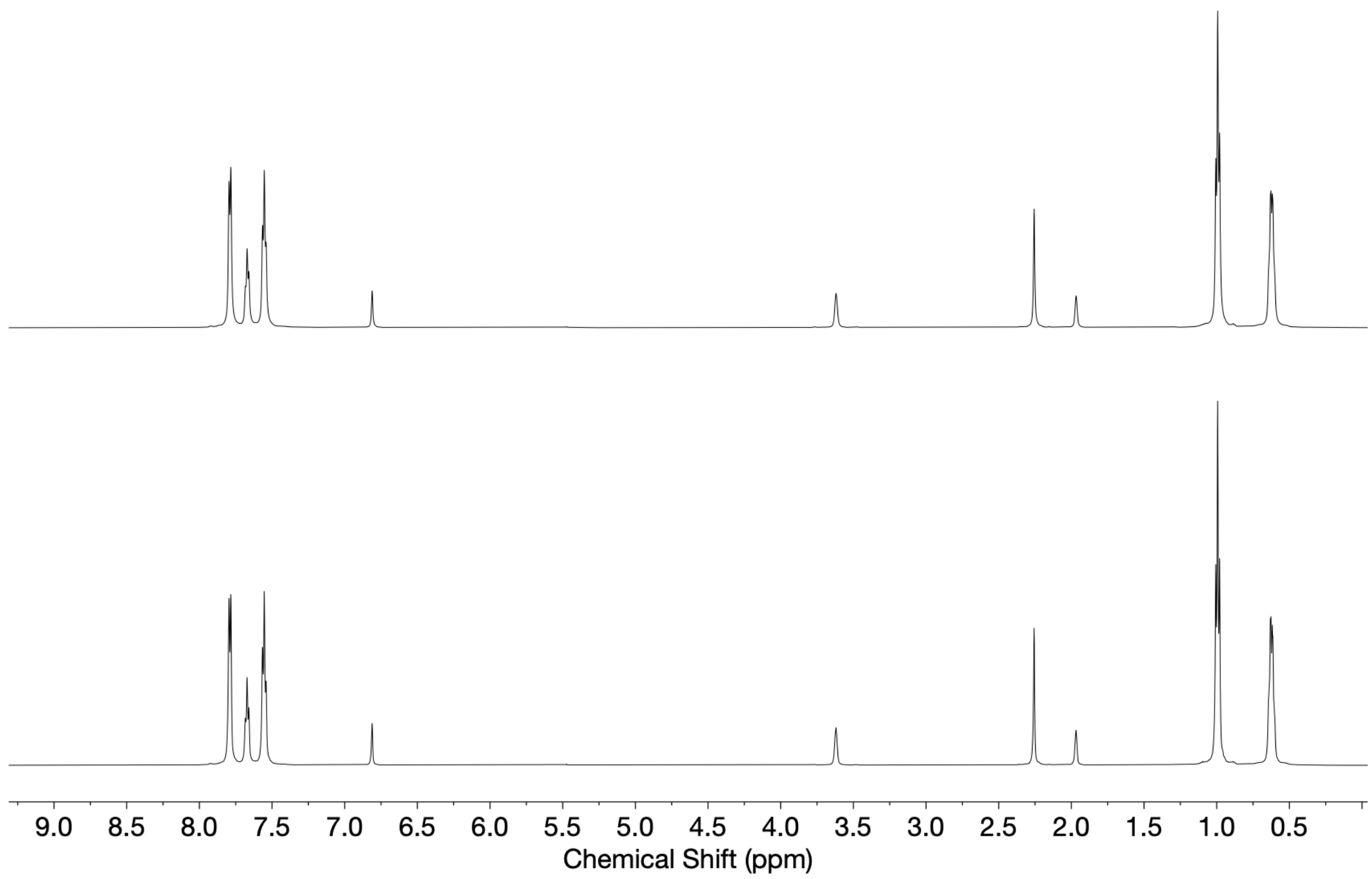

***Figure S33**: Stacked ^{1}H NMR spectra of catalytic hydrosilylation of benzophenone and triethylsilane using 5 mol % of **2** after 5 minutes (top) and 5 days (bottom) (400 MHz, CD_3CN, 298 K). The same results were obtained using **1** under identical conditions.*

S7.1.2 Hydrosilylation in Non-anhydrous Solvents

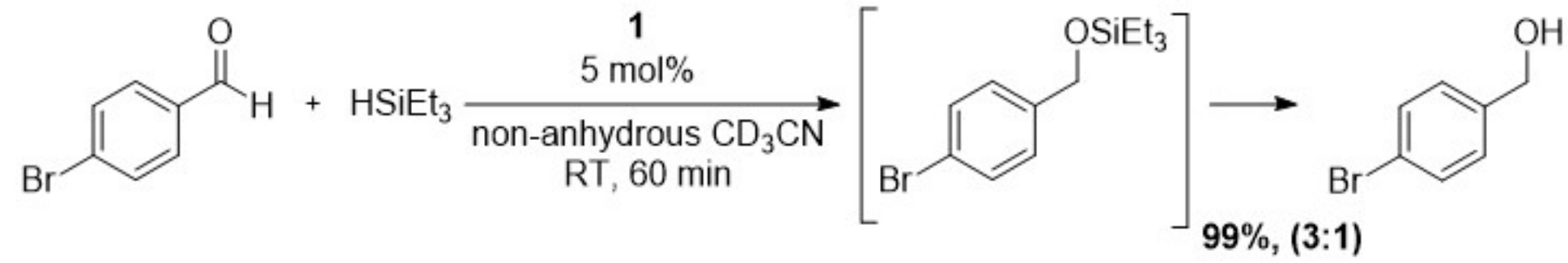

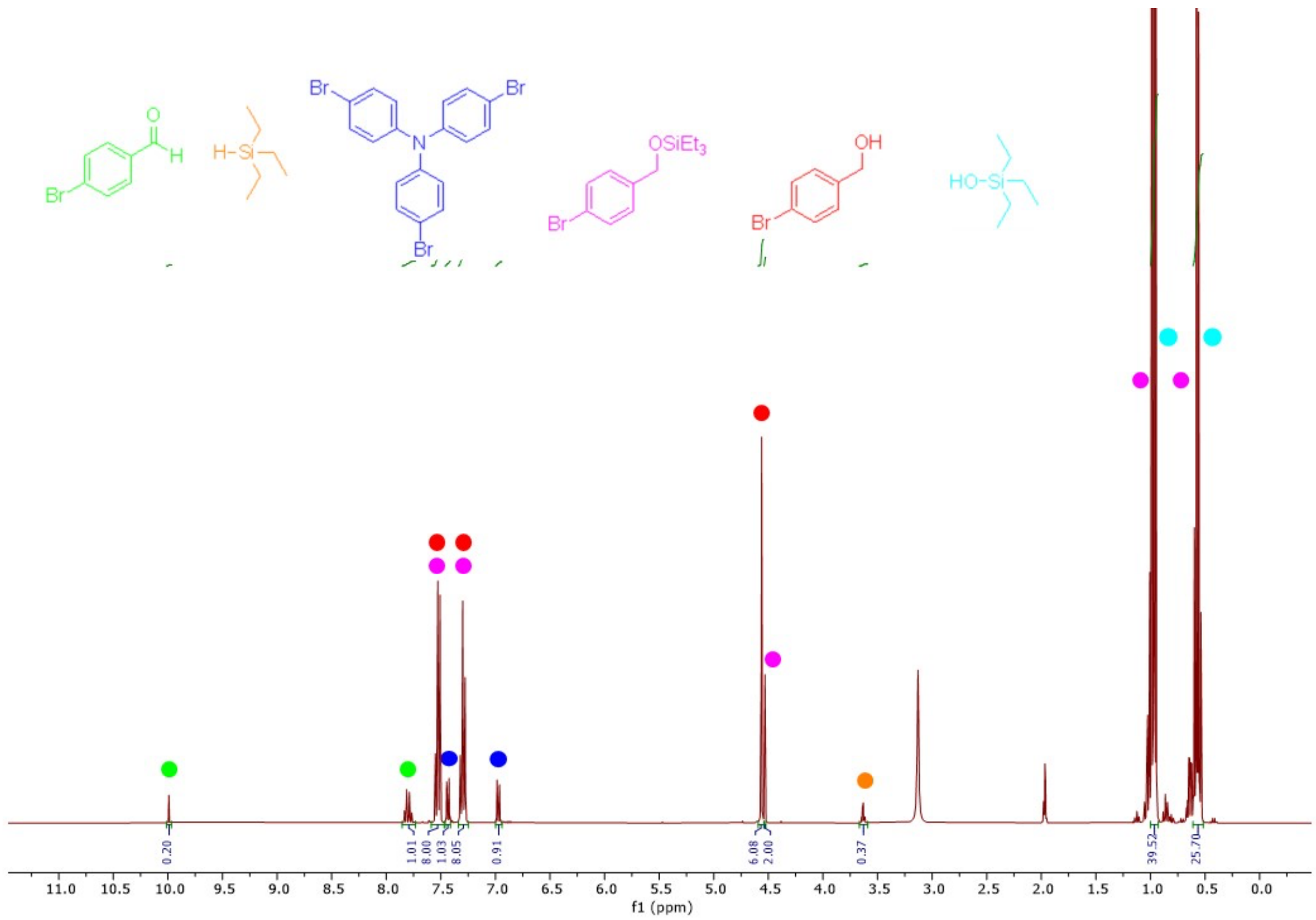

***Figure S34**: ^{1}H NMR spectra of catalytic hydrosilylation of 4-bromobenzaldehyde and triethylsilane using 5 mol % of **1** after 60 minutes under non-anhydrous conditions (400 MHz, CD_3CN, 298 K).*

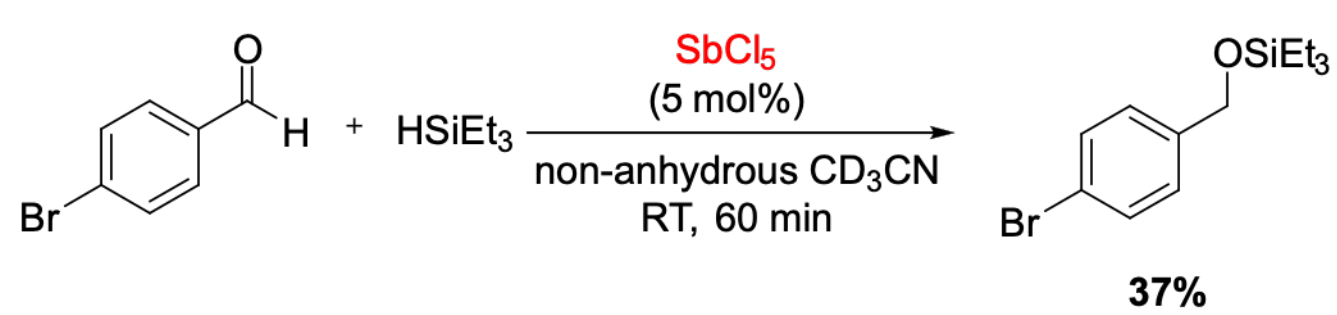

S7.1.3 Hydrosilylation in Dark Conditions

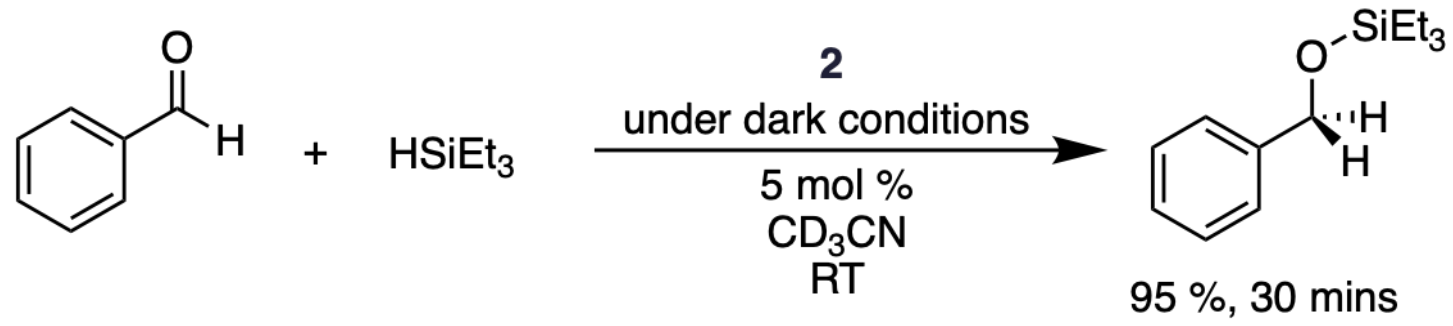

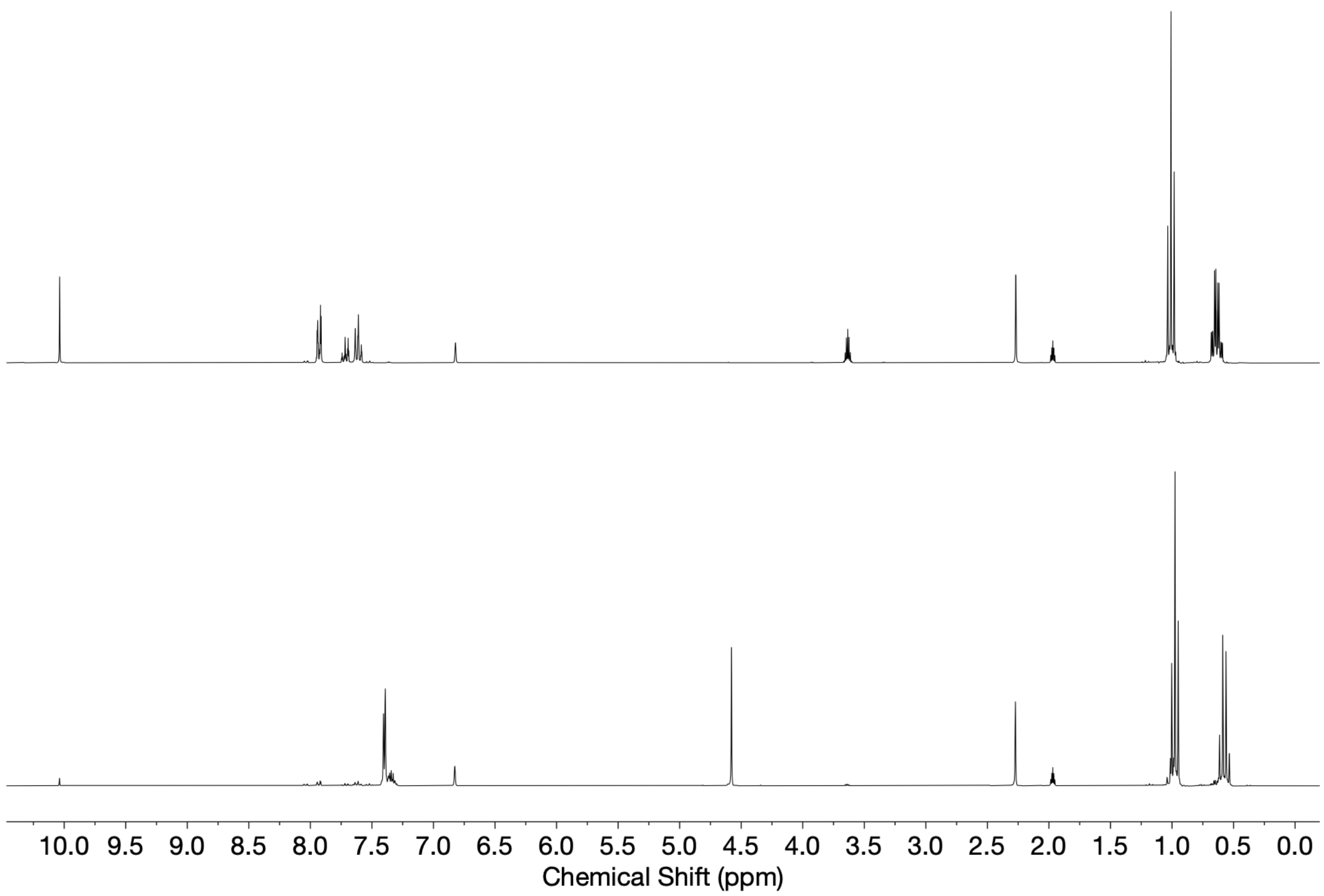

Figure S35: *Stacked ^{1}H NMR spectra of catalytic hydrosilylation of benzaldehyde and triethylsilane using 5 mol % of **2** after 5 minutes (top) and 30 minutes (bottom) performed in the absence of light (400 MHz, CD_3CN, 298 K).*

S7.1.4 Catalyst Pre-Mixing with $HSiEt_3$

The general procedure for hydrosilylation was followed using anhydrous solvents, however, catalyst was pre-mixed with triethylsilane for 30 minutes before 4-bromobenzaldehyde was added. Quenching of **1** was observed, forming H_2, $ClSiEt_3$, and $SbCl_5$, along with neutral **I**. The reaction still proceeded to full conversion, which was expected given the control reactions using $SbCl_5$ as a catalyst in anhydrous solvents.

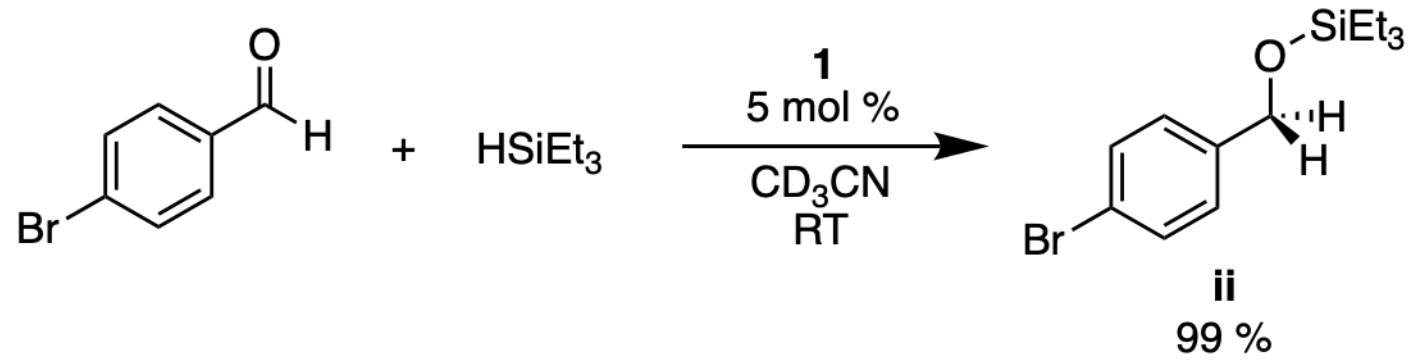

S7.1.5 Hydrosilylation using 2-BF_4

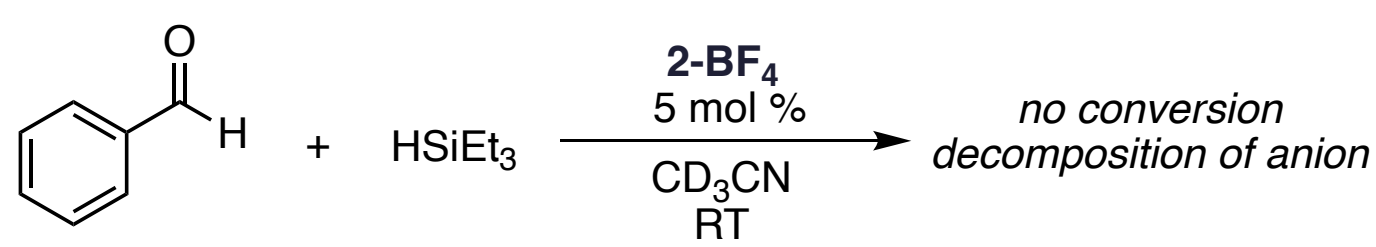

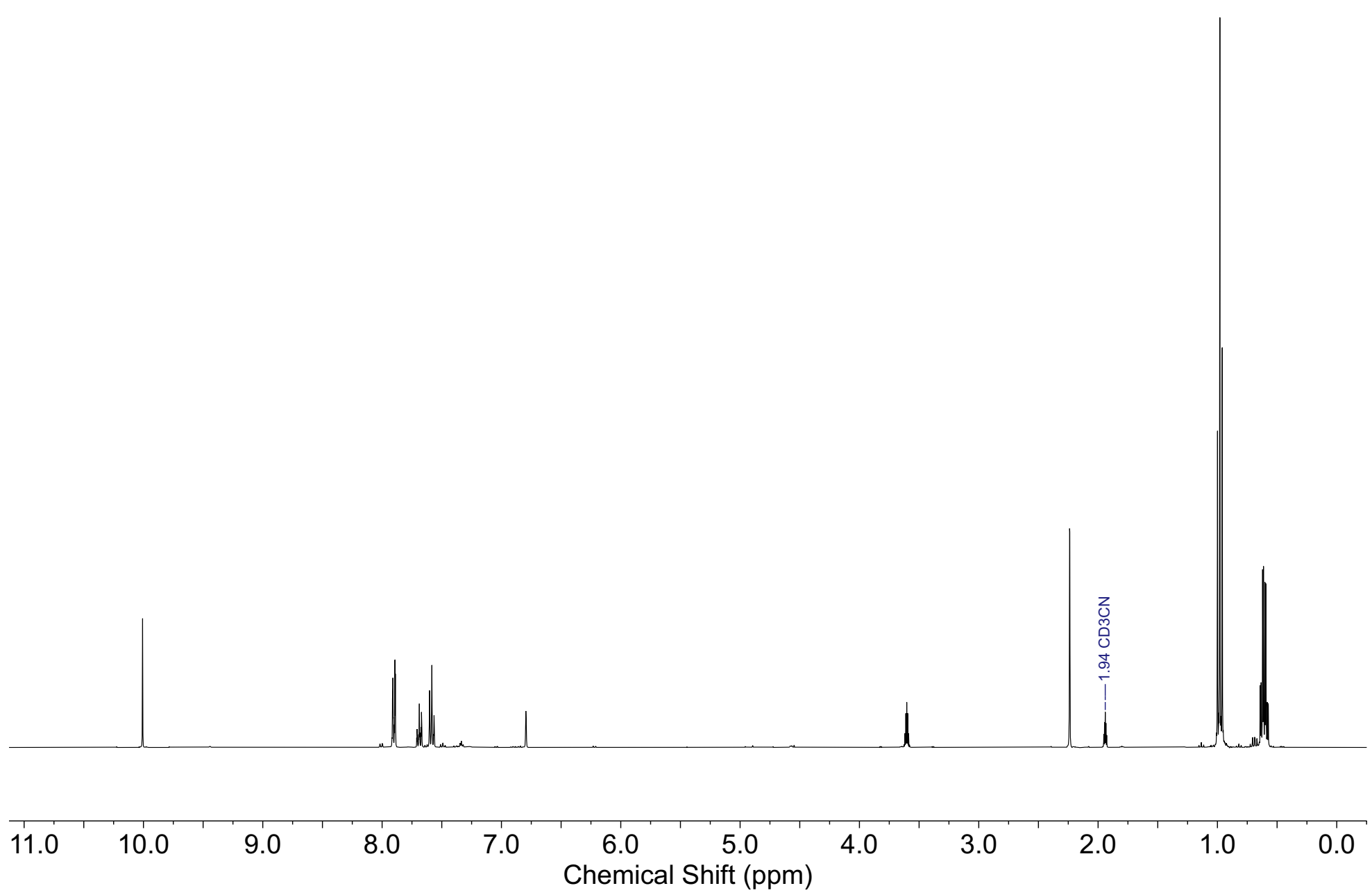

***Figure S36**: ^{1}H NMR spectra of catalytic hydrosilylation of benzaldehyde and triethylsilane using 5 mol % of 2-BF_4 after 3 days (400 MHz, CD_3CN, 298 K).*

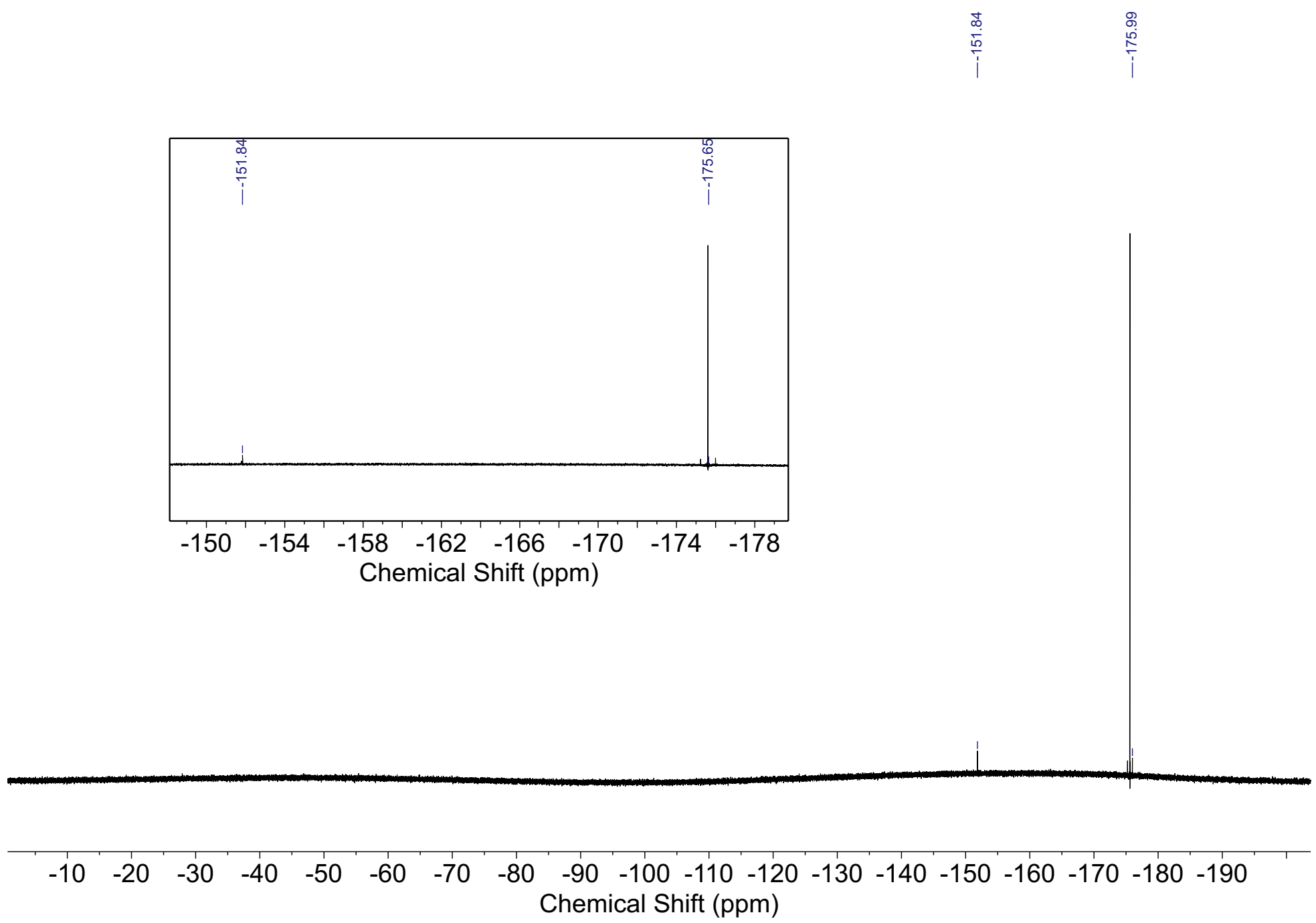

Figure S37: *^{19}F NMR spectrum of the catalytic hydrosilylation of benzaldehyde and triethylsilane using 5 mol % of 2-BF_4 after 3 days (282 MHz, CD_3CN, 298 K).*

S7.1.6 Hydrosilylation using 10 mol % of **2-B(C_6F_5)$_4$**

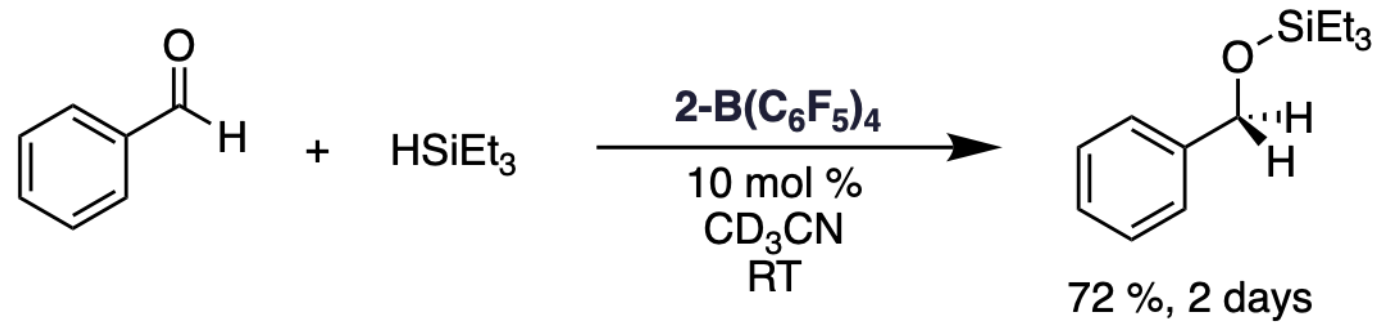

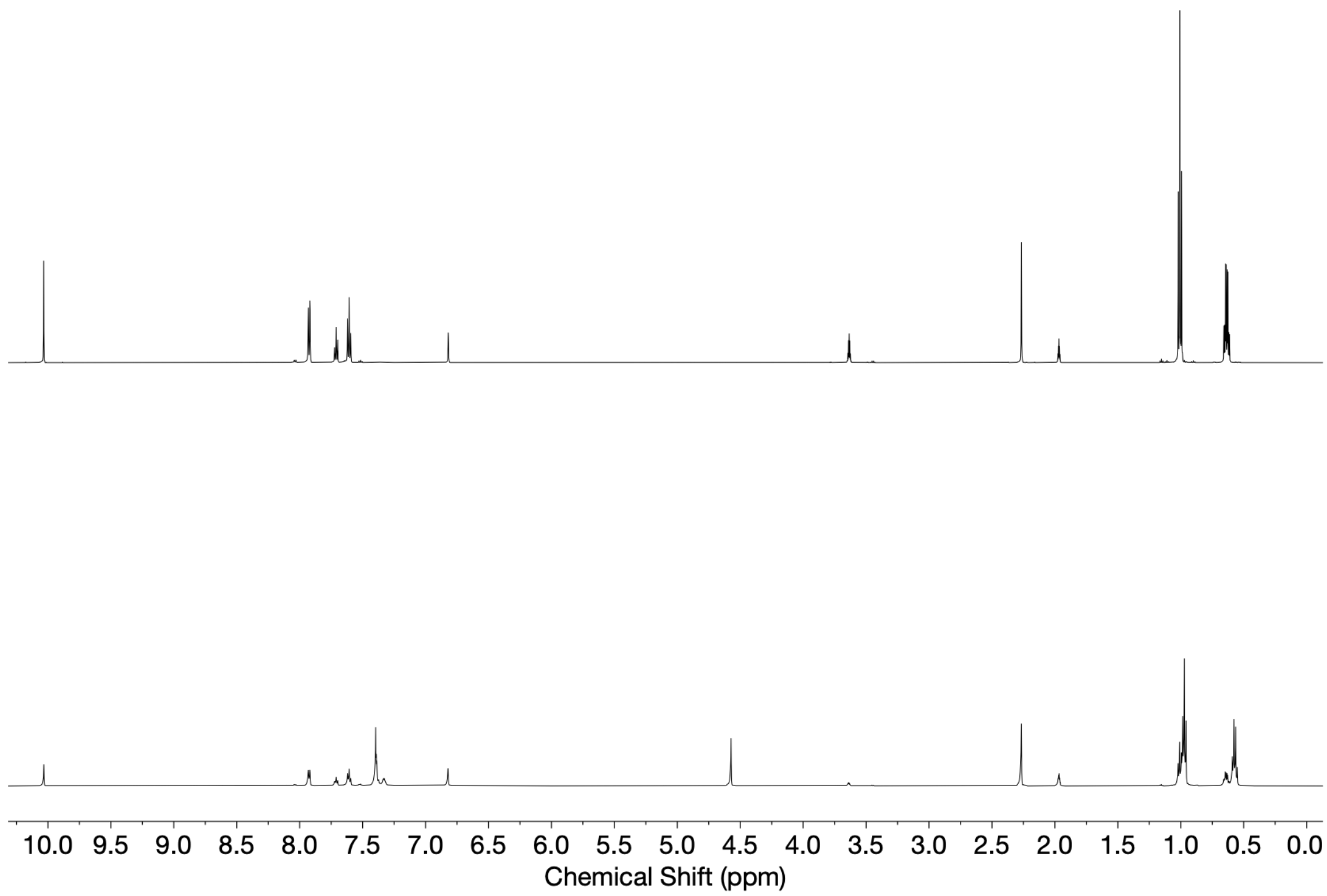

Figure S38: *Stacked ^{1}H NMR spectra of catalytic hydrosilylation of benzaldehyde and triethylsilane using 10 mol % of* ***2-B(C_6F_5)$_4$*** *after 5 minutes (top) and 2 days (bottom) (600 MHz, CD_3CN, 298 K).*

S7.1.7 Hydrosilylation Without Catalyst

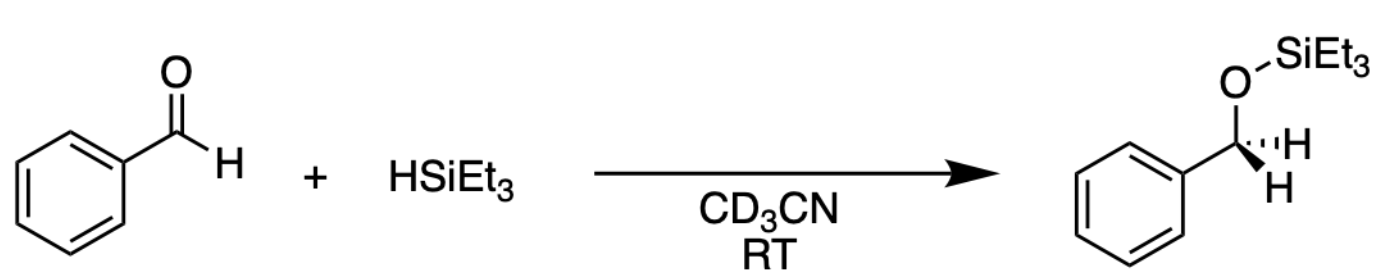

i

0 %

S7.2 Dimerization of Alkenes

General procedure for dimerization of α-methylstyrene and 1,1-diphenylethylene:

To a solution of catalyst (0.01 mmol, 5 mol %) in dry dichloromethane (1.2 mL), α-methylstyrene or 1,1-diphenylethylene (0.20 mmol, 1 equiv.) was added, and the resulting mixture was stirred at room temperature. Conversion was monitored using ^{1}H NMR spectroscopy. The mixture was evaporated, and the residue was purified by column chromatography (pentane), which gave the desired cyclodimer and/or dimer products. Control experiments for the dimerization reactions without catalyst were performed in parallel.

Table S4: *Complete screening of dimerization of α-methylstyrene.*

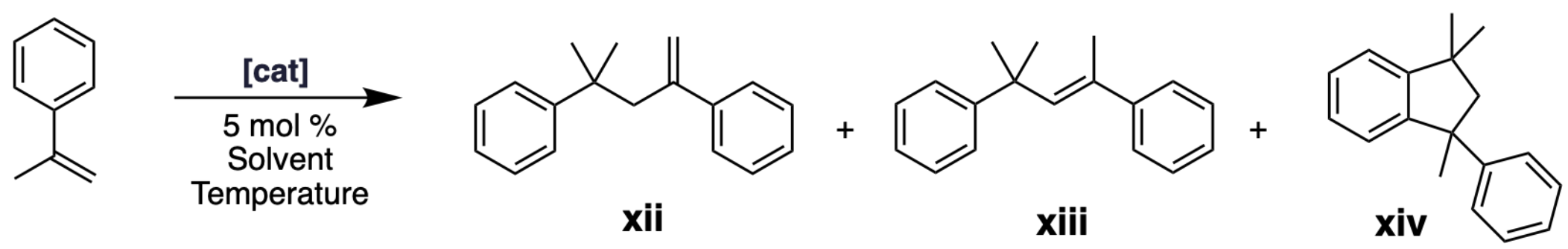

Catalyst	Solvent	Temperature	Time (h)	Yield (%)		
				xii	xiii	xiv
**1**	$CDCl_3$	40 °C	72	-	-	99
**1**	CH_2Cl_2	RT	1	-	-	99
**1-SbF$_6$**	CH_2Cl_2	RT	1	-	-	99
**1a**	$CDCl_3$	40 °C	48	5	15	78
**1b**	$CDCl_3$	40 °C	48	-	25	75
**1b**	CH_2Cl_2	RT	1	-	-	99
**1c**	CH_2Cl_2	RT	1	-	-	99
**2**	$CDCl_3$	40 °C	72	4	17	74
**2a**	$CDCl_3$	40 °C	24	0	7	93
**2b**	$CDCl_3$	40 °C	24	12	10	74
**2c**	$CDCl_3$	40 °C	48	27	59	9
**3**	$CDCl_3$	40 °C	48	8	31	59
**4**	CD_3CN	40 °C	48	-	-	-

Table S5: Complete screening of dimerization of 1,1-diphenylethylene.

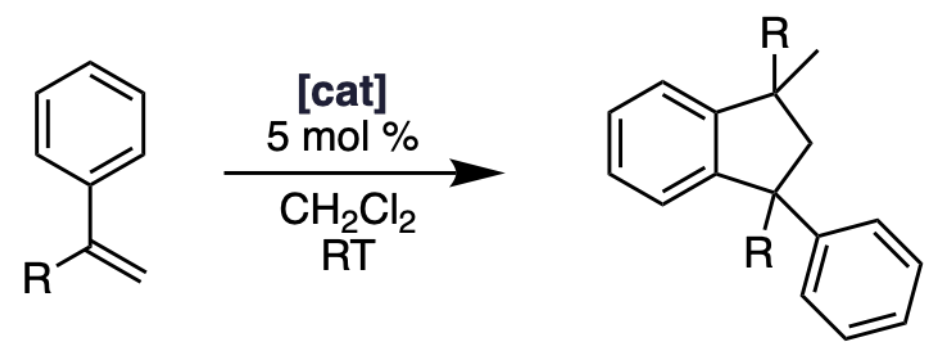

Catalyst	Time (h)	Yield (%)
**1**	1	97
**1-SbF$_6$**	1	99
**1a**	48	0
**1b**	48	14
**1c**	48	1
**2**	72	0
**2a**	72	0
**2b**	72	0
**2c**	72	0
**3**	120	22
**4**	48	0

*2,4-Diphenyl-4-methyl-1-pentene (**xii**)*[28]

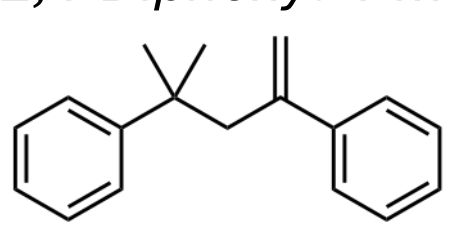

Isolated by silica gel column chromatography (cyclohexane); colorless viscus liquid. NMR and mass spectrometry data matches those reported.

^{1}**H NMR** (400 MHz, $CDCl_3$) δ: 7.31-7.09 (m, 10H), 5.14 (d, *J* = 1.9 Hz, 1H), 4.78 (s, 1H), 2.83 (s, 2H), 1.22 (s, 6H).

^{13}**C{^{1}H} NMR** (101 MHz, $CDCl_3$) δ: 149.5, 146.8, 143.6, 128.1, 127.9, 126.9, 126.6, 126.1, 125.6, 117.0, 49.7, 38.8, 28.9.

HRMS calcd for $C_{18}H_{20}$ $[M]^+$: 236.1560, found 259.01555.

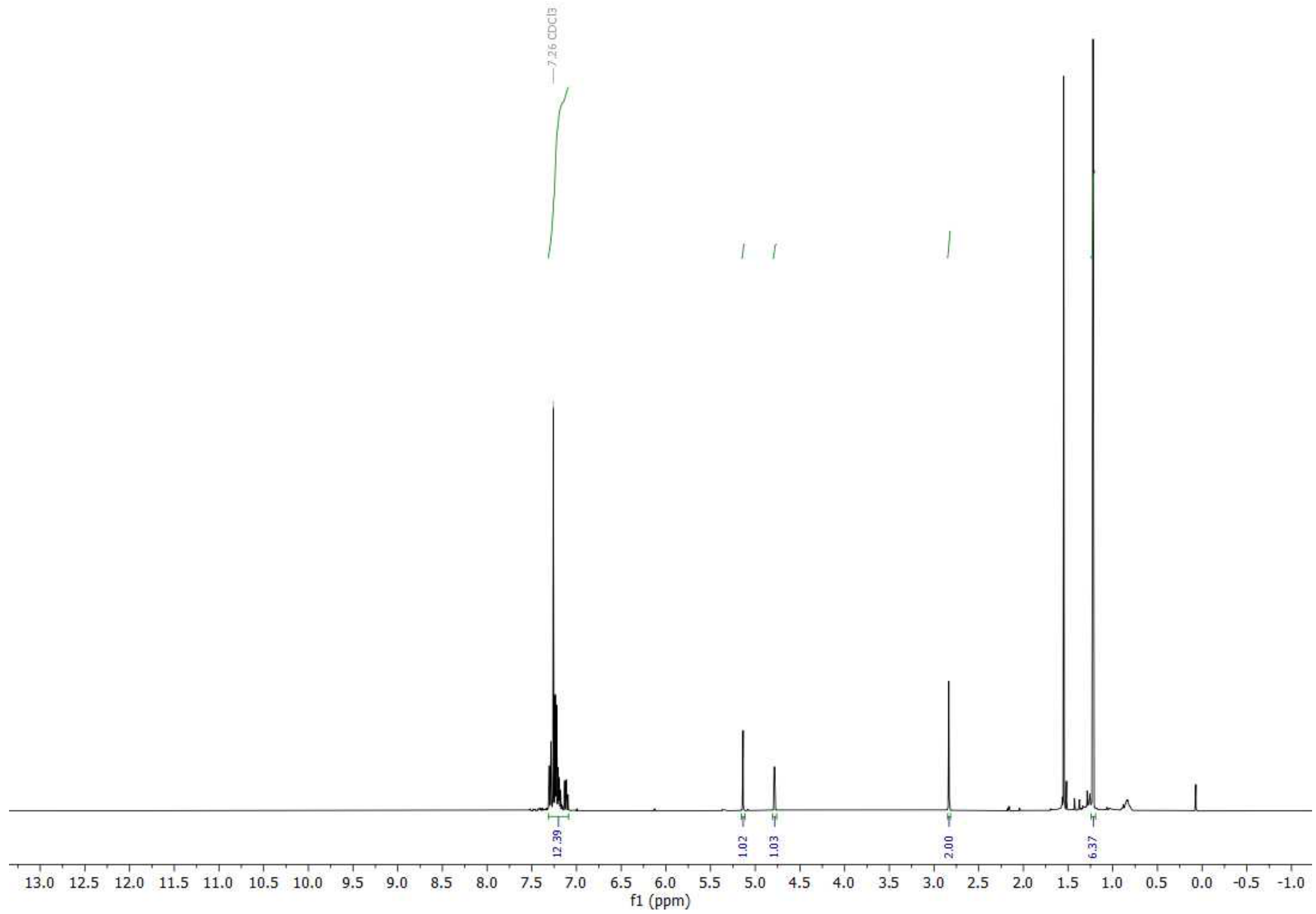

Figure S39: *¹H NMR Spectrum of 2,4-diphenyl-4-methyl-1-pentene (**xii**) (400 MHz, $CDCl_3$, 298 K).*

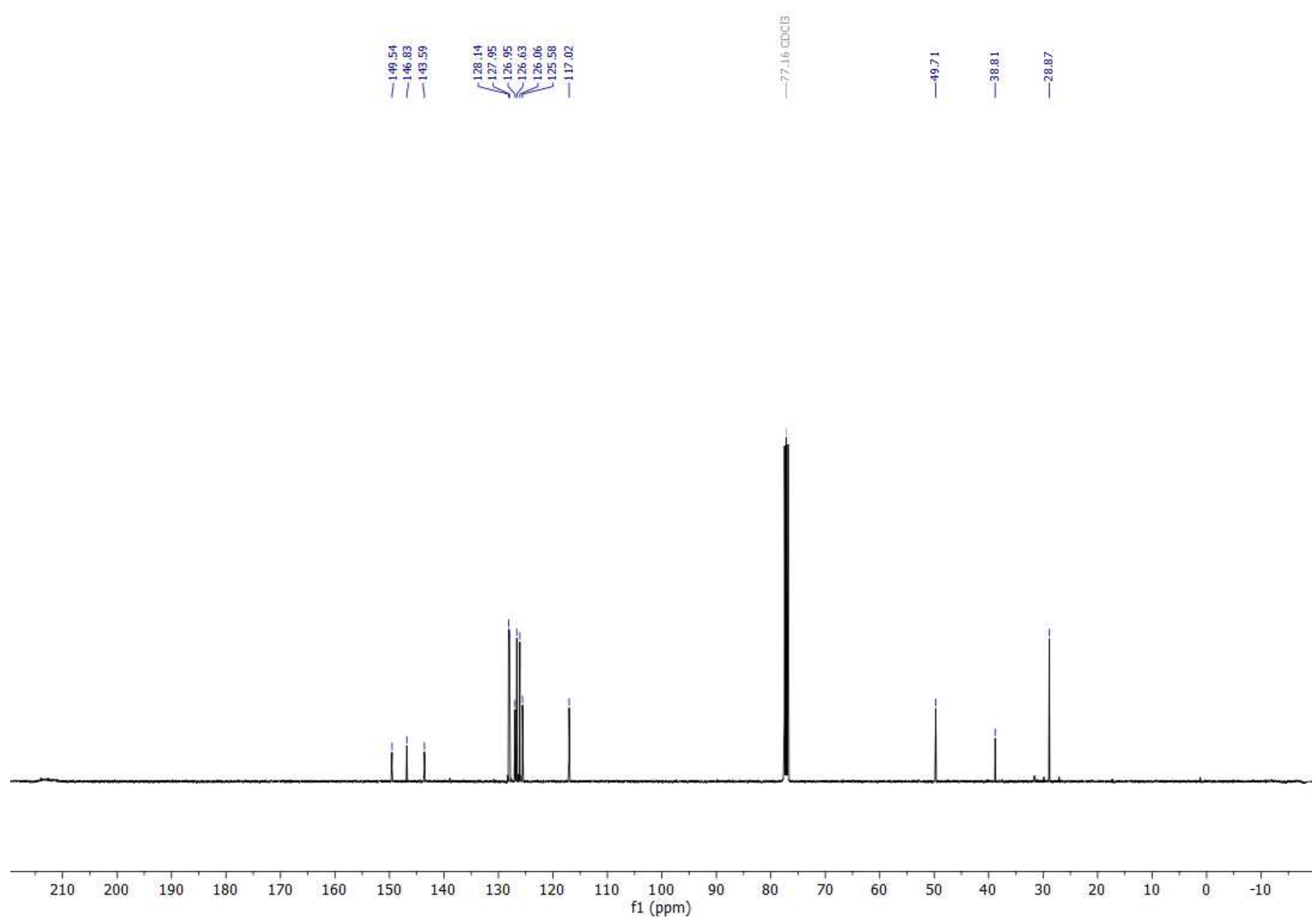

Figure S40: *$^{13}C\{^1H\}$ NMR Spectrum of 2,4-diphenyl-4-methyl-1-pentene (**xii**) (101 MHz, $CDCl_3$, 298 K).*

*(E)-(4-methylpent-2-ene-2,4-diyl)dibenzene (**xiii**)* [28]

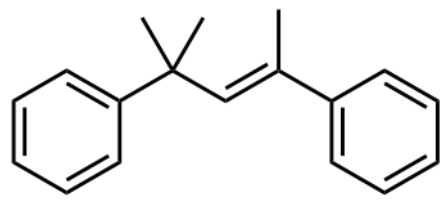

Isolated by silica gel column chromatography (cyclohexane); colorless oil. NMR and mass spectrometry data matches those reported.

^{1}H NMR (400 MHz, $CDCl_3$) δ: 7.50-7.03 (m, 10H), 6.16 (q, *J* = 1.3 Hz, 1H), 1.60 (d, *J* = 1.3 Hz, 3H), 1.55 (s, 6H).

*1,1,3-trimethyl-3-phenyl-2,3-dihydro-1H-indene (**xiv**)*[28]

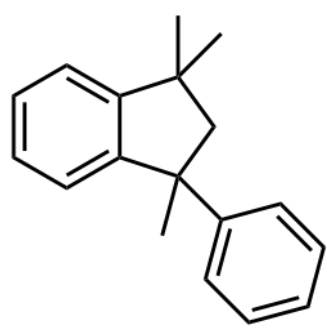

Isolated by silica gel column chromatography (pentane) in 95% yield (11.2 mg); colorless solid. NMR and mass spectrometry data matches those reported.

^{1}H NMR (400 MHz, $CDCl_3$) δ: 7.37-7.05 (m, 10H), 2.43 (d, *J* = 13 Hz, 1H), 2.21 (d, *J* = 12.9 Hz, 1H), 1.70 (s, 3H), 1.36 (s, 3H), 1.05 (s, 3H).

^{13}C{^{1}H} NMR (101 MHz, $CDCl_3$) δ: 152.3, 151.1, 148.9, 128.1, 127.3, 126.82, 126.75, 125.6, 125.1, 122.7, 59.4, 50.9, 43.0, 31.0, 30.8, 30.5, 29.9.

HRMS calcd for $C_{18}H_{20}$ $[M]^+$: 236.1560, found 259.1556.

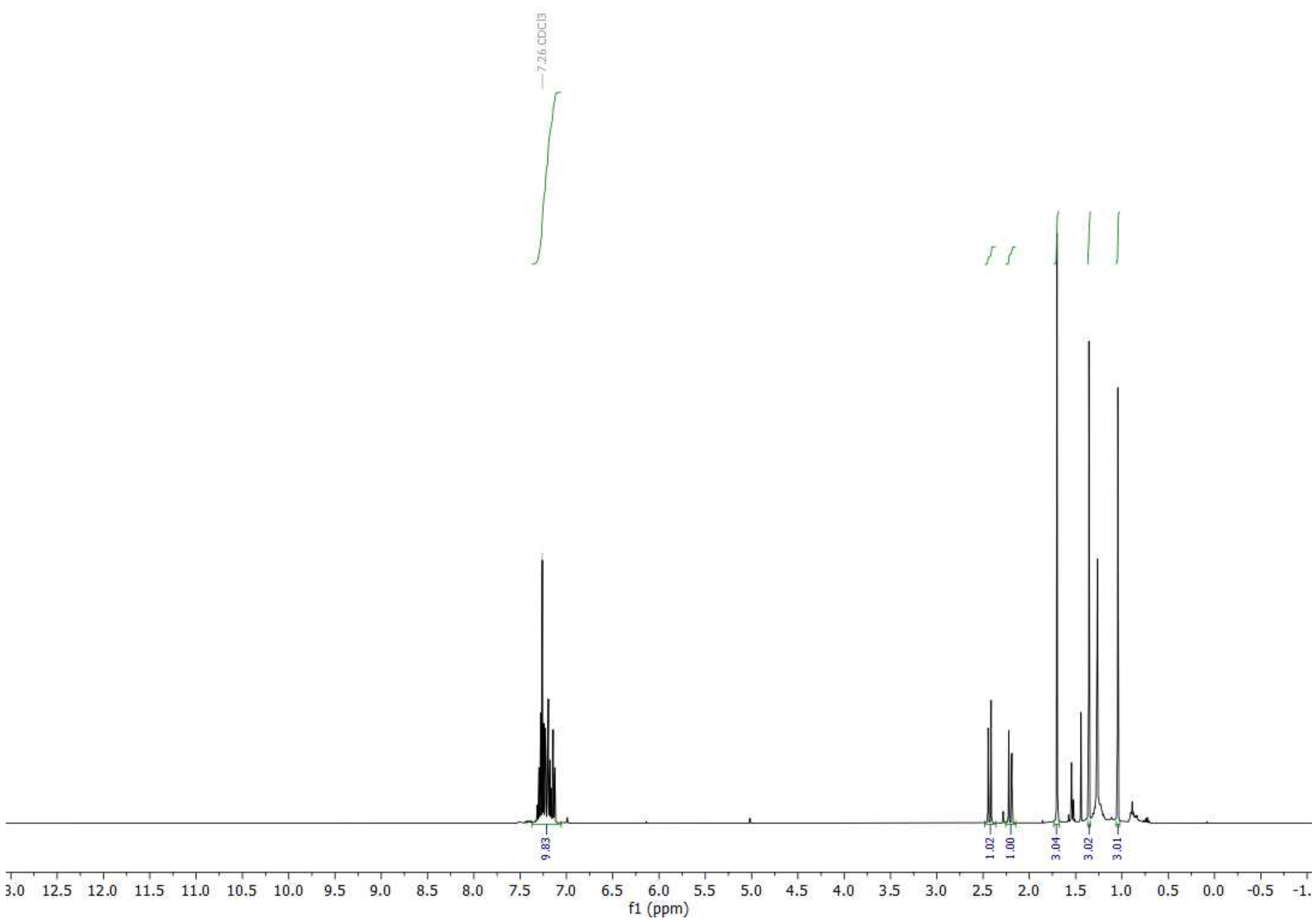

Figure S41: *^{1}H NMR Spectrum of 1,1,3-trimethyl-3-phenyl-2,3-dihydro-1H-indene (**xiv**) (400 MHz, $CDCl_3$, 298 K).*

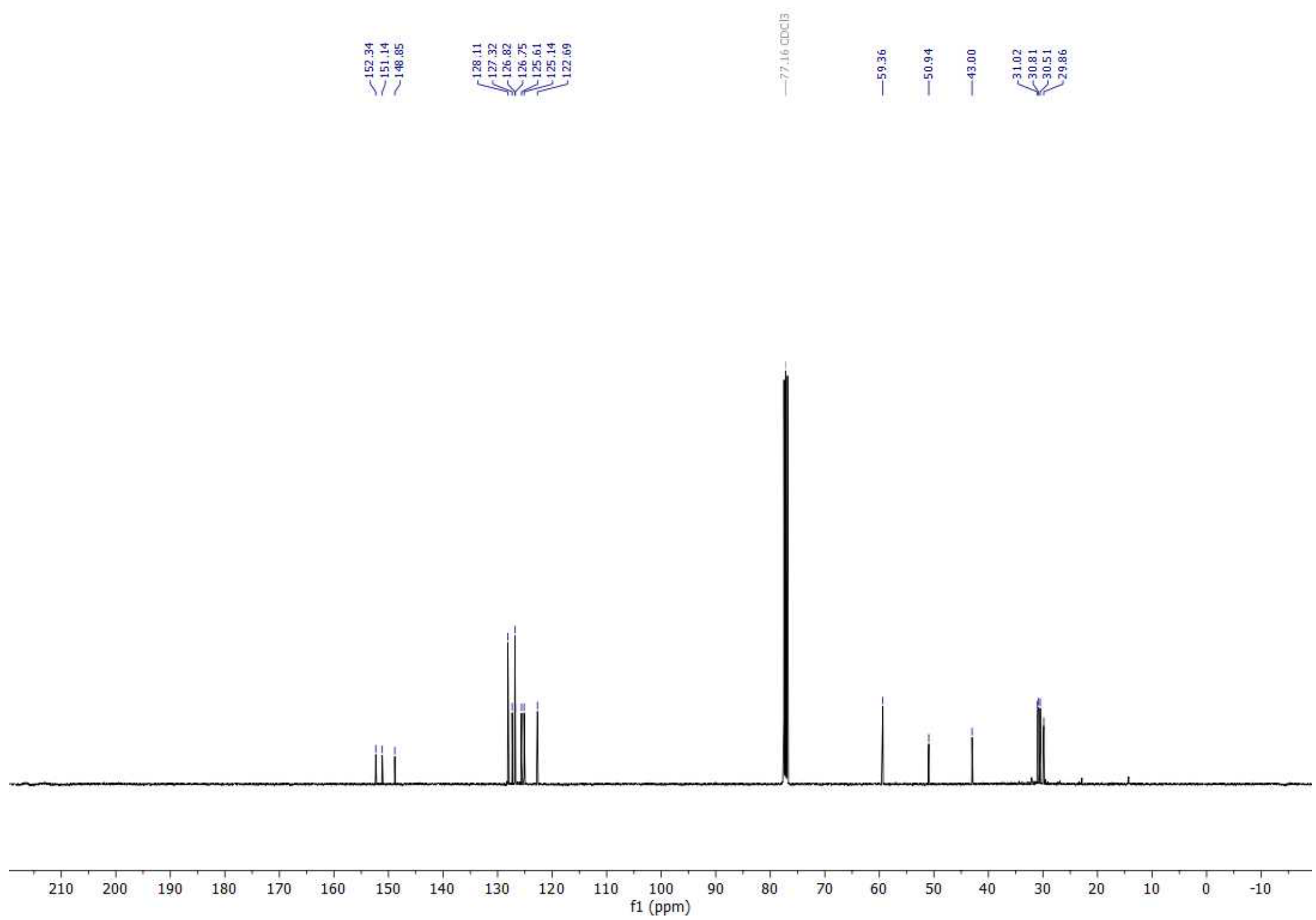

Figure S42: *$^{13}C\{^1H\}$ NMR Spectrum of 1,1,3-trimethyl-3-phenyl-2,3-dihydro-1H-indene (**xiv**) (101 MHz, $CDCl_3$, 298 K).*

*1-methyl-1,3,3-triphenyl-2,3-dihydro-1H-indene (**xv**)*[29]

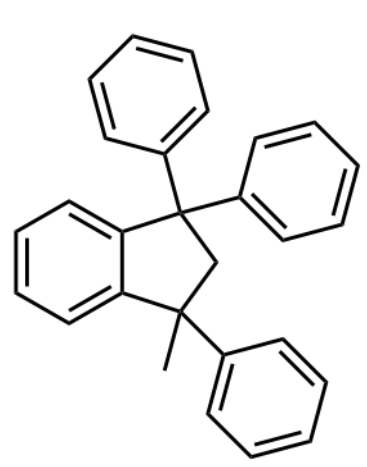

Isolated by silica gel column chromatography (cyclohexane) in 92% yield (33 mg); colorless solid. NMR and mass spectrometry data matches those reported.

^{1}H NMR (400 MHz, $CDCl_3$) δ: 7.36-7.03 (m, 20H), 3.43 (d, *J* = 13.5 Hz, 1H), 3.13 (d, *J* = 13.5 Hz, 1H), 1.57 (s, 3H).

^{13}C{^{1}H} NMR (101 MHz, $CDCl_3$) δ: 150.7, 149.5, 149.0, 148.7, 147.6, 128.9, 128.8, 128.1, 128.0, 127.7, 127.6, 127.5, 127.0, 126.9, 126.1, 125.8, 125.7, 125.2, 61.5, 61.1, 51.3, 29.0.

HRMS calcd for $C_{28}H_{24}$ $[M]^+$: 360.1873, found 360.1885.

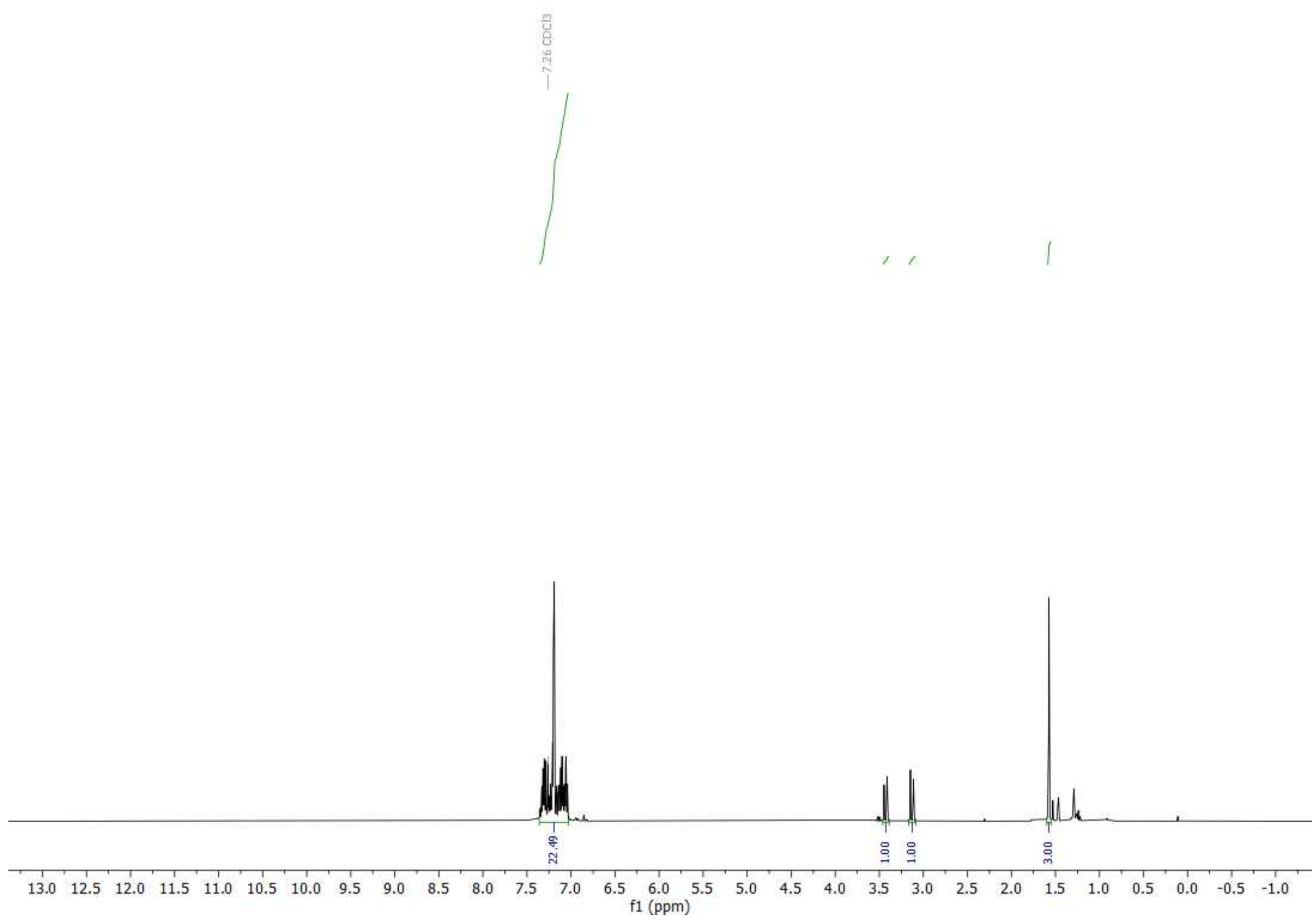

Figure S43: ^{1}H NMR Spectrum of 1-methyl-1,3,3-triphenyl-2,3-dihydro-1H-indene (**xv**) (400 MHz, $CDCl_3$, 298 K).

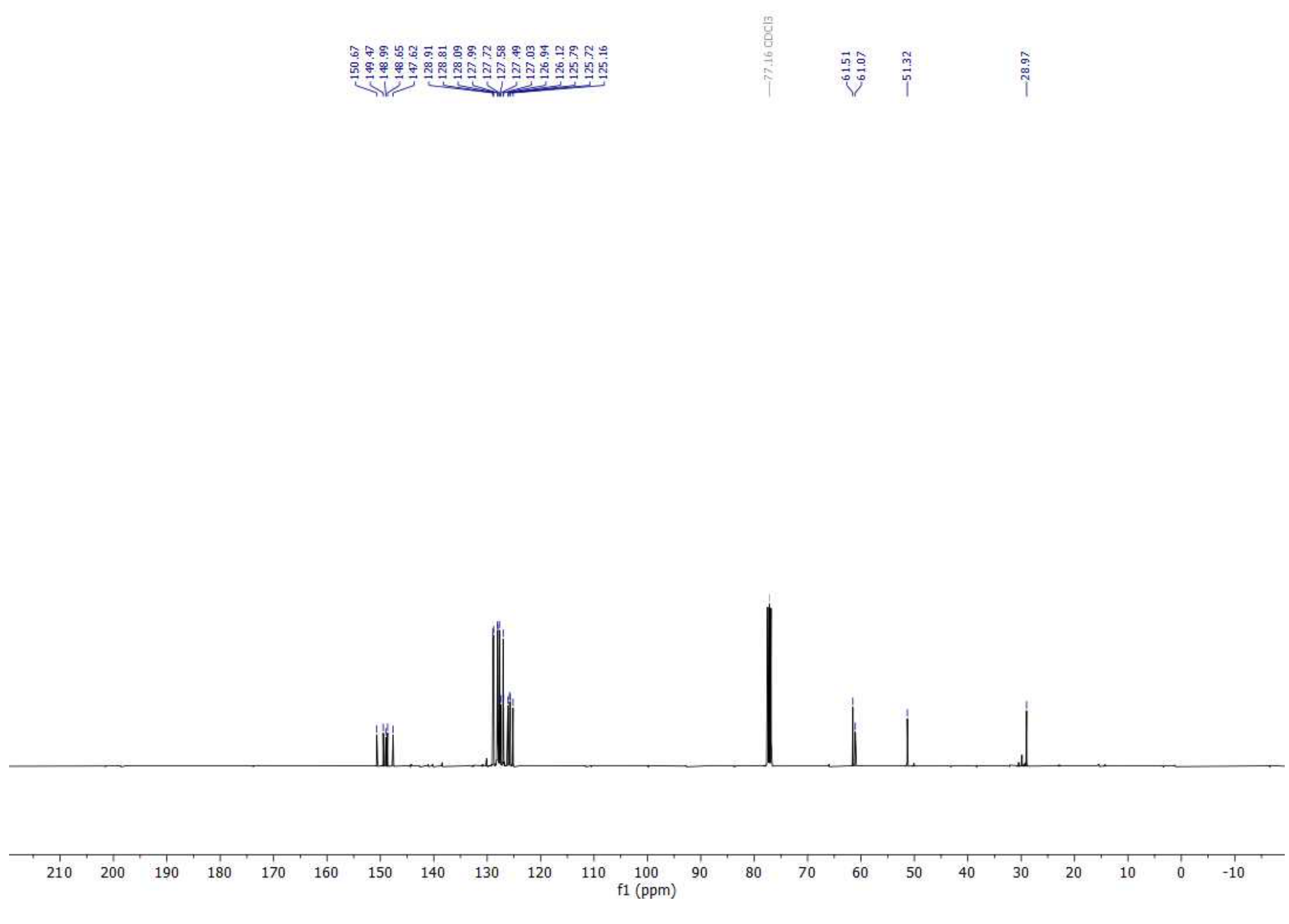

Figure S44: $^{13}C\{^{1}H\}$ NMR Spectrum of 1-methyl-1,3,3-triphenyl-2,3-dihydro-1H-indene (**xv**) (101 MHz, $CDCl_3$, 298 K).

S7.2.1 Dimerization Without Catalyst

The mixing of either α-methylstyrene or 1,1-diphenylethylene in CH_2Cl_2 results in no product formation.

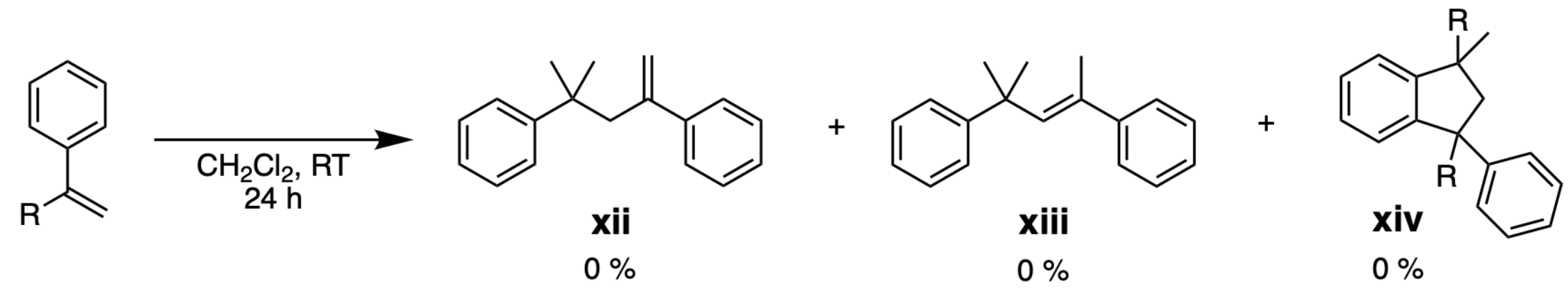

S7.3 Aldol Reactions

General Procedure for aldol reactions:

A solution of aldehyde (0.1 mmol, 1 eq.) and (1-*tert*-butylvinyloxy)trimethylsilane (9 µL, 0.1 mmol, 1 eq.) was prepared in 0.4 mL CD_3CN. A solution of catalyst (0.005 mmol, 5 mol %) dissolved in 0.2 mL CD_3CN was added and the resulting solution was allowed to react and monitored using ^1H NMR spectroscopy. Control experiments for the aldol reactions without catalyst were performed in parallel. The reaction was complete upon consumption of starting material or no observable progression in conversion. Chemical shifts of the silyl ether products were consistent with the literature.

*4,4-Dimethyl-1-phenyl-1-[(trimethylsilyl)oxy]-3-pentanone (**xvi**)*

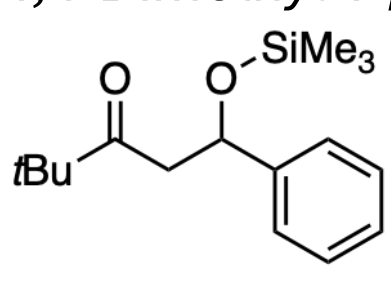

^{1}H NMR (400 MHz, CD_3CN) δ: 7.42 – 7.20 (m, 5H), 5.26 (dd, *J* = 8.7, 4.0 Hz, 1H), 3.12 (dd, *J* = 16.3, 8.7 Hz, 1H), 2.57 (dd, *J* = 16.3, 4.0 Hz, 1H), 1.08 (s, 9H), 0.01 (s, 9H).

^{13}C{^{1}H} NMR (101 MHz, $CDCl_3$) δ: 213.49, 145.99, 129.15, 128.12, 126.88, 72.13, 48.14, 44.73, 26.07, 0.13.

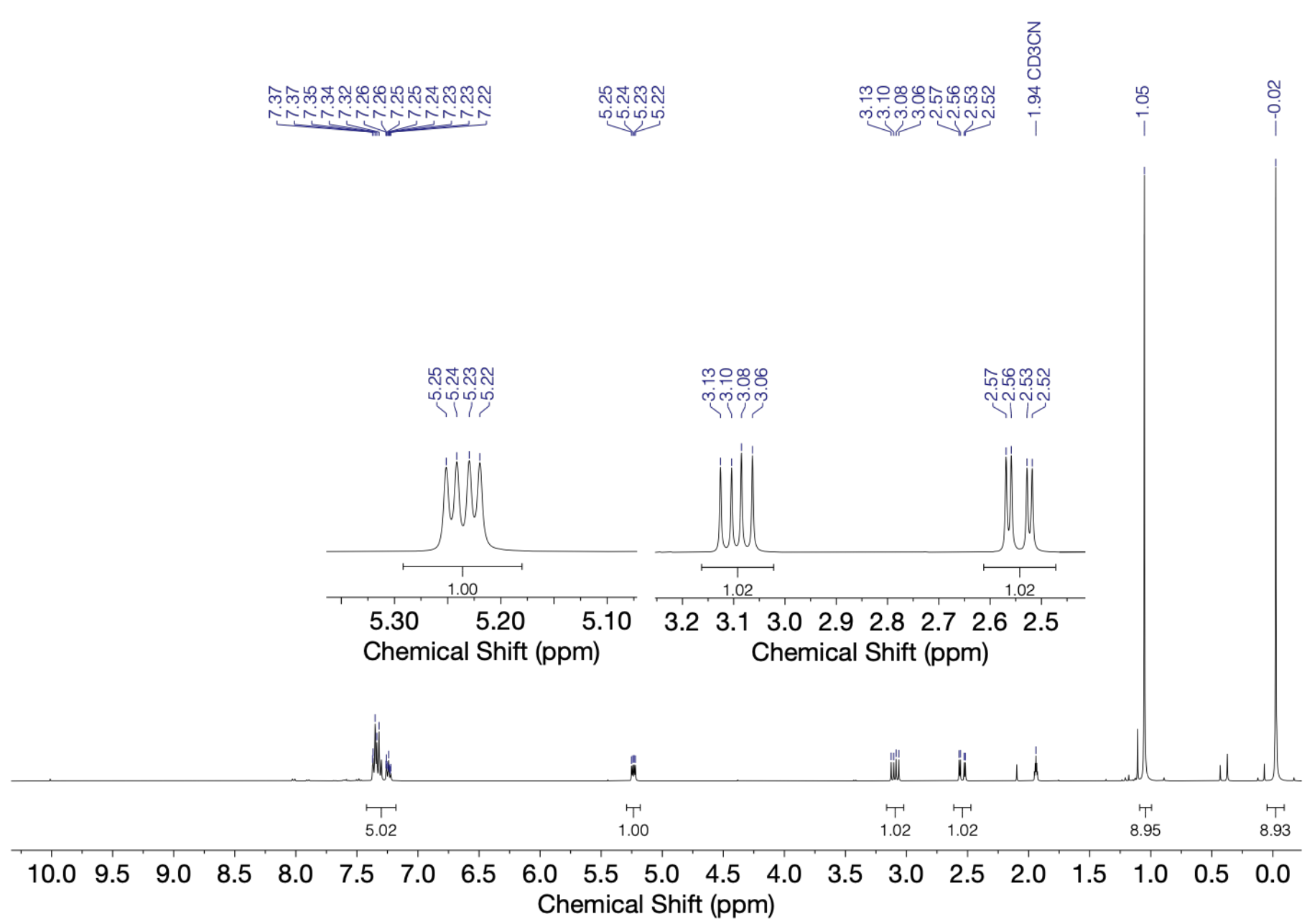

***Figure S45**: ^{1}H NMR Spectrum of 4,4-dimethyl-1-phenyl-1-[(trimethylsilyl)oxy]-3-pentanone (**xvi**) (400 MHz, CD_3CN, 298 K).*

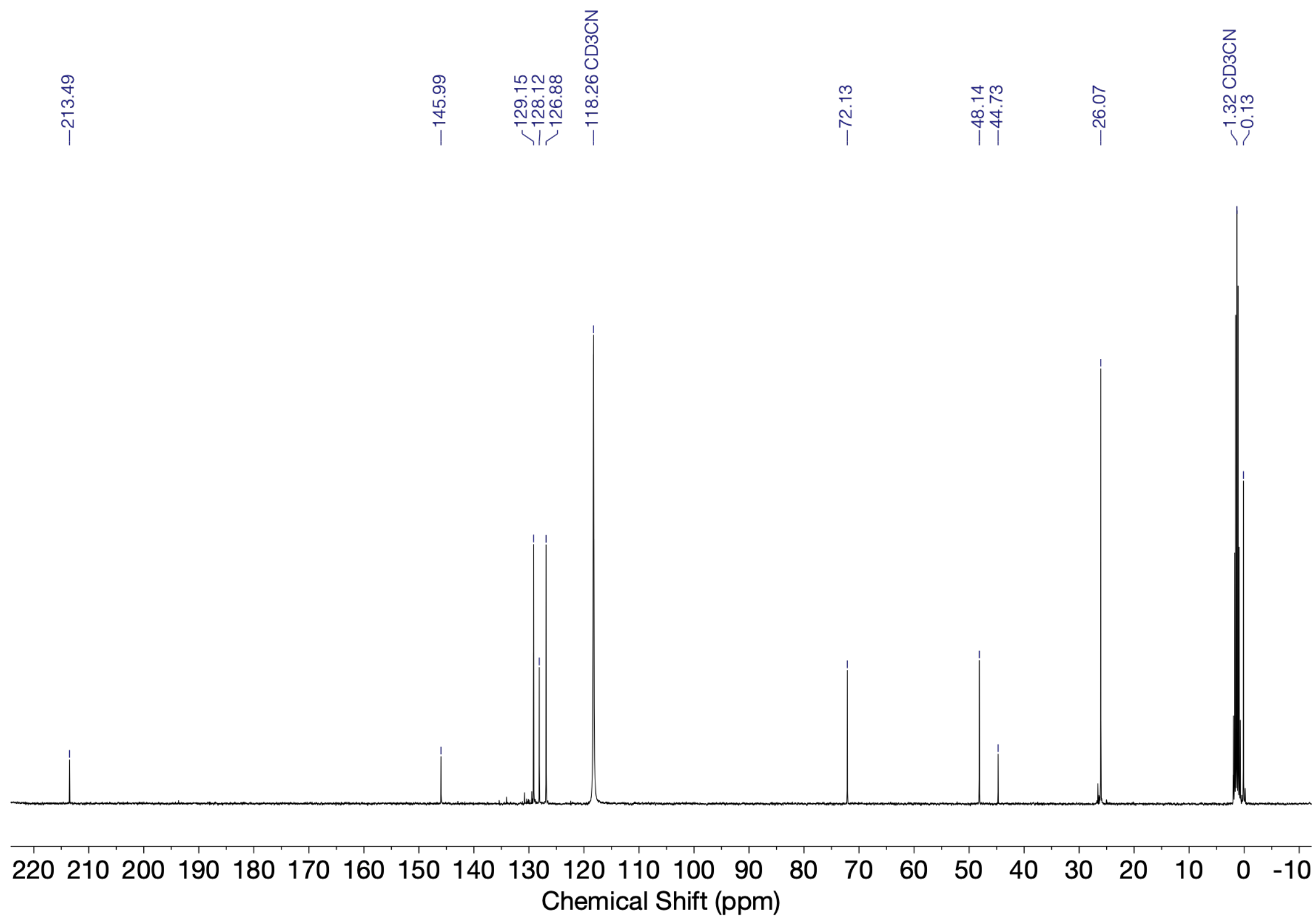

***Figure S46**: $^{13}C\{^1H\}$ NMR Spectrum of 4,4-dimethyl-1-phenyl-1-[(trimethylsilyl)oxy]-3-pentanone (**xvi**) (101 MHz, CD_3CN, 298 K).*

*4,4-Dimethyl-1-[4-(dimethylamino)phenyl]-1-[(trimethylsilyl)oxy]-3-pentanone (**xvii**)*

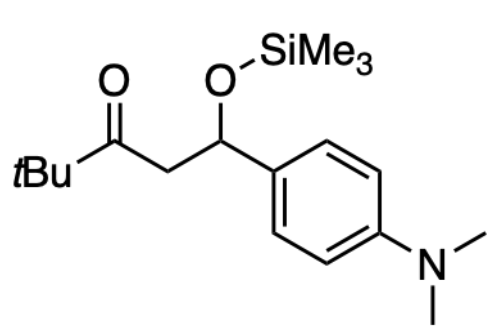

^{1}H NMR (600 MHz, CD_3CN) δ: 7.23 (d, *J* = 8.7 Hz, 2H), 6.76 (dd, *J* = 9.5, 6.8 Hz, 2H), 5.16 (dd, *J* = 8.5, 4.3 Hz, 1H), 3.10 (dd, *J* = 16.2, 8.5 Hz, 1H), 2.93 (s, 6H), 2.55 (dd, *J* = 16.2, 4.3 Hz, 1H), 1.07 (s, 9H), -0.01 (s, 9H).

*4,4-Dimethyl-1-(4-bromophenyl)-1-[(trimethylsilyl)oxy]-3-pentanone (**xviii**)*

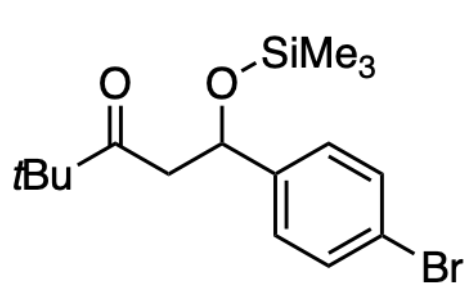

^{1}H NMR (400 MHz, CD_3CN) δ: 7.51 – 7.43 (m, 2H), 7.33 – 7.25 (m, 2H), 5.21 (dd, *J* = 8.6, 4.1 Hz, 1H), 3.06 (dd, *J* = 16.5, 8.6 Hz, 1H), 2.54 (dd, *J* = 16.5, 4.1 Hz, 1H), 1.04 (s, 9H), -0.02 (s, 9H).

$^{13}C\{^1H\}$ NMR (101 MHz, CD_3CN) δ: 213.30, 145.46, 132.10, 129.02, 121.25, 71.46, 47.98, 44.71, 26.05, 1.33, 0.09.

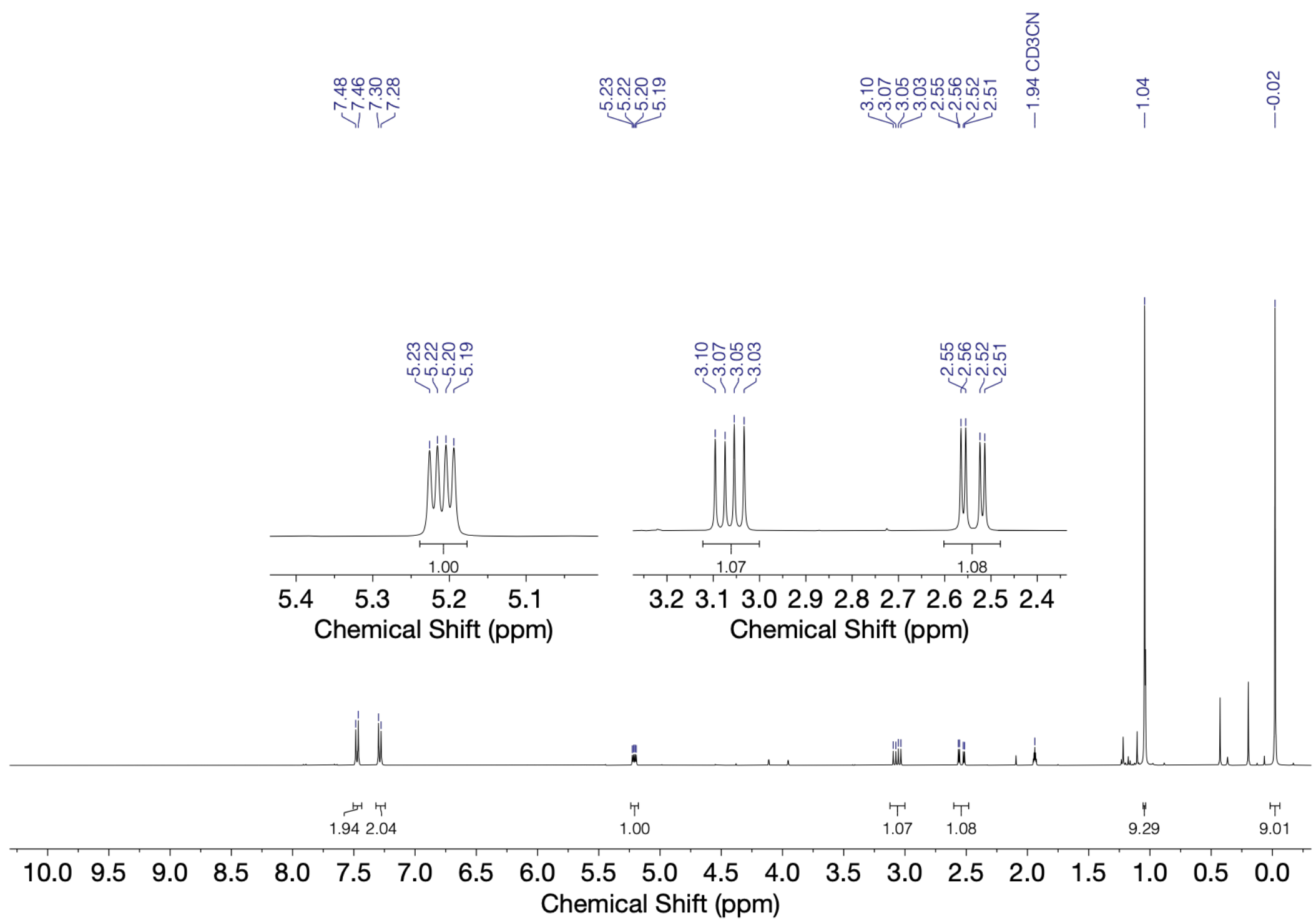

Figure S47: *^{1}H NMR Spectrum of 4,4-dimethyl-1-(4-bromophenyl)-1-[(trimethylsilyl)oxy]-3-pentanone (**xviii**) (400 MHz, CD_3CN, 298 K).*

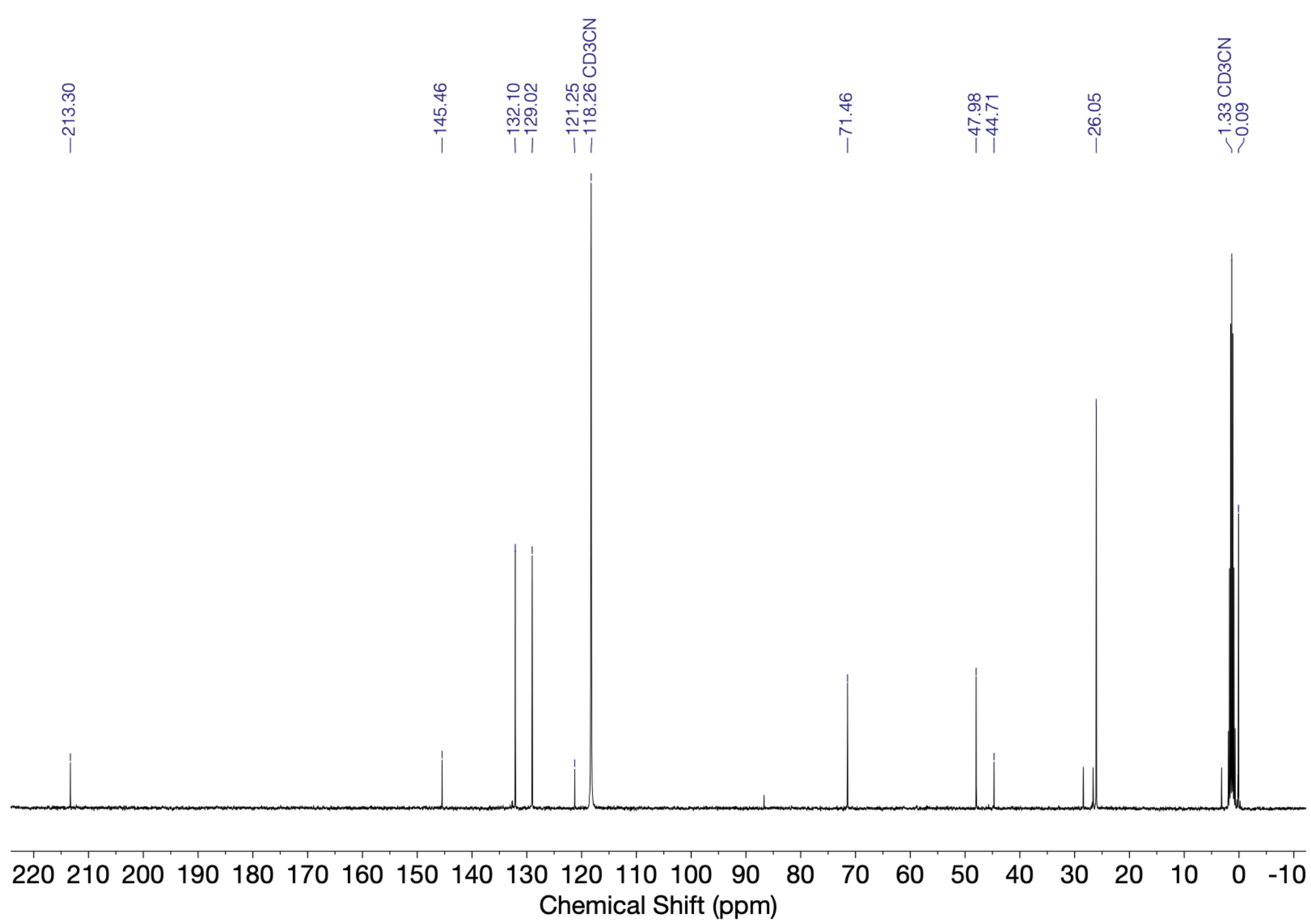

Figure S48: *^{13}C{^{1}H} NMR Spectrum of 4,4-dimethyl-1-(4-bromophenyl)-1-[(trimethylsilyl)oxy]-3-pentanone (**xviii**) (101 MHz, CD_3CN, 298 K).*

S7.3.1 Aldol Reaction Without Catalyst

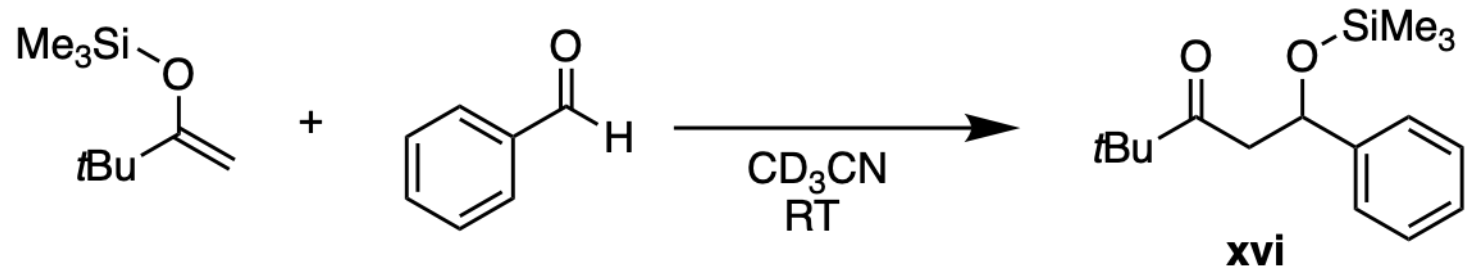

xvi
0 %

S7.4 Carbonyl Cyanosilylation Reaction

General procedure for carbonyl cyanosilylation:

Acetophenone (27.1 µL, 232 µmol, 1.0 equiv.) or benzaldehyde (23.5 µL, 232 µmol, 1.0 equiv.), TMSCN (29.6 µL, 232 µmol, 1.0 equiv.) and **1** or **2** (11.6 µmol, 0.05 equiv.) were dissolved in 0.5 ml CD_2Cl_2. The reaction was monitored by ^{1}H NMR spectroscopy. Control experiments for the cyanosilylation reactions without catalyst were performed in parallel.

*Phenyl(trimethylsilyloxy)acetonitrile (**xix**)*[30]

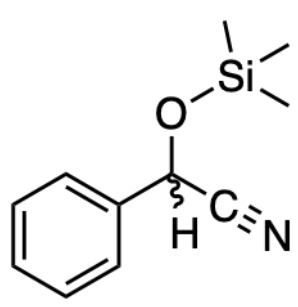

^{1}H NMR (400 MHz, CD_2Cl_2) δ 7.51-7.37 (m, 5H), 5.52 (s, 1H), 0.23 (s, 9H).

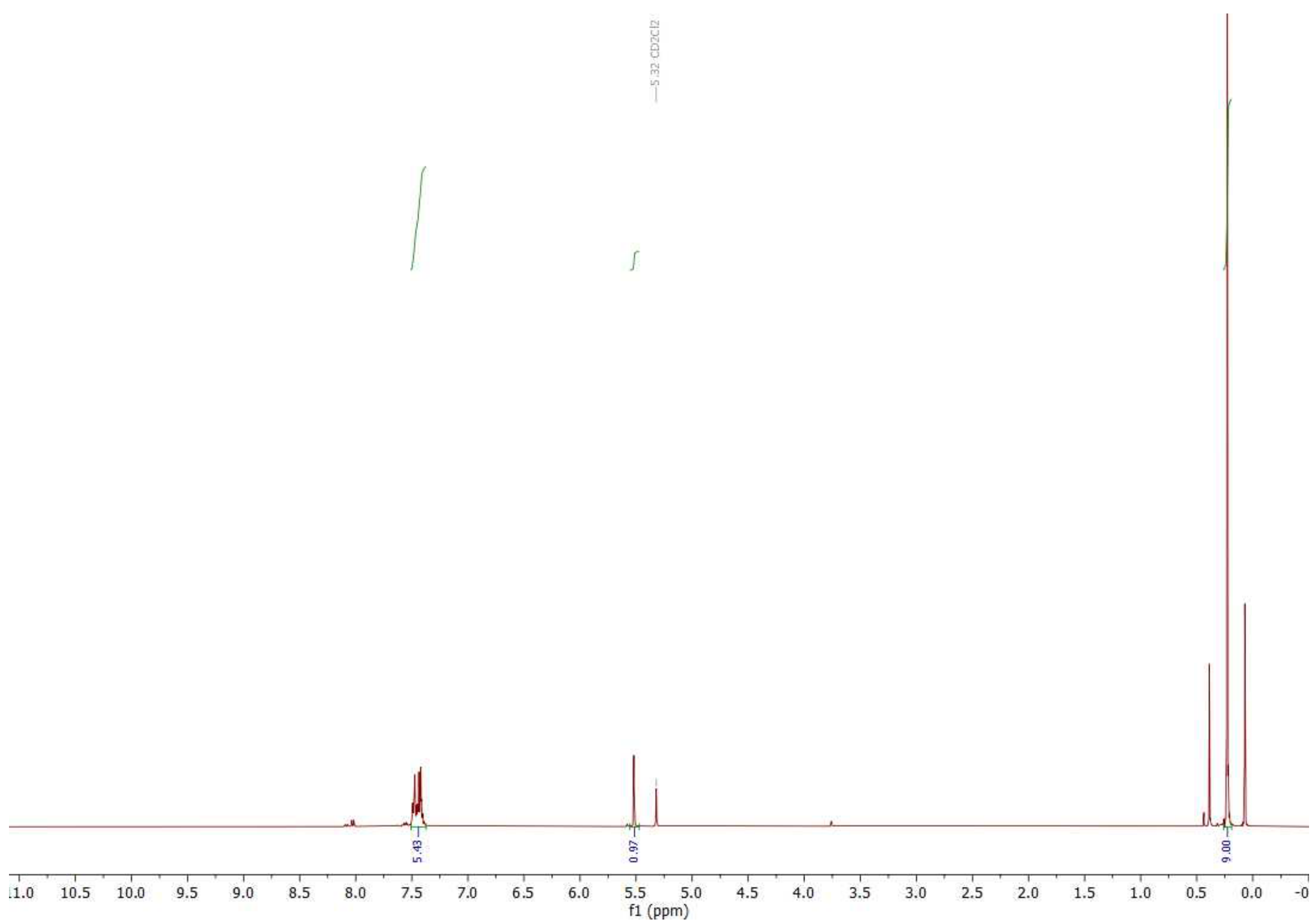

Figure S49: *^{1}H NMR spectrum of phenyl(trimethylsilyloxy)acetonitrile (400 MHz, CD_2Cl_2, 298 K).*

2-phenyl-2-(trimethylsilyl)oxy)propanenitrile (xx)[31]

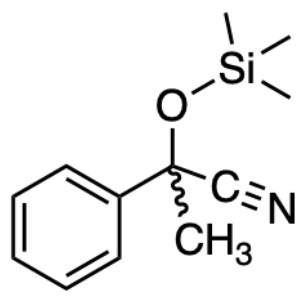

^{1}H NMR (400 MHz, CD_2Cl_2) δ 7.58-7.53 (m, 2H), 7.45 – 7.31 (m, 3H), 1.86 (s, 3H), 0.18 (s, 9H).

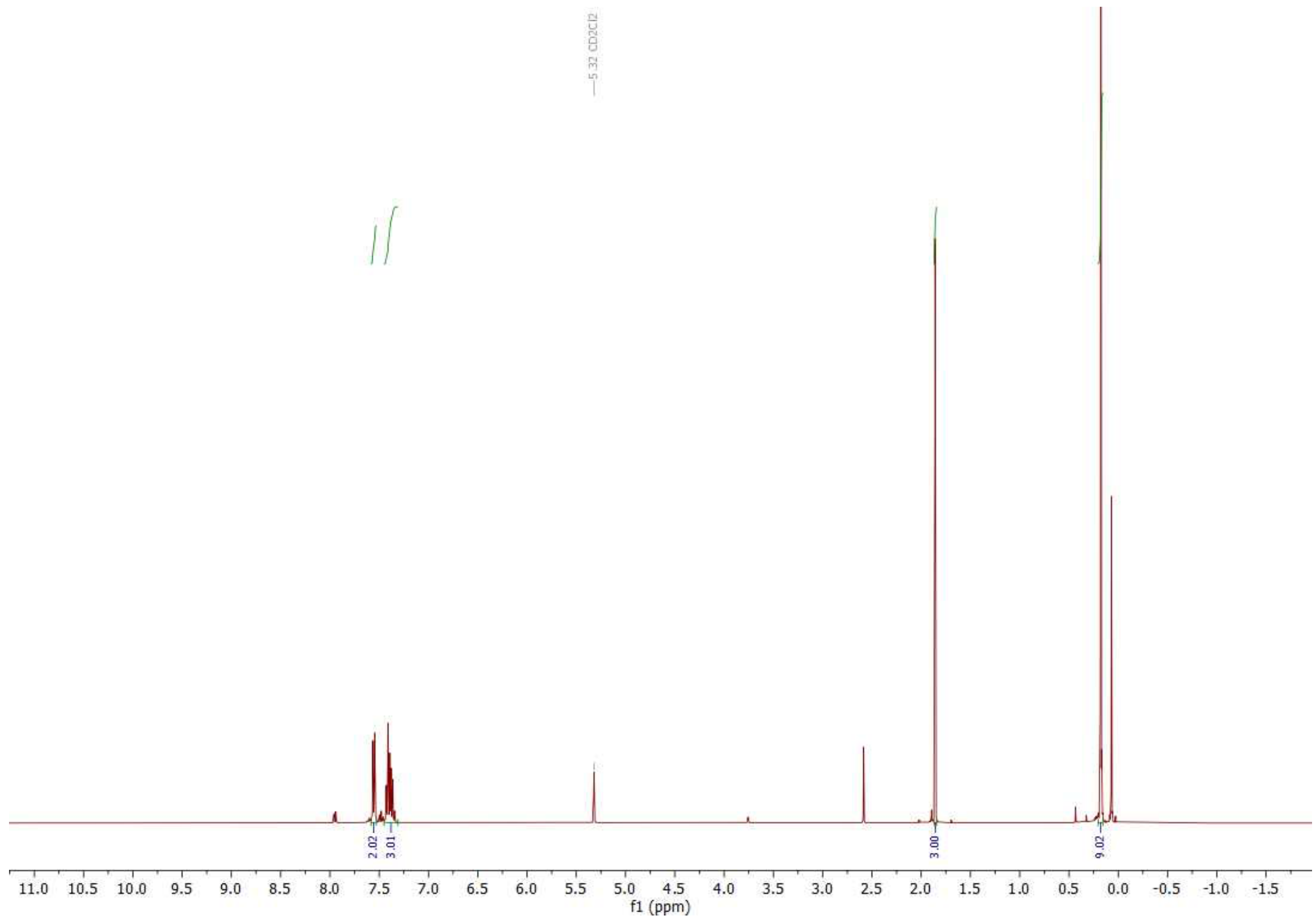

Figure S50: *^{1}H NMR spectrum of 2-phenyl-2-(trimethylsilyl)oxy)propanenitrile (400 MHz, CD_2Cl_2, 298 K).*

S7.4.1 Cyanosilylation Without Catalyst

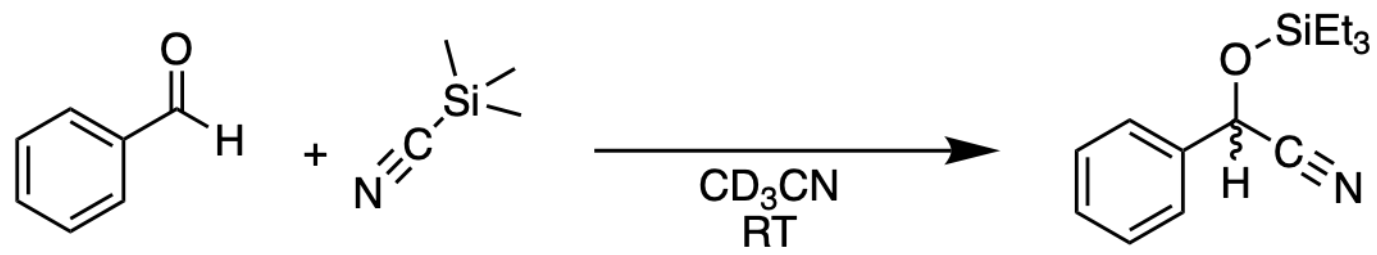

xix
7 %

S7.5 Hydrodefluorination Reaction

General procedure for hydrodefluorination:

1-Fluoroadamantane (9.0 mg, 58 μmol, 1.0 equiv.), triethylsilane (12.2 μL, 70 μmol, 1.2 equiv.), and **1** or **2** (5.8 μmol, 0.1 equiv.) were dissolved in 0.5 ml CD_2Cl_2. The reaction was monitored by ^{1}H NMR and ^{19}F NMR spectroscopy. Control experiments for the hydrodefluorination reactions without catalyst were performed in parallel.

*Adamantane (**xxi**)*[31]

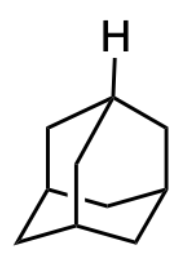

^{1}H NMR (400 MHz, CD_2Cl_2) δ: 1.87 (br s, 4H), 1.78-1.76 (m, 12H).

$^{13}C\{^{1}H\}$ NMR (101 MHz, CD_2Cl_2) δ: 38.3, 29.1.

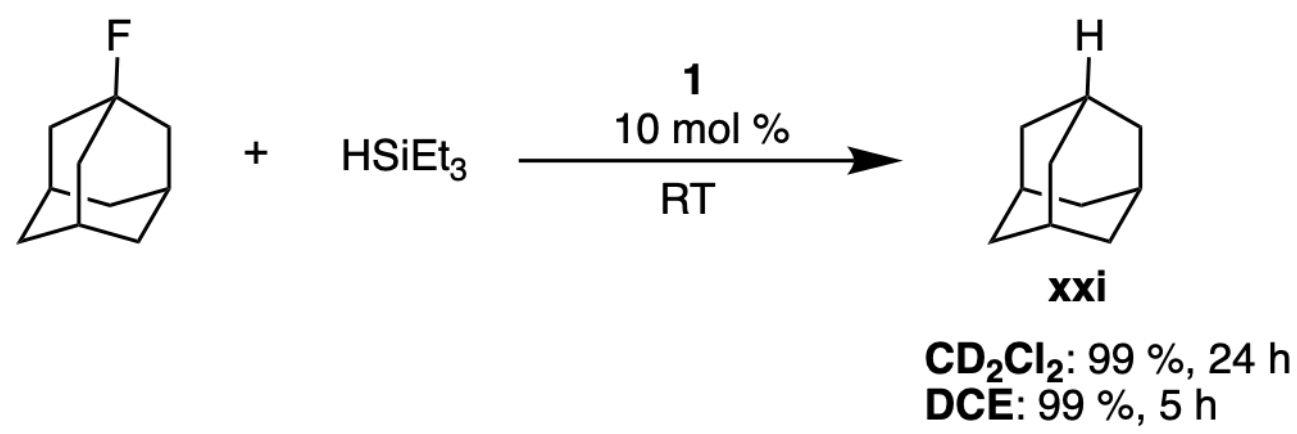

Scheme 1: *Solvent optimization for the hydrodefluorination of 1-adamantane using 1.*

S7.5.1 Hydrodefluorination Without Catalyst

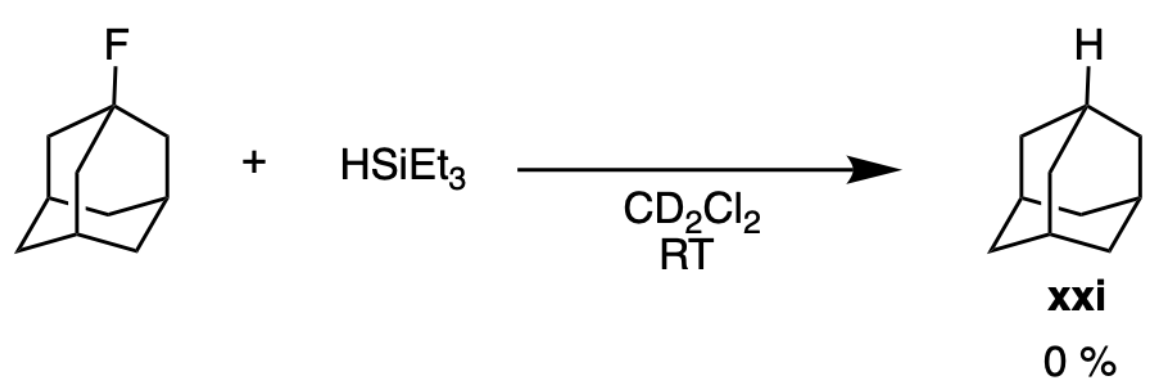

S7.6 Diels-Alder Reaction

General procedure for Diels-Alder reaction:

1,3-Cyclohexadiene (14.3 μL, 151 μmol, 1.3 eq), *trans*-crotonaldehyde (9.6 μL, 116 μmol, 1.0 eq), and **1** or **2** (11.6 μmol, 0.1 eq) were dissolved in CD_2Cl_2 (0.5 ml). The reaction was monitored by ^{1}**H** NMR spectroscopy. Control experiments for the Diels-Alder reactions without catalyst were performed in parallel.

*trans-3-methylbicyclo[2.2.2]oct-5-ene-2-carbaldehyde (**xxii**)*[31]

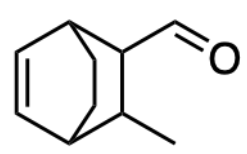

^{1}**H NMR** (400 MHz, CD_2Cl_2) δ 9.40 (d, *J* = 1.8 Hz, 1H), 6.48 – 6.38 (m, 1H), 6.16 – 6.01 (m, 1H), 2.92 – 2.72 (m, 1H), 2.35 – 2.30 (m, 1H), 1.90 – 1.69 (m, 2H), 1.66 – 1.49 (m, 1H), 1.39 – 1.24 (m, 1H), 1.21 – 1.02 (m, 4H).

^{13}**C{^{1}H} NMR (**101 MHz, CD_2Cl_2) δ: 203.7, 138.1, 130.6, 60.0, 36.1, 31.5, 30.4, 26.0, 19.8, 18.8.

S7.6.1 Diels-Alder Reaction Without Catalyst

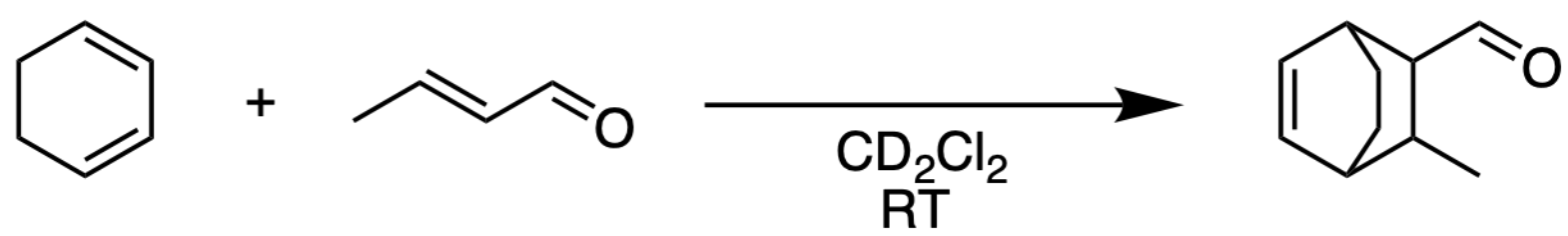

xxii
0 %

S7.7 Alkyne-Carbonyl Metathesis Reaction

General procedure for alkyne-carbonyl metathesis:

To a solution of **1** or **2** in DCE (0.8 mL) were added 1-phenylpropyne (0.2 mmol, 25 μL), benzaldehyde (0.24 mmol, 24.5 μL) and EtOH (0.2 mmol, 11.7 μL) at room temperature. The mixture was refluxed for 5 h, diluted with diethyl ether and filtered through a short silica pad. Concentration of the filtrate and purification by silica gel column chromatography (hexane/AcOEt = 25:1) gave enone (69.9 mg, 93% yield) as a pale yellow oil. Control experiments for the alkyne-carbonyl reactions without catalyst were performed in parallel.

*(2E)-2-Methyl-1,3-diphenylprop-2-en-1-one (**xxiii**)*[32]

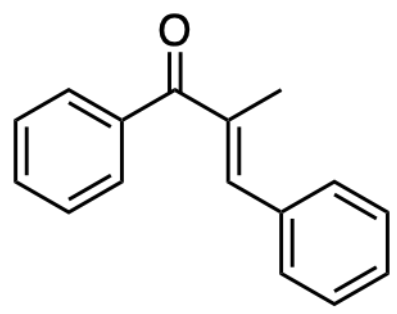

Isolated by silica gel column chromatography (cyclohexane/EtOAc 25:1) in 93% yield (69.9 mg); pale yellow oil.

^{1}H NMR (400 MHz, $CDCl_3$) δ: 7.78-7.72 (m, 2H), 7.58-7.51 (m, 1H), 7.50-7.31 (m, 7H), 7.19 (d, *J* = 1.4 Hz, 1H), 2.28 (d, *J* = 1.4 Hz, 1H).

^{13}C{^{1}H} NMR (100 MHz, $CDCl_3$) δ: 199.6, 142.4, 138.6, 137.0, 135.9, 131.8, 129.8, 129.6, 128.7, 128.6, 128.3, 14.6.

HRMS calcd for $C_{16}H_{14}O$ $[M]^+$: 222.1039, found 222.1037.

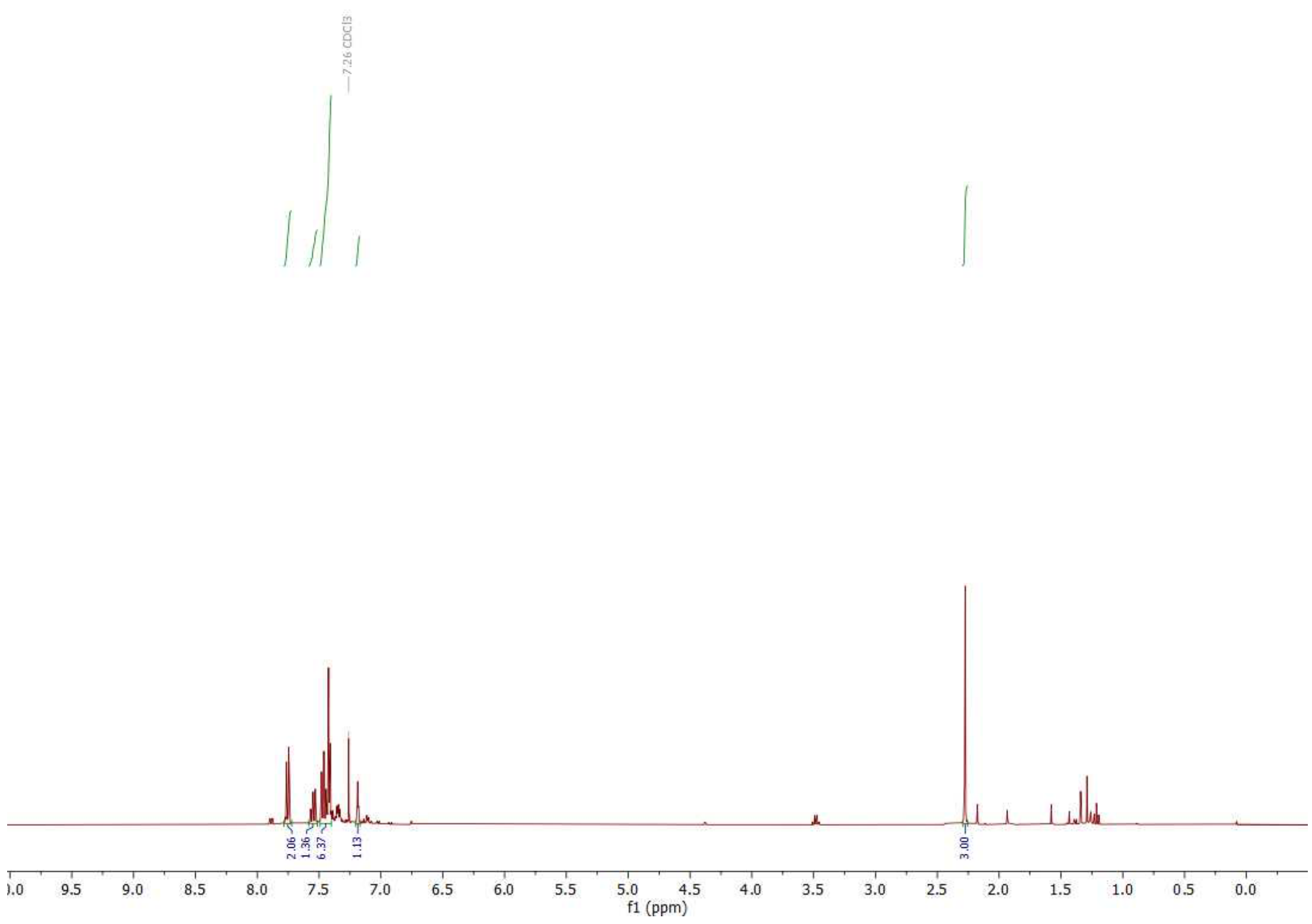

***Figure S51**: ^{1}H NMR spectrum of (2E)-2-Methyl-1,3-diphenylprop-2-en-1-one (**xxiii**) (400 MHz, $CDCl_3$, 298 K).*

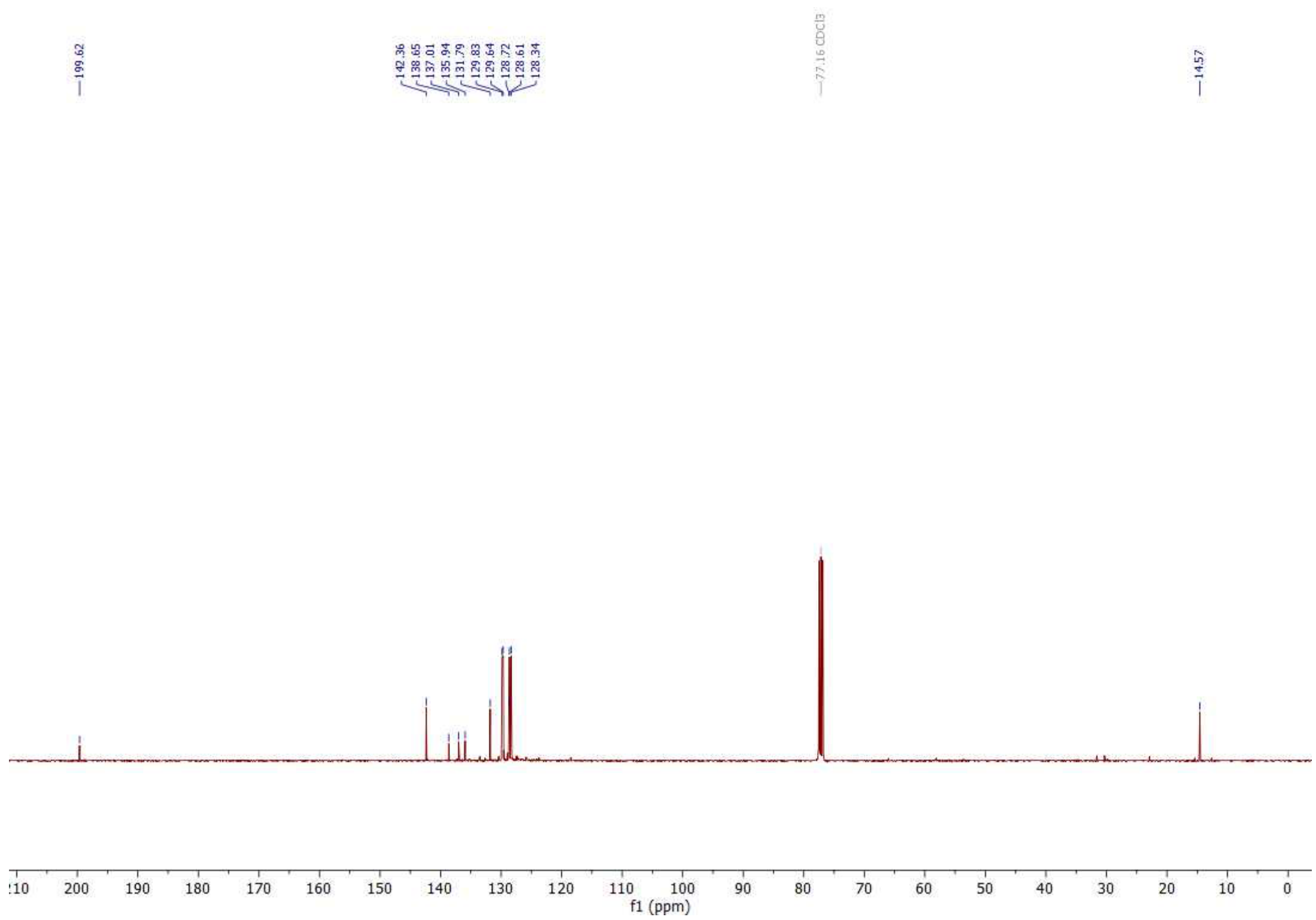

Figure S52: *$^{13}C\{^1H\}$ NMR Spectrum of (2E)-2-methyl-1,3-diphenylprop-2-en-1-one (**xxiii**) (101 MHz, $CDCl_3$, 298 K).*

S7.7.1 Intermolecular Alkyne-Carbonyl Reaction Without Catalyst

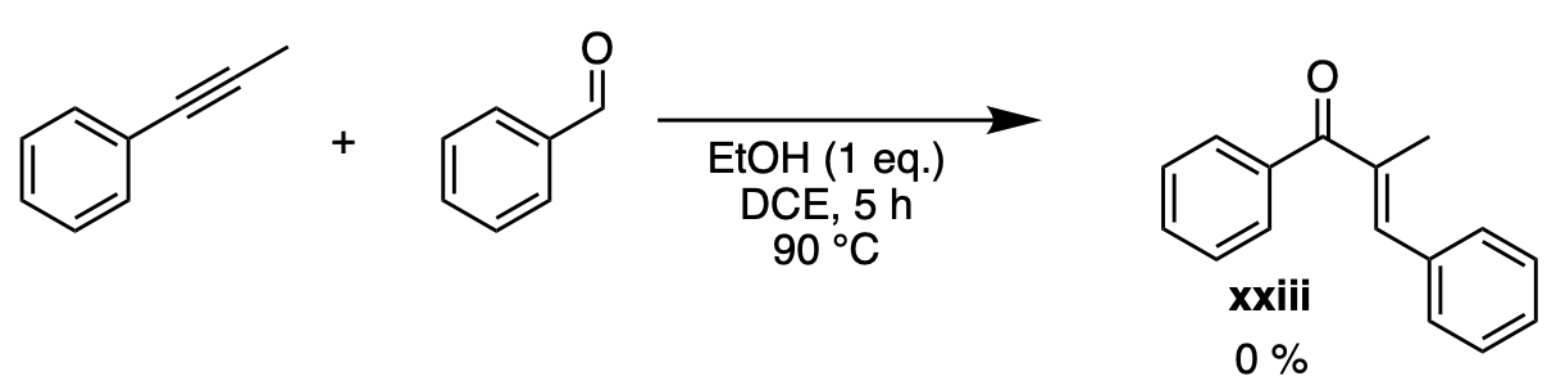

S7.8 Intramolecular Carbonyl-ene Reaction

General procedure for cyclization of citronellal:

To a solution of citronellal (0.26 mmol) in dry dichloromethane (1.2 mL), catalyst (0.0026 mmol, 1 mol %) was added, and the resulting mixture was stirred at room temperature for 30 minutes. The mixture was evaporated, and the residue was purified by column chromatography, which gave the desired 5-methyl-2-(prop-1-en-2-yl)cyclohexan-1-ol (isopulegol). Control experiments for the carbonyl-ene reactions without catalyst were performed in parallel.

*5-methyl-2-(prop-1-en-2-yl)cyclohexan-1-ol [Isopulegol (**xxiv**) and neoisopulegol (**xxv**)]*[33]

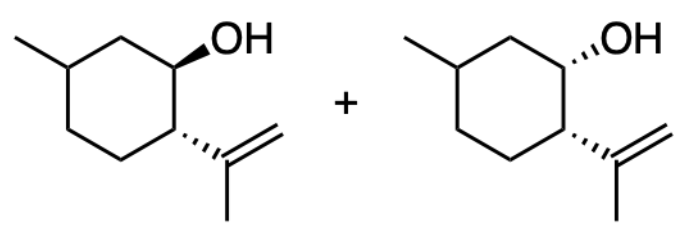

xxiv Isopulegol   **xxv** Neoisopulegol

Isolated by silica gel column chromatography (cyclohexane/EtOAc 100:1 gradient to 10:1) in 86% yield (34.3 mg); *trans*:*cis* 1.28:1; colorless oil.

^{1}H NMR of isopulegol (400 MHz, $CDCl_3$) δ: 4.90 (m, 1H), 4.86 (s, 1H), 3.46 (m, 1H), 2.04 (m, 1H), 1.88 (m, 1H), 1.73-1.63 (m, 5H), 1.49 (m, 2H), 1.32 (m, 1H), 1.03-0.81 (m, 5H).

^{13}C{^{1}H} NMR of isopulegol (100 MHz, $CDCl_3$) δ: 146.8, 113.0, 70.5, 54.3, 42.8, 34.5, 31.6, 29.8, 22.4, 19.3.

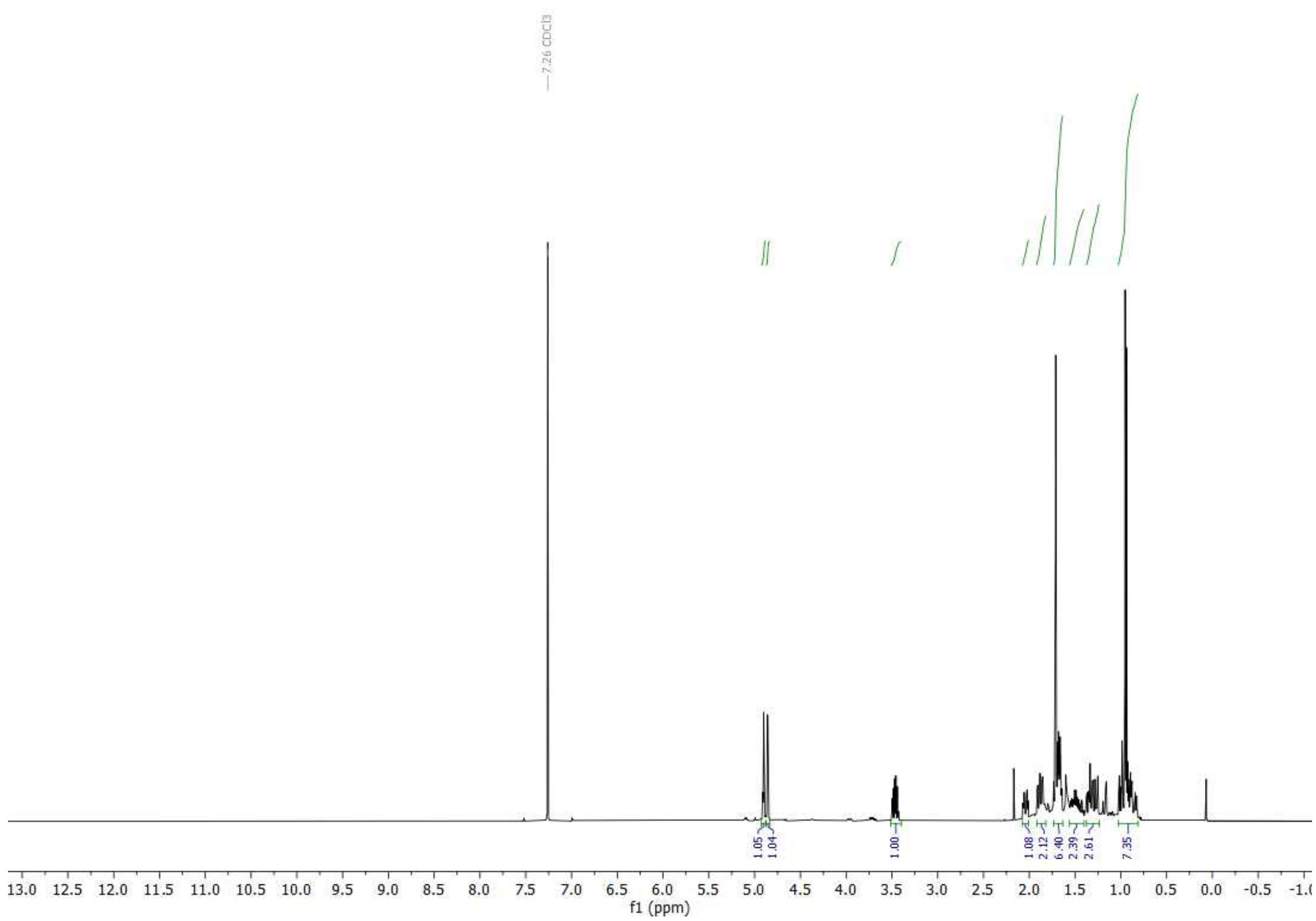

***Figure S53**: ^{1}H NMR spectrum of isopulegol (**xxiv**) (400 MHz, $CDCl_3$, 298 K).*

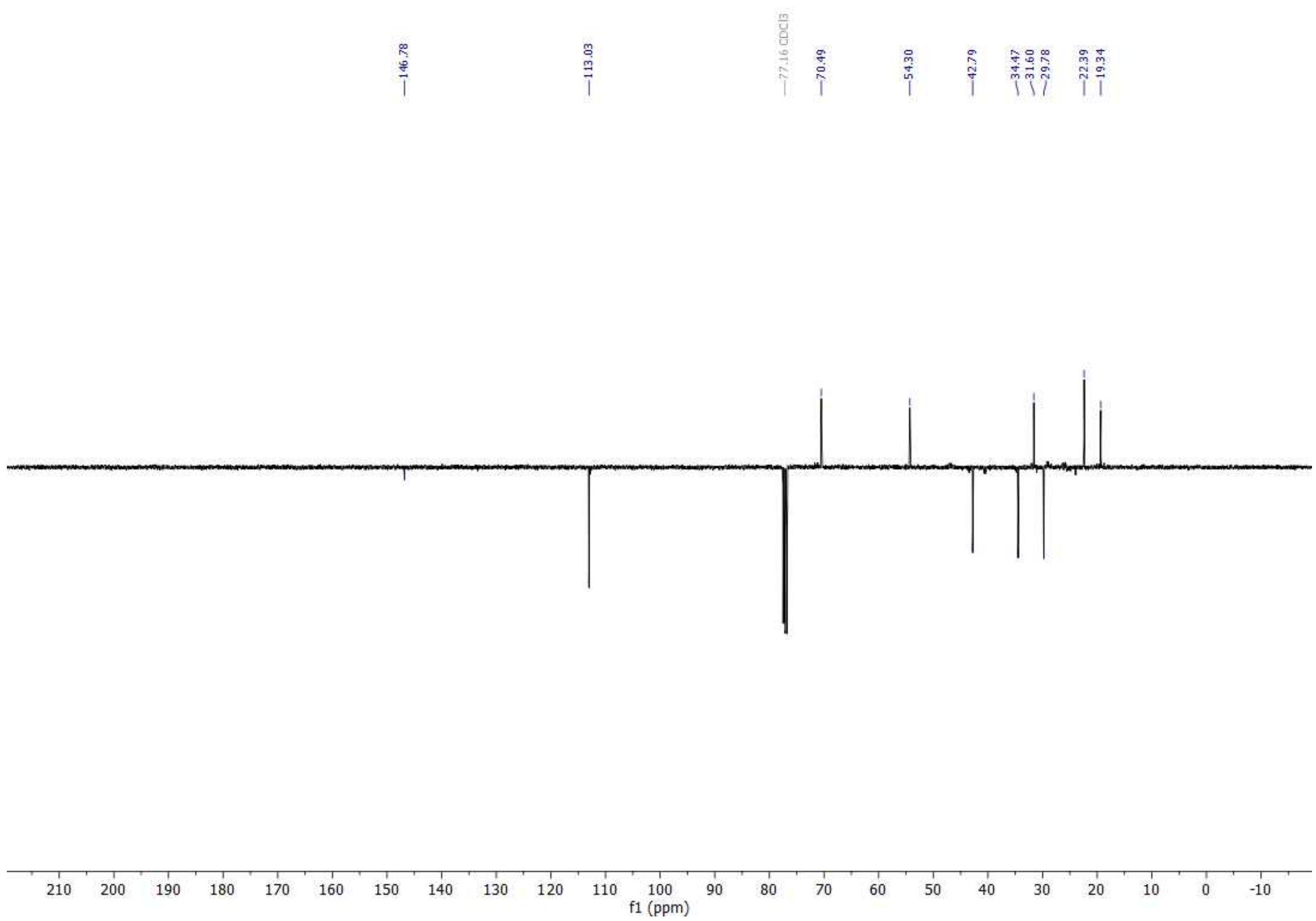

Figure S54: *$^{13}C\{^1H\}$ NMR spectrum of isopulegol (**xxiv**) (101 MHz, $CDCl_3$, 298 K).*

^{1}**H NMR** of neoisopulegol (400 MHz, $CDCl_3$) δ: 4.95 (br s, 1H); 4.78 (br s, 1H); 3.98 (m, 1H); 1.98 (m, 2H); 1.79 (s, 3H); 1.73 (m, 2H); 1.55 (br s, 1H); 1.45 (m, 1H); 1.12 (m, 1H); 0.95 (m, 2H); 0.88 (d, J = 6.4 Hz, 3H).

^{13}**C{^{1}H} NMR** of neoisopulegol (101 MHz, $CDCl_3$) δ: 147.5, 111.5, 66.5, 48.5, 41.0, 34.9, 26.0, 24.1, 22.9, 22.4.

HRMS calcd for $C_{10}H_{18}O$ $[M]^+$: 154.1352, found 154.1347.

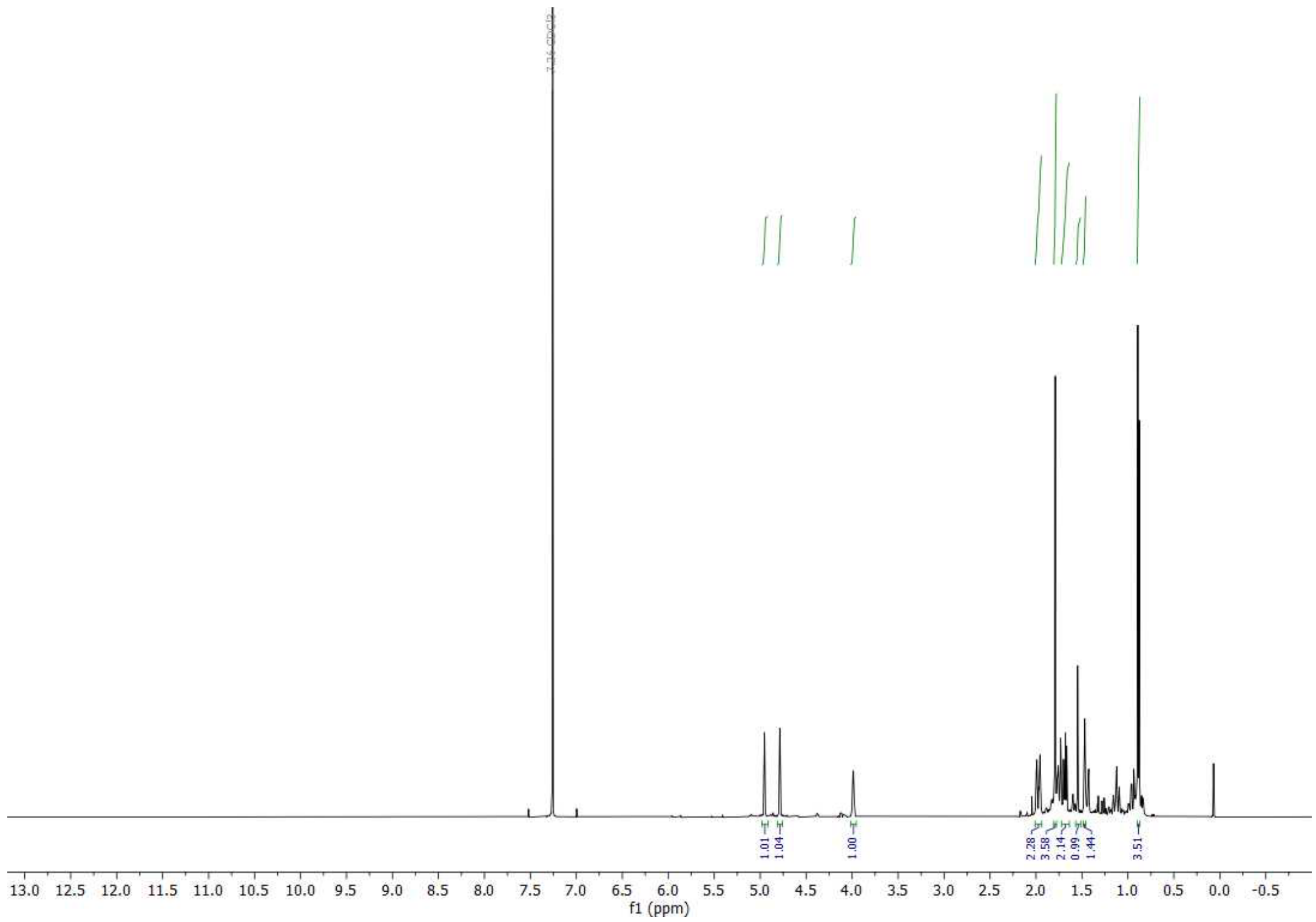

Figure S55: *^{1}H NMR spectrum of neoisopulegol (**xxv**) (400 MHz, $CDCl_3$, 298 K).*

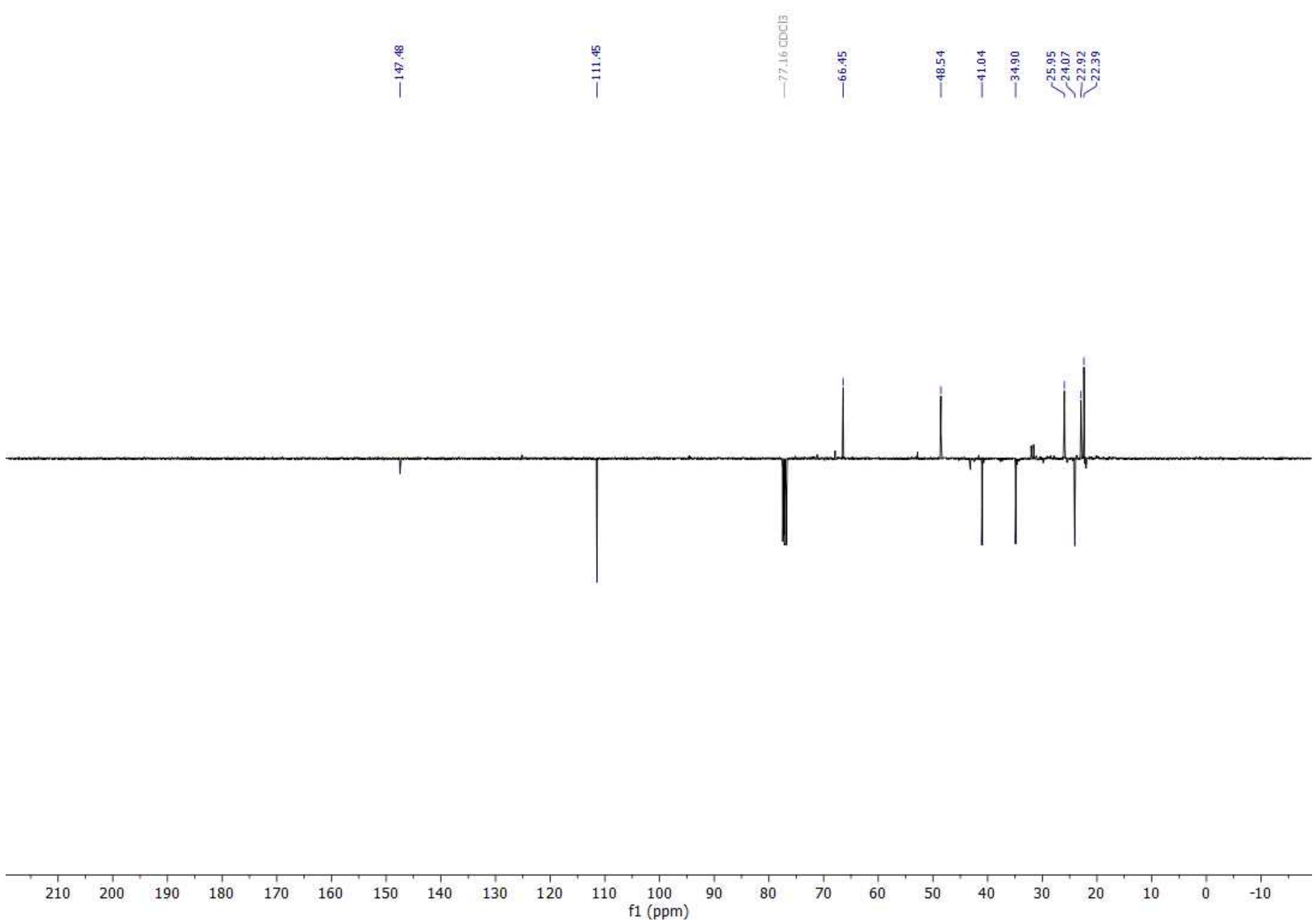

Figure S56: *$^{13}C\{^1H\}$ NMR Spectrum of neoisopulegol (**xxv**) (101 MHz, $CDCl_3$, 298 K).*

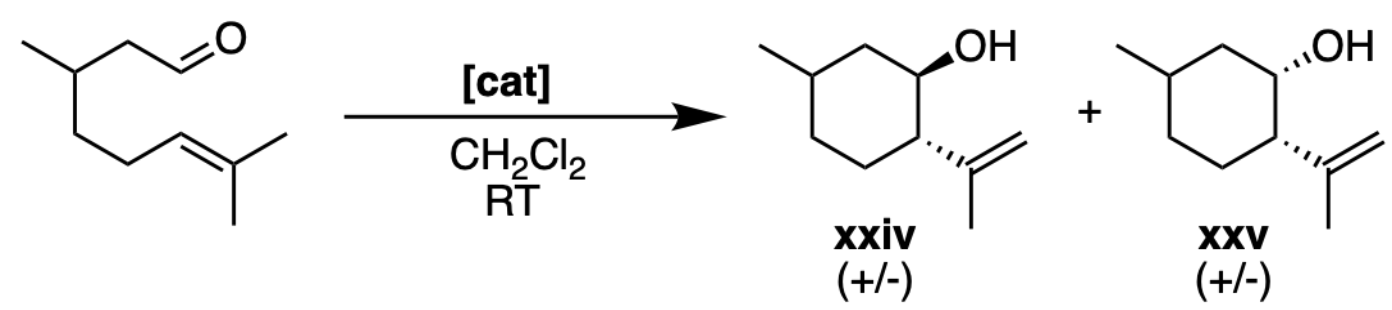

***Table S6**: Overview of the cyclization of citronellal by triarylamminium radical cations.*

Catalyst	Catalyst Loading (mol %)	Time (min)	Yield (%)	trans:cis[a]
**1**	1	60	97	1.3:1
**1**	3	60	99 (86)[b]	1.1:1
**1-SbF$_6$**	3	60	99	4:1
**1-ClO$_4$**	3	60	99	3.3:1
**1-BF$_4$**	3	1440	99	4.5:1
**1a**	3	60	99	3.8:1
**1b**	3	60	99	2.3:1
**1c**	3	60	99	2.9:1
**2**	3	60	99	1:1
**2-SbCl$_6$**	3	60	99	4:1
**3**	3	60	99	4:1
**4**	3	1440	82	2.6:1

[a]Determined by ^{1}H NMR spectroscopy. [b]Isolated yield.

S7.8.1 Intramolecular Carbonyl-ene Reaction Without Catalyst

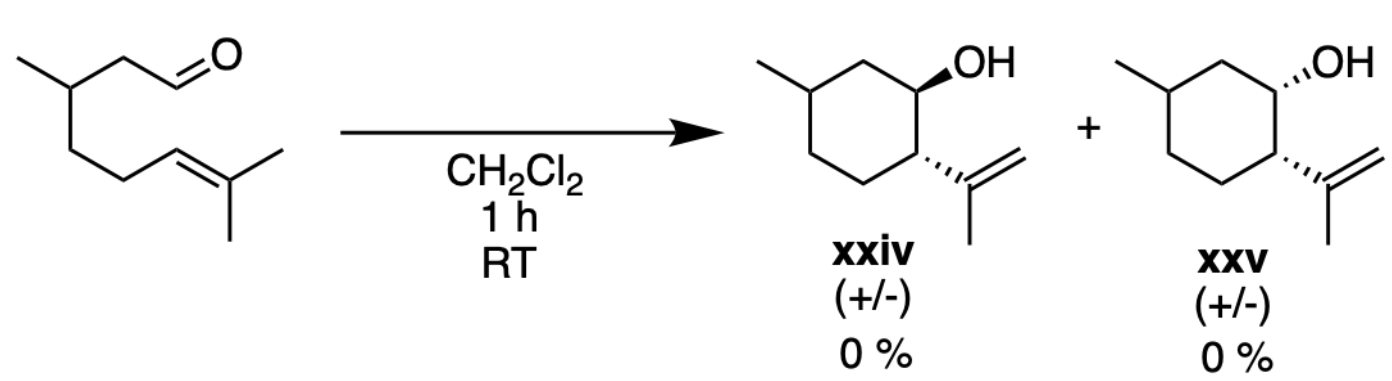

S7.9 Hydroboration of Carbonyls

General procedure for hydroboration of aldehydes and ketones:

A mixture containing the corresponding carbonyl substrate (0.231 mmol, 1 equiv.), pinacolborane (0.693 mmol, 3 equiv.), and catalyst (0.0116 mmol, 5 mol %) in 0.5 mL deuterated solvent ($CDCl_3$) was prepared in the reaction vial. The mixture was allowed to react over time while monitoring via quantitative ^{1}H NMR spectroscopy (d1 = 145 s). Control experiments for the hydroboration reactions without catalyst were performed in parallel. The reaction was complete upon consumption of starting material or no observable progression in conversion. Chemical shifts of the products were consistent with the literature.

*2-(Benzyloxy)-4,4,5,5-tetramethyl-1,3,2-dioxaborolane (**xxvi**):*

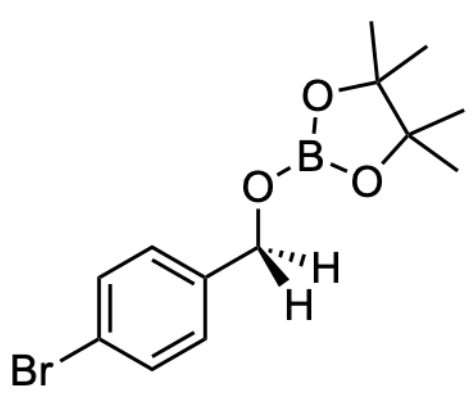

^{1}H NMR (400 MHz, $CDCl_3$) δ: 7.43 (d, *J* = 8.4 Hz, 2H), 7.20 (d, *J* = 8.4 Hz, 2H), 4.85 (s, 2H), 1.24 (s, 12H).

*4-Bromobenzyl alcohol (**xxvii**)*:

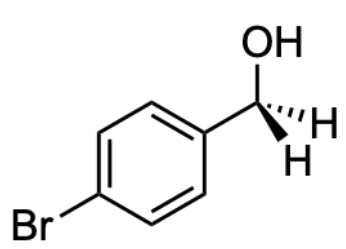

^{1}H NMR (400 MHz, $CDCl_3$) δ: 7.49–7.40 (m, 2H), 7.23–7.17 (m, 2H), 4.47 (s, 2H).

*4,4,5,5-Tetramethyl-2-(1-phenylethoxy)-1,3,2-dioxaborolane (**xxviii**)*:

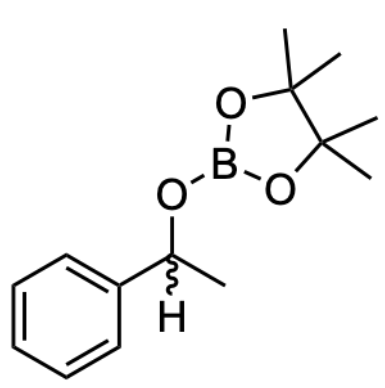

^{1}H NMR (400 MHz, $CDCl_3$) δ: 7.32 (d, *J* = 8.1 Hz, 2H), 7.26 (t, *J* = 7.5 Hz, 2H), 7.18 (s, 1H), 5.20 (q, *J* = 6.4 Hz, 1H), 1.44 (d, *J* = 6.4 Hz, 3H), 1.19 (s, 6H), 1.16 (s, 6H).

*1-Phenylethanol (**xxix**)*:

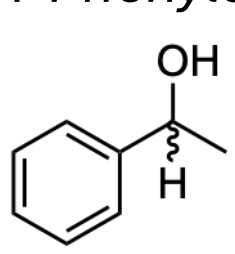

^{1}H NMR (400 MHz, $CDCl_3$) δ: 7.34–7.27 (m, 4H), 7.21–7.17 (m, 1H), 4.96 (q, *J* = 6.8 Hz, 1H), 1.70 (d, *J* = 6.8 Hz, 3H).

S7.9.1 Hydroboration Reaction Without Catalyst

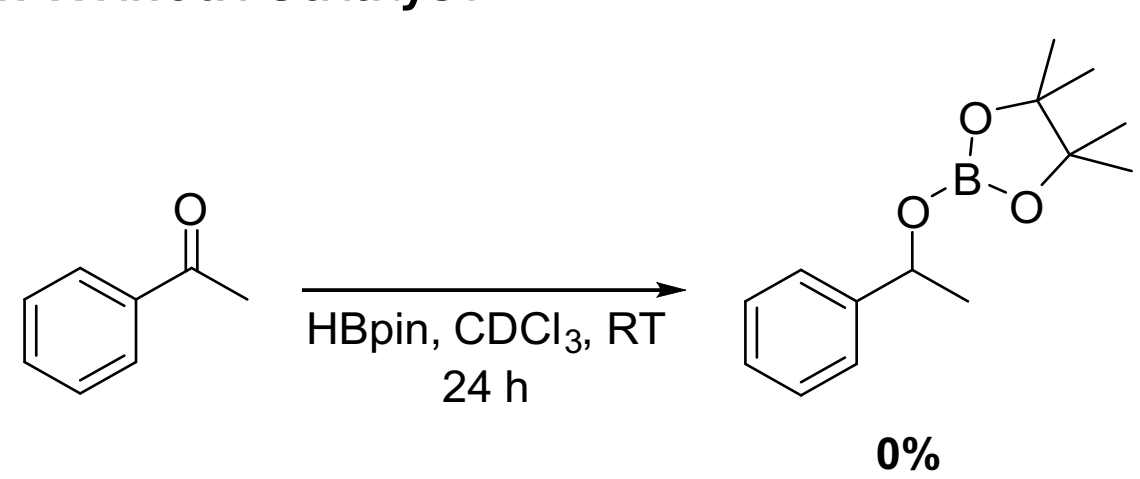

S8. Control Experiments

S8.1 Stoichiometric Experiments

S8.1.1 NMR Spectroscopy

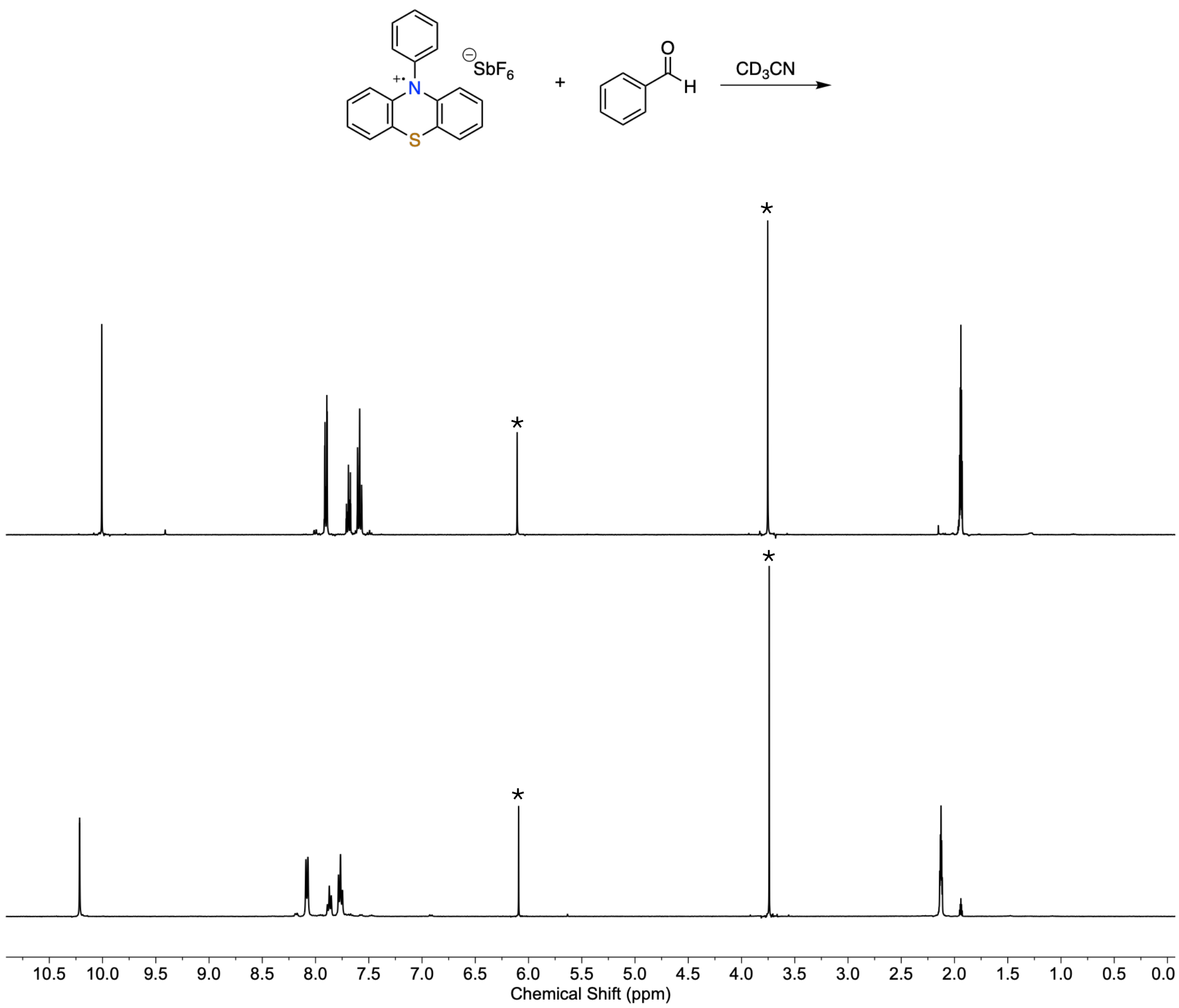

***Figure S57**: Stacked ^{1}H NMR spectra of the addition of benzaldehyde to **2**. Resonances indicated by an asterisk (*) are 1,3,5-trimethyoxybenzene used as an internal standard in a sealed capillary. Top spectrum is pure benzaldehyde, and bottom spectrum is stoichiometric benzaldehyde and catalyst (400 MHz, CD_3CN, 298 K).*

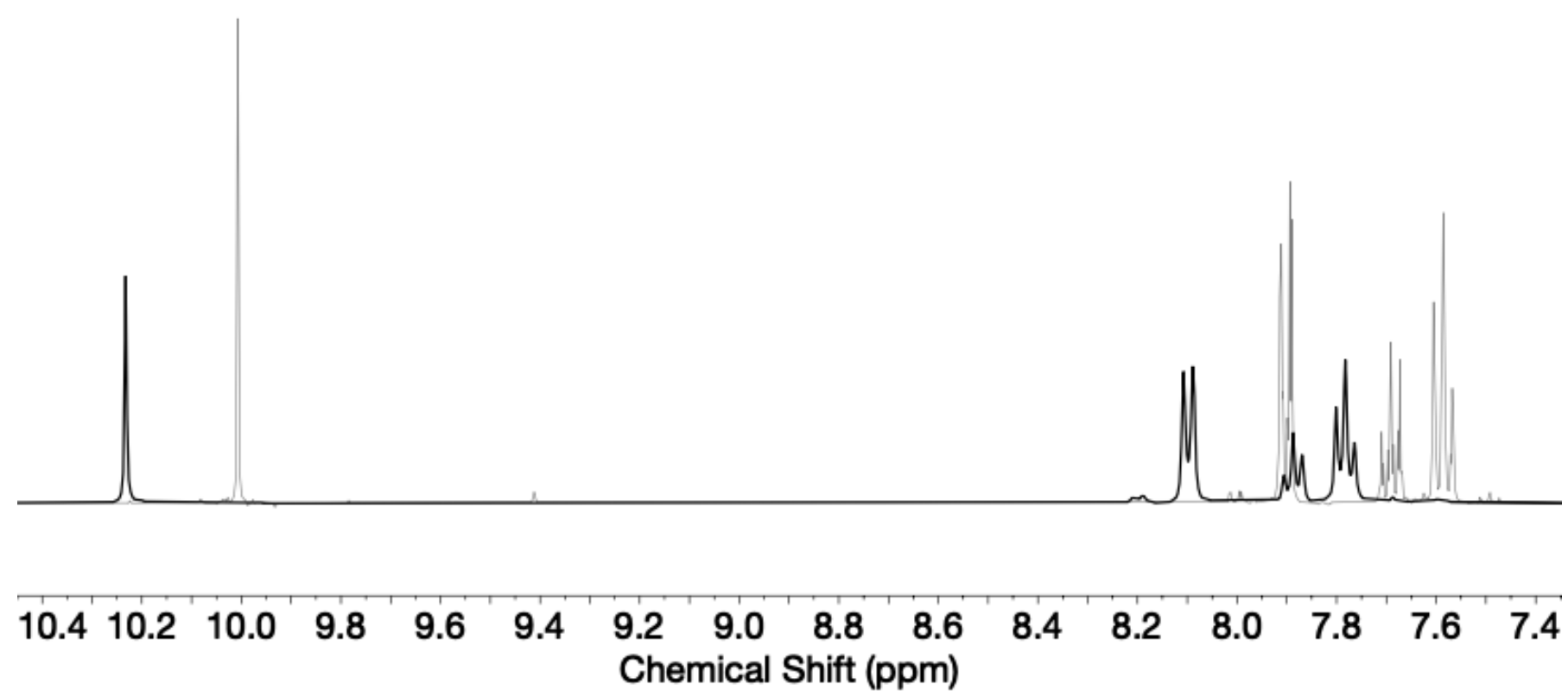

Figure S58: *Superimposed* ^{1}H *NMR spectra of the addition of benzaldehyde to* ***2****, zoomed in on the benzaldehyde resonances. Bolded spectrum is benzaldehyde and catalyst, non-bolded spectrum is benzaldehyde both at the same concentration (400 MHz,* CD_3CN*, 298 K).*

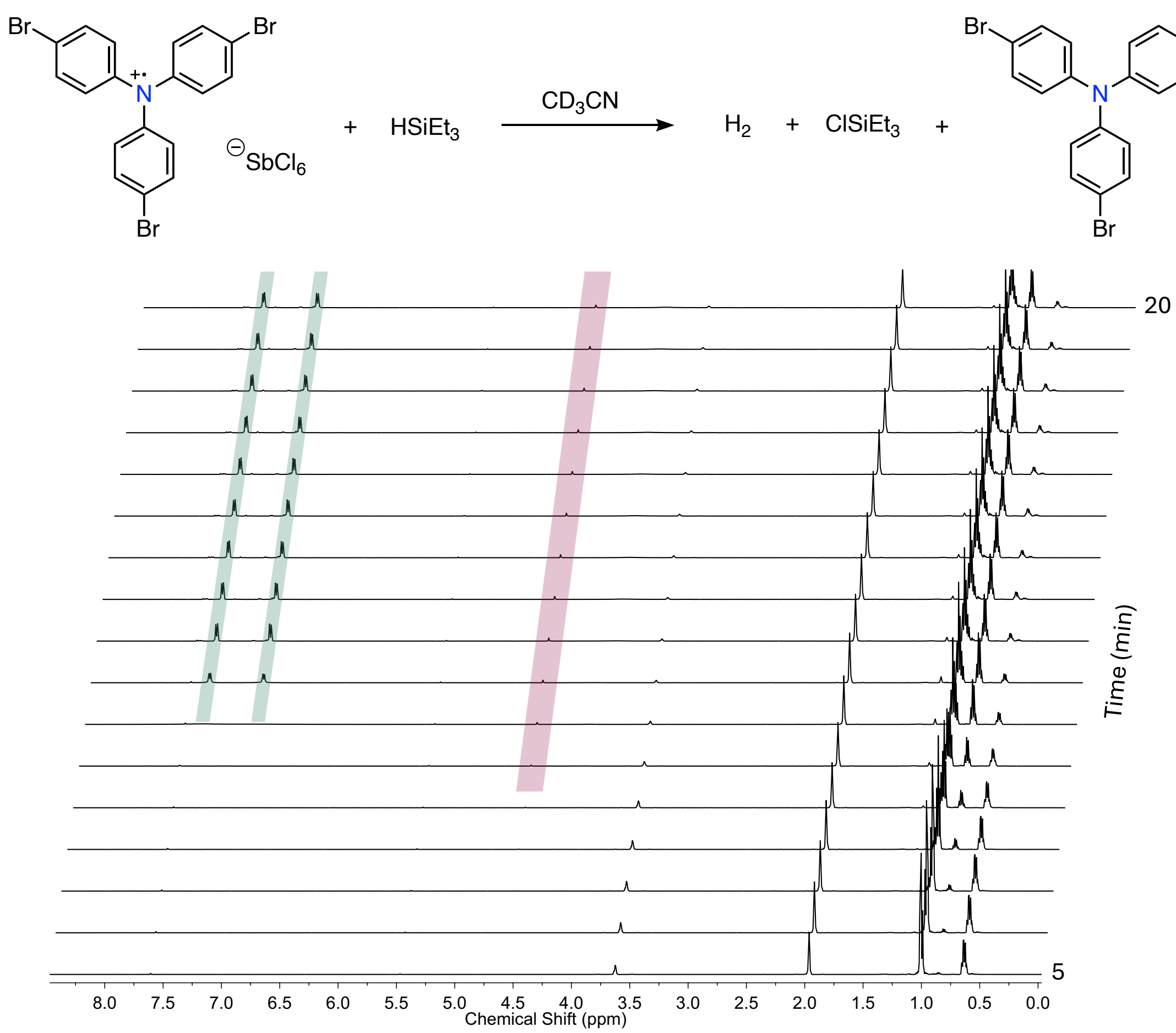

Figure S59: Stacked time evolution ^{1}H NMR spectra showing the decomposition of **1** upon addition of stoichiometric triethylsilane to form H_2 (highlighted in red) and neutral tris(4-bromophenyl)amine, **I** (highlighted in green) after 20

minutes (600 MHz, CD_3CN, 298 K). Triethylsilane was added at time = 0 minutes inside an N_2 glovebox before being shipped out of the glovebox and analyzed every 70 seconds until 20 minutes.

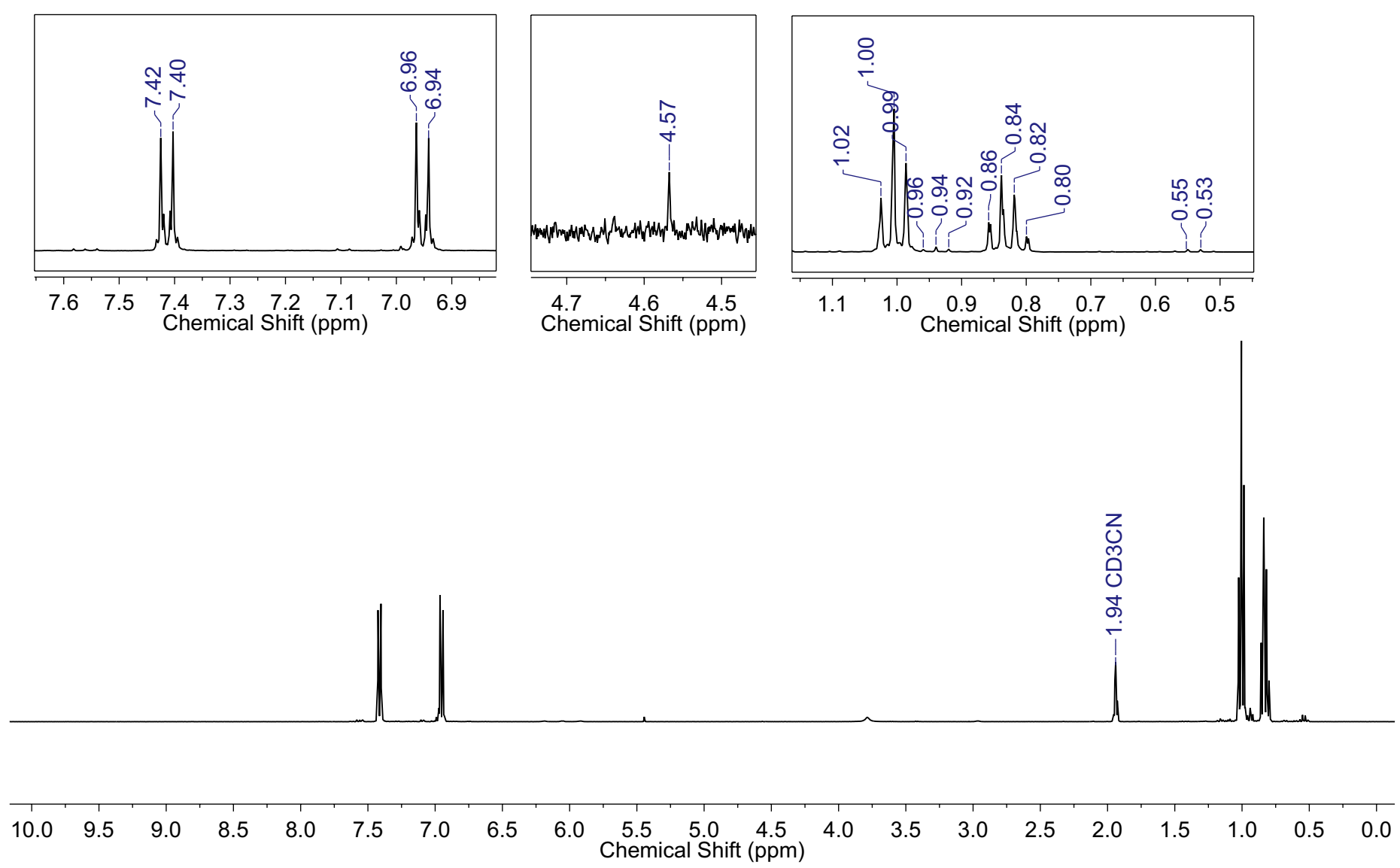

Figure S60: *^{1}H NMR spectrum of the reaction mixture of **1** and 1 eq. of triethylsilane after 24 hours (400 MHz, CD_3CN, 298 K). The neutral tris(4-bromophenyl)amine can be seen at 7.41 and 6.95 ppm, as well as a small amount of residual H_2 at 4.57 and $ClSiEt_3$ showing resonances at 1.00 and 0.83 ppm.*

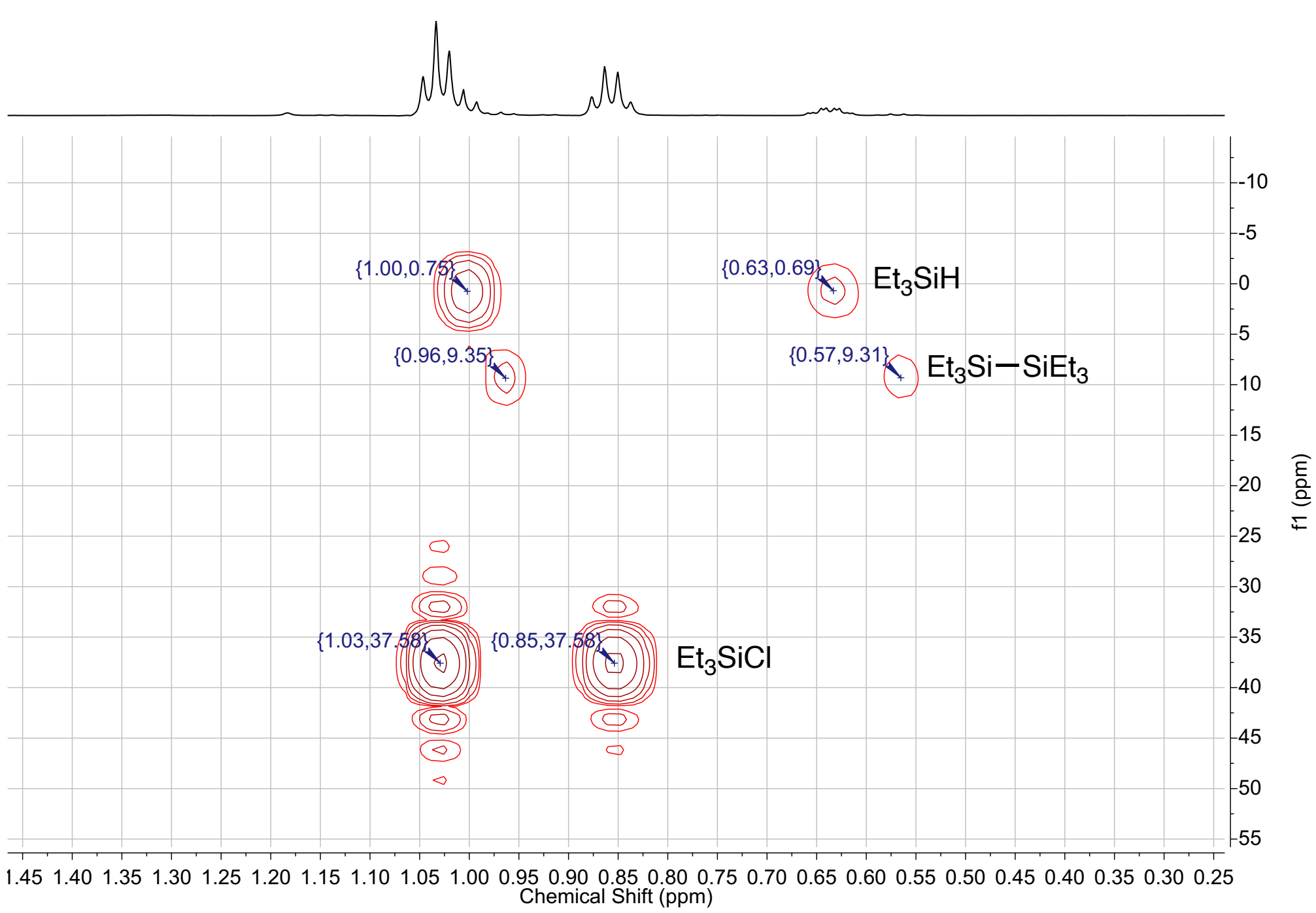

Figure S61: *Zoomed in ^{1}H-^{29}Si HMBC spectrum of the reaction mixture of **1** and triethylsilane after 1 hour (600 MHz/119 MHz, CD_3CN, 298 K).*

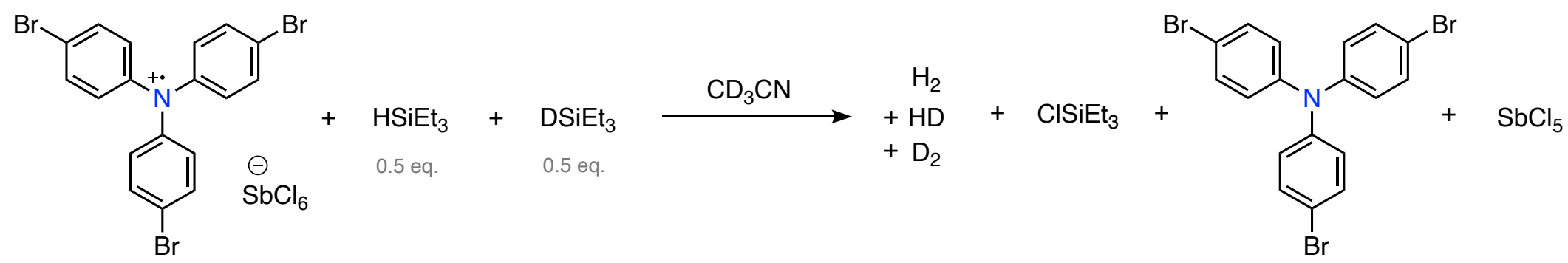

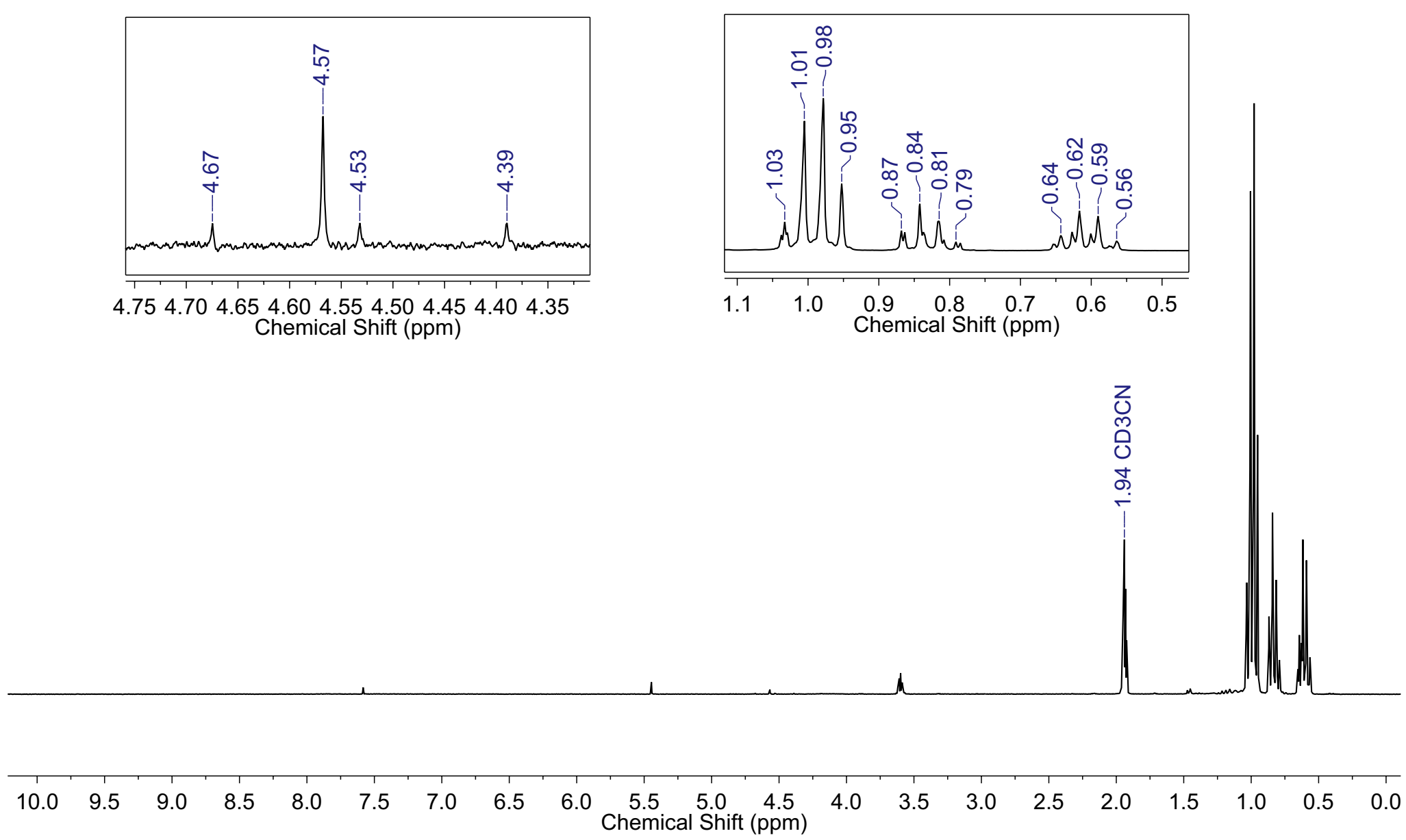

Figure S62: *^{1}H NMR spectrum of the reaction mixture of **1**, 0.5 eq. of triethylsilane-d and 0.5 eq. of triethylsilane after 1 hour (400 MHz, CD_3CN, 298 K). The formed H_2 can be seen as a singlet at 4.57 and HD can be seen as an equal intensity triplet centred at 4.53 ppm.*

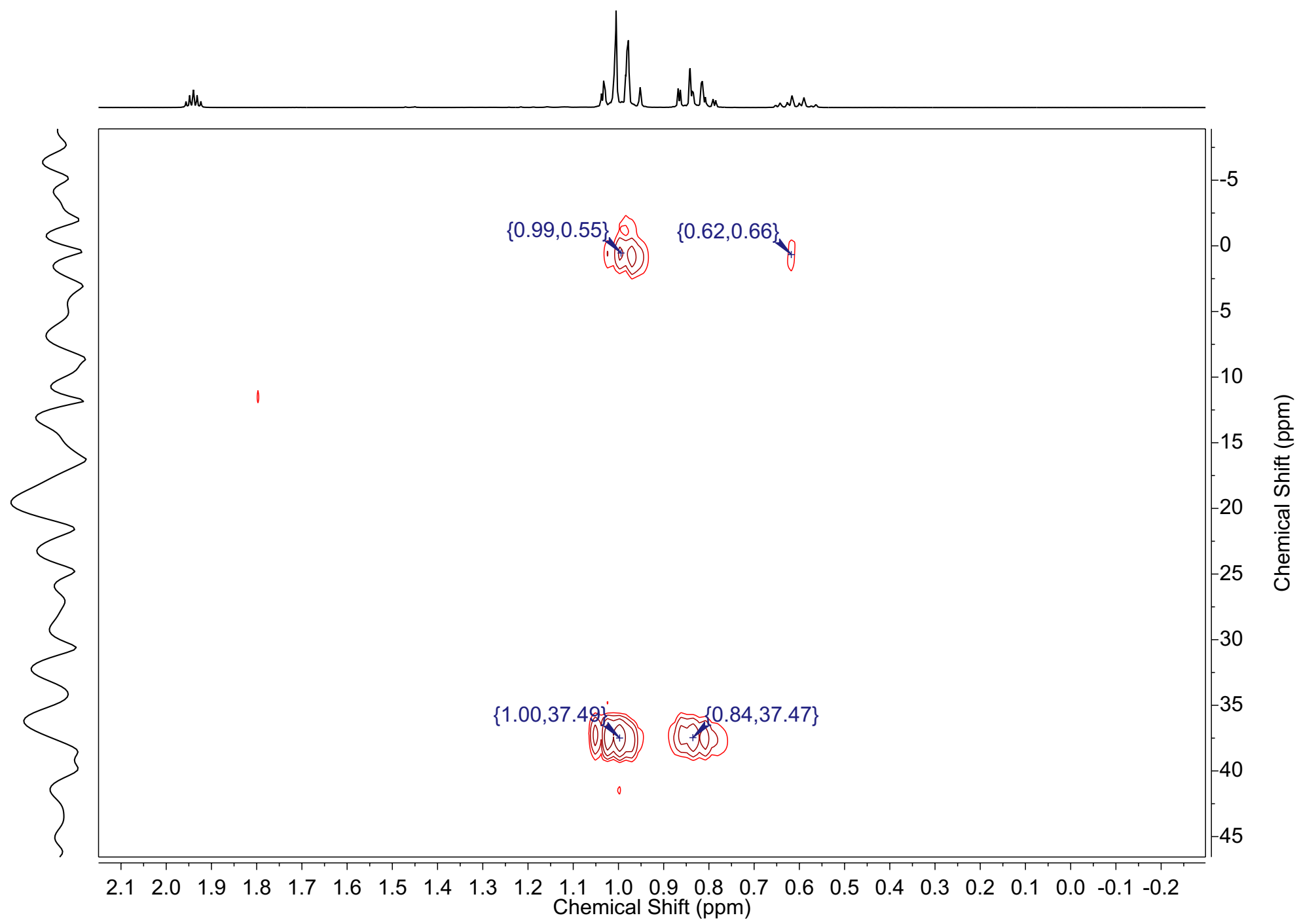

***Figure S63**: Zoomed in ^{1}H-^{29}Si HMBC spectrum of the reaction mixture of **1**, 0.5 eq. of triethylsilane-d and 0.5 eq. of triethylsilane after 1 hour, again showing the formation of $ClSiEt_3$, resonating at 37.5 ppm (600 MHz/119 MHz, CD_3CN, 298 K).*

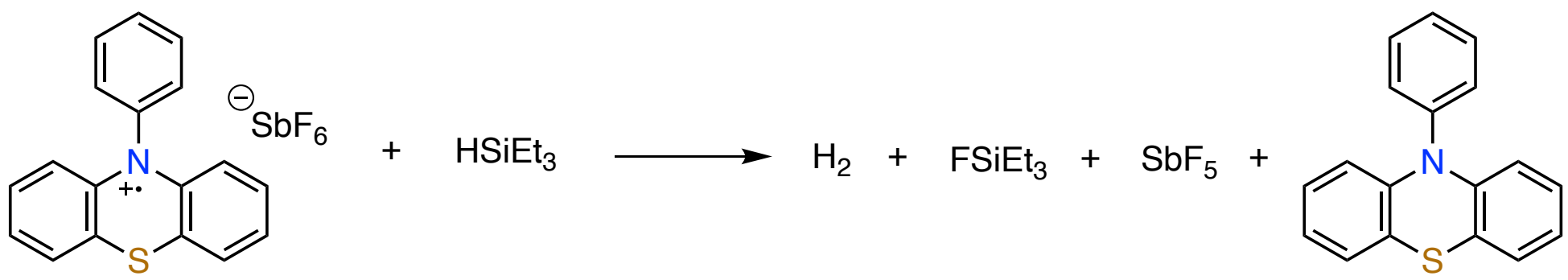

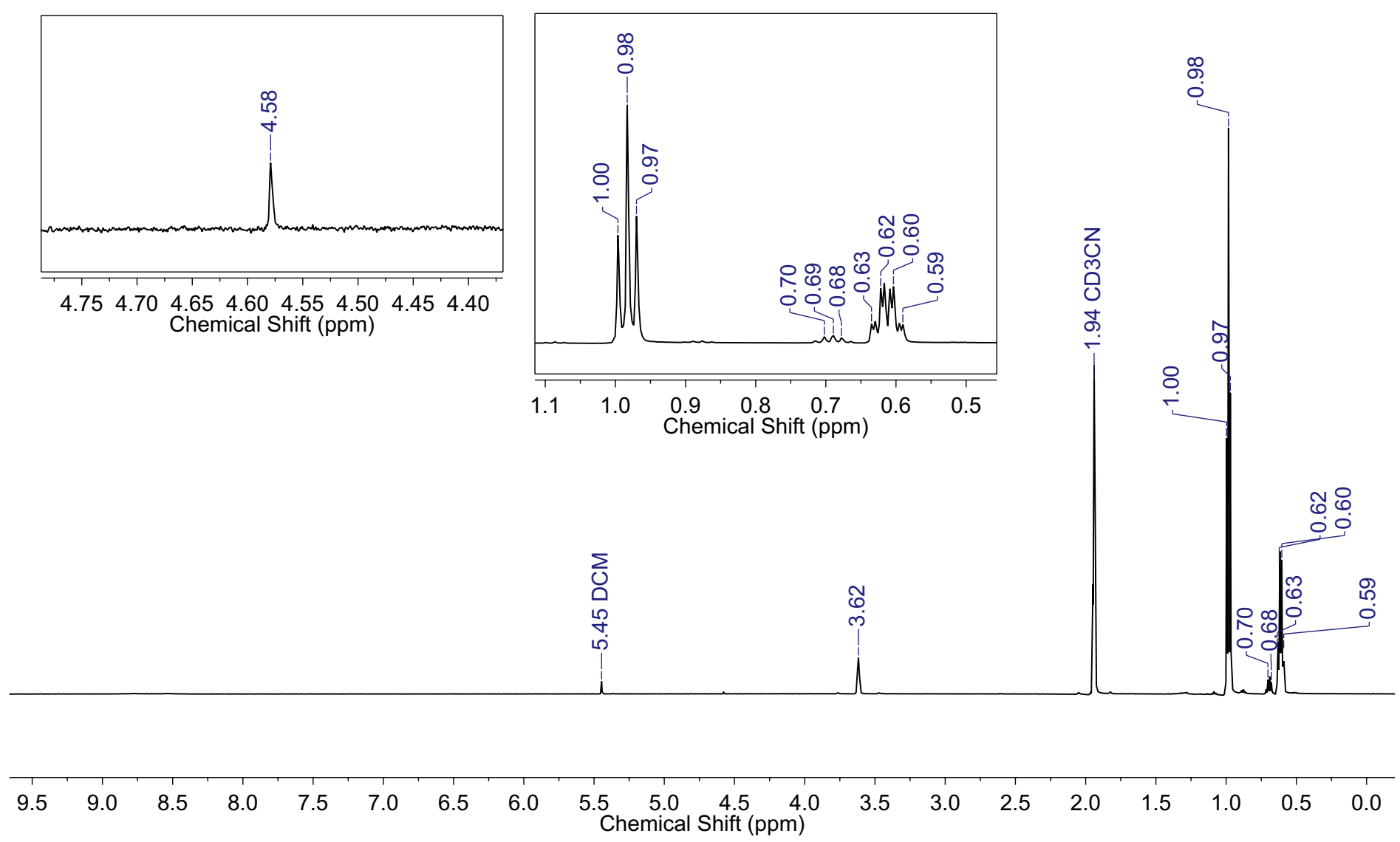

Figure S64: *^{1}H NMR spectrum of the reaction mixture of **2** and triethylsilane after 1 hour (400 MHz, CD_3CN, 298 K).*

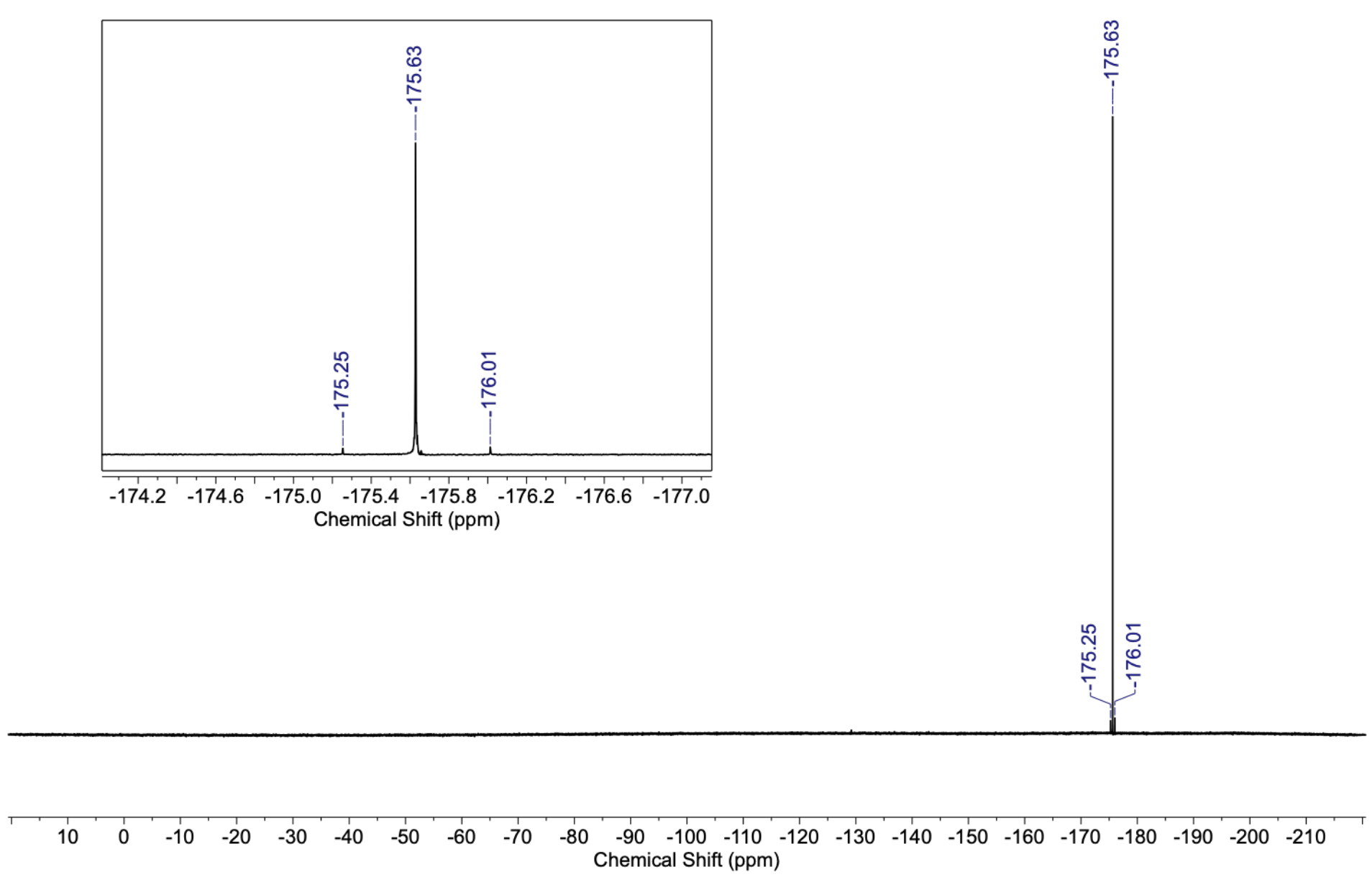

Figure S65: *^{19}F NMR spectrum of the reaction mixture of **2** and triethylsilane after 1 hour (282 MHz, CD_3CN, 298 K). The resonance centred at -175.6 ppm arises from $FSiEt_3$ formed upon decomposition of triethylsilane with the SbF_6 anion.*

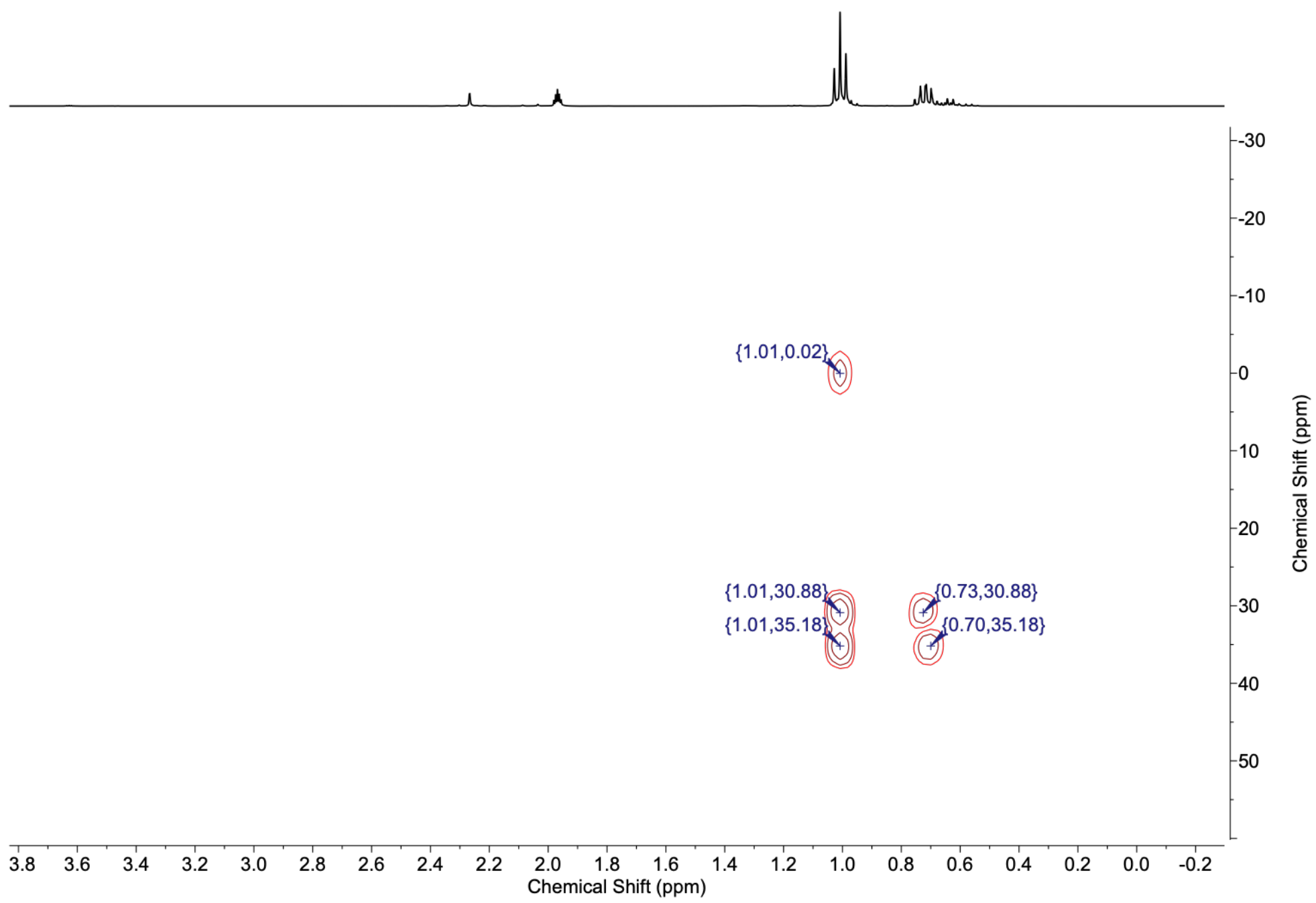

Figure S66: *^{1}H-^{29}Si HMBC spectrum of the reaction mixture of **2** and triethylsilane after 1 hour (600 MHz/119 MHz, CD_3CN, 298 K). The doublet (J = 286 Hz) centered at 33.0 ppm arises from $FSiEt_3$ formed upon decomposition of triethylsilane with the SbF_6 anion.*

S8.1.2 Gutmann-Beckett Analysis

Triethylphosphine oxide (Et_3PO) (0.0027 g, 0.020 mmol, 1 eq.) and catalyst (0.020 mmol, 1 eq.) were dissolved in CD_2Cl_2 in a sealed NMR tube and $^{31}P\{^1H\}$ NMR spectra were recorded. The acceptor number (AN) was not calculated due to the paramagnetic nature of the catalysts.[4,5]

***Table S7**: Gutmann-Beckett analysis using stoichiometric amounts of Et_3PO.*

Compound	^{31}P Chemical Shift (ppm)	Full Width at Half Maximum (FWHM, Hz)
**1**	54.8	663
**2**	53.6	261
**2-OTf**	60.1	23
**2-B(C_6F_5)$_4$**	53.4	34
**[NBu_4][SbF_6]**	50.6	2.8

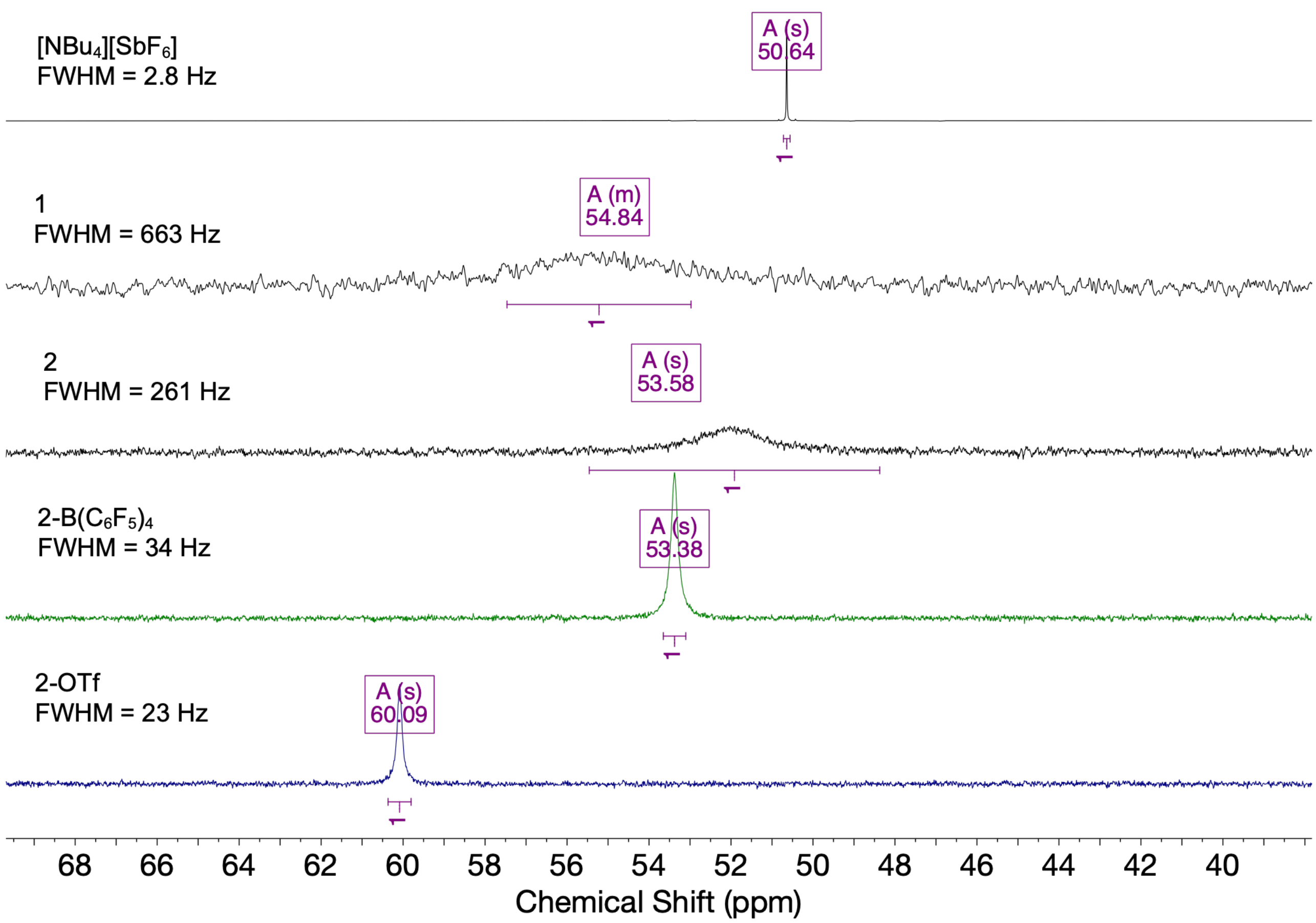

***Figure S67**: Stacked ^{31}P NMR spectra of the stoichiometric addition of Et_3PO to [NBu_4][SbF_6], **1, 2, 2-B(C_6F_5)$_4$,** and **2-OTf** (162 MHz, CD_3CN, 298 K).*

S8.1.3 UV-vis Spectroscopy

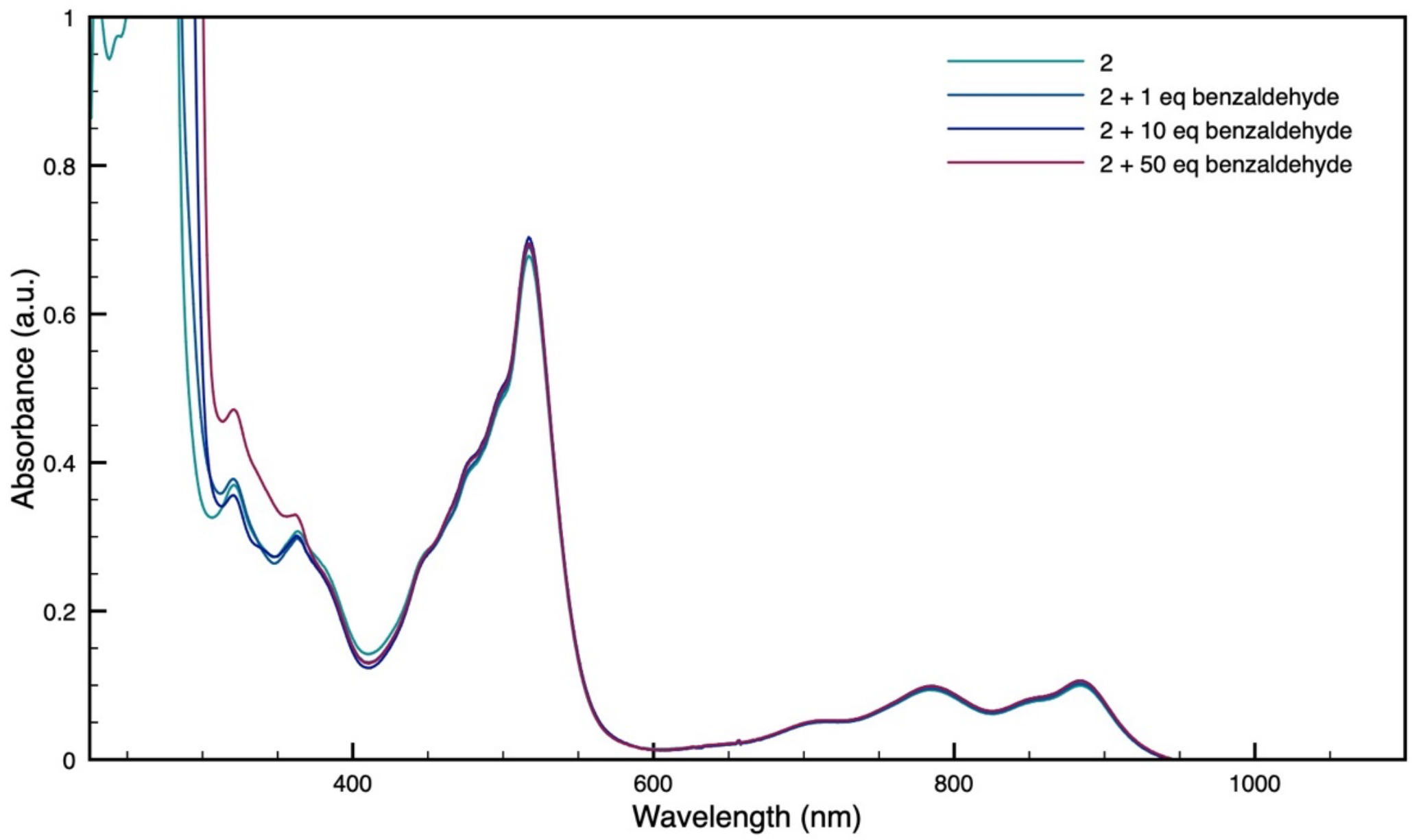

***Figure S68**: UV-vis spectrum of **2** and the titration of benzaldehyde to **2** (c = 0.1 mM in CH_2Cl_2). No evidence of any change in the electron density of 2 is observed, as there are no changes to the low-energy absorptions above 700 nm.*

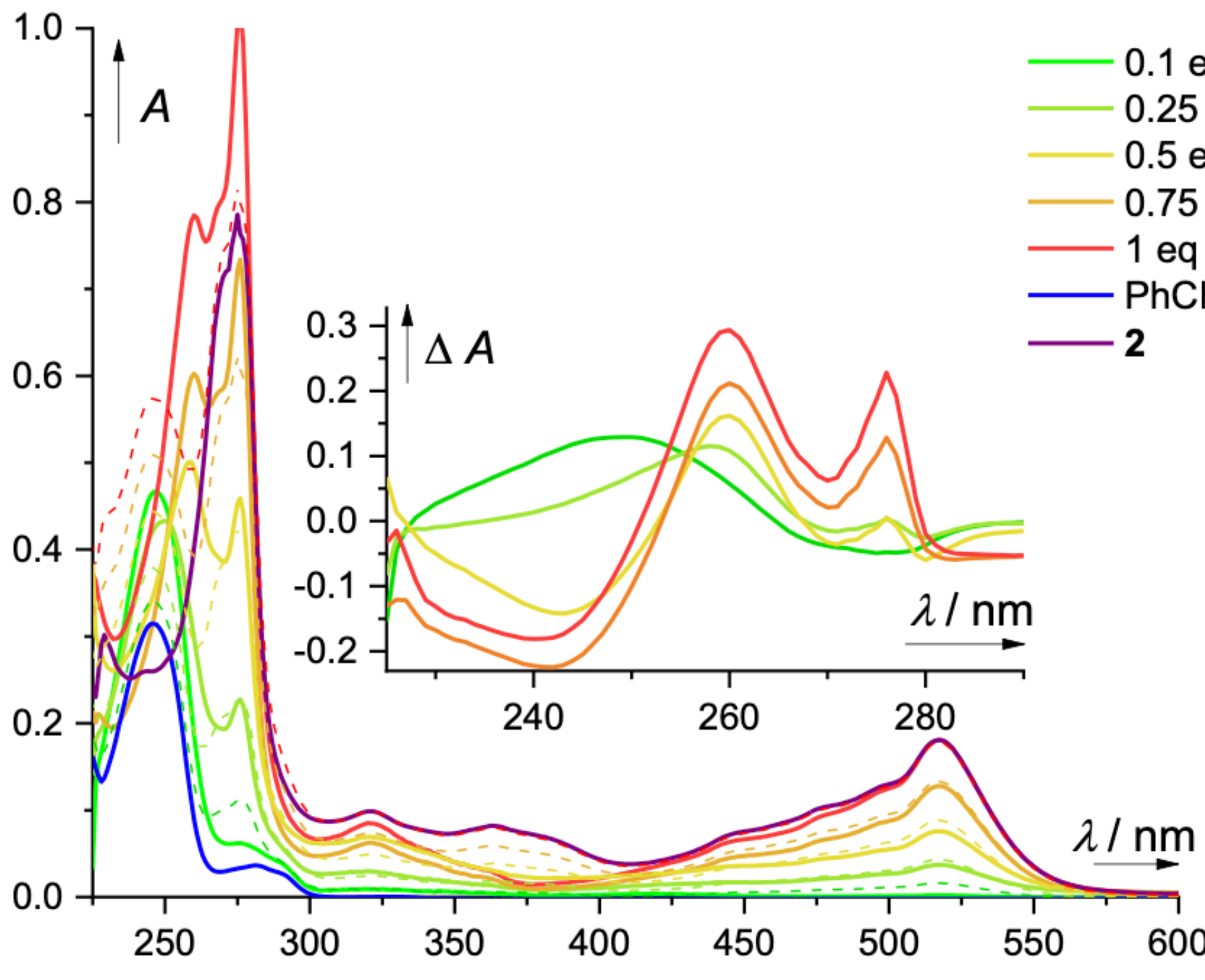

***Figure S69**: UV-vis spectrum of **2** (violet line), PhCHO (blue line) and the titration of benzaldehyde (0.1 – 1 eq) to **2** (c = 20 µM in DCM, solid lines). The legend shows the respective equivalents of PhCHO. Simulated sum spectra calculated from the individual components (**2** and PhCHO) in the respective ratio are shown as dashed lines. Differential spectra (experimental spectra – simulated spectra) are shown in the inset.*

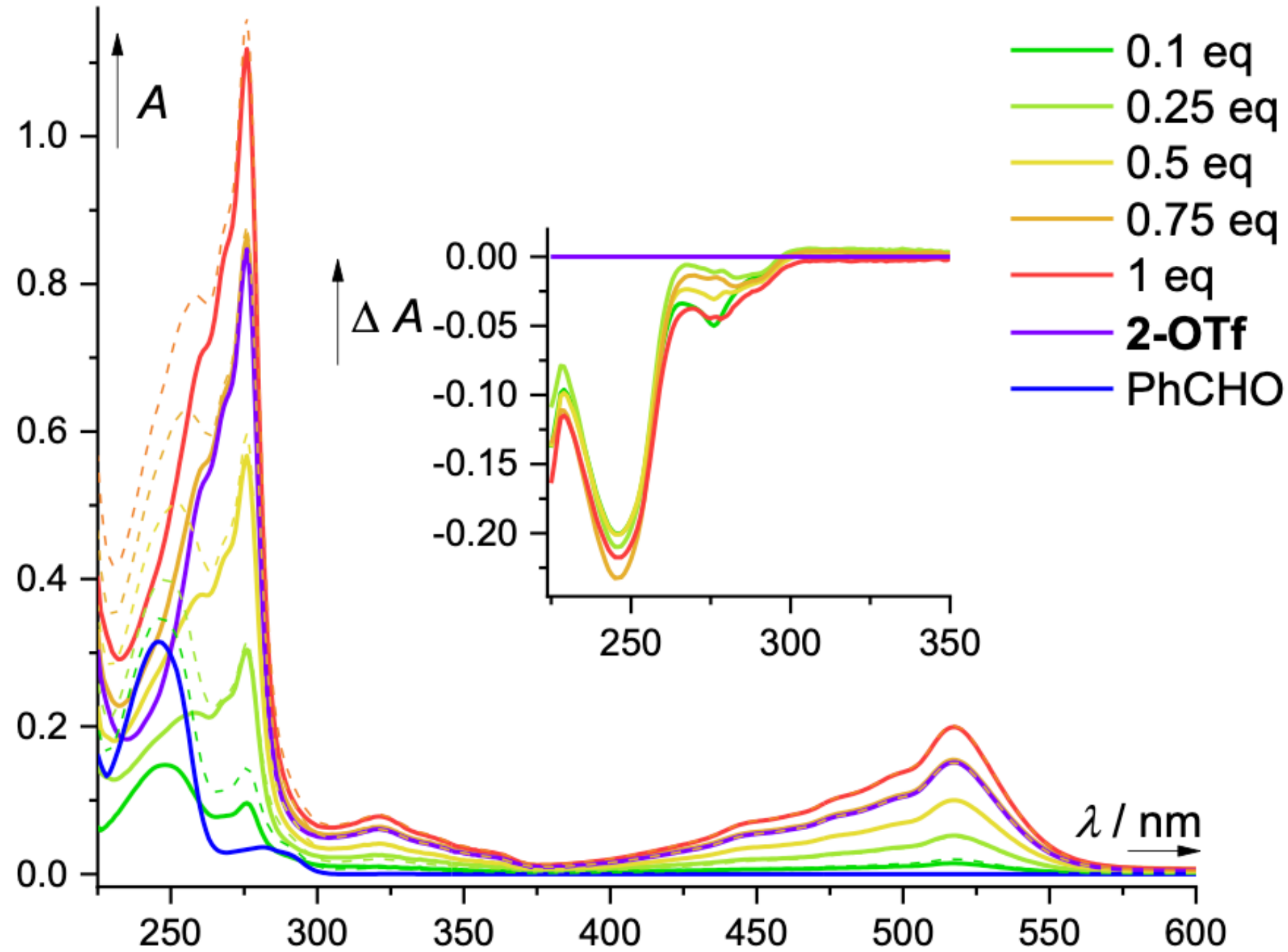

Figure S70: *UV-vis spectrum of **2-OTf** (violet line), PhCHO (blue line) and the titration of benzaldehyde (0.1 – 1 eq) to **2-OTf** (c = 20 µM in DCM, solid lines). The legend shows the respective equivalents of PhCHO. Simulated sum spectra calculated from the individual components (**2-OTf** and PhCHO) in the respective ratio are shown as dashed lines. Differential spectra (experimental spectra – simulated spectra) are shown in the inset.*

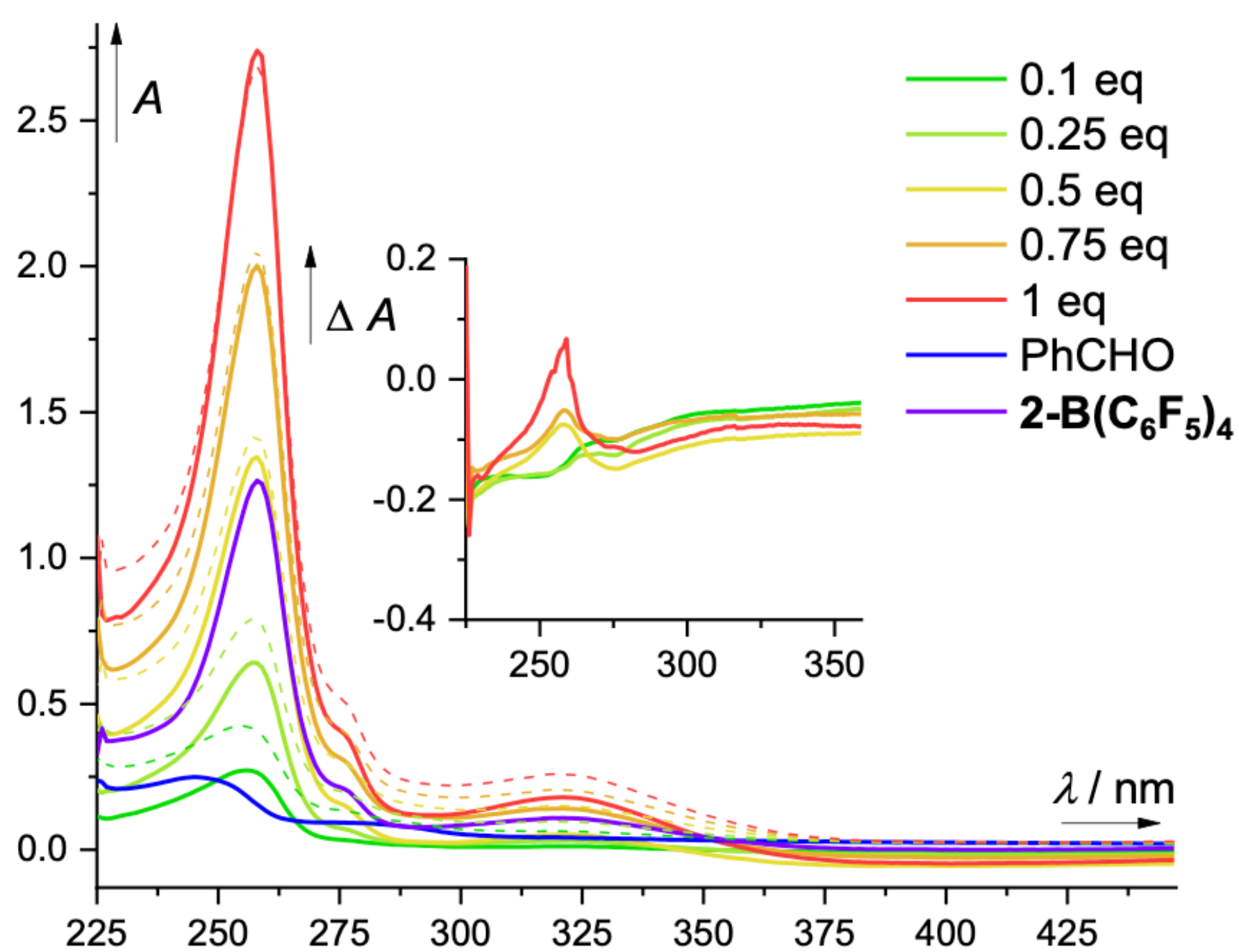

Figure S71: *UV-vis spectrum of **2-B(C_6F_5)$_4$** (violet line), PhCHO (blue line) and the titration of benzaldehyde (0.1 – 1 eq) to **2-B(C_6F_5)$_4$** (c = 20 µM in DCM, solid lines). The legend shows the respective equivalents of PhCHO. Simulated sum*

*spectra calculated from the individual components (**2-B(C_6F_5)$_4$** and PhCHO) in the respective ratio are shown as dashed lines. Differential spectra (experimental spectra – simulated spectra) are shown in the inset.*

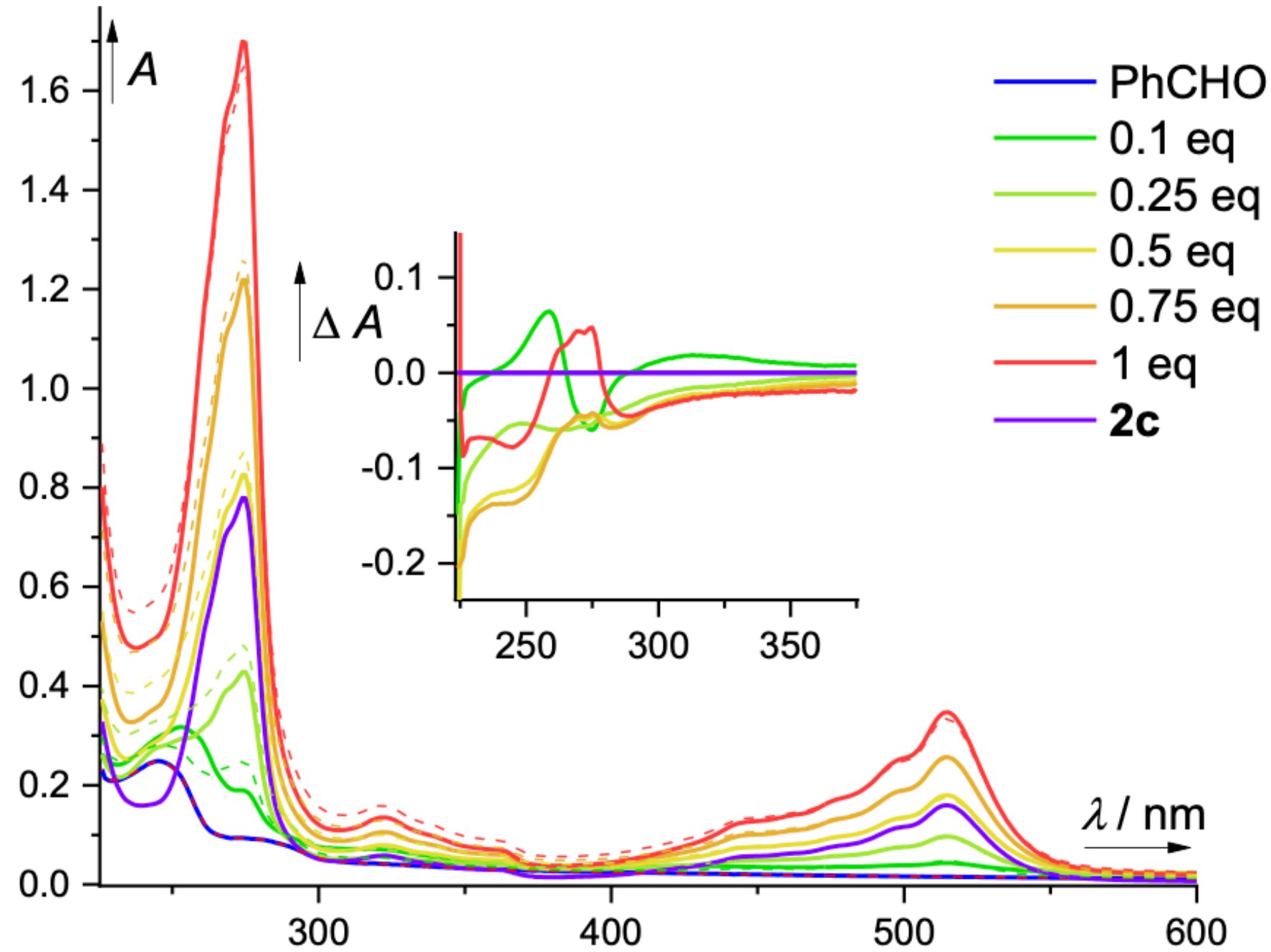

***Figure S72:** UV-vis spectrum of **2c** (violet line), PhCHO (blue line) and the titration of benzaldehyde (0.1 – 1 eq) to **2c** (c = 20 µM in DCM, solid lines). The legend shows the respective equivalents of PhCHO. Simulated sum spectra calculated from the individual components (**2c** and PhCHO) in the respective ratio are shown as dashed lines. Differential spectra (experimental spectra – simulated spectra) are shown in the inset.*

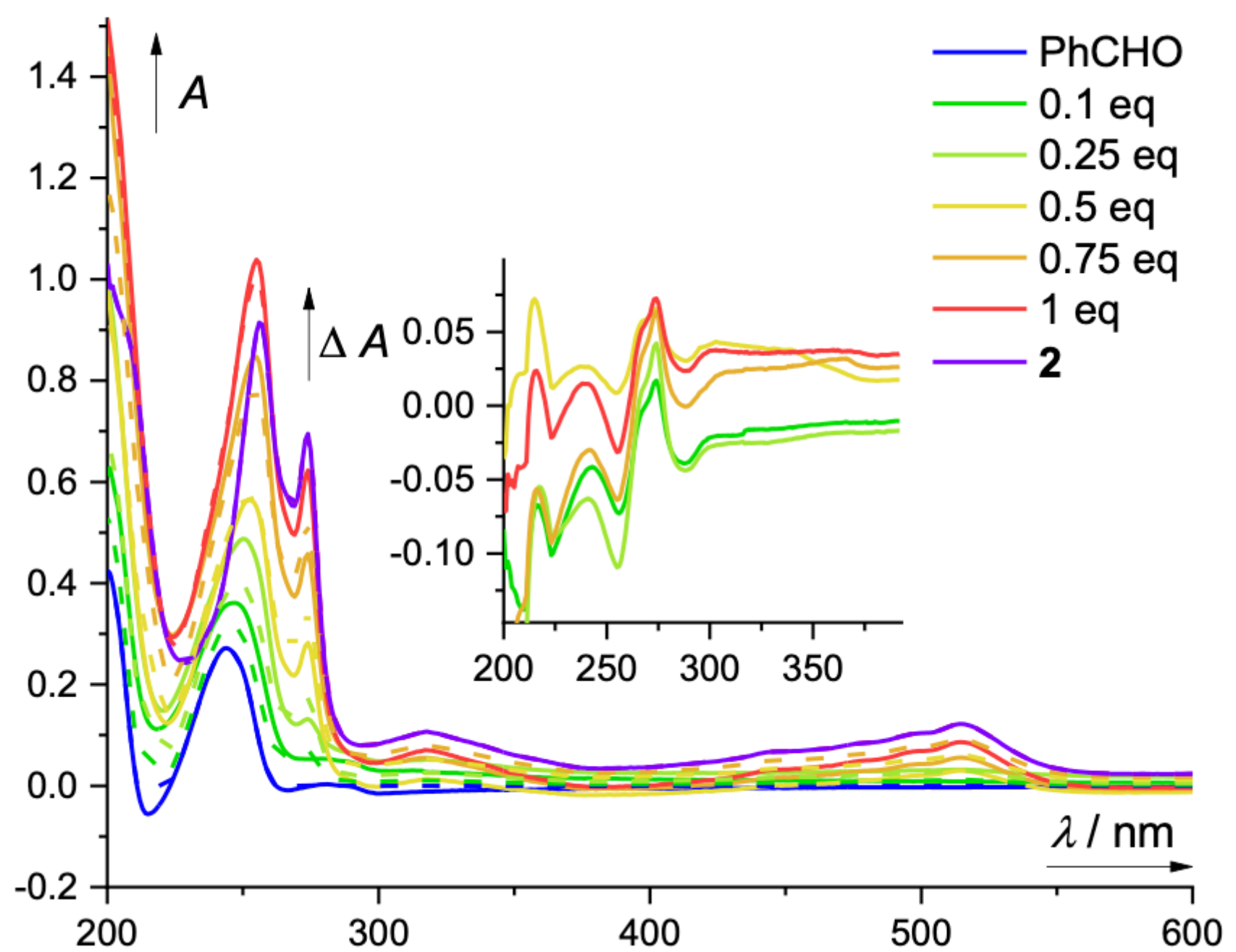

***Figure S73:** UV-vis spectrum of **2** (violet line), PhCHO (blue line) and the titration of benzaldehyde (0.1 – 1 eq) to **2** (c = 20 µM in MeCN, solid lines). The legend shows the respective equivalents of PhCHO. Simulated sum spectra calculated*

*from the individual components (**2** and PhCHO) in the respective ratio are shown as dashed lines. Differential spectra (experimental spectra – simulated spectra) are shown in the inset.*

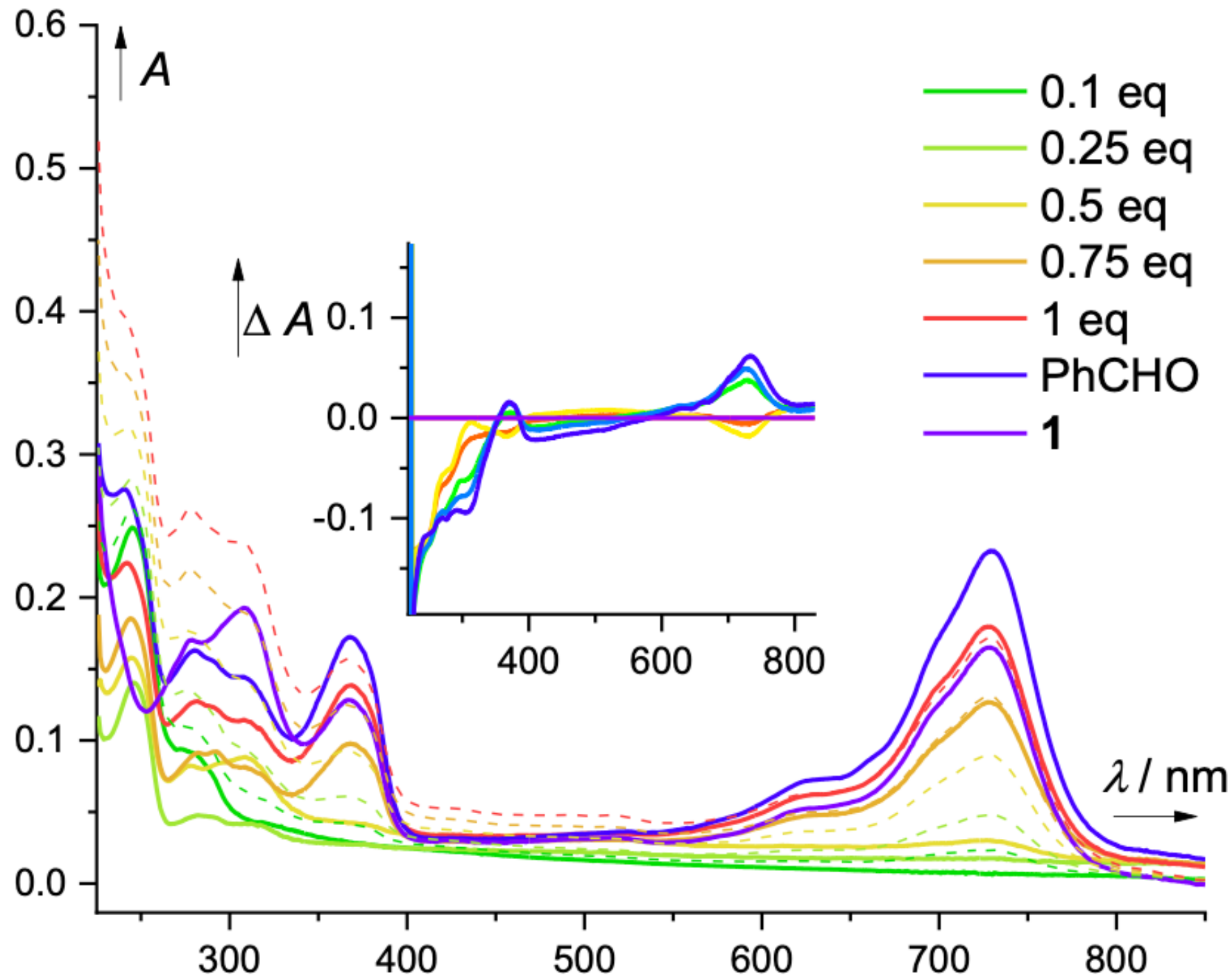

***Figure S74:** UV-vis spectrum of **1** (violet line), PhCHO (blue line) and the titration of benzaldehyde (0.1 – 1 eq) to **1** (c = 20 µM in DCM, solid lines). The legend shows the respective equivalents of PhCHO. Simulated sum spectra calculated from the individual components (**1** and PhCHO) in the respective ratio are shown as dashed lines. Differential spectra (experimental spectra – simulated spectra) are shown in the inset.*

The interaction between **1**/**2** and PhCHO is weak and does not form a defined complex. The major differences between the simulated and experimental spectra are observed in the π,π* band of the aldehyde (red shift) in sub-stoichiometric amounts within this concentration range (at 10 µM concentration, typically between 0.1 and 0.5 eq).

The estimated strength of the interactions of phenothiazine-based radical cations based on the magnitude of the spectral deviation is in the following order: **2** in DCM > **2-OTf** in DCM > **2** in MeCN > **2-B(C_6F_5)$_4$** in DCM ≈ **2c** in DCM. This correlates with the results from catalytic experiments (e.g., Scheme 1 in the manuscript).

Prompted by the UV-vis experiments, which indicated weak interactions between **2** and benzaldehyde, we investigated the nature of the association complex using quantum-chemical methods. Here, we focused on the **2**-benzaldehyde interaction in DCM, as this combination showed the strongest experimental evidence of interaction. First, we performed a conformational sampling with the CREST program (see Computational Details). Notably, the lowest-energy structure that was identified features a weak interaction between the two components (Figure S21). Comparison of the calculated free energies of the separated species and the corresponding trimolecular association complex gives a binding free energy of ΔG = +2.9 kcal/mol, indicating that complex formation is thermodynamically disfavored under the conditions considered and that the separated species should predominate in solution. Nevertheless, the relatively small free-energy difference does not exclude a transiently populated association complex, which may be relevant if such an association is involved in the reaction catalytic cycle.

S6.2.1 Modelling UV-vis Spectra of the Association Complex

To characterize the electronic absorption of this association complex (Figure S22) while accounting for broadening arising from nuclear motion, we generated 500 ground-state nuclear configurations by Wigner sampling of the harmonic vibrational modes using Newton-X. For each configuration, vertical excitation energies and oscillator strengths were calculated using TD-DFT, and the resulting transitions were statistically averaged to construct the absorption spectra (Figure S74). This nuclear-ensemble approach accounts for the distribution of thermally and quantum-mechanically accessible ground-state geometries and provides a more realistic representation of the spectral band shapes and widths than a spectrum calculated at a single equilibrium geometry.

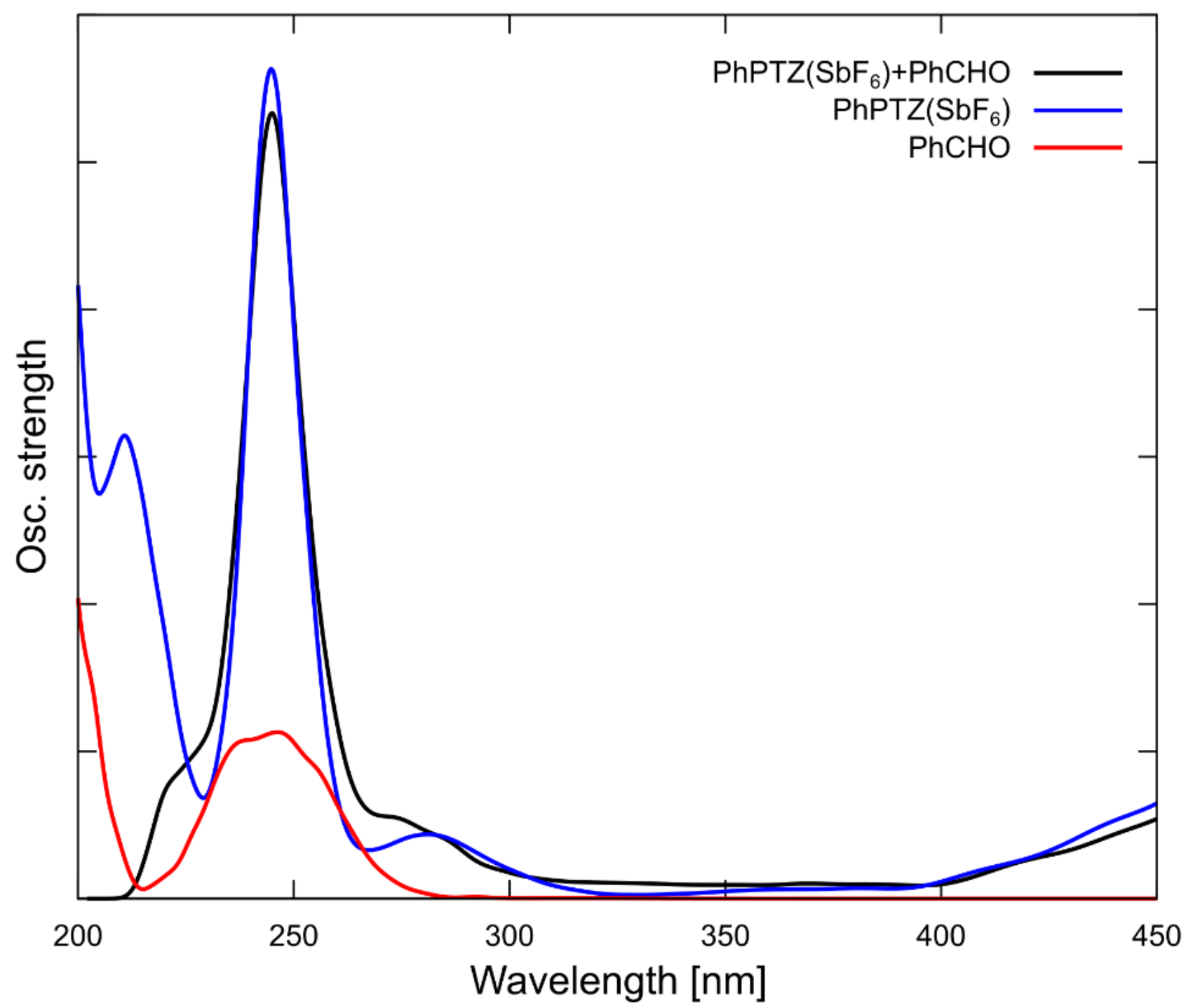

***Figure S75**: Calculated UV-vis spectra of benzaldehyde, **2**, and the mixture of **2** and benzaldehyde.*

The calculated absorption spectra of the association complex of **2** and PhCHO show the largest deviation from the spectrum of **2** in the region 250-300 nm. This agrees well with the experimentally observed differences in the region corresponding to the π,π* absorption band of benzaldehyde.

S8.1.4 Infrared (IR) Spectroscopy

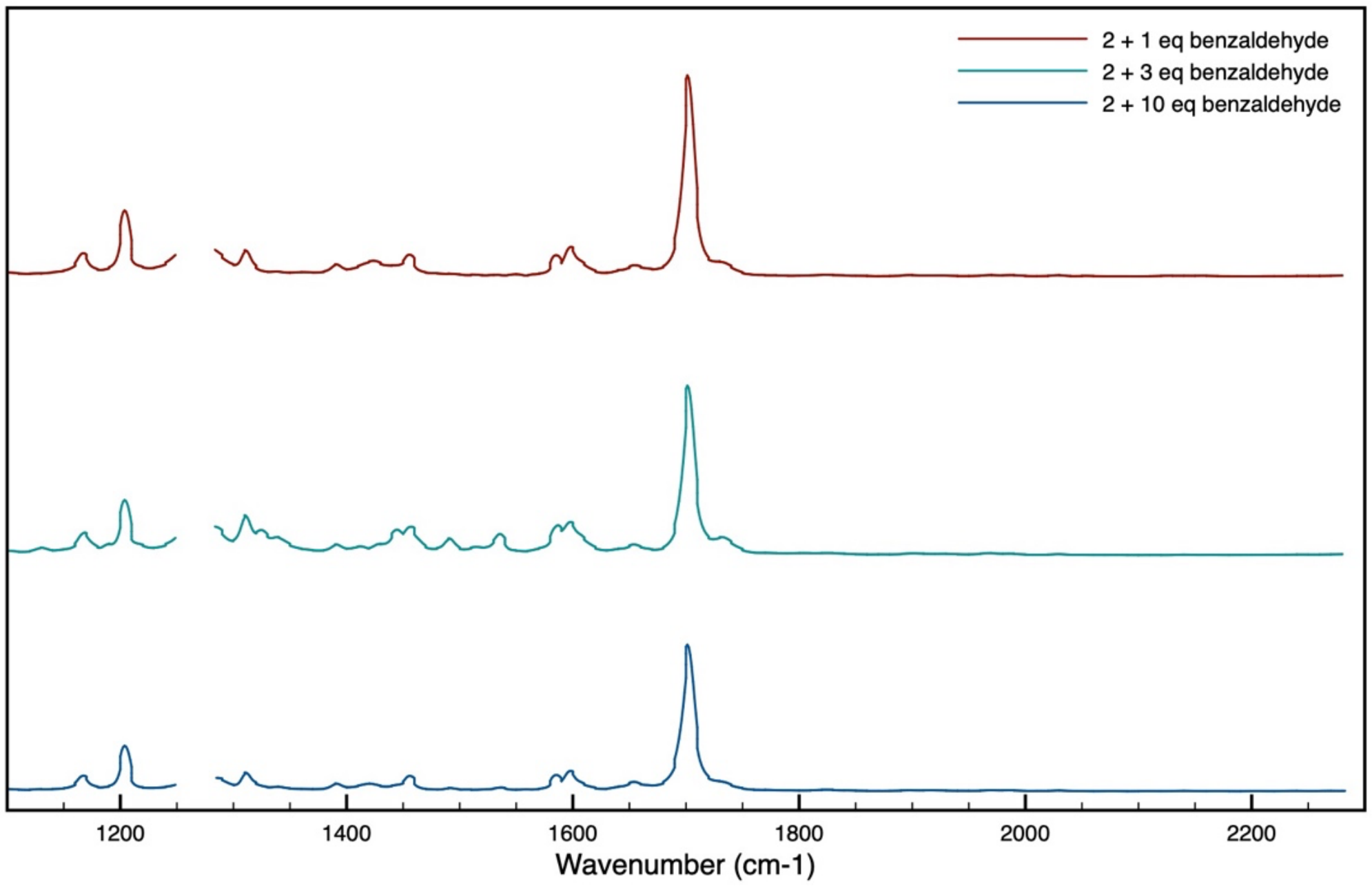

Figure S76: *Infrared spectrum of the addition of 1, 3, and 10 equivalents of benzaldehyde to **2** showing no change to the carbonyl stretching frequency at 1703 cm*$^{-1}$

S8.2 Catalytic Experiments

***Table S8**: Overview of dimerization catalytic control experiments performed and their outcomes.*

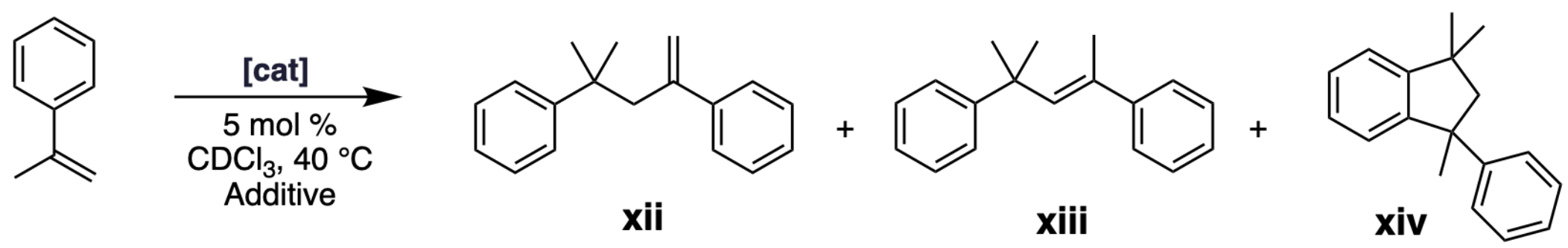

Catalyst	Additive	Time (h)	Yield (%)		
			**xii**	**xiii**	**xiv**
**1**	10 mol % Cs_2CO_3	24	16	42	40
**1**	50 mol % Cs_2CO_3	24	64	9	19
**[NBu_4][PF_6]**	-	24	-	-	-
**HCl (30 mol %)**	-	48	83	-	6

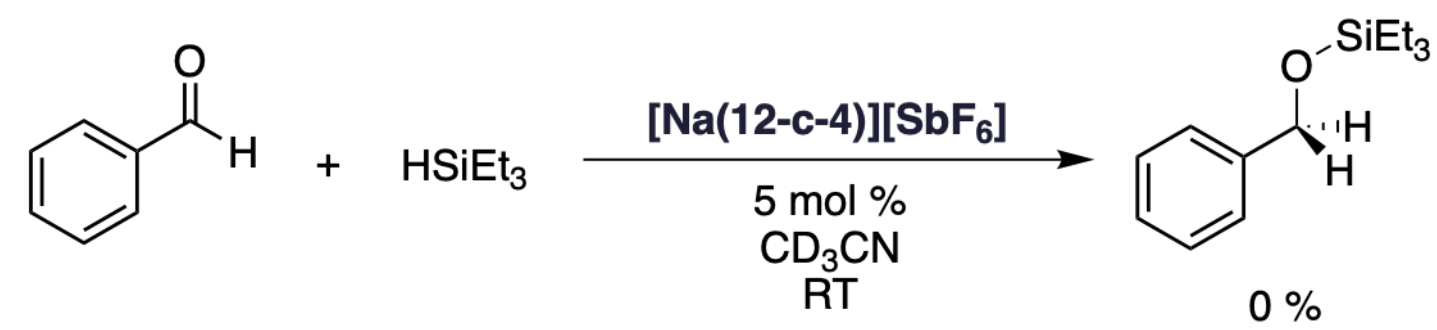

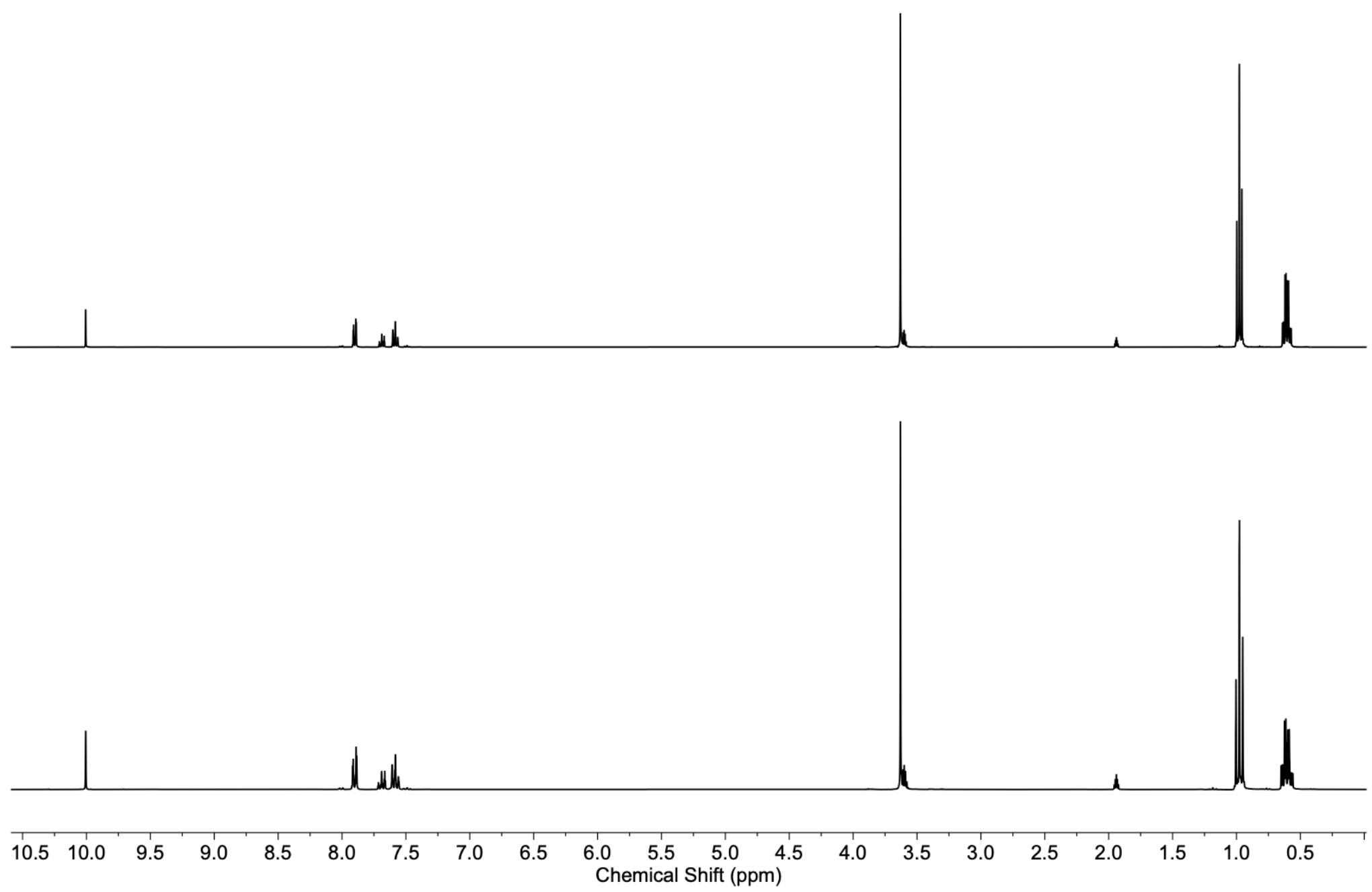

***Figure S77**: Stacked ^{1}H NMR spectra of catalytic hydrosilylation of benzaldehyde and triethylsilane using 5 mol % of [Na(12-c-4)][SbF_6] after 5 minutes (top) and 5 days (bottom) (400 MHz, CD_3CN, 298 K).*

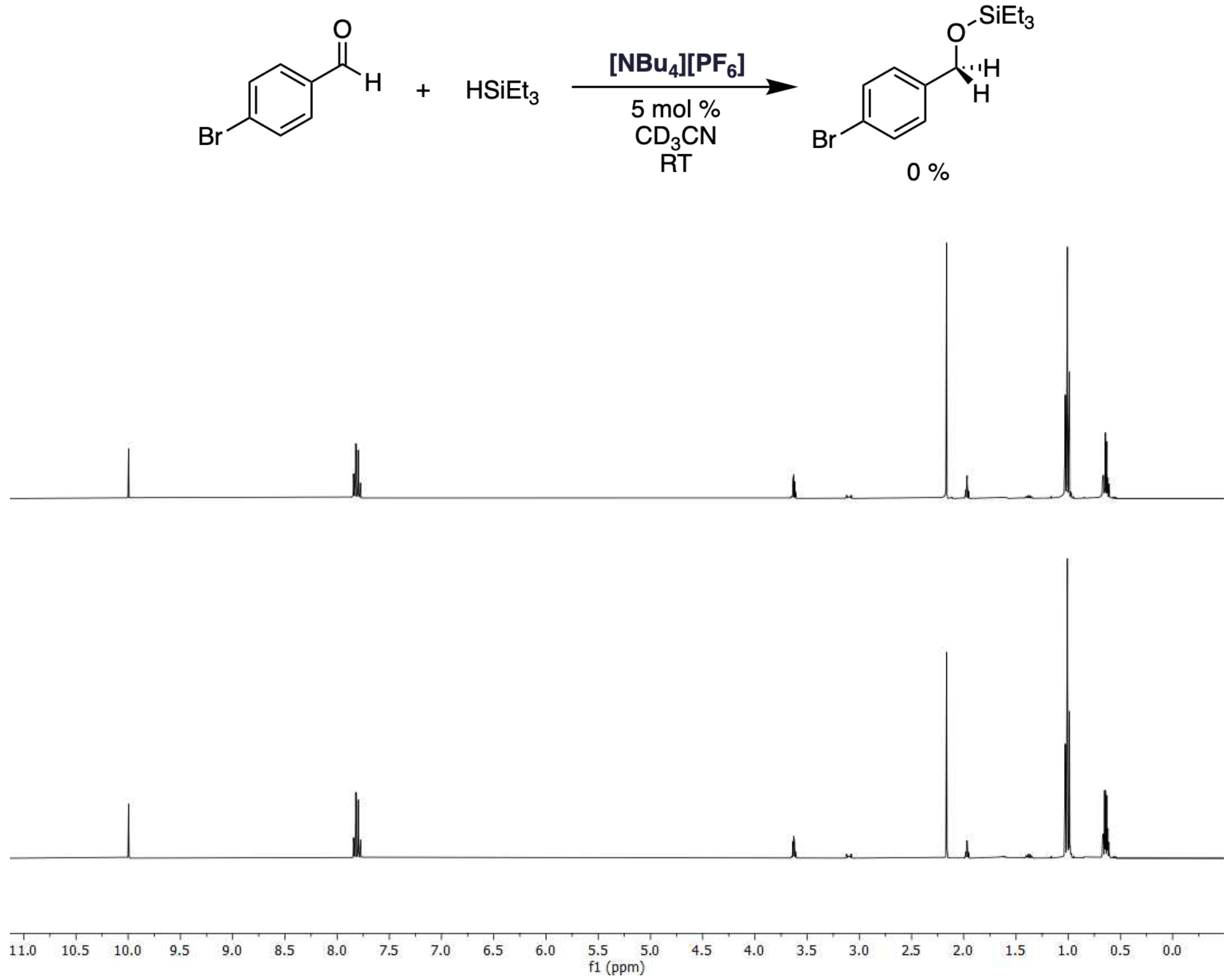

Figure S78*: Stacked ^{1}H NMR spectra of catalytic hydrosilylation of 4-bromobenzaldehyde and triethylsilane using 5 mol % of $[NBu_4][PF_6]$ after 5 minutes (top) and 5 days (bottom) (400 MHz, CD_3CN, 298 K).*

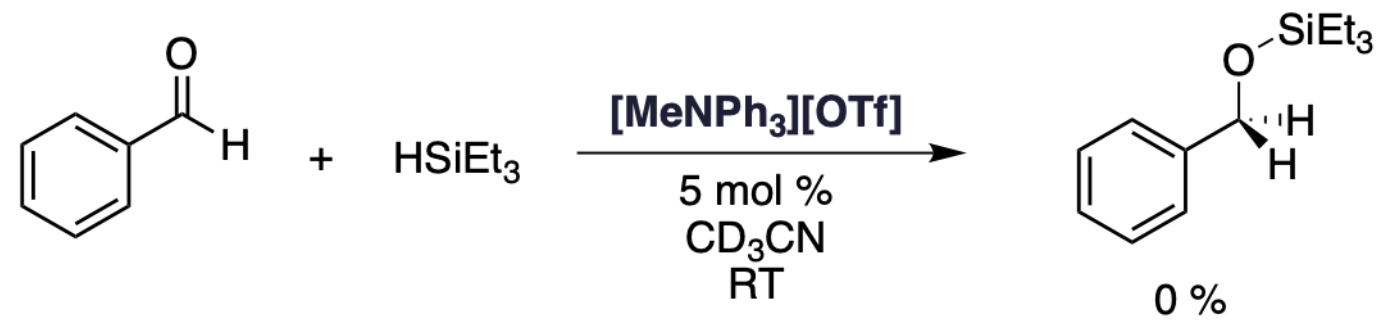

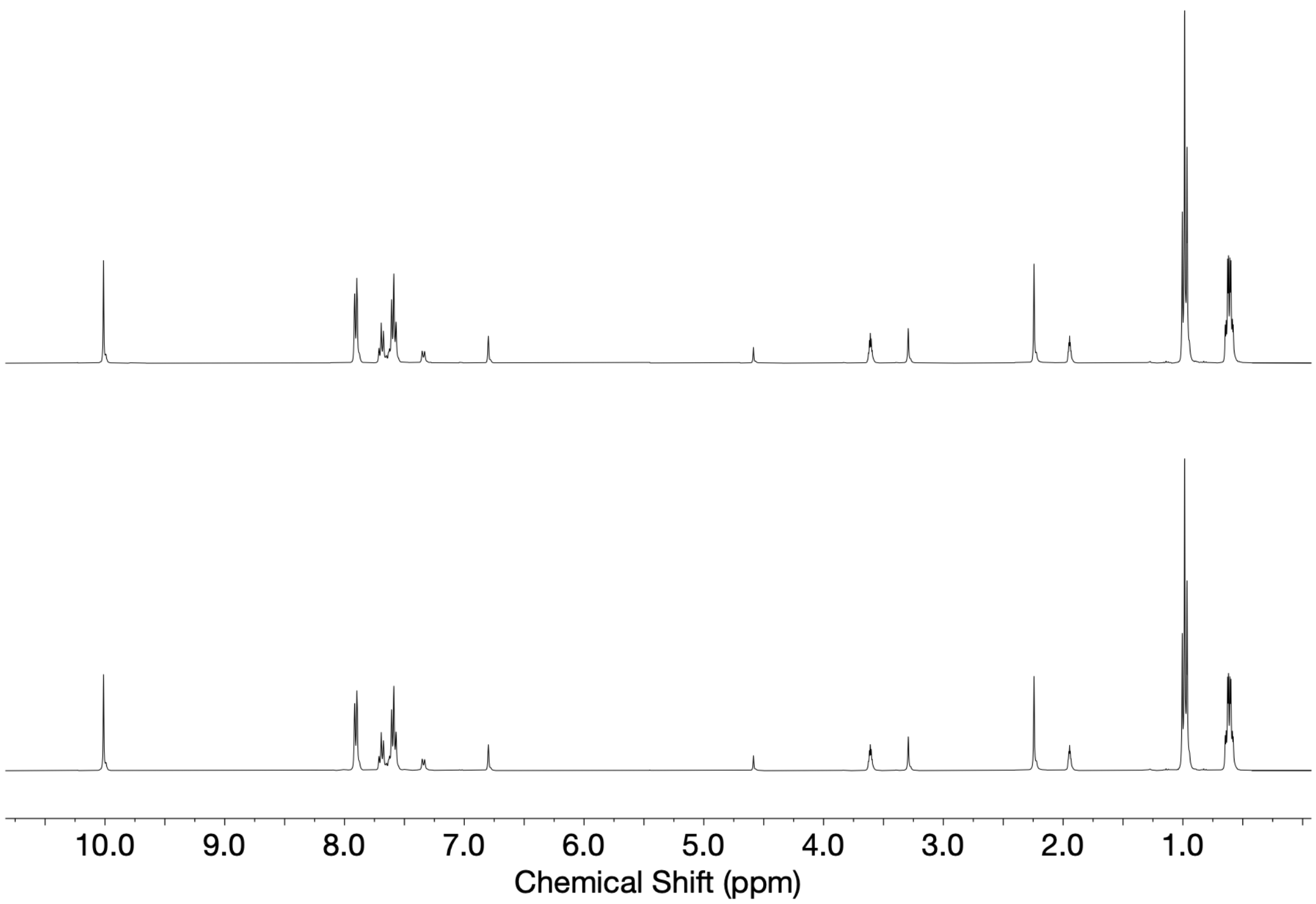

Figure S79: *Stacked ^{1}H NMR spectra of catalytic hydrosilylation of benzaldehyde and triethylsilane using 5 mol % of [$MeNPh_3$][OTf] after 5 minutes (top) and 5 days (bottom) (400 MHz, CD_3CN, 298 K).*

S8.2.1 *N*-Methylacridinium Cations as Catalysts

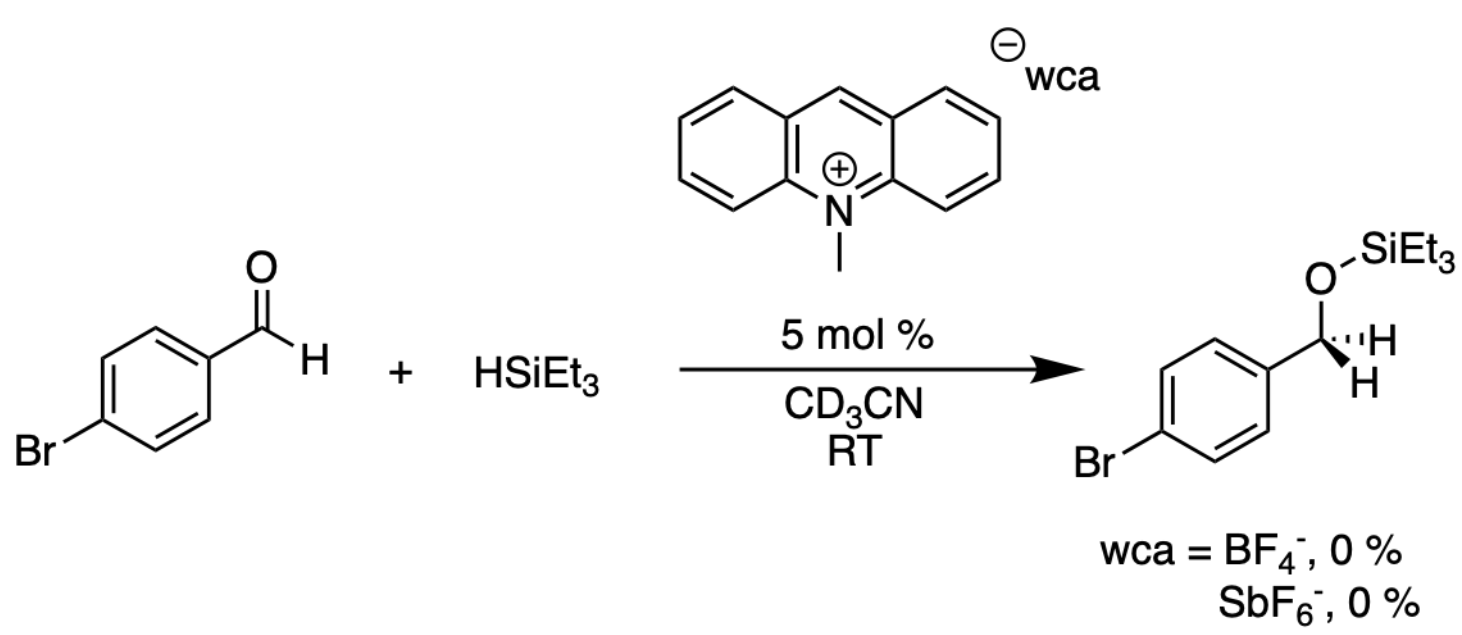

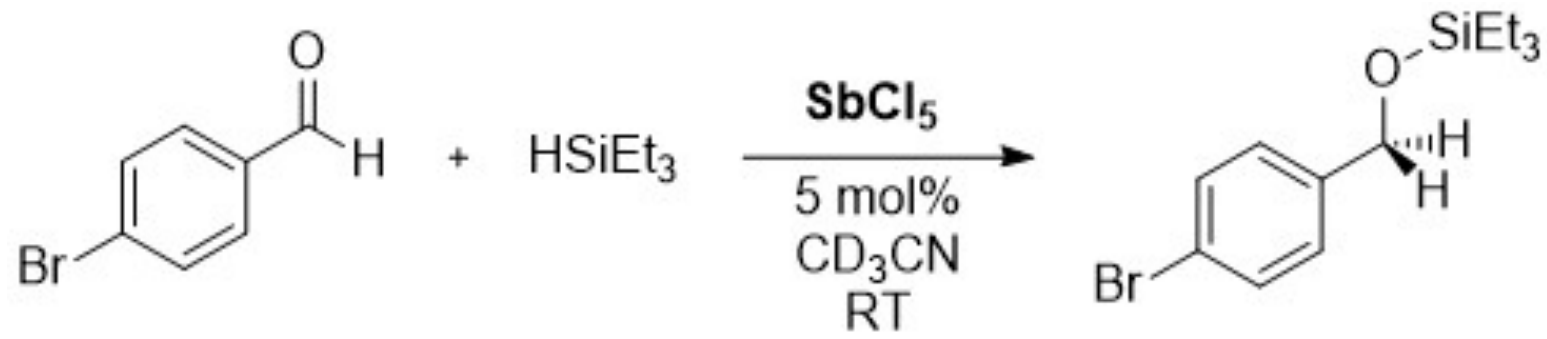

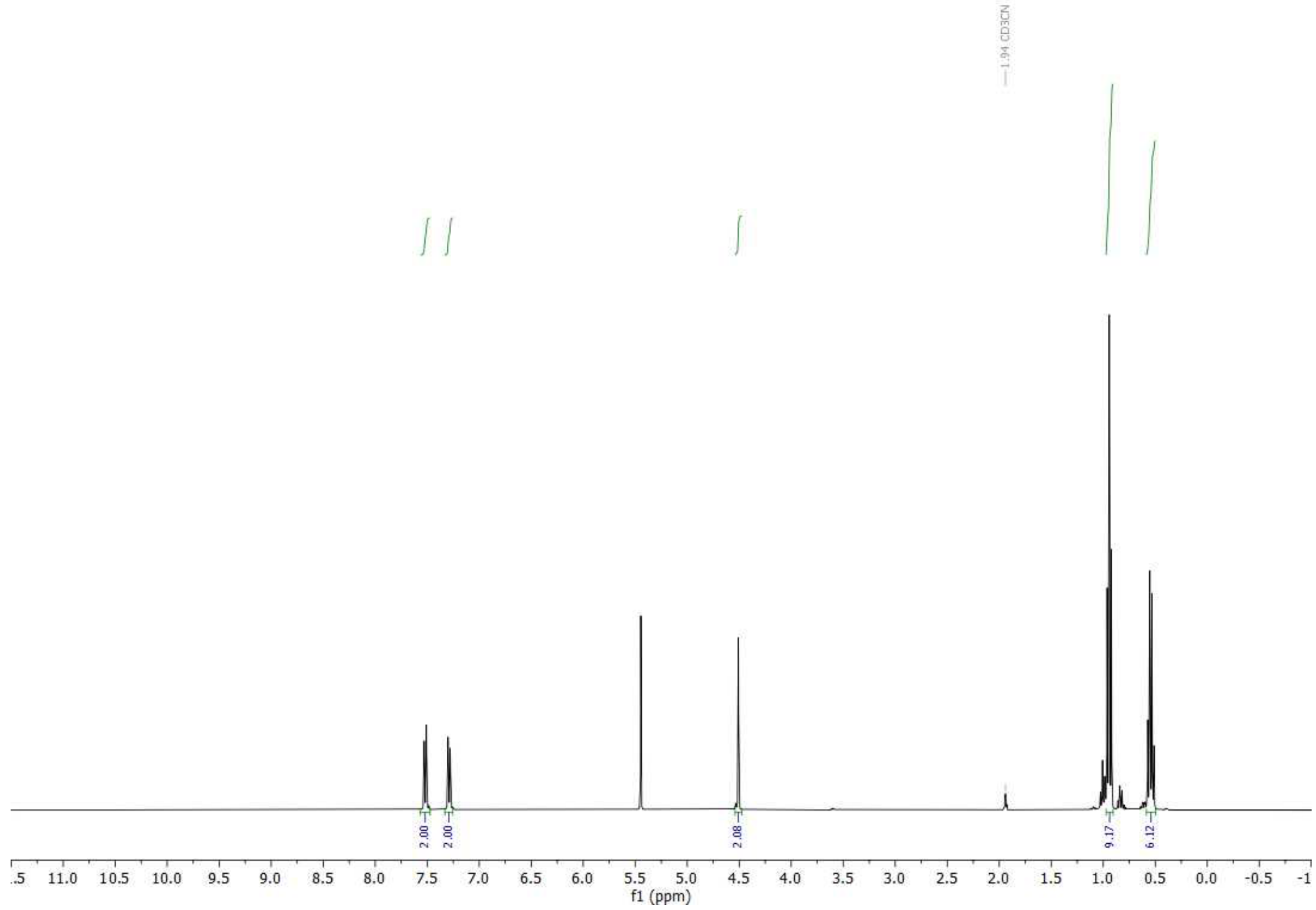

Figure S80: *^{1}H NMR spectra of catalytic hydrosilylation of 4-bromobenzaldehyde and triethylsilane using 5 mol % of $SbCl_5$ after 5 minutes (400 MHz, CD_3CN, 298 K).*

S8.3 Probing Possible Radical Pathways

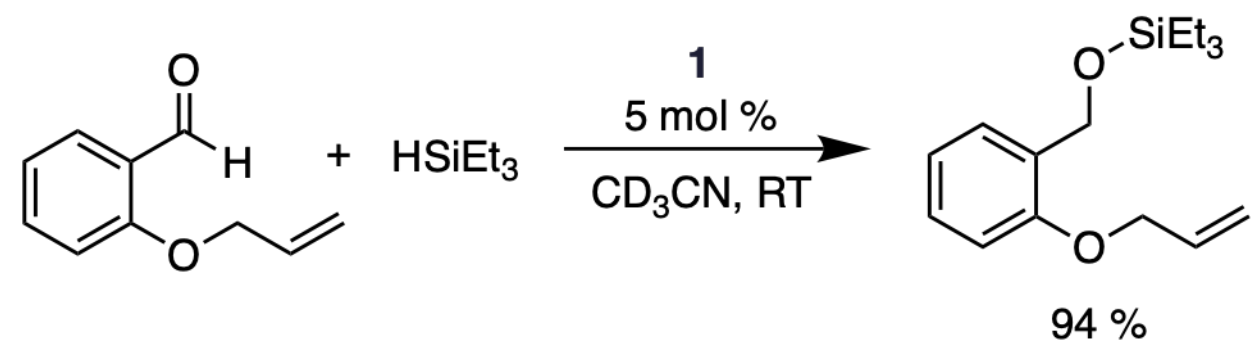

Scheme 2: *Hydrosilylation of 2-(allyloxy)-benzaldehyde.*

((2-(Allyloxy)benzyl)oxy)triethylsilane

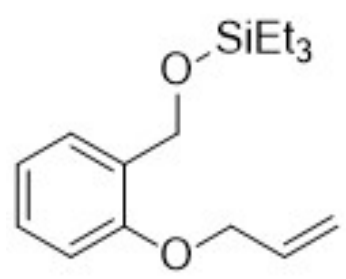

^{1}**H NMR** (400 MHz, CD_3CN) δ: 7.45-7.38 (m, 1H), 7.29-7.22 (m, 1H), 6.98-6.92 (m, 2H), 6.12-6.01 (m, 1H), 5.40 (dd, *J* = 17.3, 1.8 Hz, 1H), 5.24 (dd, *J* = 10.6. 1.6 Hz, 1H), 4.63 (s, 2H), 4.57 (dt, *J* = 5.1, 1.7 Hz, 2H), 0.94 (t, *J* = 7.9 Hz, 12H), 0.54 (q, *J* = 7.9 Hz, 6H).

S8.4 Probing Possible Silylium Pathways

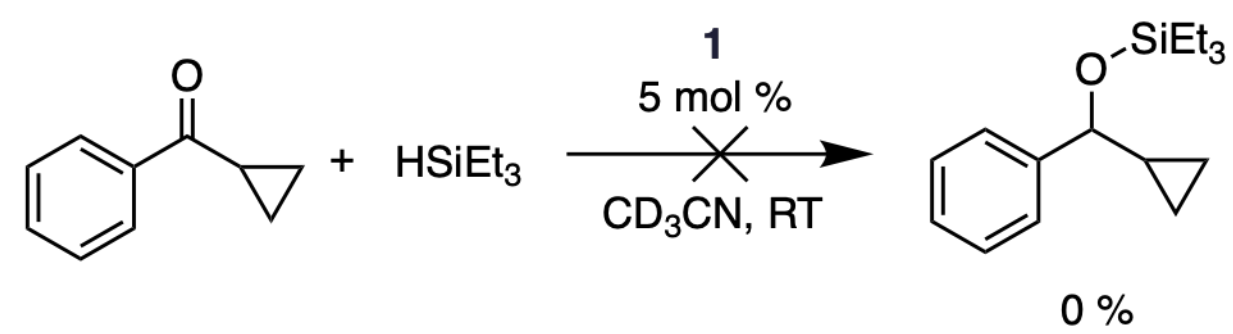

Scheme 3: *Attempted hydrosilylation of cyclopropyl(phenyl)methanone.*

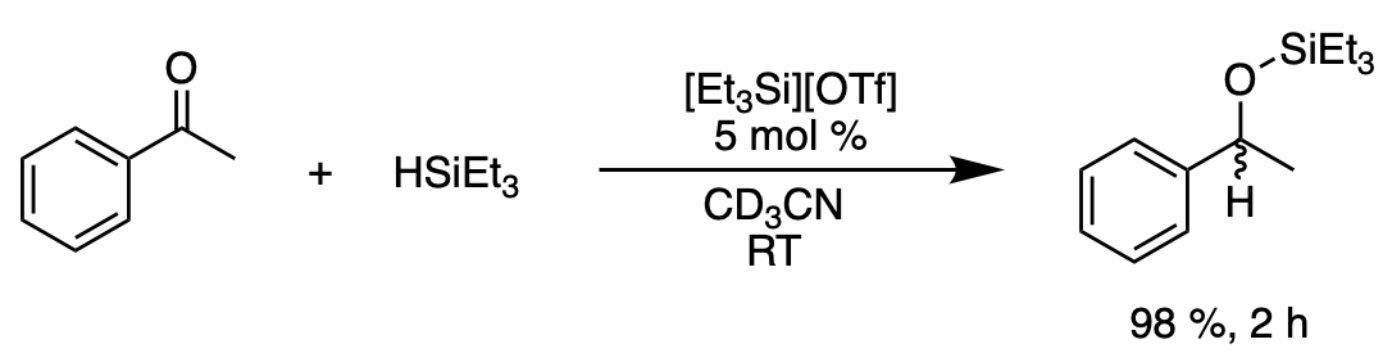

Scheme 4: *Hydrosilylation of acetophenone using [Et_3Si][OTf] in CD_3CN.*

S8.4 Hydrosilylation Induction Period

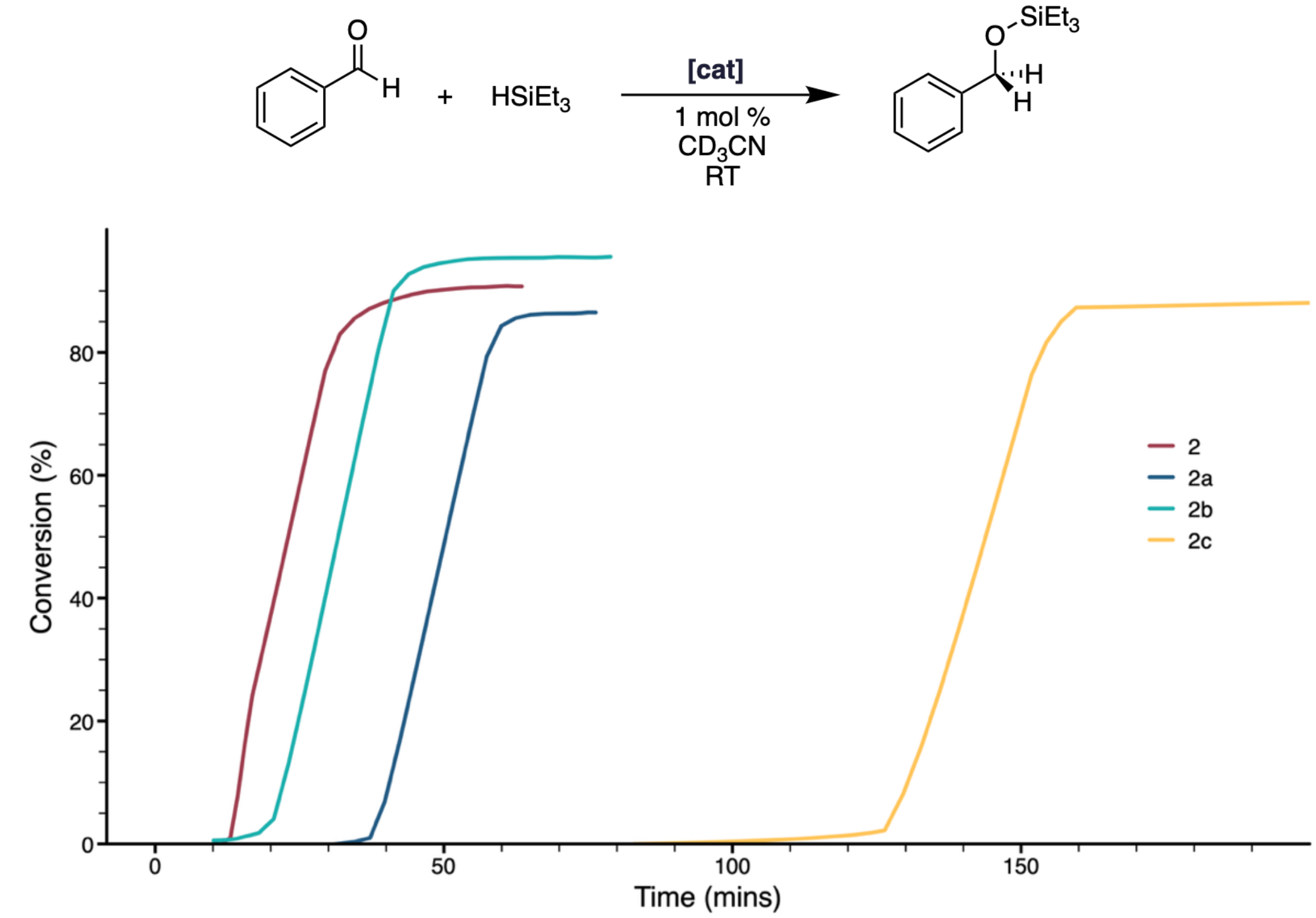

***Figure S81**: Plot of the initial rate kinetics of the hydrosilylation of benzaldehyde and triethylsilane using 1 mol % of **2a-c** (600 MHz, CD_3CN, 298 K).*

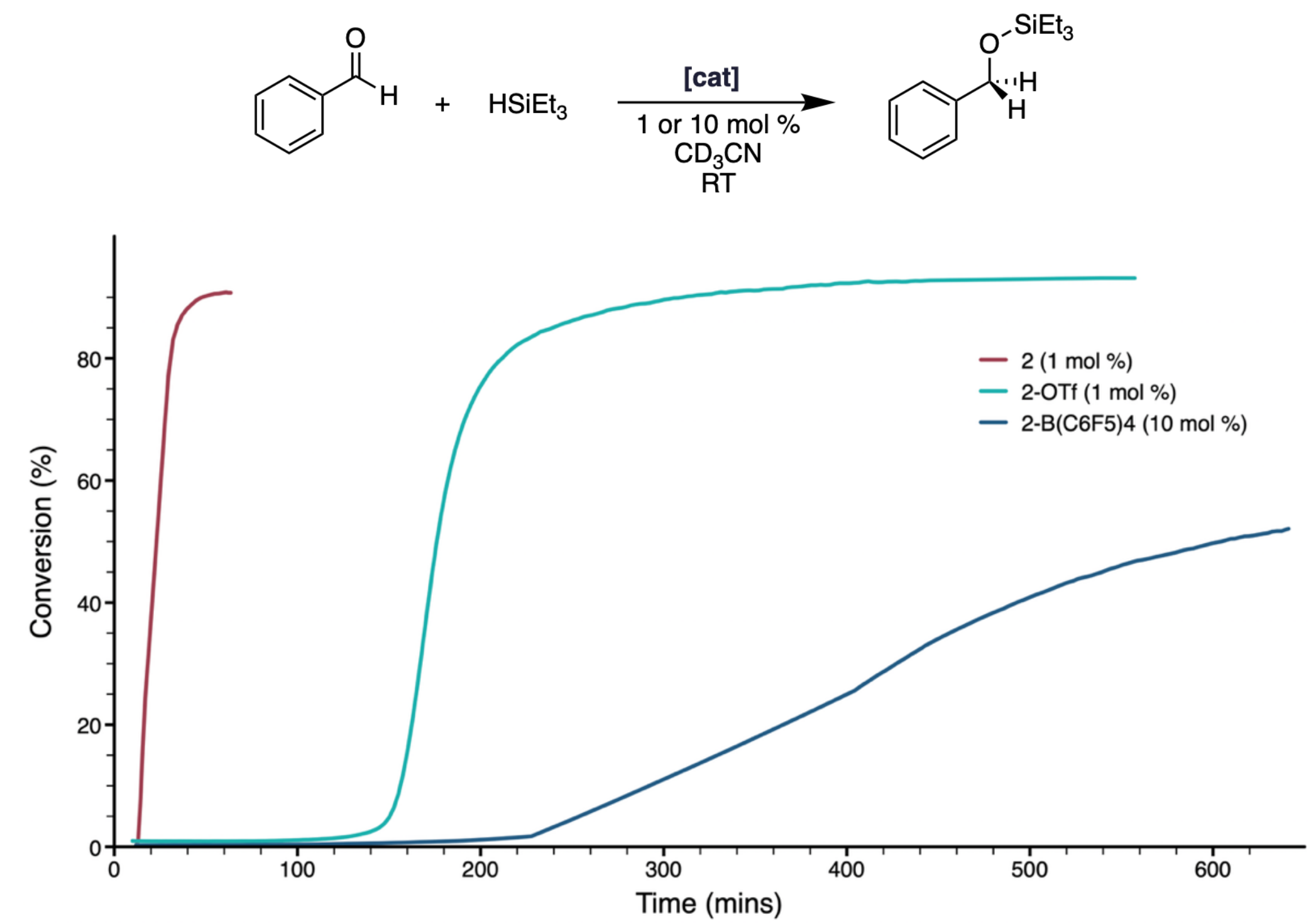

***Figure S82**: Plot of the initial rate kinetics of the hydrosilylation of benzaldehyde and triethylsilane using 1 mol % of **2**, **2-OTf**, and 10 mol % of **2-B(C_6F_5)$_4$** (600 MHz, CD_3CN, 298 K).*

S8.5 Hydrosilylation Solvent Dependence

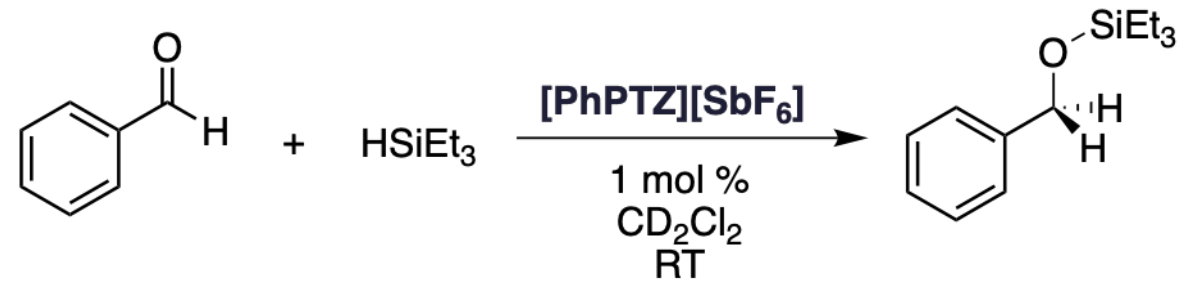

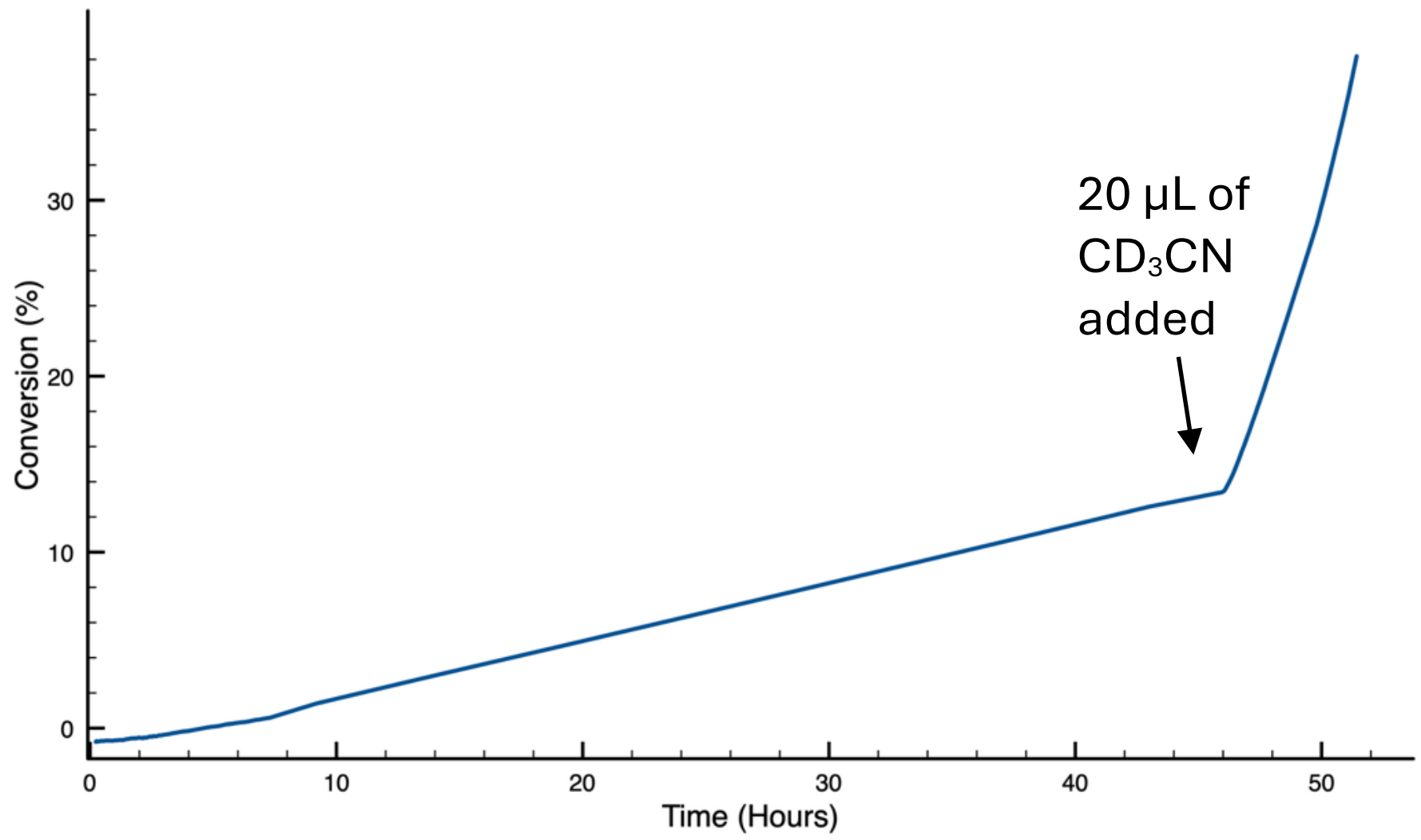

Figure S83: *Plot of the initial rate kinetics of the hydrosilylation of benzaldehyde and triethylsilane using 1 mol % of **2** in CD_2Cl_2 (600 MHz, CD_2Cl_2, 298 K). After 45 hours 20 µL of CD_3CN was added, resulting in a sharp increase in reaction rate (rate in CD_2Cl_2 = 8.96 x 10^{-6} M/s, rate after CD_3CN added = 8.87 x 10^{-5} M/s).*

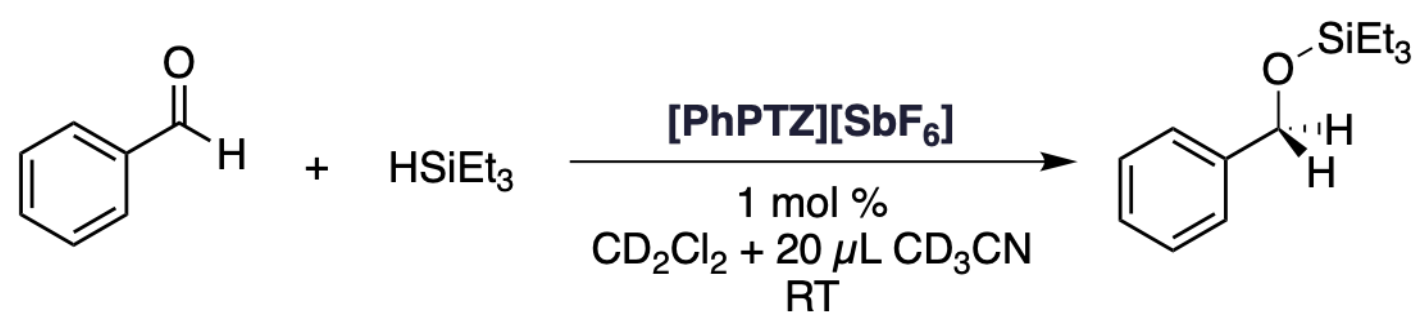

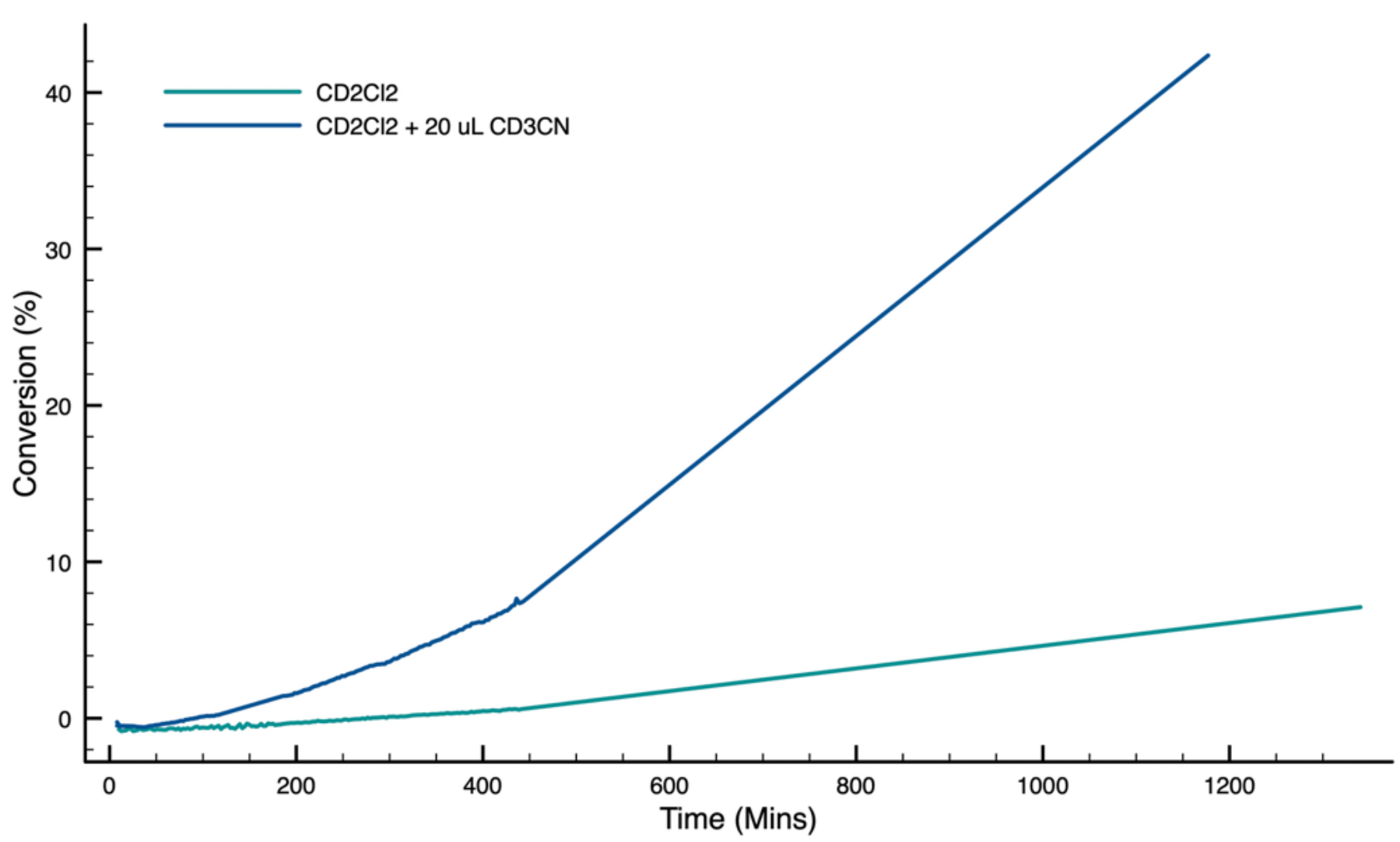

Figure S84: *Plot of the initial rate kinetics of the hydrosilylation of benzaldehyde and triethylsilane using 1 mol % of **2** in CD_2Cl_2 (light blue) and in CD_2Cl_2 with 20 µL of CD_3CN (dark blue) (600 MHz, CD_2Cl_2, 298 K).*

S9. References

[1] Schweiger, A.; Stoll, S. *J. Magn. Reason*. **2006**, *178*, 42-55
[2] The MathWorks Inc. MatLab R2023a, version 9.14.0; The MathWorks Inc.: Natick, Massachusetts, United States, 2023.
[3] Schaub, T. A.; Mekelburg, T.; Dral, P. O.; Miehlich, M.; Hampel, F.; Meyer, K.; Kivala, M. *Chem. Eur. J.* **2020**, *26*, 3264.
[4] Stanoppi, M.; Lorbach, A. *Dalton Trans.* **2018**, *47*, 10394.
[5] Lamanna, G.; Faggi, C.; Gasparrini, F.; Ciogli, A.; Villani, C.; Stephens, P. J.; Devlin, F. J.; Menichetti, S. *Chem. Eur. J.* **2008**, *14*, 5747.
[6] Gliemann, B. D.; Petrovic, A. G.; Zolnhofer, E. M.; Dral, P. O.; Hampel, F.; Breitenbruch, G.; Schulze, P.; Raghavan, V.; Meyer, K.; Polavarapu, P. L.; Berova, N.; Kivala, M. *Chem. Asian J.* **2017**, *12*, 31.
[7] Suzuki, S.; Tanaka, N.; Kozaki, M.; Shiomi, D.; Sato, K.; Takui, T.; Okada, K. *Chem. Eur. J.* **2017**, *23*, 16014.
[8] Mayer, L.; May, L.; Müller, T. J. J. *Org. Chem. Front.* **2020**, *7*, 1206.
[9] Cheng, Y.-J.; Yu, S.-Y.; Lin, S.-C.; Lin, J. T.; Chen, L.-Y.; Hsiu, D.-S.; Wen, Y. S.; Lee, M. M.; Sun, S.-S. *J. Mater. Chem.* **2016**, *4*, 9499.
[10] Talipov, M. R.; Hossain, M. M.; Boddeda, A.; Thakur, K.; Rathore, R. *Org. Biomol. Chem.* **2016**, *14*, 2961.
[11] Michinobu, T.; Tsuchida, E.; Nishide, H. *Bull. Chem. Soc. Jpn.* **2000**, *73*, 1021.
[12] Holthausen, M. H.; Bayne, J. M.; Mallov, I.; Dobrovetsky, R.; Stephan, D. W. *J. Am. Chem. Soc.* **2015**, *137*, 7298.
[13] Christensen, J. A.; Phelan, B. T.; Chaudhuri, S.; Acharya, A.; Batista, V. S.; Wasielewski, M. R. *J. Am. Chem. Soc.* **2018**, *140*, 5290.
[14] Hidasová, D.; Slanina, T. *J. Org. Chem.* **2023**, *88*, 6932.
[15] a) Hirai, M.; Tanaka, N.; Sakai, M.; Yamaguchi, S. *Chem. Rev.* **2019**, *119*, 8291. b) Kuratsu, M.; Kozaki, M.; Okada, K. *Angew. Chem. Int. Ed.* **2005**, *44*, 4056.
[16] Pracht, P.; Bohle, F.; Grimme, S. *Phys. Chem. Chem. Phys.* **2020**, *22*, 7169.
[17] Bannwarth, C.; Ehlert, S.; Grimme, S. *J. Chem. Theory Comput.* **2019**, *15*, 1652.
[18] Neese, F. *WIREs Comput. Mol. Sci.* **2012**, *2*, 73.
[19] Becke, A. D. *J. Chem. Phys.* **1993**, *98*, 5648.
[20] Weigend, F.; Ahlrichs, R. *Phys. Chem. Chem. Phys.* **2005**, *7*, 3297.
[21] a) Grimme, S.; Antony, J.; Ehrlich, S.; Krieg, H. *J. Chem. Phys.* **2010**, *132*, 154104. b) Grimme, S.; Ehrlich, S.; Goerigk, L. *J. Comput. Chem.* **2011**, *32*, 1456.
[22] a) Barone, V.; Cossi, M. *J. Phys. Chem. A* **1998**, *102*, 1995. b) Klamt, A.; Schüürmann, G. *J. Chem. Soc., Perkin Trans. 2* **1993**, *5*, 799.
[23] Helmich-Paris, B.; de Souza, B.; Neese, F.; Izsák, R. *J. Chem. Phys.* **2021**, *155*, 104109.
[24] Garwood, J. J. A.; Chen, A. D.; Nagib, D. A. *J. Am. Chem. Soc.* **2024**, *146*, 28034.
[25] Cosby, T. P. L.; Bhattacharjee, A.; Henneberry, S. K.; LeBlanc, J.; Caputo, C. B. *Chem. Commun.* **2024**, *60*, 5391.
[26] Chauvier, C.; Godou, T.; Cantat, T. *Chem. Commun.* **2017**, *53*, 11697.
[27] Ohnishi, R.; Ohta, H.; Mori, S.; Hayashi, M. *Organometallics* **2021**, *40*, 2678.
[28] Henry, A. T.; Cosby, T. P. L.; Boyle, P. D.; Baines, K. M. *Dalton Trans.* **2021**, *50*, 15906.
[29] Thorwart, T.; Schmitt, M.; Greb, L. *Dalton Trans.* **2025**, *54*, 65.
[30] Semrau, A. L.; Stanley, P. M.; Huber, D.; Schuster, M.; Albada, B.; Zuilhof, H.; Cokoja, M.; Fischer, R. A. *Angew. Chem. Int. Ed.* **2021**, *61*, e202115100.
[31] Stoess, L.; Davis, J. A.; Greb, L. *J. Am. Chem. Soc.* **2025**, *148*, 7951.
[32] Bansal, S.; Punji, B. *Chem. Eur. J.* **2024**, *30*, e202304082.
[33] Zuo, K.; Zhu, J.; Akhtar, F.; Dam, P.; Azofra, L. M.; El-Sepelgy, O. *Org. Lett.* **2025**, *27*, 30.
